# A sustainable synthesis of the SARS-CoV-2 M^pro^ inhibitor nirmatrelvir, the active ingredient in Paxlovid

**DOI:** 10.1038/s42004-022-00758-5

**Published:** 2022-11-21

**Authors:** Joseph R. A. Kincaid, Juan C. Caravez, Karthik S. Iyer, Rahul D. Kavthe, Nico Fleck, Donald H. Aue, Bruce H. Lipshutz

**Affiliations:** Department of Chemistry and Biochemistry, University of California, Santa Barbara, CA 93106 USA

**Keywords:** Asymmetric synthesis, Medicinal chemistry, Synthetic chemistry methodology

## Abstract

Pfizer’s drug for the treatment of patients infected with COVID-19, Paxlovid, contains most notably nirmatrelvir, along with ritonavir. Worldwide demand is projected to be in the hundreds of metric tons per year, to be produced by several generic drug manufacturers. Here we show a 7-step, 3-pot synthesis of the antiviral nirmatrelvir, arriving at the targeted drug in 70% overall yield. Critical amide bond-forming steps utilize new green technology that completely avoids traditional peptide coupling reagents, as well as epimerization of stereocenters. Likewise, dehydration of a primary amide to the corresponding nitrile is performed and avoids use of the Burgess reagent and chlorinated solvents. DFT calculations for various conformers of nirmatrelvir predict that two rotamers about the tertiary amide would be present with an unusually high rotational barrier. Direct comparisons with the original literature procedures highlight both the anticipated decrease in cost and environmental footprint associated with this route, potentially expanding the availability of this important drug worldwide.

## Abstract (web summary)

The antiviral drug nirmatrelvir is the active ingredient in Paxlovid, a SARS-CoV-2 M^pro^ inhibitor developed by Pfizer, however, its synthetic procedure faces limitations for meeting urgent demand. Here, the authors optimize the key synthetic steps and develop a sustainable route to nirmatrelvir in 7 steps and 3 pots with an overall 70% yield.

## Introduction

The ongoing COVID-19 pandemic caused by the SARS-CoV‑2 virus and its variants represents a once in a century public health disaster that continues to impact mankind, as well as the global economy^1–4^. The disease has created an urgent need for rapid development of both preventative measures (e.g., vaccines) and post-infection treatment options^5^. Both aims have been achieved in record time, the latter initially being driven by investigations into repurposing existing drugs, which has produced relatively few effective treatment options^6,7^. The search for novel SARS-CoV-2 antivirals, on the other hand, has led to the development of molnupiravir by Merck^8^, EDP-235 by Enanta^9^, S-217622 by Shionogi^10^, and several others^11–14^. The first such antiviral to receive emergency approval by the FDA, Paxlovid, is an orally-active combination of the SARS-CoV-2 main protease inhibitor nirmatrelvir (**1**; PF‑07321332) and the HIV antiviral ritonavir, disclosed by Pfizer in late 2021^15,16^. This drug combination was shown to reduce risk of progression to severe COVID-19 in high-risk, symptomatic patients by 89% compared to placebo^17^, exemplifying the crucial role the drug is expected to play worldwide in the continuing efforts to combat the COVID-19 pandemic.

In addition to cost, the environmental impact associated with the synthesis of nirmatrelvir in such an immediate and high demand situation cannot be overlooked, especially since the prescribed dosage is 3 g of total API per patient over the course of the 5-day treatment. While Pfizer’s more recently reported route to nirmatrelvir^18^ improves upon their originally reported methodology^15^, there are still numerous opportunities to reduce the amount of waste generated by the existing routes, especially focusing on the variety of organic solvents used, and peptide coupling reagents. Therefore, there clearly exists an urgent need for the development of both a green and economically attractive synthesis of nirmatrelvir, especially for its distribution in the third world.

**Fig. 1:**
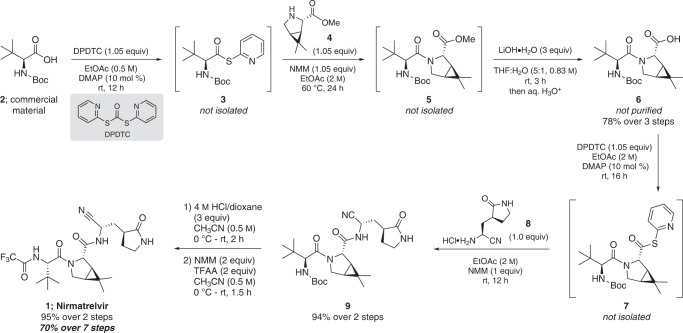
Overall route to nirmatrelvir featuring thioester intermediates 3 and 7 en route to peptides 5 and 9, respectively. The final conversion of **9** to nirmatrelvir (**1**) requires only *N*-Boc deprotection and trifluoroacetylation.

In continuing our group efforts to develop scalable routes to APIs under cost effective and environmentally friendly conditions^19–21^, and in an ongoing partnership with the Bill and Melinda Gates Foundation initially formed for purposes of preparing APIs for the treatment of malaria (e.g., pyronaridine)^19^, we have developed a route to nirmatrelvir that simultaneously addresses these issues while maximizing both time and pot economies (Fig. 1)^22,23^. Furthermore, special attention has been directed toward avoiding epimerization of chiral centers during crucial peptide bond-forming steps. Workups, which often give rise to enormous volumes of both organic and aqueous waste, have also been streamlined; only simple, in-pot aqueous washes are involved, as are minimal amounts of far greener (and recoverable) organic solvents (e.g., EtOAc)^24,25^. The strategy selected for the synthesis of nirmatrelvir, therefore, focused on the inherent benefits on scale of a convergent route^26^. Moreover, it seemed advantageous to perform our palladium-catalyzed amide dehydration^27^ in this convergent fashion, as subsequent steps provide opportunities to limit the amount of residual palladium in the final API.

## Results and discussion

The 1-pot thioesterification/amide bond formation featured in this sequence (details surrounding the development of this technology will be disclosed in due course), which uses di-2-pyridyldithiocarbonate (DPDTC)^28–30^ to activate the carboxylic acid, avoids traditional peptide coupling reagents (e.g., HATU, DCC, COMU, etc.) and epimerization, and allows for facile removal of the (odorless) 2-mercaptopyridine by-product via an in-flask extraction with limited amounts of aqueous hydroxide. In contrast to the by-products of conventional amide bond coupling reagents, as used in the Pfizer route^18^ and which are known to be genotoxic,^19,20^^,^^31,32^ benign 2-mercaptopyridine formed can be easily recovered for recycling to DPDTC (see SI, section 4). Both intermediate thioesters **3** and **7** are stable, isolable species constituting activated carboxylic acids, potentially simplifying their large-scale manufacture and distribution compared to other activated acid derivatives. In this sequence, however, their individual isolation/purification was not required.

### 3-Step, 1-pot sequence en route to intermediate 6

Commercially available *N*-Boc protected *t*-leucine (**2**) was converted to its thioester derivative **3** using DPDTC in environmentally preferrable EtOAc^24,25^ containing catalytic DMAP at rt. Product **3** was then processed via simple in-flask treatment with aqueous base, followed (after removal of the aqueous phase and removal of EtOAc in vacuo) by addition of bicyclic proline **4**, *N*-methylmorpholine (NMM), and EtOAc (the academic scale at which this route was developed necessitated dilution of the reaction medium with EtOAc prior to aqueous extraction. This accounts for the EtOAc being concentrated following aqueous washes, and then being freshly added to bring the concentration to the desired level for use in the following step. Such handling would not be involved on scale up). Gentle heating led to the desired amide bond in product **5**. This newly formed peptide containing a methyl ester originally present in **4** was then hydrolyzed with LiOH in aqueous THF, after which the reaction mixture was neutralized with aqueous HCl and then extracted with minimal EtOAc to afford **6** (78% over 3 steps). It is noteworthy that the product did not require purification since the small amounts of impurities present, primarily 2,2’‑dipyridyldisulfide, were of no synthetic consequence. It was, however, necessary to adjust the stoichiometry of reagents used in the second thioesterification step based on the amounts of impurities present in crude **6** (as determined by quantitative NMR). Optimization of the thioesterification and amide bond-forming steps can be found in the SI, Sections 3.1 and 3.2.

### 2-Step, 1-pot sequence en route to intermediate 9

Carboxylic acid **6** was subjected to thioester formation in the same manner as used earlier to form **3**. The resulting **7**, without isolation, was then treated with nitrile amine salt **8** in concentrated EtOAc (2 M) at rt to afford **9** (94% over 2 steps). This convergent approach avoids the route used by Pfizer involving a primary amide intermediate that then requires the Burgess reagent to effect dehydration. While column chromatography was required in our hands for isolation of pure **9** owing to practical limitations associated with small-scale academic work, large-scale purification of this intermediate may be possible by precipitation.

### 2-Step, 1-pot sequence en route to nirmatrelvir (1)

**Fig. 2:**
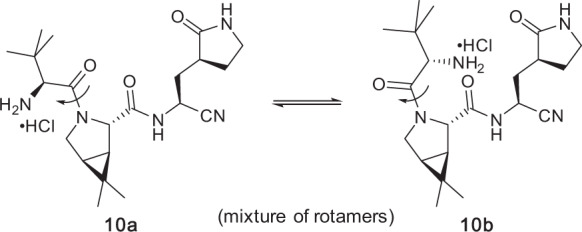
The rotameric mixture associated with intermediate amine salts 10a and 10b, obtained following *N*-Boc deprotection. Both rotamers, and the relative amounts present, can be seen by variable temperature ^1^H NMR.

Intermediate **9** was initially dried azeotropically using recoverable toluene. *N*-Boc Deprotection was then carried out using a concentrated solution of HCl/dioxane in CH_3_CN. All attempts using standard conditions involving TFA were quite low-yielding and involved creation of multiple side-products, presumably including various materials resulting from epimerization due to the presence of TFA (we found that exposing pure nirmatrelvir to TFA led to 8% epimerization by chiral HPLC analysis, whereas exposure to either excess base (NMM) or HCl did not lead to epimerization (see SI Section 3.3, especially Fig. S9)). Use of HCl, however, was very clean and led to an initial mixture of conformers, likely to be a rotameric mix associated with the tertiary amide present (**10a** and **10b**, Fig. 2) as seen by Pfizer with related compounds bearing the same bicyclic proline moiety^15^, and supported by variable temperature ^1^H NMR (see SI, Section 3.7). Remarkably, and unexpectedly, it was also observed that greater degrees of epimerization occurred during subsequent trifluoroacetylation when the kinetically formed proportion of the minor conformer was used. Interestingly, equilibration of the *N*-Boc deprotected material, which was a 70:30 mixture of the initially formed HCl salt, at rt overnight reproducibly led to a ratio of conformers between 94:6 and 96:4. After equilibration, the solvent and excess HCl were removed in vacuo and the resulting amine hydrochloride salt was subjected to trifluoroacetic anhydride (TFAA) and NMM to install the trifluoroacetamide moiety. Removal of TFAA and NMM via aqueous washes afforded nirmatrelvir (**1**) in 95% yield over this 1-pot, 2-step approach, and with 95% purity by HPLC. Small amounts of the undesired diastereomer could be effectively removed by formation and recrystallization of the MTBE solvate, as described previously by Pfizer^15^. The resulting nirmatrelvir was isolated, seemingly as a pure rotamer based on NMR data (see SI, Fig. S8). Data relating to optimization and characterization of intermediates and products in this 2-step procedure can be found in the SI, Section 3.3.

**Fig. 3:**
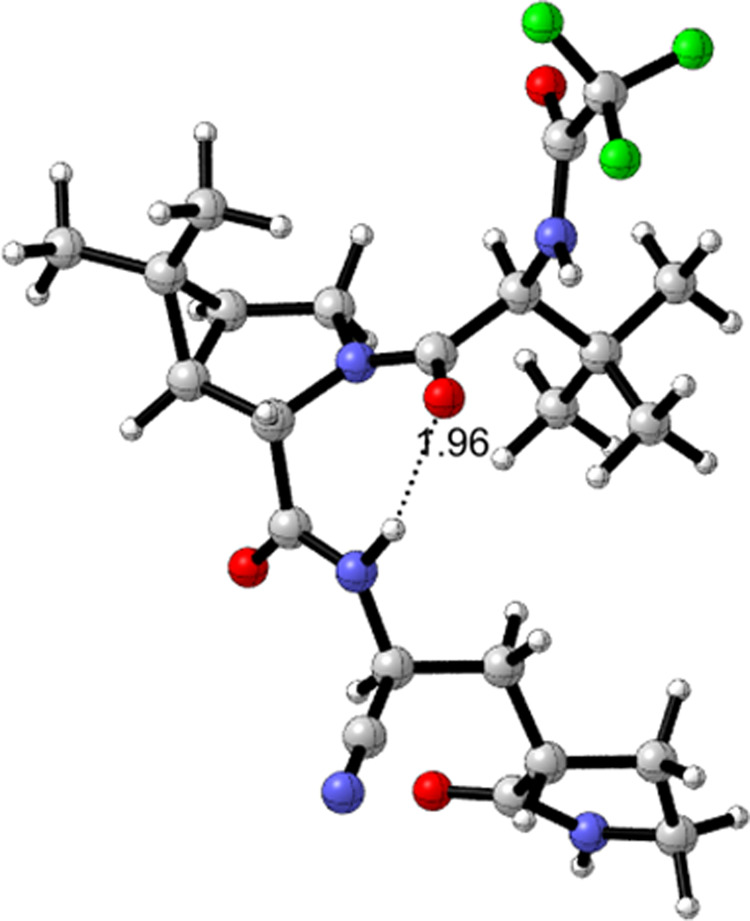
B3LYPD3BJ/6-31+G(d,p)/SMD(MeCN) optimized structure in acetonitrile for the major rotamer of 1. Hydrogen bond O-H distance = 1.96 Å. Atom colors: nitrogen, blue; oxygen, red; fluorine, green.

**Fig. 4:**
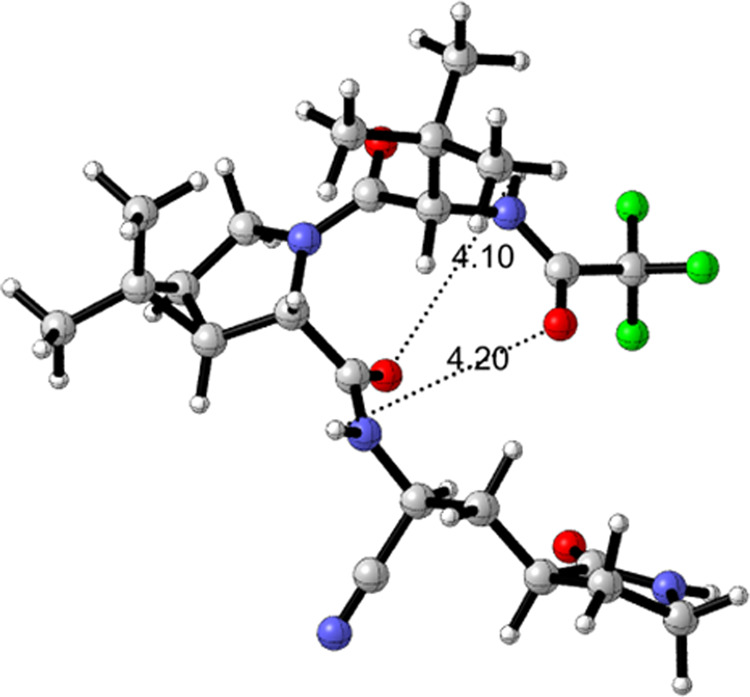
B3LYPD3BJ/6-31+G(d,p)/SMD(MeCN) optimized structure in acetonitrile for the minor rotamer of 1. Weak hydrogen bond O-H distances = 4.10, 4.20 Å. Atom colors: nitrogen, blue; oxygen, red; fluorine, green.

The proton NMR spectrum of nirmatrelvir (**1**) (obtained prior to treatment with MTBE) in dimethyl sulfoxide solvent showed mainly one rotamer, with only ca. 5% of the minor rotamer present. DFT calculations on conformers of nirmatrelvir were carried out looking to support observations that nirmatrelvir and related compounds featuring the bicyclic proline residue in **1** exist as two rotamers about the tertiary amide bond that are unusually slow to equilibrate at room temperature as seen in the NMR spectrum (see SI Section 6 and Supplementary Data 1). Calculations at the B3LYPD3BJ/6-31+G(d,p) level in the gas phase and in acetonitrile using the SMD solvation model showed a predominance of the rotamer with the tertiary amide carbonyl group participating in a cyclic, 7-membered ring containing a hydrogen bond to the NH of the pyrrolidinone side-chain amide (Fig. 3). The structure of the minor rotamer (calculated in acetonitrile) is shown in Fig. 4 with two marginal long-distance, non-linear hydrogen bonds. The rotational free energy barrier for nirmatrelvir in acetonitrile was calculated to be 25.25 kcal mol^−1^ at 298 K, significantly higher than the experimental barrier of 17.77 kcal mol^−1^ for acetamide in acetonitrile solvent^33^.

### Synthesis of intermediate 8

**Fig. 5:**
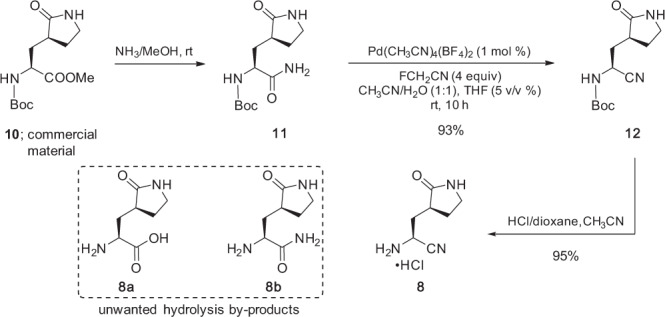
Preparation of aminonitrile 8 from readily available starting material 11. This amine is crucial for the second peptide bond-forming step leading to the targeted drug, nirmatrelvir (**1**).

The commercially available *N*-Boc protected methyl ester **11** was converted to the corresponding primary amide **12** using the published procedure (methanolic ammonia; Fig. 5)^15^. Although Pfizer’s approach to the dehydration of their primary amide relied on the Burgess reagent in chlorinated solvent^15^, **12** could alternatively be smoothly dehydrated applying recently disclosed technology based on an “amide exchange”^27,34^; that is, using commercially available fluoroacetonitrile as the sacrificial acceptor of water under palladium-catalyzed conditions, resulting in nitrile **13** (93%). It is appreciated that fluoroacetonitrile may be undesirable owing to both its cost and the toxicity of the fluoroacetamide by-product. Crystallization of educt **13** is sufficient, however, to remove this impurity. Should an alternative be desirable on scale, methoxyacetonitrile is a viable choice for further development (see SI, Table S6, entry 23). Data relating to the optimization of this amide dehydration are presented in the SI, Section 3.4.

*N*-Boc Deprotection of **13** using HCl in various organic solvents was initially problematic in that adventitious water present in both the solvent and starting material **13** led to varying degrees of hydrolysis (13–30+%) to form carboxylic acid **8a** and/or primary amide **8b**. It was anticipated that hydrolysis could also occur during Boc-deprotection of **9**. Formation of by-products **8a** and **8b** were minimized via azeotropic removal of residual water in **13** using recoverable toluene under high vacuum. Prior observations by BMS on related nitriles indicated that inclusion of a sacrificial nitrile, such as CH_3_CN, reduced undesired competitive hydrolysis during *N*-Boc deprotection, likewise under acidic conditions^35^. Indeed, applying both procedures (azeotropic drying of the educt and then adding dry CH_3_CN) afforded the desired nitrile amine **8** as its HCl salt (95%) with only 3% hydrolysis. Separation and removal of residual **8a** and/or **8b** could be easily accomplished by dissolving the mixture in MeOH and precipitating pure **8** using ice-cold Et_2_O. Optimization of this *N*-Boc deprotection methodology is presented in the SI, Section 3.5.

Importantly, notwithstanding the use of catalytic palladium in the synthesis of intermediate **13**, the existence of subsequent steps en route to the final API (**1**) provided multiple opportunities for removal of this metal. It was anticipated, therefore, that the amount of residual palladium would be below the acceptable FDA limit of 10 ppm/dose/day^36^. The level of residual palladium in nirmatrelvir **1** synthesized using this route, as determined by ICP-MS was found to be below the detectable limit.

This convergent route to nirmatrelvir has been accomplished in 70% overall yield, a considerable improvement over the 48% reported by Pfizer. Table 1 summarizes a direct comparison of several additional key features associated with each route with respect to environmental aspects, as well as potential costs. Importantly, given the global volumes of drug needed (leading to a projected $22 billion in revenue)^37,38^, and using Sheldon’s E Factors^39,40^ as a reasonable guide to waste creation, the sequence described herein is suggestive of a far more attractive process (see the SI, Section 5 for E Factor calculations). What the E Factors do not highlight, however, is the significant decrease in hazardous waste generated, including chlorinated solvents, DMF, and excess reagents such as HATU and the Burgess reagent.

**Table 1:** Comparisons between this work and Pfizer’s route^15^ to nirmatrelvir (**1**).

Reaction parameter	Pfizer	This work
Amide bond formations	Uses HATU, EDCI, non-recyclable solvents: DMF, MEK	Uses DPDTC, recyclable solvent: EtOAc
Amide dehydration	Burgess reagent, solvent: CH_2_CI_2_	cat. Pd, FCH_2_CN, medium: H_2_O/CH_3_CN
*N-*Boc deprotection	Solvent: CH_2_CI_2_	Solvents: CH_3_CN, dioxane
E Factor (all waste)	214	120^a^
E Factor (excluding aq. waste)	108	54^a^
Overall yield	48%	70%

^a^Artificially elevated due to scale; the actual E Factor of this sequence would likely be much lower on scale, as dilution of the reaction medium would not be necessary. Also the academic scale at which this route was developed necessitated dilution of the reaction medium with EtOAc prior to aqueous extraction. This accounts for the EtOAc being concentrated following aqueous washes, and then being freshly added to bring the concentration to the desired level for use in the following step. Such handling would not be involved on scale up.

It is appreciated that Pfizer has altered its initial route to nirmatrelvir, as disclosed in a recent patent filing^18^. However, full experimental details were not provided for all steps, unlike requirements associated with journal publications. Therefore, it was not possible to make direct comparisons involving their second-generation route. An alternative route laid out in the patent which does include all pertinent experimental details, and does follow a related convergent route to that disclosed in the present work, leads to an overall yield of 7.6%. This is due to a 50% yield in the first amide bond formation, and 17% yield in the final Boc/trifluoroacetamide exchange. Hence, we opted to make the comparisons shown in Table 1 using their best results^15^.

## Conclusions

The first potentially scalable and economically attractive sequence to nirmatrelvir for consideration by companies interested in providing this drug, at scale, to the third world is outlined herein. It provides streamlined, efficient, convergent, and environmentally responsible access to the key ingredient in Paxlovid for treatment of COVID-19. Particularly notable features that distinguish it from prior art include:

- Peptide bond constructions that take place in 1-pot processes in highly concentrated EtOAc that completely avoid traditional peptide coupling reagents in both peptide bond-forming steps, which can otherwise be costly, dangerous, and produce considerable waste, especially at scale.
- Use of our newly developed, green technology for primary amide dehydrations, applied to a “real” molecule, that by-passes employment of unattractive reagents at scale (e.g., the Burgess reagent).
- Conditions that avoid potentially costly separation of unwanted isomers due to epimerization.
- A significantly reduced environmental footprint, thereby avoiding much of the waste being generated by the currently utilized routes.
- New insights gained regarding the identity of major and minor tertiary amide rotamers in nirmatrelvir, and their unusually high rotational barrier, based on previously unknown DFT calculations.

## Methods

Characterization of the synthesized products are presented in the Supplementary Information (Supplementary Note 1: Experimental Data). NMR spectra are presented in Supplementary Data 2,

### Synthesis of di-2-pyridyldithiocarbonate (DPDTC)

All glassware was flame dried. To a 500 ml round-bottom flask equipped with a PTFE-coated magnetic stir bar was added 2-mercaptopyridine (6 equiv, 60 mmol, 6.67 g), then the flask was sealed with a rubber septum and anhydrous acetone (100 ml) was added via syringe under a positive flow of argon, followed by anhydrous Et_3_N (6 equiv, 60 mmol, 8.36 ml) and the solution was stirred until all components were fully dissolved. An ice bath was used to cool the resulting solution, an argon balloon was affixed to the septum via a needle, then a solution of triphosgene (1 equiv, 10 mmol, 2.967 g) in acetone (12.5 ml) was slowly added over the course of 15 min. The ice was replaced as needed to keep the solution cool during addition of triphosgene to prevent excessive generation of phosgene gas. Upon full addition, triethylammonium chloride was observed to precipitate. The reaction was allowed to warm to room temperature and stir overnight. Upon completion, the septum was removed inside a fume hood and allowed to expel any excess phosgene gas, then the reaction mixture was filtered to remove triethylammonium chloride, and the filtrate was concentrated in vacuo to afford a crude oil containing crystals of remaining triethylammonium chloride. The crude residue was redissolved in EtOAc in 10 ml portions and filtered into a separatory funnel. The combined organic extracts were washed with saturated aqueous NaHCO_3_ (100 ml), followed by DI water (100 ml), followed by saturated brine (100 ml). The organic layer was separated and dried over anhydrous Na_2_SO_4_, filtered, and concentrated in vacuo and residual solvent was removed under high vacuum overnight to afford DPDTC as a light-yellow solid (6.596 g, 89% yield).

Caution: Triphosgene is acutely toxic and releases toxic phosgene gas on contact with moisture. It should be handled on small scale in a fume hood or glove box and weighed out using a pre-weighed, tightly sealed container.

Note: Commercial sources of inexpensive 2-mercaptopyridine may require purification prior to use due to the presence of the derived disulfide. This can be accomplished via recrystallization from EtOAc.

### Thioesterification en route to thioester 3

To a 1-dram vial equipped with a PTFE-coated magnetic stir bar, was added Boc-Tle-OH (2; 1 equiv, 0.25 mmol, 57.8 mg), DPDTC (1.05 equiv, 0.263 mmol, 65.2 mg), and DMAP (10 mol%, 3.1 mg). The reaction mixture was dissolved in EtOAc (0.5 M, 0.5 ml) and stirred at rt for 12 h. Upon completion (by TLC), the reaction mixture was washed with 1.25 ml of 5% aqueous KHSO4 to remove DMAP, then twice with 1.25 ml of 1 M NaOH solution to remove 2-mercaptopyridine. The organic layer was concentrated to afford 3 (73.0 mg, 90% yield, 99.6% ee) as an off-white solid. This material was used in the next step without further purification. Alternatively, thioester 3 could be purified by flash column chromatography (25–30% EtOAc/hexanes).

### Amide bond coupling en route to dipeptide 5

To a 1-dram vial equipped with a PTFE-coated magnetic stir bar was added thioester 3 (1 equiv, 0.5 mmol, 162.2 mg), free-based amine 4 (1.05 equiv, 0.525 mmol, 88.8 mg), EtOAc (0.25 ml leading to a 2 M solution) and *N*-methylmorpholine (NMM; 1.05 equiv, 0.525 mmol, 58 μl). The reaction was stirred at 60 °C for 24 h, with completion being monitored by TLC (consumption of thioester). The crude material was purified by flash column chromatography (15–20%, EtOAc/hexanes) to afford 5 (172.0 mg, 90% yield, >99:1 dr) as a colorless oil.

### Ester hydrolysis to afford carboxylic acid 6

To a 3-dram vial equipped with a PTFE-coated magnetic stir bar was added 5 (1 equiv, 0.79 mmol, 303.9 mg) and LiOH•H_2_O (3 equiv, 2.38 mmol, 100 mg), followed by THF (0.8 ml) and water (0.2 ml). The reaction vial was capped and stirred for 3 h at rt. Upon completion, the reaction mixture was concentrated in vacuo to remove most of the THF, then acidified to pH 2 using a 1 M aqueous solution of HCl. The aqueous phase was extracted with EtOAc (3 × 3 ml), and the combined organic layers were washed with brine, dried over anhydrous Na_2_SO_4_, and then concentrated in vacuo to afford carboxylic acid 6 (268 mg, 92% yield) as a white solid. This material was used without further purification.

### Thioesterification to afford thioester 7

To a 2-dram vial equipped with a PTFE-coated magnetic stir bar was added 6 (1 equiv, 1 mmol, 368.5 mg), DPDTC (1.05 equiv, 1.05 mmol, 260.7 mg), and DMAP (10 mol%, 12.2 mg). The reaction mixture was dissolved in EtOAc (2 M, 0.5 ml) and stirred at rt for 16 h. Upon completion (by TLC), the reaction mixture was diluted with 2 ml EtOAc then washed with 3 ml of 5% aqueous KHSO_4_ to remove DMAP, then twice with 3 ml 1 M NaOH aqueous solution to remove 2-mercaptopyridine. The organic layer was concentrated in vacuo to afford 7 (409 mg, 89% yield) as a light-yellow solid. This material was used in the subsequent step without further purification.

### Amide bond coupling to afford tripeptide 9

To a 1-dram vial equipped with a PTFE-coated magnetic stir bar was added thioester 7 (1 equiv, 0.431 mmol, 198.9 mg) and amine hydrochloride salt 8 (1 equiv, 0.431 mmol, 81.7 mg), followed by EtOAc (2 M, 215 μl) and *N*-methylmorpholine (NMM; 1 equiv, 0.431 mmol, 47 μl). The vial was then capped and allowed to stir at rt for 12 h. Upon completion (as determined by TLC, consumption of thioester), the reaction mixture was diluted with EtOAc (0.2 ml) and washed with 5% KHSO_4_ (0.2 ml) to remove NMM, followed by 1 M NaOH (2 × 0.2 ml) to remove 2-mercaptopyridine, and finally the mixture was washed with water (0.2 ml) to remove residual NaOH in order to prevent hydrolysis of the nitrile moiety as the organic layer was concentrated in vacuo to afford tripeptide 9 (200.8 mg, 93% yield) as a white solid. This material was used without further purification. If desired, material could be purified by flash column chromatography (2% MeOH/CH_2_Cl_2_).

#### 3-Step, 1-pot sequence to afford carboxylic acid 6

(Step 1) Thioesterification to afford thioester 3: to a 2-dram vial equipped with a PTFE-coated magnetic stir bar, was added Boc-Tle-OH (2; 1 equiv, 1 mmol, 231.3 mg), DPDTC (1.05 equiv, 1.05 mmol, 260.7 mg), and DMAP (10 mol%, 0.1 mmol, 12.2 mg). The mixture was dissolved in EtOAc (0.5 M, 2 ml) and stirred at rt for 12 h. Upon completion (by TLC), the reaction mixture was washed twice with 5% aqueous KHSO_4_ (2 × 1.5 ml) to remove DMAP, then with 1 M NaOH (2 × 1.5 ml) to remove 2‑mercaptopyridine. The organic layer was concentrated to afford 3. This material was used in the next step without further purification.

(Step 2) Amide bond formation to afford dipeptide 5: the material from the previous step was subjected to the free-based amine 4 (1.05 equiv, 1.05 mmol, 177.7 mg), EtOAc (2 M, 0.5 ml) and *N*-methylmorpholine (NMM; 1.05 equiv, 1.05 mmol, 115 µl). The reaction was stirred at 60 °C for 24 h, with reaction progress being monitored by TLC (consumption of thioester). Upon completion, the reaction mixture was first diluted with 1.5 ml of EtOAc, then washed with 1 M HCl (2 × 1.5 ml) to remove unreacted amine and NMM, then with 1 M NaOH (2 × 1.5 ml) to remove 2-mercaptopyridine. The organic layer was concentrated to afford 5. This material was used in the next step without further purification.

(Step 3) Ester hydrolysis to afford carboxylic acid 6: the material from the previous step was subjected to LiOH•H_2_O (3 equiv, 3 mmol, 126 mg), followed by THF (1 ml) and water (0.2 ml). The reaction vial was capped and stirred for 3 h at rt. Upon completion, the reaction mixture was concentrated in vacuo to remove most of the THF, then acidified to pH 2 using a 1 M aqueous solution of HCl. The aqueous phase was extracted with EtOAc (3 × 1 mL), then the combined organic layers were concentrated in vacuo to afford the crude carboxylic acid 6 as a light-yellow powder (78% yield, 310 mg, over 3 steps). This crude material was used in the subsequent step. Quantitative NMR analysis using 1,3,5-trimethoxy-benzene as internal standard showed that the material was 93% pure. The yield shown above was adjusted to account for the 7% impurity.

#### 2-Step, 1-pot sequence to afford tripeptide 9

(Step 4) Thioesterification to afford thioester 7: a portion of the material from the first pot was used, and the mass of carboxylic acid 6 was adjusted to account for purity. To a 2-dram vial equipped with a PTFE-coated magnetic stir bar was added 6 (93% purity; 1 equiv, 0.465 mmol, 184.2 mg), DPDTC (1.05 equiv, 0.488 mmol, 121.2 mg), and DMAP (10 mol%, 48.8 μmol, 5.7 mg). The reaction mixture was dissolved in EtOAc (232 μl, making a 2 M solution) and stirred at rt for 16 h. Upon completion (by TLC), the reaction mixture was diluted with 1.5 ml EtOAc and washed with 5% aqueous KHSO_4_ (2 × 0.5 ml) to remove DMAP. The organic layer was concentrated in vacuo to afford crude material 7. This material was used in the subsequent step without further purification.

(Step 5) Amide bond coupling to afford tripeptide 9: to the crude material from step 4 above was added the amine hydrochloride salt 8 (1 equiv, 0.465 mmol, 88.2 mg) followed by EtOAc (2 M, 232 μl) and *N*-methylmorpholine (NMM; 1 equiv, 51 µl). The vial was then capped and allowed to stir at rt for 12 h. Upon completion (as determined by TLC, consumption of thioester), the reaction mixture was diluted with EtOAc (1 ml), washed with 5% KHSO_4_ (2 × 0.5 ml) to remove NMM, followed by 1 M NaOH (2 × 0.5 ml) to remove 2-mercaptopyridine, and finally the mixture was washed with water (0.5 ml) to remove residual NaOH to prevent hydrolysis of the nitrile moiety when the organic layer was concentrated in vacuo and subjected to flash chromatography (2% MeOH/CH_2_Cl_2_) to afford tripeptide 9 (221.1 mg, 94% yield) as a white solid.

#### 2-Step, 1-pot *N-*Boc deprotection/trifluoroacetylation to afford nirmatrelvir (1)

(Step 6) *N*-boc deprotection: starting material 9 was azeotropically dried by suspending it in anhydrous toluene and removing solvent in vacuo at rt a total of three times. To a flame-dried 1-dram vial equipped with a PTFE-coated magnetic stir bar was added tripeptide 9 (1 equiv, 0.5 mmol, 251.8 mg) and anhydrous CH_3_CN (0.5 M, 1 ml). The reaction mixture was cooled in an ice bath, then 4 M HCl/dioxane (3 equiv, 1.5 mmol, 375 μl) was added dropwise via syringe with slow stirring so as to avoid splashing. The resulting clear, colorless solution was allowed to warm to rt (ca. 21 °C) and stir for 2 h, whereupon the product precipitated as a white solid. The reaction mixture was removed from stirring and allowed to stand at rt for 16 h to equilibrate the mixture of rotamers (see SI Section 3.3). The reaction mixture was then placed in a freezer for 1 h prior to removal of the solvent in vacuo. The white solid was washed with Et_2_O (3 × 1 ml) and collected via centrifugation and removal of the supernatant via syringe. The amine hydrochloride salt was obtained as a white powder following drying under high vacuum (218.2 mg, >99% yield). The material was used without purification.

(Step 7) Trifluoroacetylation: to the amine hydrochloride salt obtained in step 6 was added anhydrous CH_3_CN (0.5 M, 1 ml) and the mixture was chilled in an ice bath. Trifluoroacetic anhydride (TFAA; 2 equiv, 1 mmol, 139 μl) was added, followed by dropwise addition of *N*-methylmorpholine (NMM; 2 equiv, 1 mmol, 110 μl) and the reaction was allowed to warm to rt and stir for 1.5 h. The solvent was removed in vacuo followed by addition of 5% aqueous KHSO_4_ (2 ml) and the mixture was centrifuged. The supernatant was removed via syringe, and the residue was washed twice with water (2 × 1 ml) and filtered to afford crude nirmatrelvir as an off-white solid (1; 237.8 mg, 95% yield over 2 steps). This material contained 3% of an undesired diastereomer and showed an overall purity of 95% (as determined by chiral HPLC; see ESI section 1, HPLC method 2; see Fig. S3). The unwanted diastereomer could be removed either by column chromatography or formation and recrystallization of an MTBE solvate (see SI Section 3.3). Nirmatrelvir 1 was obtained in 70% overall yield over 7 steps in 3 pots, and contained no detectable residual palladium, as determined by ICP-MS.

### Dehydration of primary amide 12 to nitrile 13

A 1-dram vial equipped with a PTFE-coated magnetic stir bar was placed in a glovebox under an atmosphere of Ar into which was added Pd(CH_3_CN)_4_(BF_4_)_2_ (1 mol%, 0.01 mmol, 4.4 mg), after which the vial was capped and removed from the glovebox. To the vial was added primary amide compound 12 (1 equiv, 1 mmol, 271.3 mg), followed by a 1:1 mixture of MeCN and DI water (0.5 M, 2 ml), tetrahydrofuran (THF; 5 v/v %, 0.1 ml), and fluoroacetonitrile (FCH_2_CN; 4 equiv, 4 mmol, 223 μl). The vial was capped and allowed to stir on a magnetic stir plate at rt for 10 h. Upon completion, the reaction was directly dried onto 2 g of silica by use of a rotary evaporator and purified by flash chromatography (5% MeOH/CH_2_Cl_2_). Alternatively, the reaction mixture was separated into a biphasic mixture by addition of NaCl, then the organic layer was separated and concentrated in vacuo, and the crude residue was recrystallized from *i*PrOAc. Product nitrile 13 was obtained as a white solid (235.6 mg, 93% yield). No epimerization was observed by ^1^H NMR.

### *N*-Boc deprotection en route to amine hydrochloride salt 8

Starting material 13 was azeotropically dried by suspending it in anhydrous toluene and removing solvent in vacuo at <60 °C a total of three times. To a flame-dried 6-dram vial equipped with a PTFE-coated magnetic stir bar was added *N*-Boc protected nitrile 13 (1 equiv, 1.777 mmol, 450 mg) and anhydrous CH_3_CN (0.5 M, 3.55 ml) generating a cloudy suspension, after which the mixture was chilled in an ice bath. To the vial was added 4 M HCl/dioxane dropwise via syringe (3 equiv, 5.33 mmol, 1.33 ml) whereupon starting material immediately went into solution. The reaction was allowed to warm to rt and stir for 2 h. As the reaction progressed, product precipitated as a white solid, and was collected via filtration and washed with ice-cold Et_2_O to afford 8 (322.1 mg, 96% yield) as a white solid. If removal of hydrolysis by-products is required, the solid can be dissolved in minimal anhydrous MeOH with heating. The solution was then cooled to 0–5 °C in an ice bath, after which ice-cold Et_2_O was added dropwise until material stopped precipitating. The precipitate was collected via filtration and dried under vacuum to afford 8 free of hydrolysis by-products.

## Supplementary information

Supplementary Information

Supplementary Data 1

Supplementary Data 2

Description of Additional Supplementary Files

## Data Availability

The data that support the findings of this study are available from the corresponding author upon reasonable request. Experimental procedures, optimization details, analytical data (NMR, HPLC, and MS), and DFT calculations are included in the Supplementary Information (see Supplementary Note 1: Experimental Data). Computational studies are included in Supplementary Data 1. ^1^H, ^13^C, and ^19^F NMR spectra are presented in Supplementary Data 2.
