# Supplementary material for: A sustainable synthesis of the SARS-CoV-2 Mpro inhibitor nirmatrelvir, the active ingredient in Paxlovid
Source: Commun Chem. 2022 Nov 21;5:156. doi: 10.1038/s42004-022-00758-5 (PMC9685088; doi:10.1038/s42004-022-00758-5)
Supplement: Supplementary file 1 — Supplementary Information [file 42004_2022_758_MOESM1_ESM.pdf]

Supplementary Information

A Sustainable Synthesis of the SARS-CoV-2 M^{pro} Inhibitor Nirmatrelvir, the Active Ingredient in Paxlovid

Joseph R. A. Kincaid, Juan C. Caravez, Karthik S. Iyer, Rahul D. Kavthe, Nico Fleck, Donald H. Aue, and Bruce H. Lipshutz*

Department of Chemistry and Biochemistry, University of California, Santa Barbara, Santa Barbara, CA
93106 USA

Phone : 805-893-2521
Fax : 805-893-8265
Email: lipshutz@chem.ucsb.edu
Website: https://lipshutz.chem.ucsb.edu/

Table of Contents

Supplementary Methods S2
1. General information S2
2. Synthetic schemes S3
3. Synthetic Procedures S4
3.1 Optimization of the thioesterification to afford thioester **3** S4
3.2 Optimization of the amide bond coupling *en route* to dipeptide **5** S6
3.3 *N*-Boc-Deprotection / trifluoroacetylation to afford nirmatrelvir **1** S8
3.4 Optimization of the amide dehydration to afford nitrile **13** S16
3.5 *N*-Boc-Deprotection *en route* to amine hydrochloride salt **8** S18
4. Recovery of 2-mercaptopyridine for recycling purposes S21
5. E Factor calculations S21
6. Computational Studies S24
7. Supplementary References S32
8. Supplementary Note 1: Experimental data S34

Supplementary Methods

1. General information

Reagents:

Reagents were purchased from Sigma-Aldrich, Combi-Blocks, Ambeed Inc., Acros Organics, BLD Pharma, Fischer Scientific, or ChemScene and used without further purification unless noted otherwise.

Chromatography:

Silica gel TLC plates (UV 254 indicator, thickness 200 mm standard grade, glass backed and 230-400 mesh from Merck) were used. The developed TLC plate was analyzed by a UV lamp (254 nm). The plates were further analyzed with the use of an aqueous ceric ammonium molybdate stain or ethanolic vanillin and developed with a heat gun. Flash chromatography was performed using Silicycle Silicaflash® P60 unbonded grade silica.

NMR:

^{1}H, ^{13}C, and ^{19}F NMR were recorded at 25 ºC on either an Agilent Technologies 400 MHz, a Bruker Avance III HD 400 MHz, or a Varian Unity Inova 600 MHz spectrometer in $CDCl_3$, DMSO-d_6, CD_3OD, or CD_3CN with residual $CHCl_3$ (^{1}H = 7.26 ppm, ^{13}C = 77.16 ppm), DMSO (^{1}H = 2.54 ppm, ^{13}C = 40.45 ppm), CH_3OH (^{1}H = 3.31 ppm, ^{13}C = 49.15 ppm), and CH_3CN (^{1}H = 1.94 ppm, ^{13}C = 1.39 ppm) as the internal standard. Chemical shifts are reported in parts per million (ppm). The data presented will be reported as follows; chemical shift, multiplicity (s = singlet, bs = broad singlet, d = doublet, dd = doublet of doublet, t = triplet, q = quartet, quin = quintet, m = multiplet), coupling constant (if applicable), and integration.

ICP-MS:

ICP-MS analysis was performed using a PerkinElmer NexION 2000 ICP-MS.

HPLC:

HPLC-grade solvents were obtained from Fischer Scientific.

Method 1: HPLC analysis was performed on an Agilent 1220 series HPLC with a Lux 5u Cellulose-2 column (250 x 4.6 mm, 5 µm) at a flow-rate of 1.25 mL/min using 5% v/v isopropanol/hexanes.

Method 2: HPLC analysis was performed on an Agilent 1220 series HPLC with a Lux 5u Cellulose-2 column (250 x 4.6 mm, 5 µm) at a flow-rate of 1.25 mL/min using 50% v/v isopropanol/hexanes.

2. Synthetic schemes

Synthesis of Nirmatrelvir

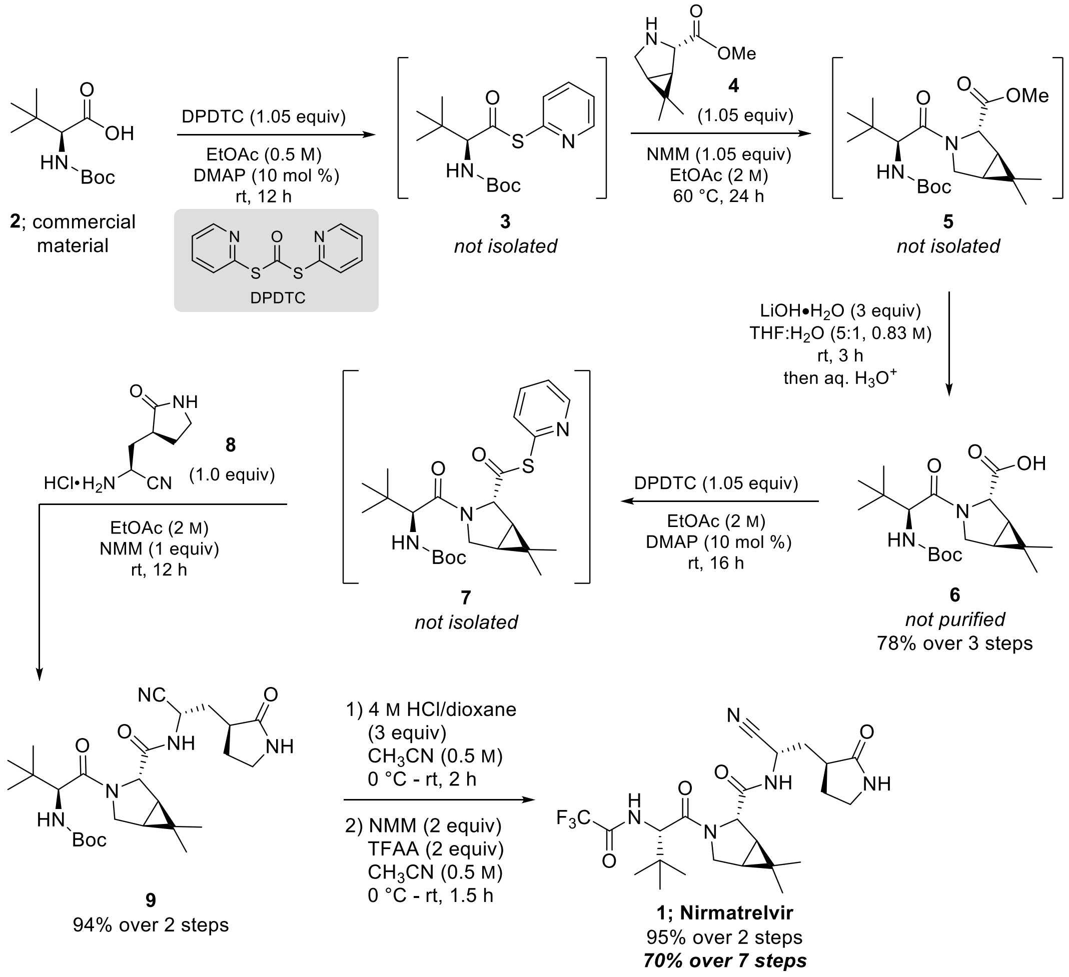

Synthesis of Intermediate 8

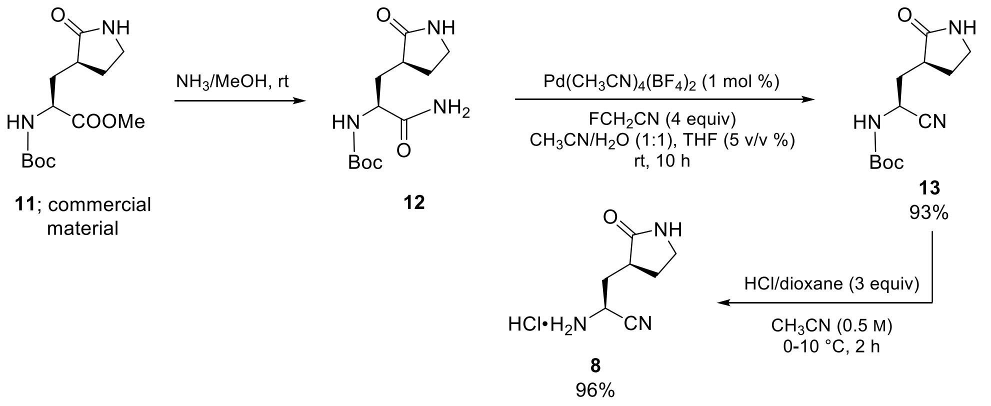

Scheme S1: Synthetic routes for the synthesis of nirmatrelvir (**1**)

3. Synthetic Procedures

3.1 Optimization of the thioesterification to afford thioester **3**

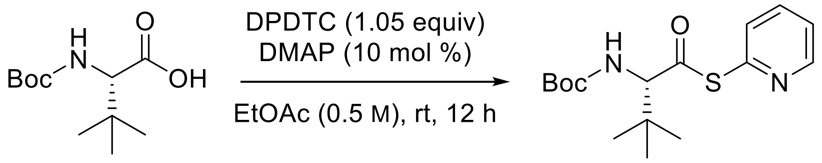

Scheme S2: Thioesterification to afford thioester **3**

Preservation of stereointegrity:

Thioesterification reactions using DPDTC can typically be performed under neat conditions (i.e., no solvent); however, it was found that this leads to considerable racemization of substrates with chiral centers *alpha* to the carboxylic acid moiety. The use of 0.5 M EtOAc and a reaction time of 12 h proved sufficient to limit the extent of racemization and afford product **3** in 99.6% ee as determined by HPLC analysis (Method 2, see section 1 of this SI).

Table S1: Optimization of concentration to minimize epimerization

entry[a]	concentration (M)	yield (%)[b]	ee (%)[c]
1	2	93	96.6
2	1	91	97.0
**3**	**0.5**	**92**	**97.8**

[a] Reactions performed on 0.25 mmol scale for 21 h; [b] ^{1}H NMR yield using 1,3,5-trimethoxybenzene as internal standard; [c] Determined by chiral HPLC (see Figures S1 and S2).

Table S2: Optimization of time to minimize epimerization

entry[a]	time (h)	yield (%)[b]	ee (%)[c]
**1**	**12**	**90**	**99.6**
2	16	89	99.0
3[d]	21	92	97.8

[a] Reactions performed on 0.25 mmol scale at 0.5 M concentration; [b] Isolated yield; [c] Determined by chiral HPLC (see Figures S1 and S2); [d] ^{1}H NMR yield using 1,3,5-trimethoxybenzene as internal standard.

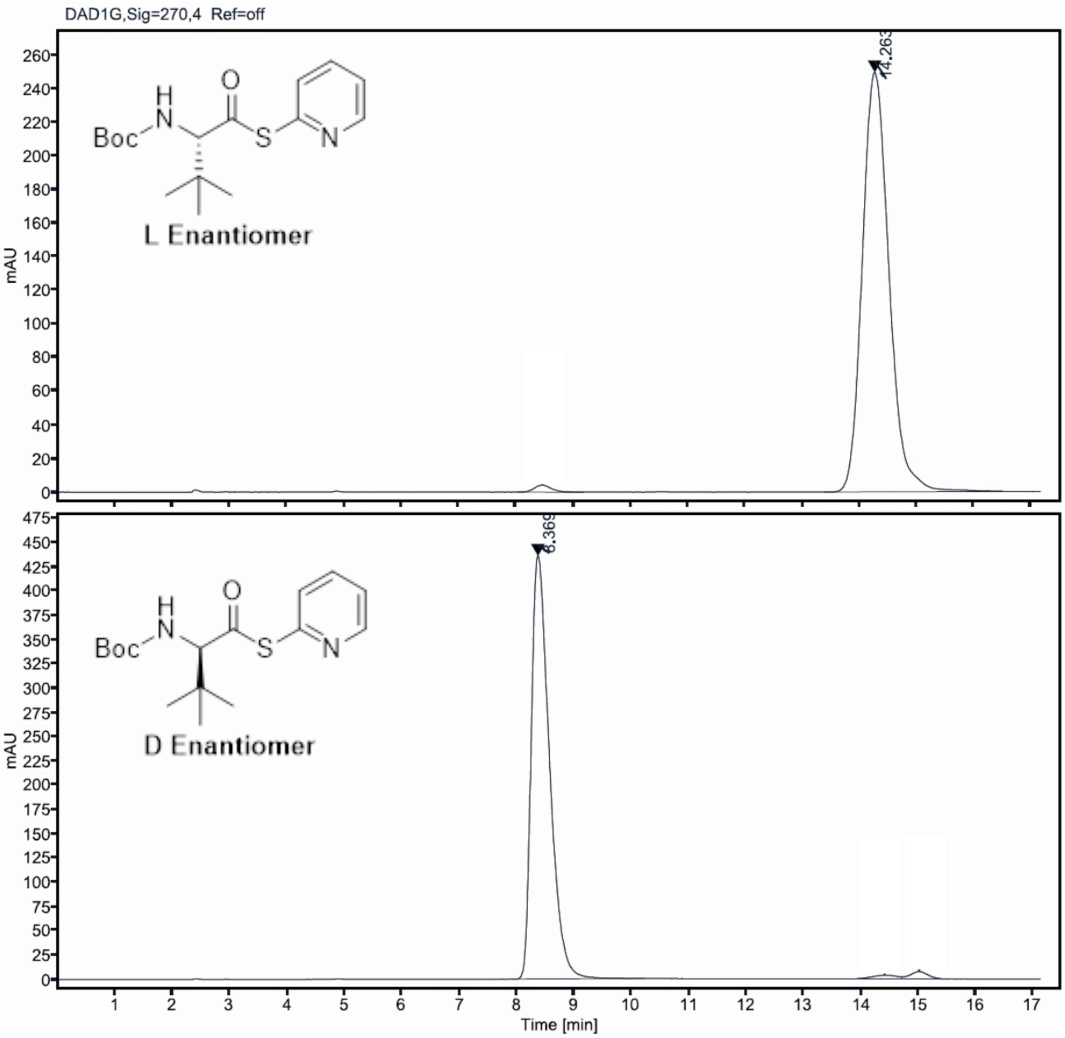

Figure S1: Chiral HPLC chromatograms for both enantiomers of **3**

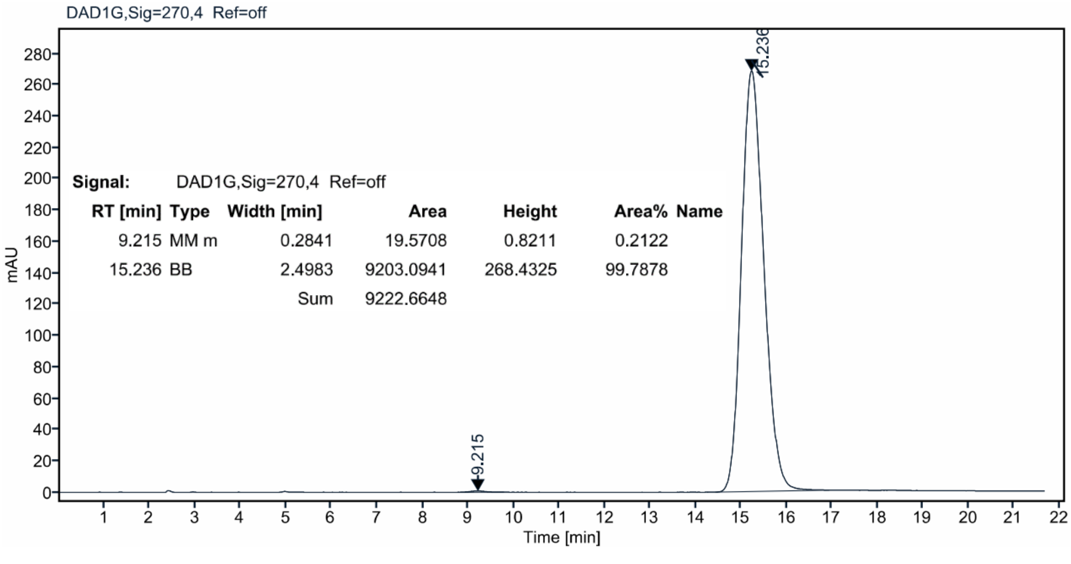

Figure S2: Chiral HPLC chromatogram used to determine ee of **3** (see Table S2, entry 1)

3.2 Optimization of the amide bond coupling *en route* to dipeptide **5**

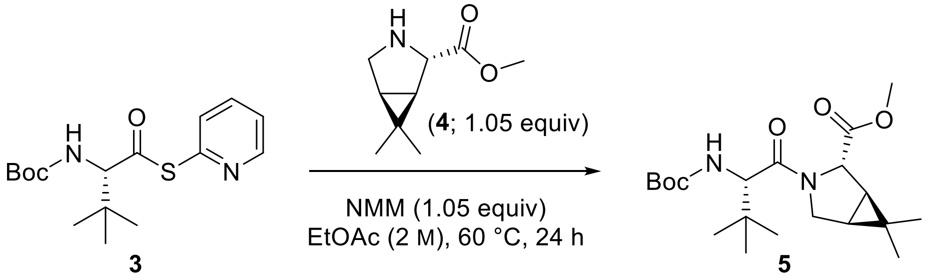

Scheme S3: Amide bond coupling *en route* to dipeptide **5**

Optimization began with the use of H-Pro-OMe hydrochloride *as a model amine* (Table S3). While neat conditions (i.e., no solvent) are typical in our group for amide bond formations using thioesters, it was found that this led to varying levels of racemization of the Boc-Tle-OH stereocenter. Solvents (including aqueous surfactant solutions using nonionic surfactants MC-1[1] and TPGS-750-M[2]), bases, and temperatures were screened, and the best results (i.e., highest yield with no racemization) were observed when using a 2 wt % aqueous solution of MC-1 with 20 v/v % EtOAc as co-solvent at 60 °C and using 2 equiv $NaHCO_3$ as the base (entry 12).

Table S3: Optimization of amide bond formation using proline methyl ester *as a model amine*

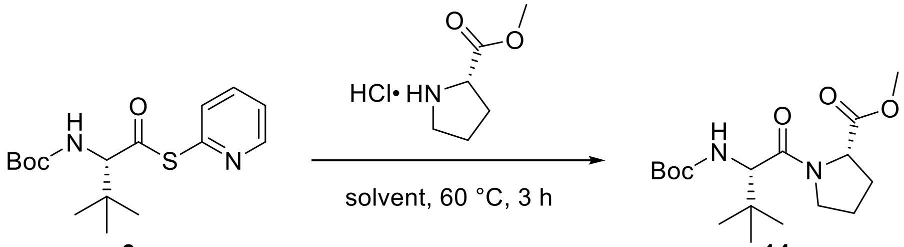

entry[a]	solvent	cosolvent (20 v/v/ %)	temp (°C)	thioester (equiv)	base	base (equiv)	yield (%)[b]	d.r.[c]
1	neat	-	rt	1.2	Et_3N	2	90	72:28
2	DMSO (2.5 M)	-	rt	1.2	Et_3N	1	92	69:31
3	sulfolane (2.5 M)	-	rt	1.2	Et_3N	1	89	68:32
4	DMSO (2.5 M)	-	60	1.2	Et_3N	1	98	67:33
5	sulfolane (2.5 M)	-	60	1.2	Et_3N	1	93	67:33
6	2 wt % TPGS-750-M/H_2O	-	60	1.2	Et_3N	1	38	88:12
7	2 wt % MC-1/H_2O	-	60	1.2	Et_3N	1	34	86:14
8	2 wt % MC-1/H_2O	-	rt	1.5	$NaHCO_3$	1	20	>99:1
9	2 wt % MC-1/H_2O	-	rt	1.5	$NaHCO_3$	2	17	>99:1
10	2 wt % MC-1/H_2O	-	40	1.5	$NaHCO_3$	2	13	>99:1
11	2 wt % MC-1/H_2O	-	60	1.5	$NaHCO_3$	2	56	96:4
**12**	**2 wt % MC-1/H_2O**	**EtOAc**	**60**	**1.5**	**$NaHCO_3$**	**2**	**81**	**>99:1**
13	2 wt % MC-1/H_2O	DMSO	60	1.5	$NaHCO_3$	2	70	93:7

[a] Reactions were performed on 0.25 mmol scale (w/r/t. HCl•H-Pro-OMe) overnight; [b] ^1H NMR yield using 1,3,5-trimethoxybenzene as internal standard; [c] Determined by ^1H NMR.

When the same conditions were applied to the authentic substrate using *bicyclic* proline **4**, a much lower yield (17%) was observed (Table S4, entry 1). The use of neat conditions led to nearly quantitative conversion, but significant racemization was noted (entry 2). The use of biphasic mixtures of H_2O/EtOAc or *i*PrOAc did not lead to racemization, but the yields were not satisfactory (entries 3-5). Ultimately, use of minimal EtOAc (2 M global concentration) allowed for full conversion of the thioester to the dipeptide **5** using the ideal 1:1 ratio of coupling partners, providing product in 90% isolated yield with no racemization detected by ^1H NMR (entry 7).

Table S4: Optimization of amide bond formation to make dipeptide **5**

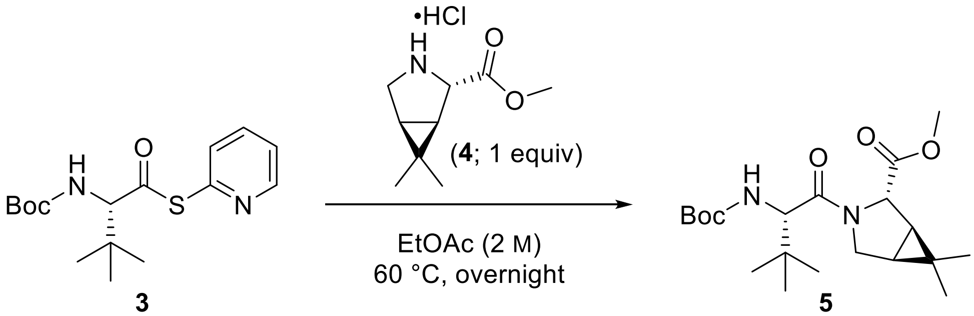

3 **5**

entry[a]	solvent	cosolvent (20 v/v %)	temp (°C)	thioester (equiv)	base	base (equiv)	yield (%) [b]	d.r.[c]
1	2 wt % MC-1/H2O	EtOAc	60	1	$NaHCO_3$	4	17	>99:1
2	neat	-	60	1	$NaHCO_3$	3	97	70:30
3	H_2O/EtOAc (4:1)	-	60	1.5	$NaHCO_3$	2	71	>99:1
4	H_2O/EtOAc (2:1)	-	60	1.5	$NaHCO_3$	2	81	>99:1
5	H_2O/*i*PrOAc (2:1)	-	60	1.5	$NaHCO_3$	2	70	>99:1
6[d,e,f]	EtOAc (2 M)	-	60	1	NMM	1	87	>99:1
**7[d,e,f,g]**	**EtOAc (2 M)**	**-**	**60**	**1**	**NMM**	**1.05**	**90**	**>99:1**

[a] Reactions performed on a 0.25 mmol scale (w/r/t. amine hydrochloride) unless otherwise noted; [b] ^1H NMR yield using 1,3,5-trimethoxybenzene as internal standard; [c] Determined by ^1H NMR; [d] Using freebase amine and 1 equiv NMM; [e] Isolated yield; [f] 0.5 mmol scale; [g] 1.05 equiv freebase amine.

3.3 *N*-Boc-Deprotection / trifluoroacetylation to afford nirmatrelvir **1**

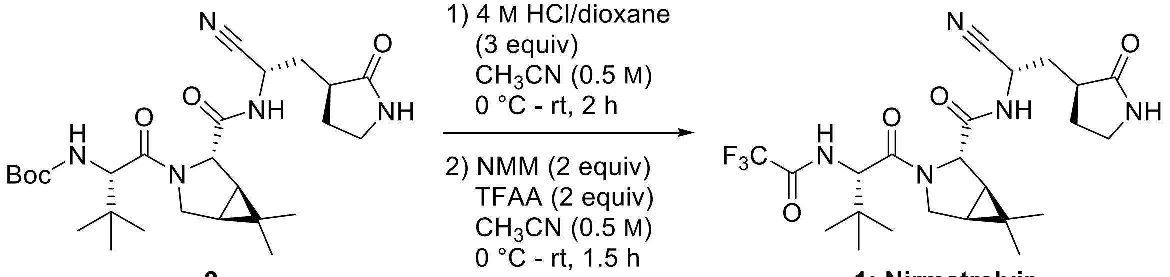

Scheme S4: *N*-boc-deprotection/trifluoroacetylation to afford nirmatrelvir **1**

The unwanted diastereomer (and other impurities) could be removed by one of two methods:

1) column chromatography (1-3% MeOH/CH_2Cl_2), affording nirmatrelvir as a white solid (**1**; 201.3 mg, 81% yield over 2 steps, >99% purity; see Figure S3). Loss in yield is the result of discarding several fractions containing both the desired drug and its unwanted diastereomer.
2) Formation and subsequent recrystallization of the MTBE solvate, as per Pfizer's protocol.[3] A yield was not obtained for this method owing to limitations of scale. However, successful removal of impurities, including the unwanted diastereomer, was observed by HPLC (Figure S4). A mixture containing even 23% of the undesired diastereomer could be reduced to 6% simply by forming the solvate. Recrystallization of the solvate would likely reduce this amount further. Formation of the same MTBE salt for a ca. 30 mg mixture containing only 3% diastereomer reduced the diastereomer to 1%.

ICP-MS analysis of nirmatrelvir (**1**) was performed by the UCLA Nano and Pico (NPC) Laboratory in order to determine the amount of residual palladium present in the final drug, and was shown to be below the detectable limit (see Table S5). This was performed on material that had not been purified via the MTBE solvate method described above.

Table S5: ICP-MS analysis of nirmatrelvir **1** to determine the amount of residual palladium

		**Palladium**	
		[µg/g]	
Sample #	Sample weight in analysis [mg]	Average*	stdev
Nirmatrelvir Pure	7.30	0.000	0.000

**Each sample was done in triplicated measurements with background correction.*

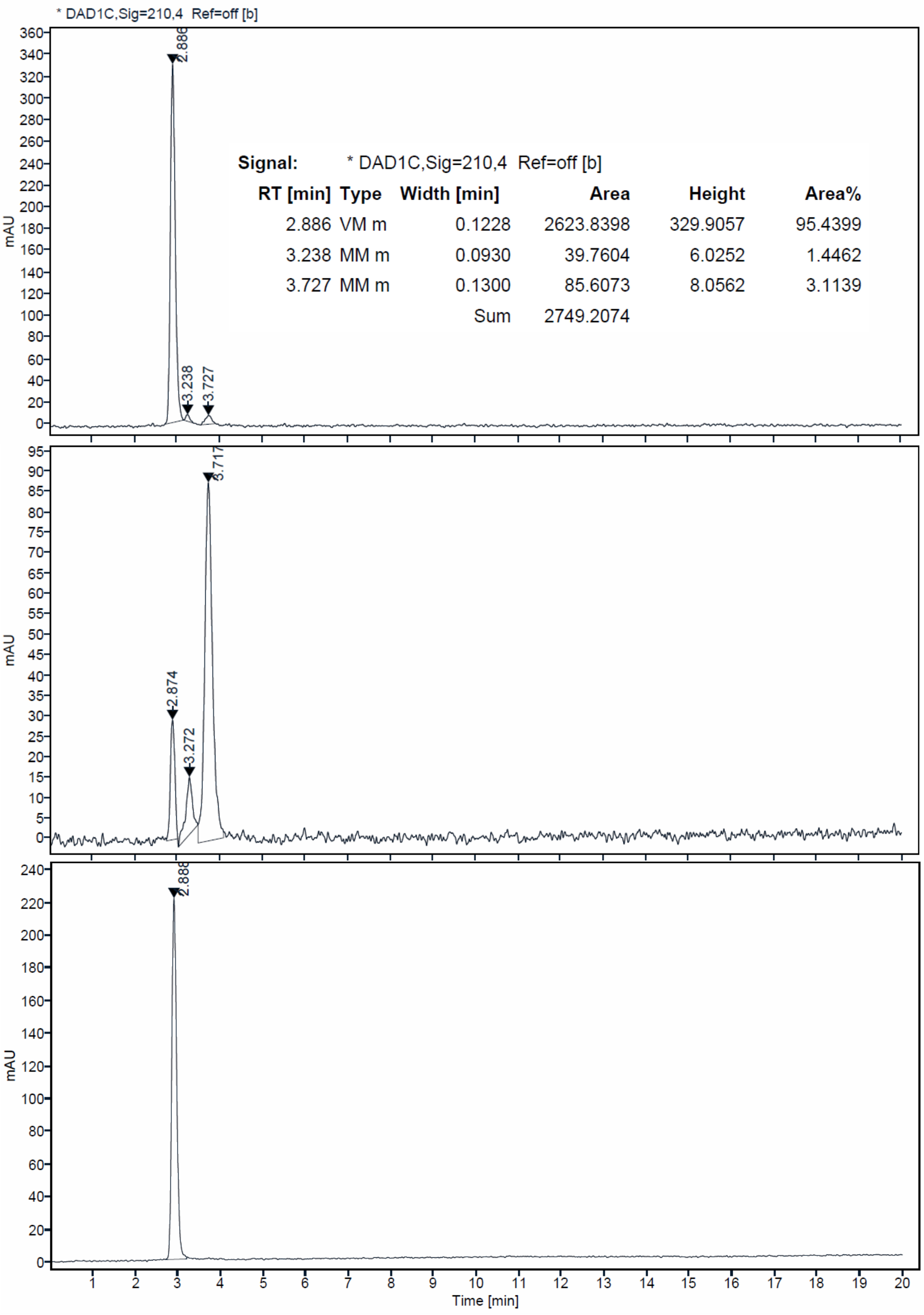

Signal: * DAD1C,Sig=210,4 Ref=off [b]

RT [min]	Type	Width [min]	Area	Height	Area%
2.886	VM m	0.1228	2623.8398	329.9057	95.4399
3.238	MM m	0.0930	39.7604	6.0252	1.4462
3.727	MM m	0.1300	85.6073	8.0562	3.1139
		Sum	2749.2074		

Figure S3: HPLC analysis of nirmatrelvir **1** via chiral HPLC (see SI section 1, HPLC method 2). Top: crude reaction mixture containing both diastereomers and an unidentified impurity; Middle: unwanted diastereomer (large peak; isolated by column chromatography); Bottom: pure nirmatrelvir **1** following purification by column chromatography.

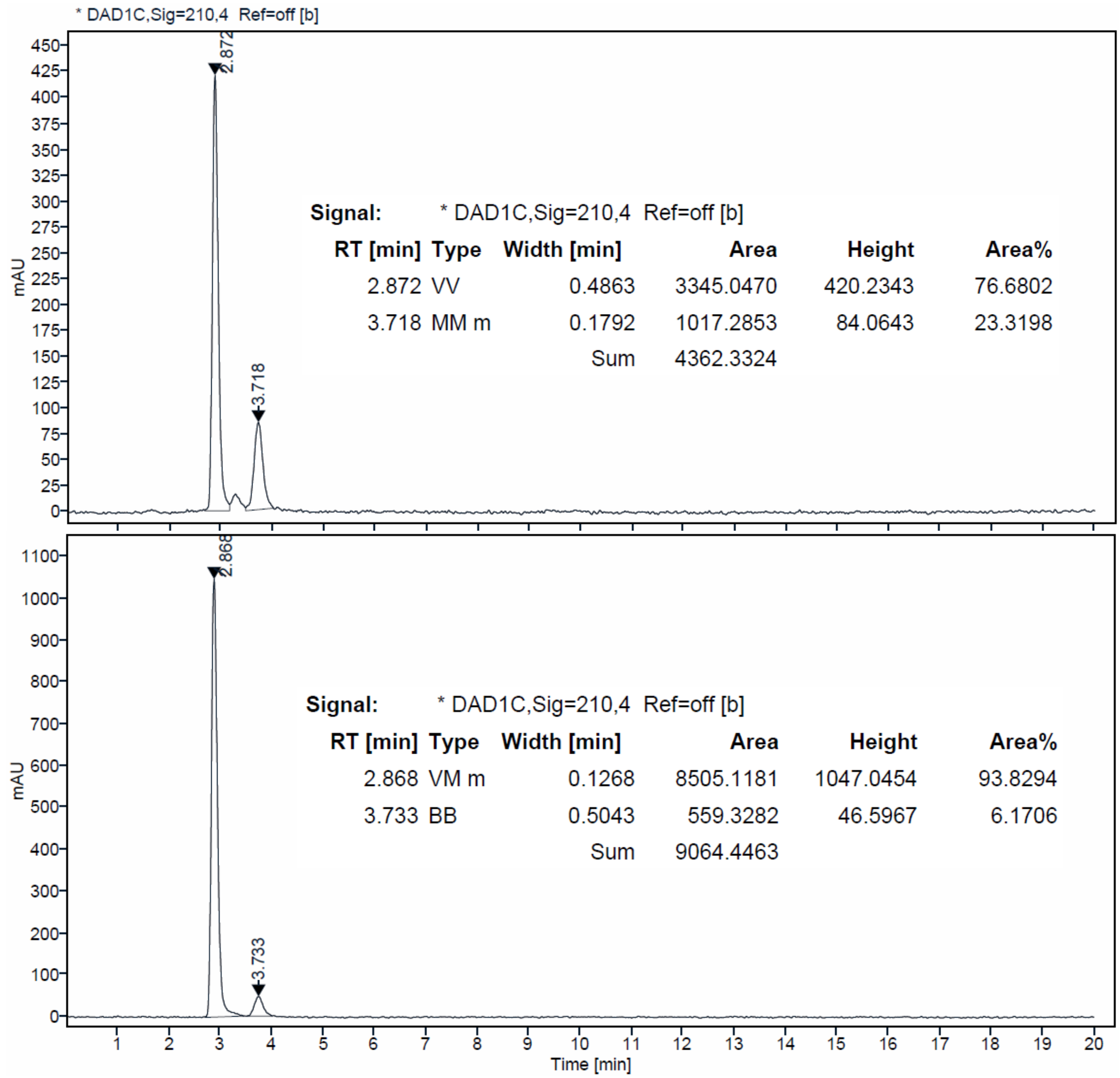

Figure S4: Chiral HPLC analysis of a sample containing a mixture of nirmatrelvir diastereomers (see SI section 1, HPLC method 2). Top: before formation of the MTBE solvate according to Pfizer's protocol;[3] Bottom: after formation of MTBE solvate, depicting significant improvement in dr.

Formation of conformers during *N*-Boc deprotection:

It was observed by ^{1}H NMR (see Figures S5 and S7) that the product of the *N*-Boc deprotection existed as a mixture of two conformers (possibly rotamers about the tertiary amide bond formed by the bicyclic proline moiety). When the ^{1}H NMR was taken in CD_3OD immediately following isolation of the crude product, the conformers existed in a 70:30 ratio via peak integration. Interestingly, the conformers could be observed on TLC following trifluoroacetylation (3% $MeOH/CH_2Cl_2$, CAM stain; major rotamer $R_f = 0.14$; minor rotamer $R_f = 0.21$). However, these

trifluoroacetylated rotamers were not separable by HPLC. In its isolated solid form (i.e., free of solvent and excess HCl), the amine hydrochloride salt remained as a 70:30 mixture indefinitely.

When the reaction mixture (i.e., precipitated product with an overlayer of CH_3CN containing HCl/dioxane) was allowed to stand at 4 °C for 1 h, the ratio of rotamers increased to 90:10. When the reaction was instead allowed to stand at rt for 16 h, the ratio increased to 94:6. Increasing the equilibration time to 45 h did not significantly improve the ratio. It was crucial to allow the mixture to equilibrate as the minor conformer leads to epimerization during the trifluoroacetylation step (see Figure S6); use of the 70:30 mixture in this reaction led to 12% epimerization, whereas use of the 94:6 mixture led to only 3% epimerization. This result was reproduced more than five times. In order to maximize time economy associated with the process, a 16 h equilibration time was taken to be optimal, thus 3% of the diastereomer was present in the final product, and could be removed by either of the purification techniques described above.

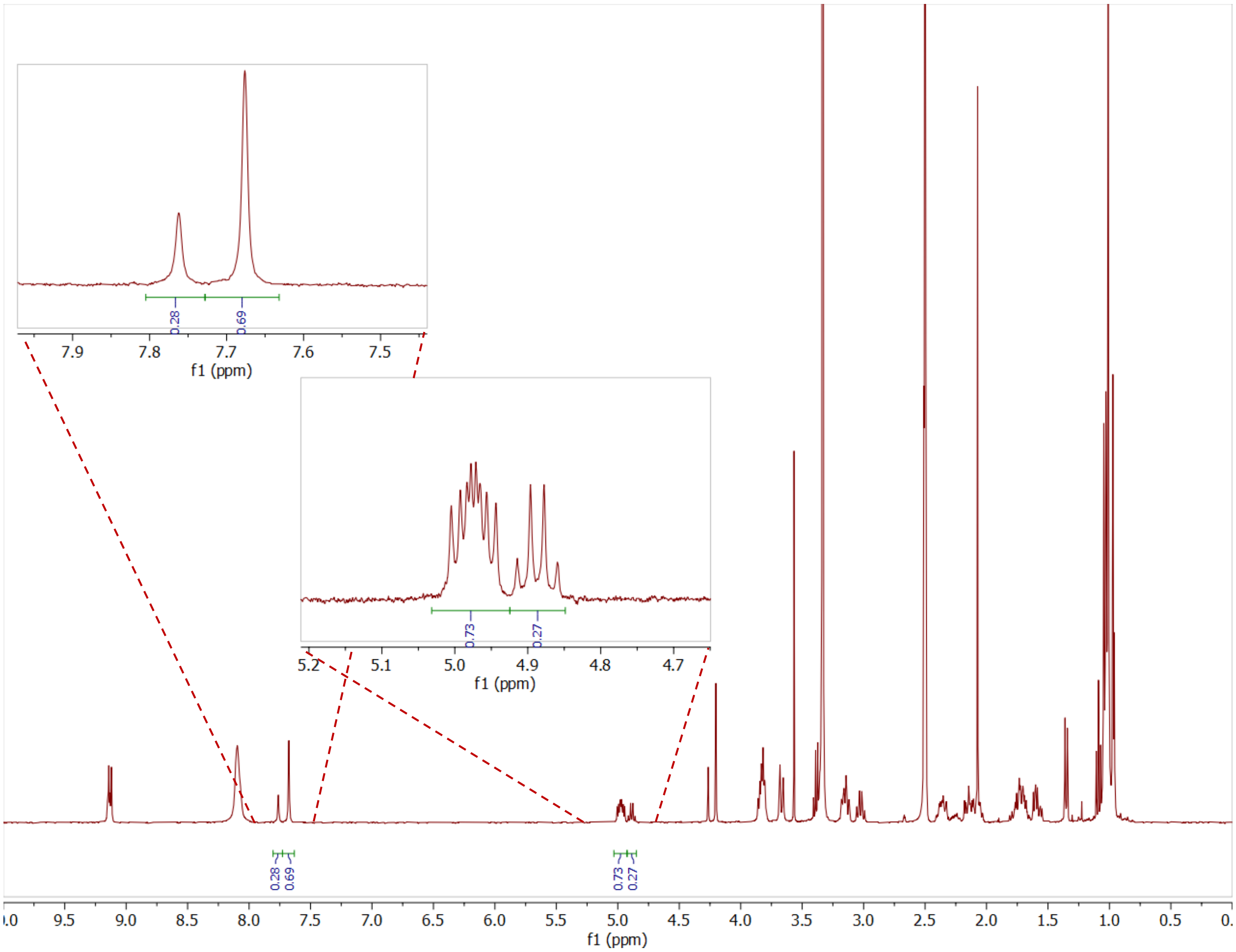

Figure S5: ^{1}H NMR spectra of the *N*-Boc-deprotection product immediately following the reaction (i.e., no equilibration time) depicting the ca. 70:30 ratio of rotamers.

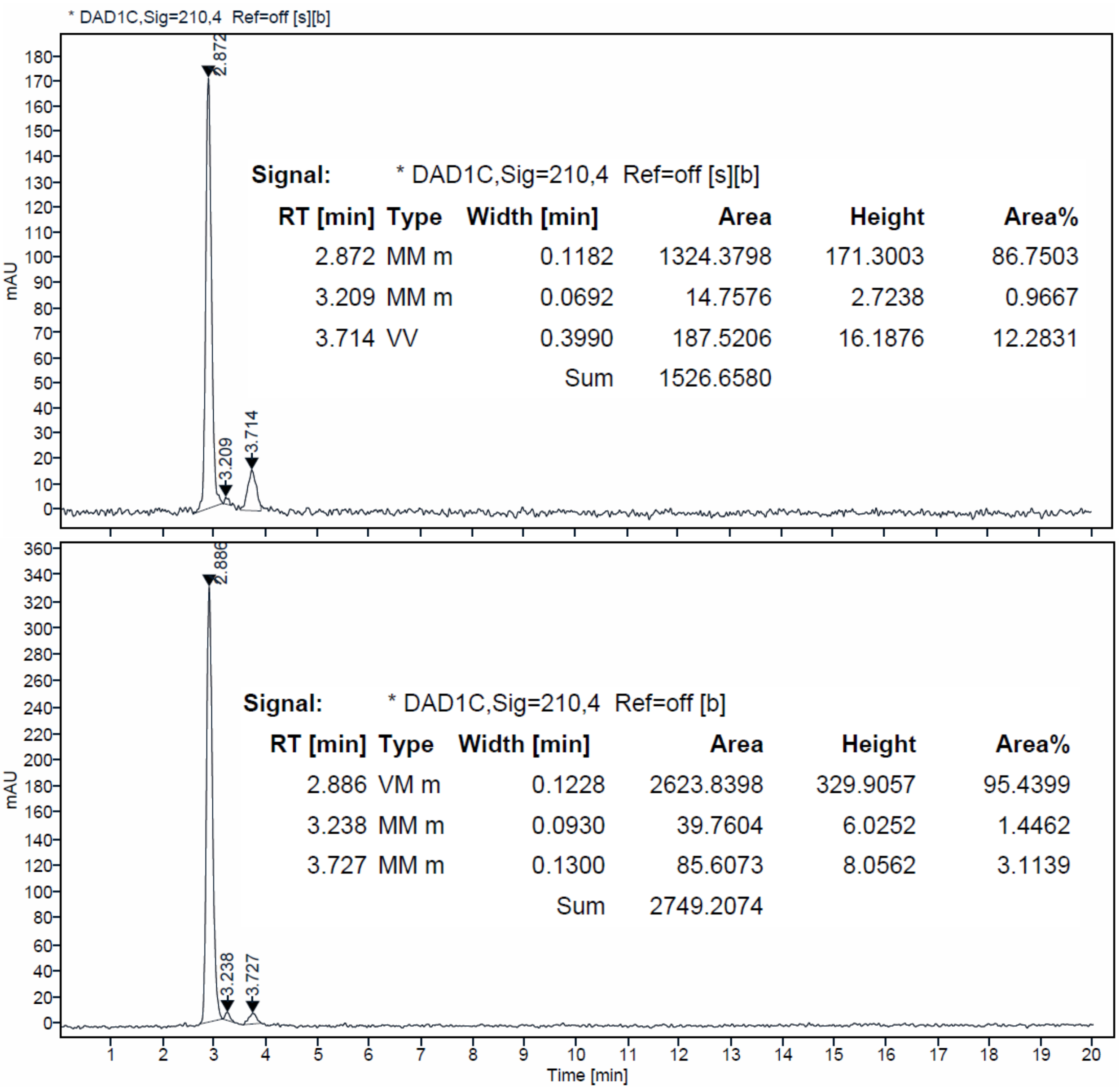

Figure S6: Chiral HPLC analysis (see ESI section 1, HPLC method 2) of the trifluoroacetylation products depicting degrees of epimerization when the *N*-Boc deprotection product was equilibrated for: Top: 0 h (no equilibration); Bottom: 16 h equilibration at rt.

Identification of the rotamers:

Pfizer observed mixtures of rotamers for compounds bearing the same bicyclic proline moiety as in nirmatrelvir.[3] They confirmed this using variable temperature NMR and ^{1}H-^{1}H NOESY. Subjecting the amine hydrochloride salt (70:30 mixture of rotamers, made using our *N*-Boc deprotection protocol) to varying temperatures in DMSO-d_6 showed a not-quite complete coalescence of peaks with increasing temperature (Figure S7); i.e., full coalescence was not observed before reaching the maximum temperature of our spectrometer (120 °C). This, in

combination with Pfizer's observations of related compounds, was sufficient to conclude that the previously unknown peaks visible on ^{1}H NMR corresponded to rotamers (as suggested by Pfizer). Plausible structures for the two rotamers were determined using DFT calculations (see SI-2).

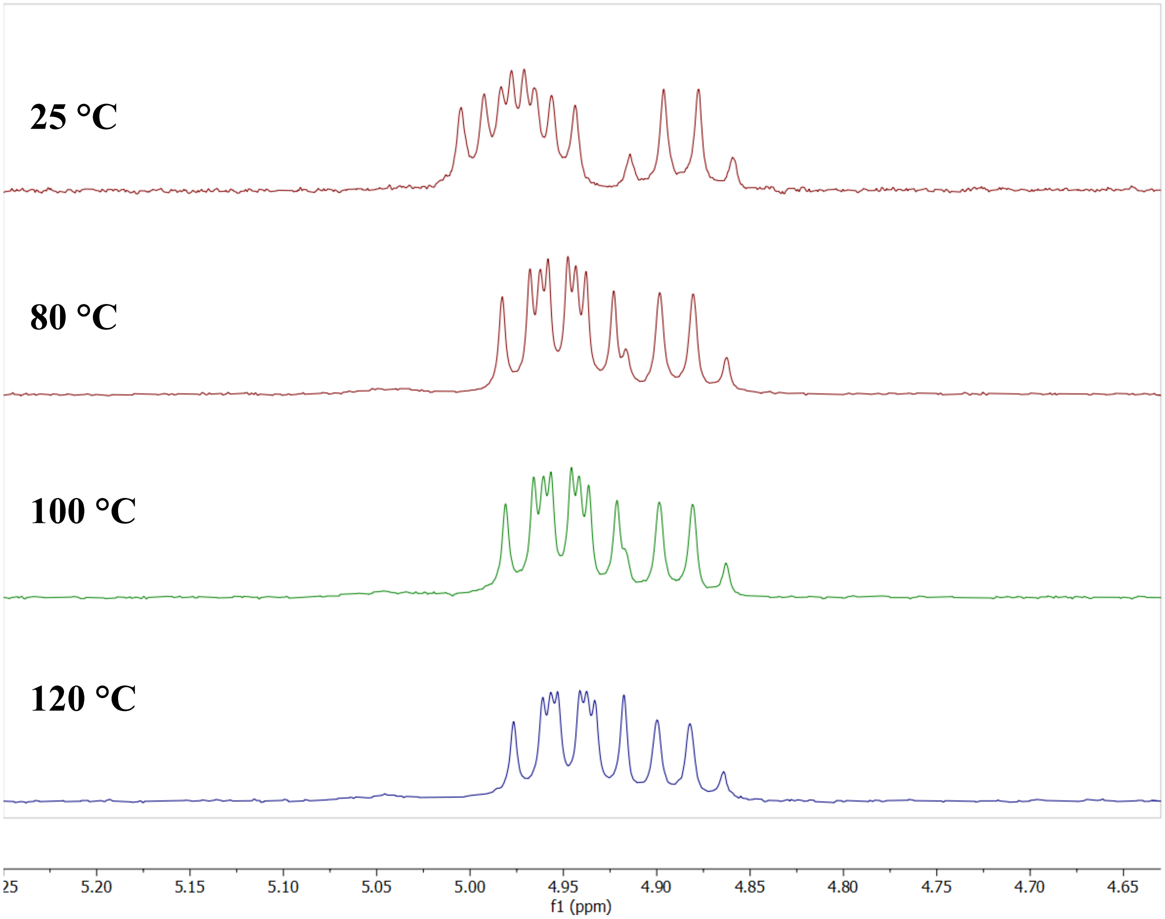

Figure S7: Variable temperature NMR spectra of a 70:30 mixture of amine hydrochloride salt rotamers depicting coalescence of peaks with increasing temperature.

Interestingly, while a mixture of rotamers was seen in the ^{1}H NMR spectrum of the final trifluoroacetylated API, the ^{1}H NMR spectrum of the subsequently formed MTBE solvate showed only a single rotamer (Figure S8).

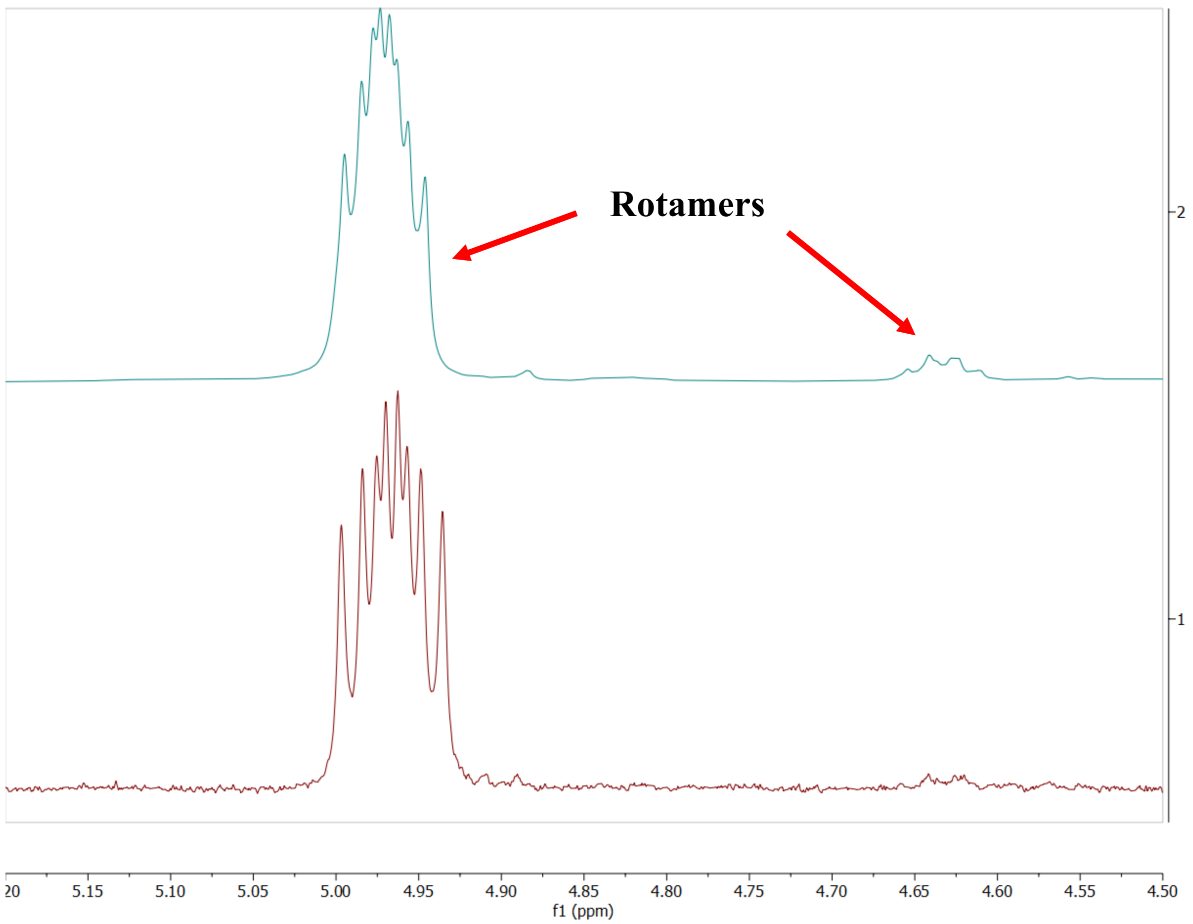

Figure S8: ^{1}H NMR spectra of: Top: nirmatrelvir before formation of the MTBE solvate, depicting presence of rotameric mixture; Bottom: MTBE solvate of nirmatrelvir, showing less of the minor rotamer.

Identification of the unwanted diastereomer:

The unwanted diastereomer was visible on TLC (3% MeOH/CH_2Cl_2, CAM stain; major diastereomer $R_f = 0.14$, minor (unwanted) diastereomer $R_f = 0.17$). The diastereomer was isolated by column chromatography (1-3% MeOH/CH_2Cl_2) and subjected to HRMS (*m*/*z* calcd for $C_{23}H_{32}F_3N_5O_4{+}H^+$: 500.2479 [*M*+H]+: found 500.2474) and chiral HPLC analysis (see Figure S3), which confirmed that the material was a diastereomer of nirmatrelvir **1**. It was not possible to isolate 100% pure diastereomer as it partially co-elutes with the minor conformer (see minor peak in HPLC spectrum of the diastereomer in Figure S3). It should be noted that we do not currently know which stereocenter epimerizes and hence, the exact identity of this material.

Control experiments to identify the source of the epimerization were performed by exposing pure nirmatrelvir to various additives in 0.5 M CH_3CN and stirring for 2 h. No evidence of epimerization was observed when either 5 equiv of NMM or 3 equiv of HCl were used. However, 8% epimerization was observed when 3 equiv of TFA was used (Figure S9), suggesting that the TFA produced as a byproduct of trifluoroacetylation plays a role in the epimerization of the minor conformer.

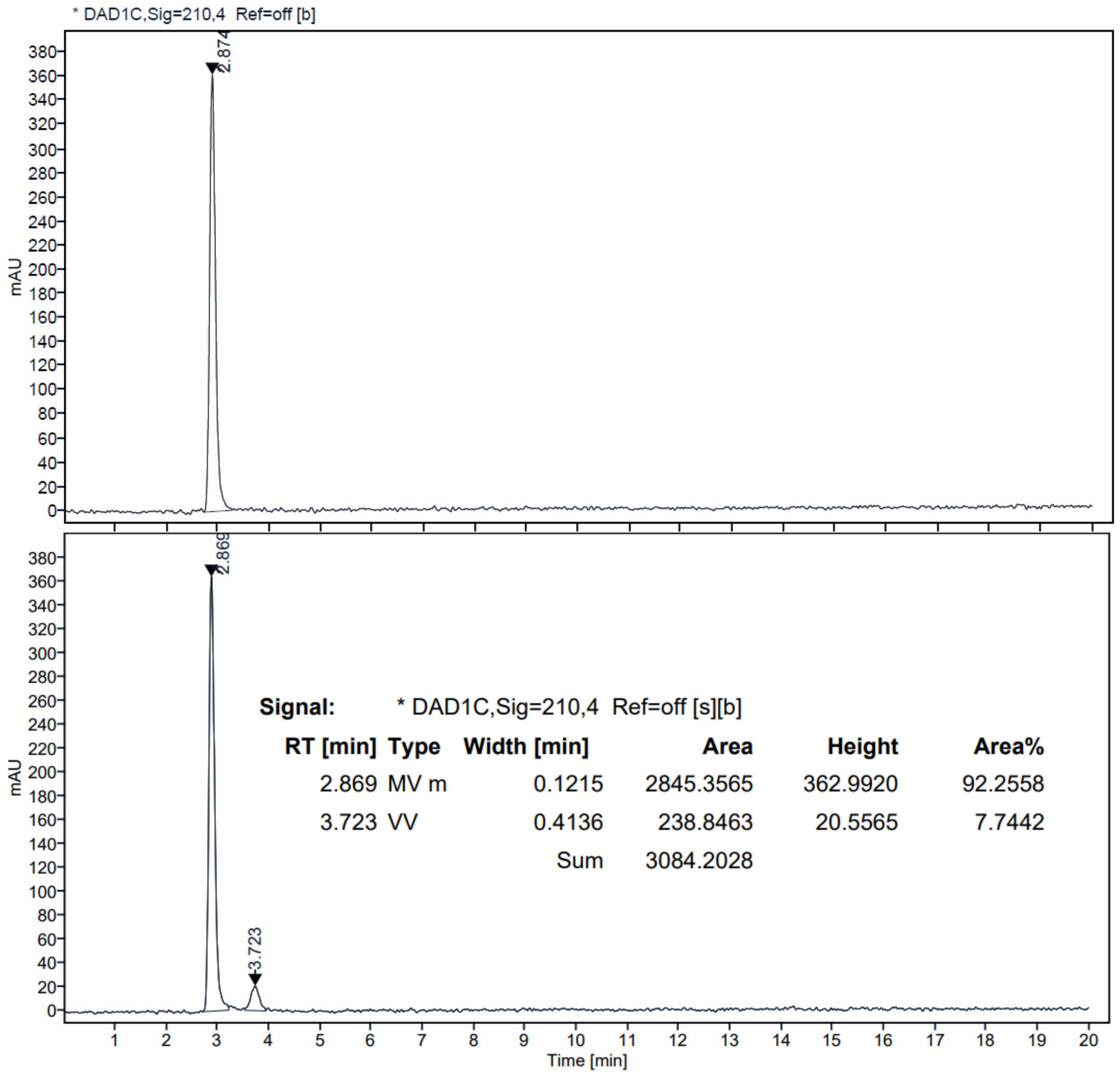

Figure S9: Top: pure nirmatrelvir before exposure to TFA; Bottom: after exposure to 3 equiv TFA for 2 h at rt in 0.5 M CH_3CN, depicting epimerization under these conditions

3.4 Optimization of the amide dehydration to afford nitrile **13**

Primary amide starting material **12** was prepared as previously reported.[3]

Optimization started with the protocol described by Wood, et al.[4] under conditions of micellar catalysis using a 2 wt % solution of the nonionic surfactant TPGS-750-M in water (Table S6, entries 1-3, 15). However, further optimization for this particular substrate led to conditions more related to those described by Okabe and coworkers.[5] Thus, use of $Pd(CH_3CN)_4(BF_4)_2$ as catalyst, fluoroacetonitrile as the sacrificial nitrile, and a 1:1 mixture of DI water and acetonitrile containing 10 v/v % THF as reaction medium at rt gave the best results (91% yield by ^1H NMR, entry 24).

Table S6: Optimization of the amide dehydration to afford nitrile **13**

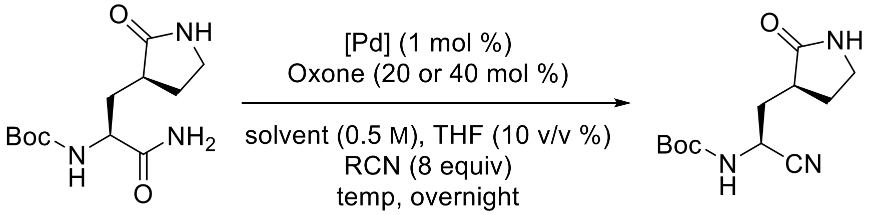

entry[a]	[Pd]	RCN	H_2O : MeCN	temp (°C)	surfactant	yield (%)[b]
1	$Pd(OAc)_2$	Methoxyacetonitrile	1 : 0	60	TPGS	NR
2	$Pd(OAc)_2$	Dichloroacetonitrile	1 : 0	45	TPGS	NR
3	$Pd(OAc)_2$	Dichloroacetonitrile	1 : 0	60	TPGS	NR
4	$Pd(OAc)_2$	Methoxyacetonitrile	1 : 1	45	-	33
5	$Pd(OAc)_2$	Methoxyacetonitrile	1 : 9	45	-	35
6	$Pd(OAc)_2$	Dichloroacetonitrile	1 : 1	45	-	13
7	$Pd(OAc)_2$	Dichloroacetonitrile	1 : 9	45	-	33
8	$Pd(OAc)_2$	Trichloroacetonitrile	1 : 1	45	-	NR
9	$Pd(OCOCF_3)_2$	Methoxyacetonitrile	1 : 1	45	-	49
10	$Pd(CH_3CN)_4(BF_4)_2$	Methoxyacetonitrile	1 : 1	45	-	67
11[c]	$Pd(OAc)_2$	Methoxyacetonitrile	1 : 1	45	-	26
12	$Pd(CH_3CN)_4(BF_4)_2$	Methoxyacetonitrile	1 : 1	60	-	47
13[d]	$Pd(CH_3CN)_4(BF_4)_2$	Methoxyacetonitrile	1 : 1	45	-	77
14[d]	$Pd(CH_3CN)_4(BF_4)_2$	Methoxyacetonitrile	1 : 1	60	-	65
15	$Pd(CH_3CN)_4(BF_4)_2$	Methoxyacetonitrile	1 : 0	60	TPGS	19
16	$Pd(CH_3CN)_4(BF_4)_2$	Trifluoropropionitrile	1 : 1	45	-	25
17[d]	$Pd(CH_3CN)_4(BF_4)_2$	Trifluoropropionitrile	1 : 1	45	-	53
18[e]	$Pd(CH_3CN)_4(BF_4)_2$	Methoxyacetonitrile	1 : 1	45	-	45
19[e]	$Pd(CH_3CN)_4(BF_4)_2$	Methoxyacetonitrile	0 : 1	45	-	60
20[f]	$Pd(CH_3CN)_4(BF_4)_2$	Methoxyacetonitrile	1 : 1	45	-	33
21[g]	$Pd(CH_3CN)_4(BF_4)_2$	Methoxyacetonitrile	1 : 1	45	-	46
22[h]	$Pd(CH_3CN)_4(BF_4)_2$	Methoxyacetonitrile	1 : 1	45	-	43
23	$Pd(CH_3CN)_4(BF_4)_2$	Methoxyacetonitrile	1 : 1	rt	-	84
**24**	**$Pd(CH_3CN)_4(BF_4)_2$**	**Fluoroacetonitrile**	**1 : 1**	**rt**	**-**	**91**
25[i]	$Pd(CH_3CN)_4(BF_4)_2$	Fluoroacetonitrile	1 : 1	rt	-	88
26	$Pd(CH_3CN)_4(BF_4)_2$	Fluoroacetonitrile	1 : 9	rt	-	53

[a] Reactions performed on 0.25 mmol scale; [b] ^1H NMR yield using 1,3,5-trimethoxybenzene as internal standard; [c] 40 mol % Oxone; [d] 2 mol % [Pd]; [e] 95% EtOH instead of MeCN; [f] Run under an Ar atmosphere; [g] Bubbled with O_2; [h] Run without THF; [i] 10 equiv RCN.

Further optimization focused on reducing the amount of catalyst and reagents, as well as the reaction medium required to effect this transformation. The loading of the sacrificial nitrile could be reduced to 4 equiv, and the amount of THF could be lowered to 5 v/v %. Moreover, the Oxone could be omitted entirely. These conditions led to a 93% isolated yield of **13** over an 18 h period (Table S7, entry 11).

Table S7: Further optimization for dehydration of amide **12**

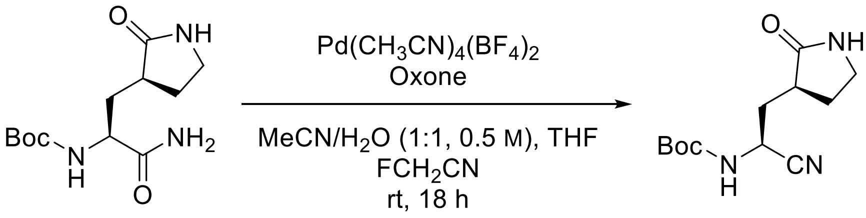

entry	[Pd] (mol %)	FCH_2CN (equiv)	H_2O : MeCN	THF (v/v %)	Oxone (mol %)	yield (%)[a]
1	1	8	1 : 1	10	20	90
2	0.5	8	1 : 1	10	20	62
3	0.8	8	1 : 1	10	20	71
4	1	6	1 : 1	10	20	89
5	1	4	1 : 1	10	20	93
6	1	2	1 : 1	10	20	60
7	1	4	1 : 1	10	10	93
8	1	4	1 : 1	5	20	95
9	1	4	3 : 1	5	10	65
10	1	4	1 : 1	5	5	94
**11**	**1**	**4**	**1 : 1**	**5**	**-**	**93**
12	1	4	1 : 1	-	5	87
13	1	4	1 : 1	-	-	86

[a] Isolated yield.

Finally, the optimal reaction time was determined by isolating reactions at several timepoints (Figure S10). This revealed an optimal reaction time of 10 h.

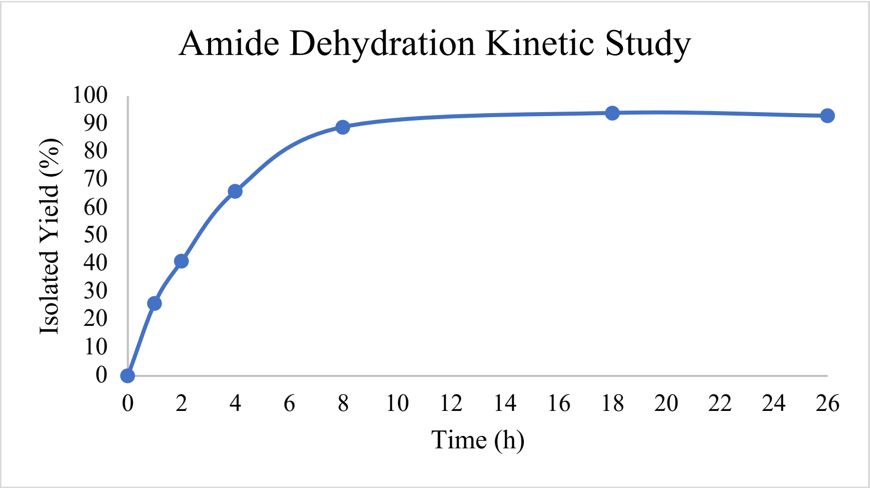

Figure S10: Conversion of primary amide **12** to nitrile **13** over time

Workup Methods:

All amide dehydration products were isolated by directly drying the reaction mixture onto silica and purifying via flash chromatography on silica gel (5% MeOH/CH_2Cl_2). Given that column chromatography is not a viable method of product isolation on scale, the following alternative workup was developed:

> After the reaction is complete, a biphasic mixture can be obtained by the addition of NaCl or saturated brine solution. The lower aqueous layer can then be removed and the remaining organic layer (containing the product, unreacted starting material, fluoroacetamide, and volatile reaction components) can be concentrated *in vacuo*. The crude residue can then be purified by recrystallization from *i*PrOAc (hot filtration of the mother liquor prior to crystallization is recommended to remove precipitated palladium catalyst).

This improved workup leads to very pure material by NMR and should be suitable for scale-up procedures, but it is not suitable for small-scale synthesis owing to inherent losses when recrystallizations are performed on milligram-scale reactions. Instead, column chromatography is preferred for small-scale reactions.

3.5 *N*-Boc-Deprotection *en route* to amine hydrochloride salt **8**

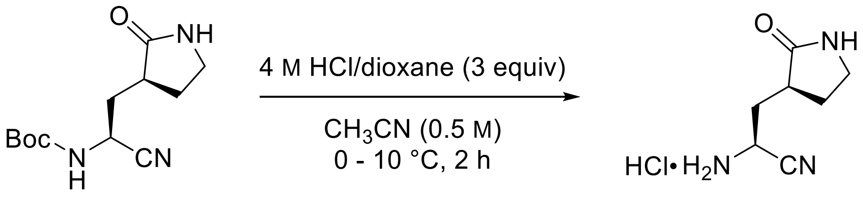

13 **8**

Scheme S5: *N*-boc-deprotection *en route* to amine hydrochloride salt **8**

N-Boc deprotection could be effected using HCl in organic solvents; however, the *N*-Boc deprotected nitrile **13** was susceptible to hydrolysis due to the presence of adventitious moisture, leading to a mixture of products (nitrile **8**, carboxylic acid **8a**, and primary amide **8b**; see Figures S11 and S12 on next pages). Multiple approaches were attempted to eliminate this issue and are summarized in Table S8. The use of neat TFA led to a messy reaction with multiple decomposition or epimerized products (entries 1 and 2). Various commercial solutions of HCl in organic solvents were investigated (entries 3-5), all of which led to hydrolysis due to varying amounts of moisture, and some led to incomplete removal of the Boc group. Attempts to generate HCl *in situ* by dissolving **13** in scrupulously dried MeOH (by refluxing over Mg turnings with I_2 and distilling over 3A molecular sieves, then allowing to stand for 3 d), then adding 3 equiv of freshly distilled AcCl, still led to large amounts of hydrolysis (entry 6). It was found that azeotropically drying the starting material with dry toluene, then using very dry (commercially available; Aldrich) 4 M dioxane/HCl in CH_3CN led to product **8** along with only 3% hydrolysis by ^1H NMR (entry 7). Hydrolysis byproducts could be removed using a procedure similar to that disclosed by Karmakar, et al.[6] Briefly, crude hydrochloride salt was dissolved in a minimal amount of anhydrous MeOH with heating, then chilled in an ice bath to 0-5 °C. Ice-cold Et_2O was then added in excess to precipitate the desired compound **8** free of hydrolysis byproducts (entry 8).

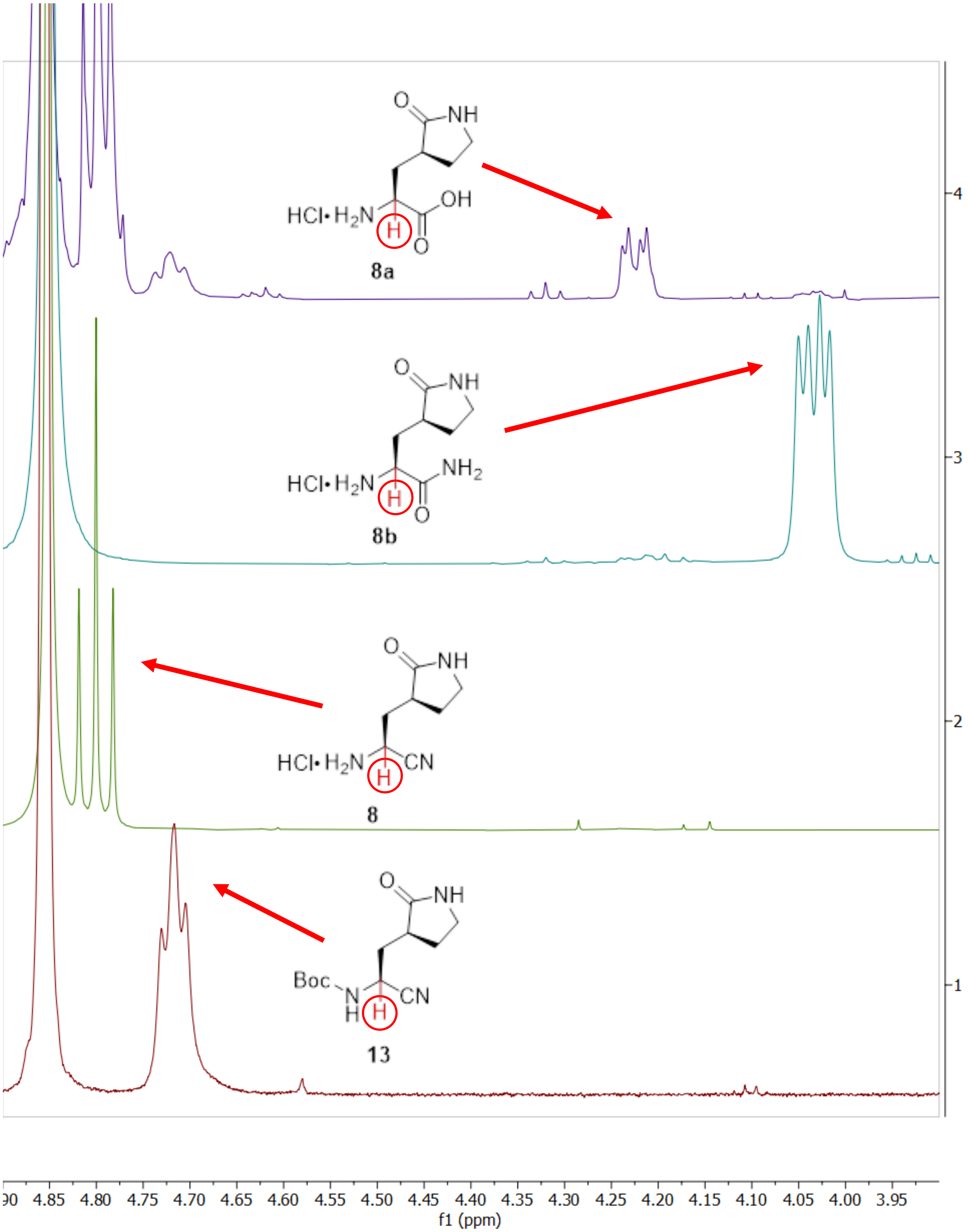

Figure S11: ^{1}H NMR spectra showing characteristic peaks for starting material, product, and hydrolysis byproducts of the *N*-Boc deprotection of **13**.

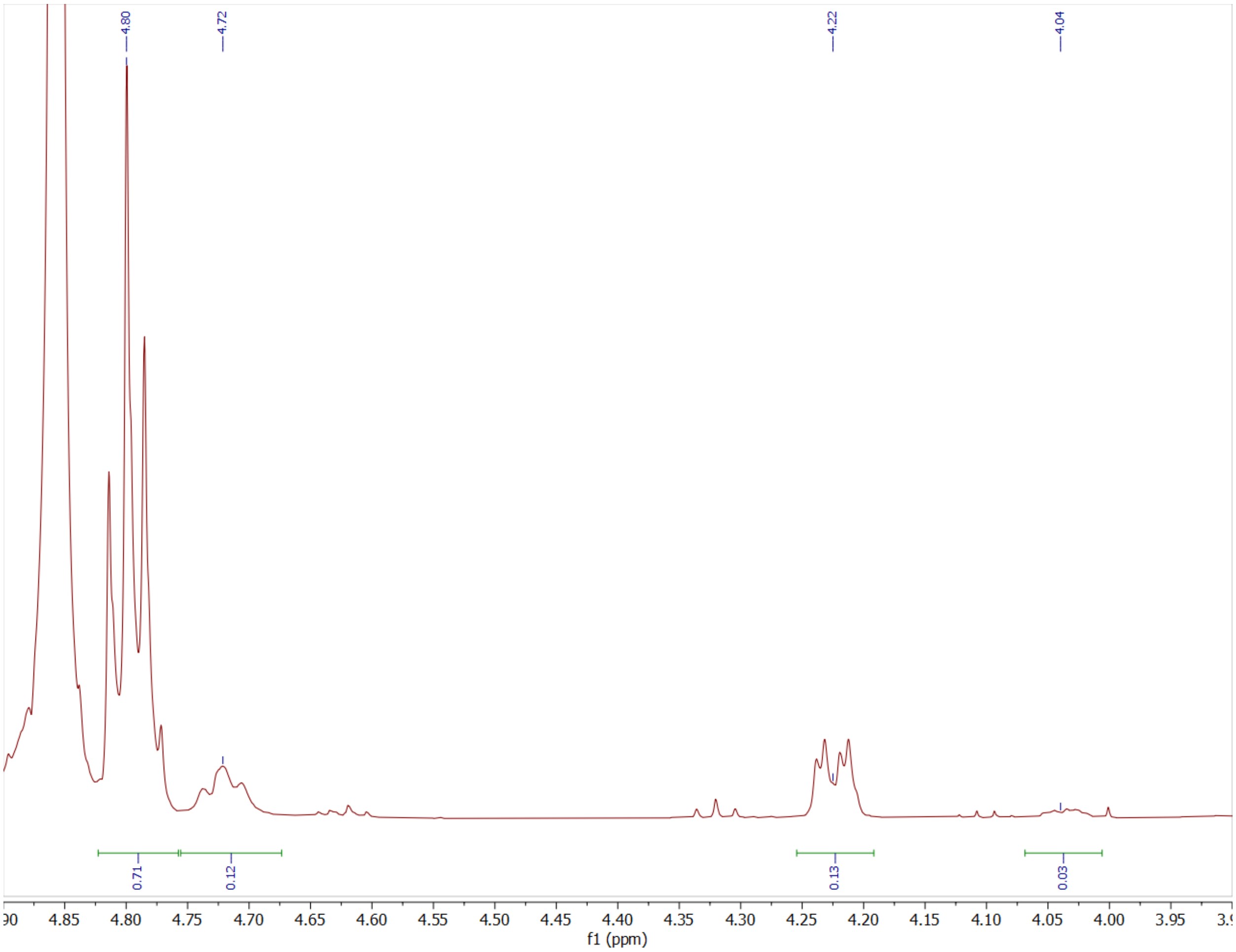

Figure S12: ^{1}H NMR depicting characteristic peaks for starting material, product, and hydrolysis products from an incomplete *N*-Boc deprotection of **13** (see Table S8, entry 3).

Table S8: Ratios of product, starting material, and hydrolysis byproducts in *N*-Boc-deprotection

entry	conditions	**13** : **8** : **8a** : **8b**	hydrolysis (%)[a]
1	TFA (10 equiv, neat, rt, 1.5 h)	messy	N/A
2	TFA (5 equiv, neat, 45 °C, 1.5 h)	messy	N/A
3	3 M HCl/MeOH (3 equiv, rt, 2 h)	12 : 71 : 13 : 3	18%
4	2 M HCl/Et_2O (3 equiv, rt, 3 h)	0 : 65 : 3 : 32	35%
5	1 M HCl/EtOAc (3 equiv, rt, 1 h)	64 : 30 : 2 : 5	19%
6	1 M MeOH/AcCl (3 equiv, rt, overnight)	0 : 50 : 32 : 17	49%
7	4 M HCl/dioxane (3 equiv, 0 – 10 °C, 3 h) in CH_3CN (0.5 M)	0 : 97 : 0 : 3	3%
**8**	**4 M HCl/dioxane (3 equiv, 0 – 10 °C, 3 h) in CH_3CN (0.5 M) + washing procedure**	**0 : >99 : 0 : 0**	**< 1%**

[a] Calculated as 100*(**8a** + **8b**)/(**8** + **8a** + **8b**)

4. Recovery of 2-mercaptopyridine for recycling purposes

Both thioesterification and amide bond formation described in the ms and SI (*vide supra*) generate 1 equiv of 2-mercaptopyridine, which is subsequently removed by washing with 1 M NaOH. A method of recovering 2-mercaptopyridine from these aqueous washes for purposes of recycling to make DPDTC is described below. The NaOH washes from the thioesterification reaction to form thioester **3** were used (see SI section 3.1).

The combined 1 M NaOH washes containing 2-mercaptopyridine were acidified to pH 5 using 1 M HCl, then NaCl was added (0.5 g) and the reaction was extracted with EtOAc (3 x 0.5 mL). The combined organic layers were concentrated *in vacuo* to afford 2-mercaptopyridine (21.9 mg, 79% recovery) as a yellow solid.

5. E Factor calculations

This work (7-step, 3-pot sequence):

Masses of waste and products are those from the 7-step, 3-pot sequence described in the "Methods" section of the manuscript. Masses were normalized to 1 mmol of starting material for the first pot, then subsequent pots were adjusted down in scale based on the yields of the preceding pots (e.g., pot 1 gave 78% yield, so pot 2 was normalized to 0.78 mmol). Purification steps were omitted as these are highly scale-dependent. The density of 5% $KHSO_4/H_2O$ was measured to be 1.03 g/mL.

First pot:

DPDTC = 0.2607 g
DMAP = 0.0122 g
EtOAc = 6.3140 g
5% $KHSO_4/H_2O$ = 3.0900 g
1 M NaOH = 6.2400 g
excess **4** = 0.0085 g
NMM = 0.1062 g
1 M HCl/H_2O = 3.0300 g
excess LiOH = 0.0479 g
THF = 0.8890 g
H_2O = 0.2000 g
Li = 0.0069 g

Second pot:

DPDTC = 0.2033 g
DMAP = 0.0096 g
EtOAc = 4.4861 g
NMM = 0.0788 g
5% $KHSO_4/H_2O$ = 1.7277 g
1 M NaOH = 1.7445 g

H_2O = 0.8387 g
HCl (from amine salt) = 0.0285 g

Third pot:
MeCN = 2.3052 g
4 M HCl/dioxane = 0.5775 g
Et_2O = 3.1366 g
excess TFAA = 0.1540 g
TFA (byproduct of trifluoroacetylation) = 0.0836 g
NMM = 0.1365 g
5% $KHSO_4/H_2O$ = 3.0208 g
H_2O = 2.9328 g
Boc group = 0.0742 g

Total waste (including aqueous) = 41.7438 g
Total waste (excluding aqueous) = 18.9193 g
Overall yield = 69.7%
Total product = 1 mmol x 0.4995 g/mmol x 0.697 = 0.3482 g
E Factor (including aqueous waste) = 41.7438/0.3482 = 120
E Factor (excluding aqueous waste) = 18.9193/0.3482 = 54

Literature method:[3]

Masses of waste and product were normalized to 1 mmol of starting material for the first step, then subsequent steps were adjusted down in scale based on the yields of the preceding steps. Purification steps were omitted.

First amide bond formation:
excess carboxylic acid = 0.0228 g
HCl (from amine salt) = 0.0365 g
DMF = 0.3768 g
MeCN = 2.8302 g
HATU = 0.4198 g
DIPEA = 0.3878 g
EtOAc = 2.2272 g
H_2O = 1.6461 g
1 M HCl/H_2O = 0.4156 g
brine = 0.4947 g

Ester hydrolysis:
THF = 0.8534 g
excess LiOH = 0.0459 g
H_2O = 0.2078 g
EtOAc = 1.1246 g
brine = 2.4977 g

Boc-deprotection:

HCl/dioxane (minus 1 equiv HCl) = 1.2308 g
DCM = 1.2826 g
Boc group = 0.0971 g
Li = 0.0067 g

Trifluoroacetylation:

MeOH = 0.4839 g
TEA = 0.3721 g
excess EtTFA = 0.0825 g
EtOH (from EtTFA) = 0.0438 g
HCl (from amine salt) = 0.0346 g
H_2O = 0.8486 g
1 M HCl/H_2O = 0.8571 g
EtOAc = 2.2962 g
brine = 1.2240 g

Second amide bond formation:

2-Hydroxypyridine *N*-oxide = 0.0186 g
excess amine hydrochloride = 0.0188 g
HCl (from amine salt) = 0.0246 g
MEK = 2.1890 g
DIPEA = 0.2616 g
EDCI = 0.1556 g
EtOAc = 1.1356 g
MTBE = 0.9316 g
H_2O = 1.0071 g
brine = 4.8423 g
1 M HCl/H_2O = 1.0172 g

Amide dehydration:

Burgess reagent = 0.4129 g
DCM = 4.3579 g
sat. $NaHCO_3/H_2O$ = 3.9320 g
brine = 5.0127 g
1 M HCl/H_2O = 2.4069 g
EtOAc = 1.6121 g
MTBE = 1.3226 g

Total waste (including aqueous) = 53.1060 g
Total waste (excluding aqueous) = 26.6962 g
Overall yield (excluding final purification step) = 49.6%
Total product = 1 mmol x 0.4995 g/mmol x 0.496 = 0.2478 g
E Factor (including aqueous waste) = 53.1060/0.2478 = 214
E Factor (excluding aqueous waste) = 26.6962/0.2478 = 108

6. Computational Studies

Theoretical Methods. Complete geometry optimizations and frequency calculations were carried out using density functional theory (DFT) methods at B3LYPD3BJ/6-31G(d) and B3LYPD3BJ/6-31+G(d,p) levels with Grimme's D3 empirical dispersion corrections and Becke-Johnson damping, and at the M062XD3/6-31+G(d,p)//6-31+G(d,p) level.[7-9] Geometry optimizations and frequency calculations at the B3LYPD3/6-31+G(d,p)-SMD level with were also done in acetonitrile, dimethylsulfoxide (DMSO), and water solvents. The SMD polarized-continuum model in solvent from Truhlar and Cramer was used for solvation calculations.[10]

Thermochemical data were calculated with zero-point energy corrections from scaled frequencies using a scaling factor of 0.99 for zero-point energies.[11] A scaling factor of 1.00 for frequencies for the thermal and entropy terms was used.[11ab] The quasiharmonic approximation was used for low frequencies to calculate entropies to avoid the large distortions found when many low-frequency vibrations are present in these large 'floppy' molecules.[11cd] Thus, frequencies below 100 cm^{-1} were treated as free rotors rather than by the harmonic approximation in calculating entropies. All calculations were performed using the Gaussian 16 program suite.[12] Thermochemical data and Cartesian coordinates are found in Table SD-1 in the Supplementary Data file for this paper.

Theoretical Results. The proton NMR spectra for nirmatrelvir in DMSO-*d*6 (see Fig. S13 below) show that it consists mainly of one component, but that a second component with closely-related NMR peaks is present as approximately 5% of the mixture. It is believed that this is a mixture of "syn" and "anti"rotamers about the tertiary amide in nirmatrelvir. This is documented for derivatives containing the tertiary amide functionality at the cyclopropanopyrrolidine ring in reports by Pfizer[3] and in the text and Supporting Information Part 1 of this paper.

Density functional theory (DFT) calculations were obtained to gain insight into the structural factors that lead to the formation of a pair of rotamers that differ in the rotation about the tertiary amide C-N bond. A search for the most likely conformational possibilities was begun at the B3LYPD3BJ/6-31G(d) level on 18 candidates, including conformational isomers from rotation about the C-C bond linkage to the side-chain pyrrolidone ring. For the best candidates, calculations were carried on with a larger 6-31+G(d,p) basis set and in different solvents. Geometries were reoptimized at each theoretical level and in each solvent, since the geometries changed significantly in the polar solvents, generally with longer hydrogen bonds, as expected. From this search emerged the four lowest-energy conformers discussed here. Their relative free energies at 298K are summarized in Table S9 and their optimized structures shown in Figs. S14-S23. The three best three conformers **A**, **B,** and **C** in the gas phase or acetonitrile or DMSO solvents all correspond to the major rotamer that we will label "syn" with the tertiary amide carbonyl syn to the side chain of the cyclopropanopyrrolidine. Perhaps as expected from steric and hydrogen bonding considerations, the tertiary amide carbonyl group of this major rotamer participates in a hydrogen bond to the NH of the pyrrolidine side-chain amide with 7-membered ring. This assignment of the major isomer is consistent with Overhauser experiments on nirmatrelvir in DMSO in the Pfizer paper and labelled "syn" there, with the amide carbonyl syn to the side chain.[3] The best conformer **D** for the minor rotamer, which we can label "anti" about the teriary amide bond, has two "hydrogen bonds" that are expected to be weak due to the long H---O distances and major distortion away from linearity.

The free energy data in Table S9 in acetonitrile and DMSO support the observation that one rotamer should be predominant, mainly made up of structures **A** and **B**, both of which are of very similar structure (with small differences in the rotation of the CF3 group) and not distinguishable at some levels of theory. The third structure **C**, however, becomes the main structure for the major rotamer in the gas phase and has a different conformation for the the side-chain pyrrolidone ring. The calculations in the gas-phase give consistent results with the larger cc-pVTZ basis set and with both the B3LYP and M06-2X methods. Curiously, and perhaps significantly in biological function, the rotamer structure **D** is predicted to become the major rotamer in water solution and **A** and **C** to have energies very similar to one another.

Table S9: Calculated relative electronic energies, enthalpies, and free energies at 298 K for selected conformers of nirmatrelvir (Paxlovid) optimized at B3LYPD3BJ/6-31+G(d,p), and M06-2XD3/6-31+G(d,p) levels of theory. Conformers **A**, **B**, and **C** all correspond to the "syn" rotamer at the tertiary amide (C=O syn to side chain), while **D** is "anti".[a]

	Gas phase					Solvent [B3LYPD3BJ-SMD]		
	B3LYPD3BJ			cc-pVTZ[b]	M062XD3	MeCN	DMSO	water
Conformer	ΔE°_{e}	ΔH°_{298K}	ΔG°_{298K}	ΔE°_{e}	ΔE°_{e}	ΔG°_{298K}	ΔG°_{298K}	ΔG°_{298K}
**A**	0	0	0	0	0	0	0	0
**B**	-0.02	-0.02	0	0.04	0.18	0.12	0.14	0
**C**	-1.5	-1.38	-1.35	-1.44	-1.25	0.41	0.42	-0.05
**D**	0.62	1.92	0.47	0.55	-0.22	0.48	0.83	-1.15

a) All values in kcal mol^{-1}.
b) B3LYPD3BJ/cc-pVTZ at B3LYPD3BJ/6-31+G(d,p) optimized geometry. All energies and thermochemistry for other theoretical levels are from optimized geometries and frequencies at the same respective theoretical levels.

Gas-phase and acetonitrile solution structures are shown in Figs. S14-S23 for the main conformers and transition states for the tertiary amide rotation below. Cyclic hydrogen bonds, one or more, with 7-, 8- 10-, 13-, and 15-membered rings (counting the H), are present in the full range of structures considered, but not all shown. While the hydrogen bonds, weak and strong, must be a factor in determining the best structures, no simple, clear pattern of relative stability is evident.

The changes in the order of stabilities of the main conformers, **A**, **B**, **C**, and **D**, in different solvents are curious. While it is hard to say exactly why one conformer is better solvated than another, we can offer some explanation of that behavior here. We note, to start, that the structures optimized in the gas-phase, acetonitrile and water are all similar to those shown in the figures. The free energies of solvation in Table S10 and the order of the stabilities in different solvents in Table S9 correlate well with the overall molecular dipole moments which should be a factor in the solvation. Additionally, one may consider specific structural features in Figs. S15, S19 and S21 that can lead to solvation energy differences. For example, there is no good internal hydrogen bond for conformer **D** which would be expected make its amide groups more accessible to water solvent and more strongly solvated than in conformers **A** or **C,** explaining, along with the dipole moment effect, why conformer **D** could become the favored form in water. Conformers **A** and **C** both have similarly strong internal hydrogen bonds, but differ in the orientation of the pyrrolidone ring. For

conformer **C**, the carbonyl group of the pyrrolidone amide is directed toward the *t*-butyl group of the adjacent side chain, helping to make it the least well solvated of the three conformers in Table S10. This orientation of the carbonyl dipole in the pyrrolidone ring in **C** also produces the most dipole vector cancellation with the nitrile dipole, leading to the smallest overall dipole moment and poorest solvation energy. Furthermore, this orientation in **C** has less dipole repulsion between the nitrile and the pyrrolidone carbonyl than in conformers **A** and **D**, which should help make it the most stable conformer in the gas phase.

Table S10: Calculated molecular total dipole moments (μ) and free energies of solvation at 298 K for selected conformers of nirmatrelvir (Paxlovid) at optimized B3LYPD3BJ/6-31+G(d,p)/SMD geometries for each solvent and the gas phase. Conformers **A** and **C** correspond to the "syn" rotamer at the tertiary amide (C=O syn to side chain), while **D** is "anti".

Conformer	Gas phase μ (D)	Solvent MeCN μ (D)	MeCN ΔG°_{solv} kcal mol^{-1}	water μ (D)	water ΔG°_{solv} kcal mol^{-1}
**A**	5.079	8.692	31.40	8.007	27.23
**C**	3.876	4.925	29.60	5.486	25.99
**D**	5.578	11.866	31.45	15.533	28.83

The rotational free energy barrier for nirmatrelvir in acetonitrile was calculated at the B3LYPD3/6-31+G(d,p)-SMD(MeCN) level to be 25.25 kcal mol^{-1} at 298 K, significantly higher than the experimental barrier of 17.77 kcal mol^{-1} for acetamide in acetonitrile solvent.[13] This is consistent with the suggestion that the barrier to rotation about the tertiary amide of nirmatrelvir is unusually large.[3] For a comparison at the same theoretical level as for nirmatrelvir in acetonitrile, the calculated barrier for acetamide was 18.15 kcal mol^{-1} at 298 K. For DMF in acetonitrile, the experimental barrier is 20.63 kcal mol^{-1} at 298 K vs. a calculated value of 22.00 kcal mol^{-1}. At the G4 level of theory in the gas phase with thermochemistry from B3LYPD3/6-311G(d,p) frequencies, the calculated free energy barriers are 14.72 kcal mol^{-1} at 298 K for DMA and 19.18 kcal mol^{-1} at 298 K for DMF vs. experimental values of 15.33 kcal mol^{-1} at 323 K for DMA and 19.25 kcal mol^{-1} at 298 K for DMF, a very good fit. The two best conformations, of nine candidates with both clockwise and counterclockwise rotations, for the tertiary amide C-N bond rotation were structures **TS1** and **TS2-2** (see Figs. S22 and S23) with quite different structures, but very similar energies. Both geometries were optimized at the B3LYPD3/6-31+G(d,p)-SMD(MeCN) level, but with **TS1** having a slightly lower free energy barrier by 0.06 kcal mol^{-1} at 298K in acetonitrile.

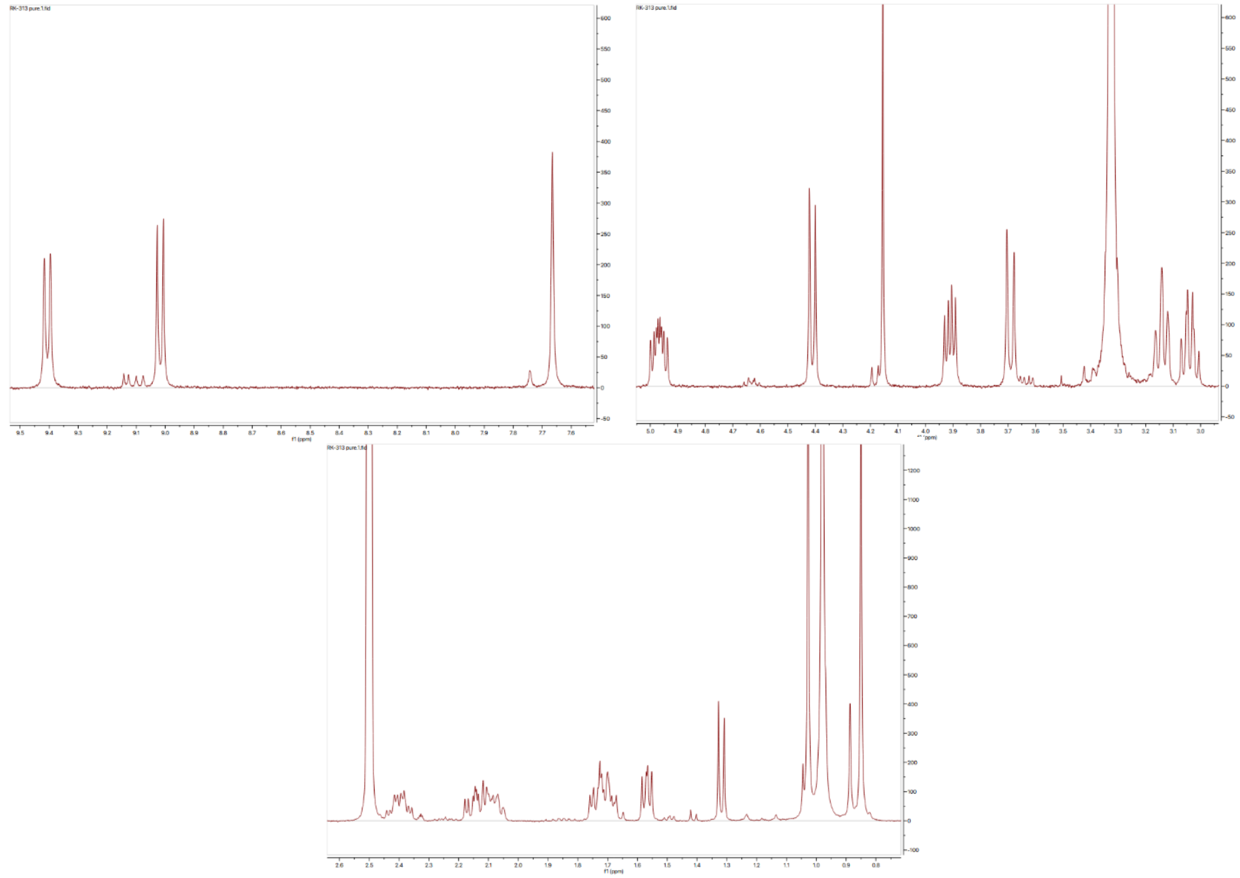

Figure S13: Proton NMR spectrum of nirmatrelvir (**1**) in DMSO-d6 showing the presence of the minor rotamer at about 5% of the mixture.

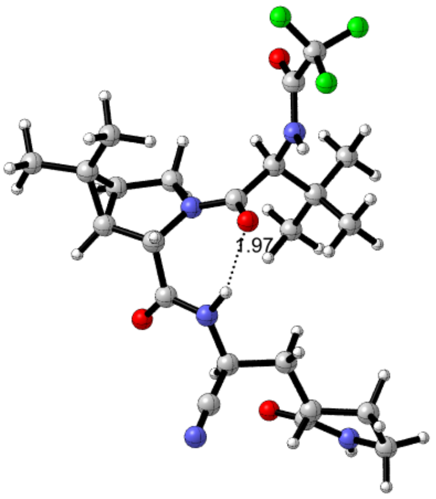

Figure S14: B3LYPD3BJ/6-31+G(d,p) optimized structure for **Conformer A**. Hydrogen bond O-H distance=1.97 Å. Atom colors: nitrogen, blue; oxygen, red; fluorine, green.

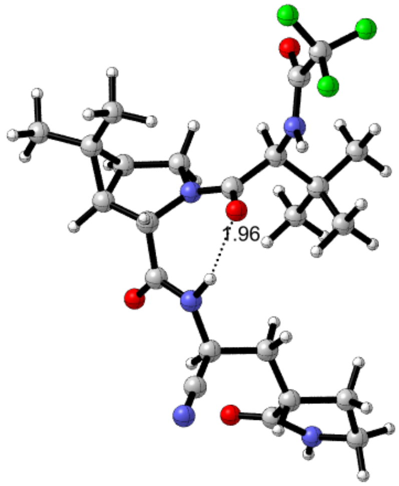

Figure S15: B3LYPD3BJ/6-31+G(d,p)/SMD(MeCN) optimized structure for **Conformer A**. Hydrogen bond O-H distance=1.96 Å. Atom colors: nitrogen, blue; oxygen, red; fluorine, green.

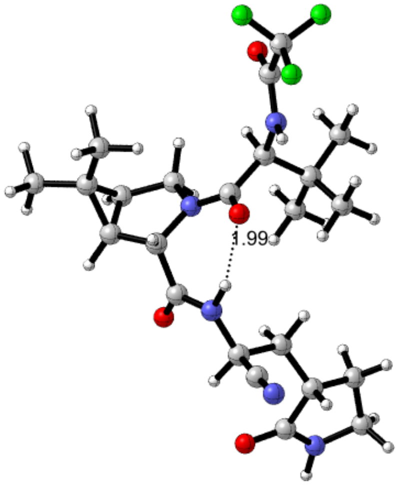

Figure S16: B3LYPD3BJ/6-31+G(d,p) optimized structure for **Conformer B**. Hydrogen bond O-H distance=1.99 Å. Atom colors: nitrogen, blue; oxygen, red; fluorine, green.

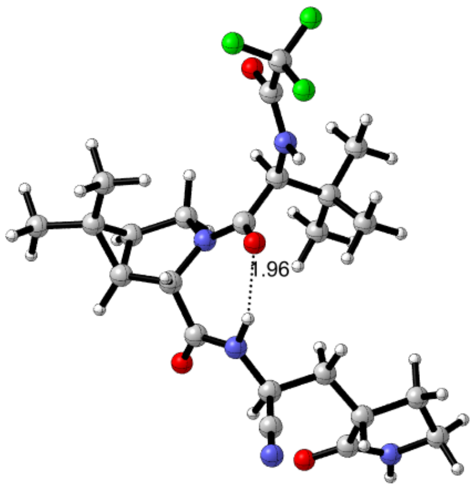

Figure S17: B3LYPD3BJ/6-31+G(d,p)/SMD(MeCN) optimized structure for **Conformer B**. Hydrogen bond O-H distance=1.96 Å. Atom colors: nitrogen, blue; oxygen, red; fluorine, green.

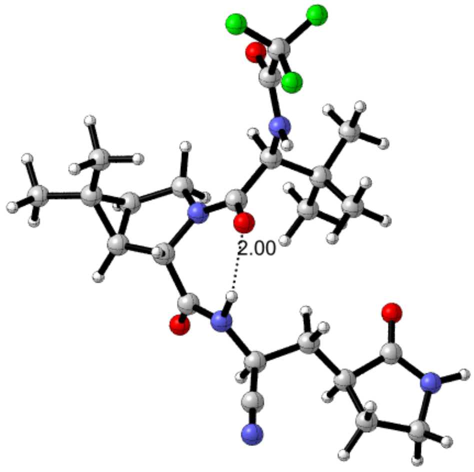

Figure S18: B3LYPD3BJ/6-31+G(d,p) optimized structure for **Conformer C**. Hydrogen bond O-H distance=2.00 Å. Atom colors: nitrogen, blue; oxygen, red; fluorine, green.

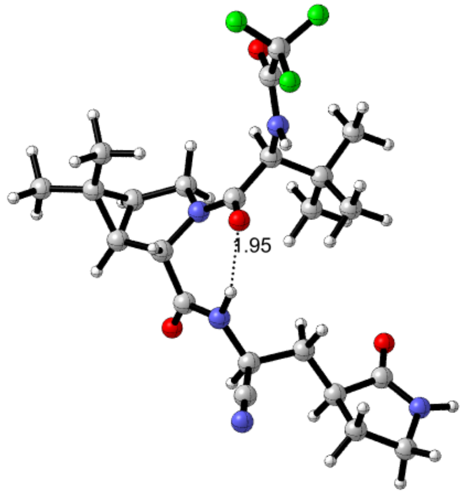

Figure S19: B3LYPD3BJ/6-31+G(d,p)/SMD(MeCN) optimized structure for **Conformer C**. Hydrogen bond O-H distance=1.95 Å. Atom colors: nitrogen, blue; oxygen, red; fluorine, green.

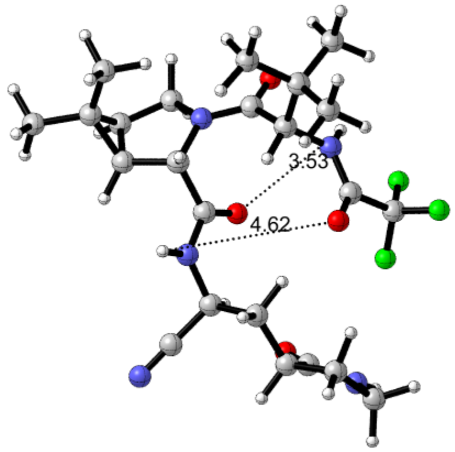

Figure S20: B3LYPD3BJ/6-31+G(d,p) optimized structure for **Conformer D**. Weak hydrogen bond O-H distances=3.527, 4.62 Å. Atom colors: nitrogen, blue; oxygen, red; fluorine, green.

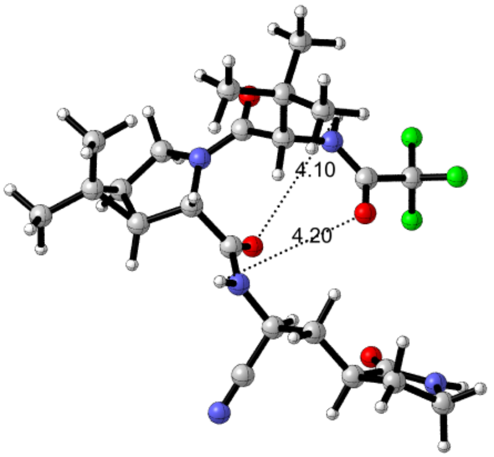

Figure S21: B3LYPD3BJ/6-31+G(d,p)/SMD(MeCN) optimized structure for **Conformer D**. Weak hydrogen bond O-H distances=4.10, 4.20 Å. Atom colors: nitrogen, blue; oxygen, red; fluorine, green.

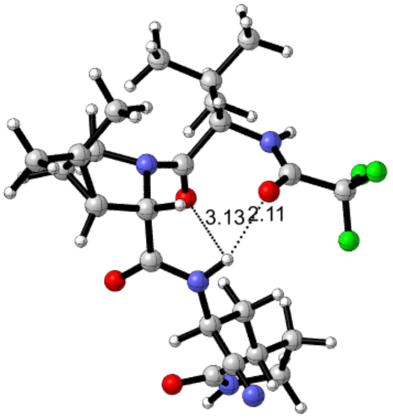

Figure S22: B3LYPD3BJ/6-31+G(d,p)/SMD(MeCN) optimized structure for **TS1**. Weak hydrogen bond O-H distances=2.11, 3.13 Å. Atom colors: nitrogen, blue; oxygen, red; fluorine, green.

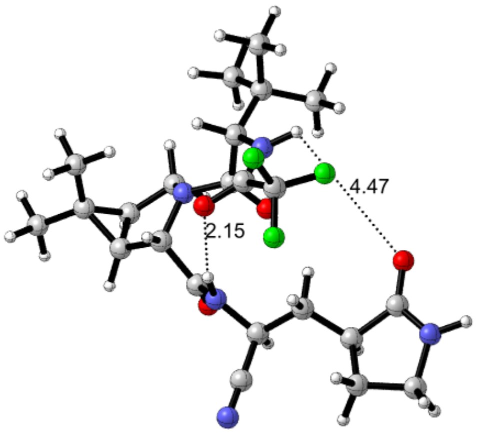

Figure S23: B3LYPD3BJ/6-31+G(d,p)/SMD(MeCN) optimized structure for **TS2**. Weak hydrogen bond O-H distances=2.15, 4.47 Å. Atom colors: nitrogen, blue; oxygen, red; fluorine, green.

7. Supplementary References

1. Cortes-Clerget, M.; Spink S. E.; Gallagher, G. P.; Chaisemartin, L.; Filaire, E.; Berthon, J. Y.; Lipshutz, B. H. MC-1. A "designer" surfactant engineered for peptide synthesis in water at room temperature. *Green Chem.* **2019**, *21*, 2610-2614.
2. Lipshutz, B. H.; Ghorai, S.; Abela, A. R.; Moser, R.; Nishikata, T.; Duplais, C.; Krasovskiy, A.; Gaston, R. D.; Gadwood, R. C. TPGS-750-M: A Second-Generation Amphiphile for Metal-Catalyzed Cross-Couplings in Water at Room Temperature. *J. Org. Chem.* **2011**, *76*, 4379-4391.
3. Owen, D. R.; Allerton, C. M. N.; Anderson, A. S.; Aschenbrenner, L.; Avery, M.; Berritt, S.; Boras, B.; Cardin, R. D.; Carlo, A.; Coffman, K. J.; Dantonio, A.; Di, L.; Eng, H.; Ferre, R.; Gajiwala, K. S.; Gibson, S. A.; Greasley, S. E.; Hurst, B. L.; Kadar, E. P.; Kalgutkar, A. S.; Lee, J. C.; Lee, J.; Liu, W.; Mason, S. W.; Noell, S.; Novak, J. J.; Obach, R. S.; Ogilvie, K.; Patel, N. C.; Pettersson, M.; Rai, D. K.; Reese, M. R.; Sammons, M. F.; Sathish, J. G.; Singh, R. S. P.; Steppan, C. M.; Stewart, A. E.; Tuttle, J. B.; Updyke, L.; Verhoest, P. R.; Wei, L.; Yang, Q.; Zhu, Y. An Oral SARS-CoV-2 M pro Inhibitor Clinical Candidate for the Treatment of COVID-19. *Science* **2021**, *374*, 1586–1593.
4. Wood, A. B.; Kincaid, J. R. A.; Lipshutz, B. H. Dehydration of primary amides to nitriles in water. Late-stage functionalization and 1-pot multistep chemoenzymatic processes under micellar catalysis conditions. *Green Chem.* **2022**, *24*, 2853-2858.
5. Okabe, H.; Naraoka, A.; Isogawa, T.; Oishi, S.; Naka, H. Acceptor-Controlled Transfer Dehydration of Amides to Nitriles. *Org. Lett.* **2019**, *21*, 4767-4770.
6. Karmakar, A.; Basha, M.; Venkatesh Babu, G. T.; Botlagunta, M.; Malik, N. A.; Rampulla, R.; Mathur, A.; Gupta, A. K. Tertiary-Butoxycarbonyl (Boc) – A Strategic

Group for *N*-Protection/Deprotection in the Synthesis of Various Natural/Unnatural *N*-Unprotected Aminoacid Cyanomethyl Esters. *Tetrahedron Lett.* **2018**, *59*, 4267–4271.

7. A. D. Becke, *J. Chem. Phys*. **1993**, *98*, 5648. S. Grimme, S. Ehrlich, L. Goerigk, *J. Comp. Chem*. **2011**, ***32*****,** 1456-1465.
8. Y. Zhao; D. G. Truhlar, *Theor. Chem. Acc.* **2008**, *120***,** 215-241.
9. H. S. Yu, X. He, S. L. Li, D. G. Truhlar, *Chem. Sci.* **2016**, *7*, 5032.
10. A. V. Marenich, C. J. Cramer, and D. G. Truhlar, *J. Phys. Chem. B* **2009**, *113*, 6378-96.
11. (a) A. P. Scott, L. Radom *J. Phys. Chem*. **1996**, *100*, 16502. (b) I. M. Alecu, J. Zheng, Y. Zhao, D. G. Truhlar,. *J. Chem. Theory Comput.* 2010**, 6***,* 2872–2887. (c) R. F. Ribeiro, A. V. Marenich, C. J. Cramer, D. G. Truhlar *J. Phys. Chem. A* 2011, **115**, 14556-14562. (d) S. Grimme *Chem. Eur. J.* **2012**, *18*, 9955-9964. (e) D. H. Aue, to be published. We have evaluated optimum scale factors for experimental fundamental frequencies for numerous organic molecules and find that the DFT scale factors differ with basis set between 0.96 for the B3LYP/6-31G(d) level and 0.954 for M06-2X/ 6-311+G(d,p) level to 0.963 for the B3LYP/ 6-31+G(d,p) level and 0.967 for the B3LYP/ 6-311G(d,p) level. For thermal terms and entropies, such variation has little effect, and one may argue that a scaling factor close to 1.00 might be best for the dominant low frequencies (we get 1.005 for 111 frequencies below 150 cm^{-1})(see also ref. 5a). For zero-point energies determined from experimental fundamental values and CCSD(T)-F12/cc-aug-pVDZ harmonic values from the Morse approximation, the scale factors 1.00 (B3LYP/6-311G(d,p) and B3LYP/6-31+G(d,p)), 0.984 (M06-2X/6-311+G(d,p)), 0.991 (M06/6-31G(d)), 0.996 (M06/6-31+G(d,p)) give the best fit for large variety of organic molecules.
12. Gaussian 16, Revision A.03, M. J. Frisch, G. W. Trucks, H. B. Schlegel, G. E. Scuseria, M. A. Robb, J. R. Cheeseman, G. Scalmani, V. Barone, G. A. Petersson, H. Nakatsuji, X. Li, M. Caricato, A. V. Marenich, J. Bloino, B. G. Janesko, R. Gomperts, B. Mennucci, H. P. Hratchian, J. V. Ortiz, A. F. Izmaylov, J. L. Sonnenberg, D. Williams-Young, F. Ding, F. Lipparini, F. Egidi, J. Goings, B. Peng, A. Petrone, T. Henderson, D. Ranasinghe, V. G. Zakrzewski, J. Gao, N. Rega, G. Zheng, W. Liang, M. Hada, M. Ehara, K. Toyota, R. Fukuda, J. Hasegawa, M. Ishida, T. Nakajima, Y. Honda, O. Kitao, H. Nakai, T. Vreven, K. Throssell, J. A. Montgomery, Jr., J. E. Peralta, F. Ogliaro, M. J. Bearpark, J. J. Heyd, E. N. Brothers, K. N. Kudin, V. N. Staroverov, T. A. Keith, R. Kobayashi, J. Normand, K. Raghavachari, A. P. Rendell, J. C. Burant, S. S. Iyengar, J. Tomasi, M. Cossi, J. M. Millam, M. Klene, C. Adamo, R. Cammi, J. W. Ochterski, R. L. Martin, K. Morokuma, O. Farkas, J. B. Foresman, D. J. Fox, Gaussian, Inc., Wallingford CT, 2016.
13. K. B. Wiberg, P. R. Rablen, G. J. Rush, T. A. Keith, *J. Am. Chem. Soc.* **1995**, *117*, 4261-4270.
14. Lee, J. I.; Park, H. A Convenient Synthesis of *N*-Methoxy-*N*-methylamides from Carboxylic Acids Using *S,S*-Di(2-pyridyl) Dithiocarbonate. *Bull. Korean Chem. Soc.* **2001**, *22*, 421-423.
15. Owen, D. R.; Pettersson, M. Y.; Reese, M. R.; Sammons, M. F.; Tuttle, J. B.; Verhoest, P. R.; Wei, L.; Yang, Q.; Yang, X. Nitrile-Containing Antiviral Compounds. U.S. Patent US 20220062232A1, March 3, 2022.

8. Supplementary Note 1: Experimental data

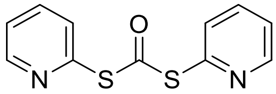

***S,S*-Di(pyridin-2-yl) carbonodithioate (DPDTC): ^{1}H NMR (400 MHz, $CDCl_3$) δ** 8.58 (ddd, *J* = 4.9, 2.0, 0.9 Hz, 1H), 7.78 – 7.59 (m, 2H), 7.27 (ddd, *J* = 7.3, 5.4, 1.4 Hz, 1H). **^{13}C NMR (101 MHz, $CDCl_3$) δ** 185.7, 150.7, 150.7, 137.5, 130.5, 124.2. **R_f:** 0.13 (30% EtOAc/hexanes). Spectral data matched those previously reported.[14]

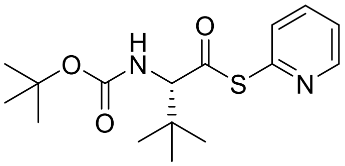

***S*-(Pyridin-2-yl) (*S*)-2-((*t*-butoxycarbonyl)amino)-3,3-dimethylbutanethioate (3): ^{1}H NMR (400 MHz, CD_3CN) δ** 8.60 (ddd, *J* = 4.8, 2.0, 0.9 Hz, 1H), 7.81 (td, *J* = 7.7, 1.9 Hz, 1H), 7.54 (dt, *J* = 7.8, 1.0 Hz, 1H), 7.37 (ddd, *J* = 7.6, 4.8, 1.2 Hz, 1H), 5.96 (d, *J* = 7.7 Hz, 1H), 4.10 (d, *J* = 8.8 Hz, 1H), 1.47 (s, 9H), 1.05 (s, 9H)**. ^{13}C NMR (101 MHz, CD_3CN) δ** 199.9, 156.8, 152.8, 151.6, 138.4, 131.7, 124.9, 80.6, 70.0, 35.2, 28.7, 27.2. **R_f:** 0.27 (30% EtOAc/hexanes). **HRMS (ESI):** *m/z* calcd for $C_{16}H_{24}N_2O_3S+Na^+$: 347.1405 $[M+Na]^+$: found 347.1400.

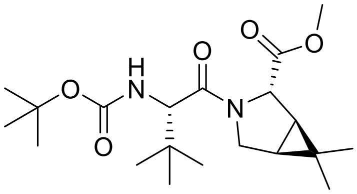

Methyl (1*R*,2*S*,5*S*)-3-((*S*)-2-((*tert*-butoxycarbonyl)amino)-3,3-dimethylbutanoyl)-6,6-dimethyl-3-azabicyclo[3.1.0]hexane-2-carboxylate (5): ^{1}H NMR (500 MHz, DMSO-*d_6*) δ 6.63 (d, *J* = 9.5 Hz, 1H), 4.23 (s, 1H), 4.08 (d, *J* = 9.5 Hz, 1H), 3.95 (d, *J* = 10.5 Hz, 1H), 3.83 – 3.76 (m, 1H), 3.65 (s, 3H), 1.54-1.49 (m, 1H), 1.41 (d, *J* = 7.5 Hz, 1H), 1.36 (s, 9H), 1.01 (s, 3H), 0.94 (s, 9H), 0.86 (s, 3H). **^{13}C NMR (126 MHz, DMSO-*d_6*) δ** 171.4, 170.2, 155.9, 78.1, 58.7, 58.5, 51.8, 47.0, 34.0, 29.6, 28.0, 27.0, 26.19, 25.7, 18.9, 12.1. **R_f:** 0.42 (30% EtOAc/hexanes, I_2 stain). Spectral data matched those previously reported.[3]

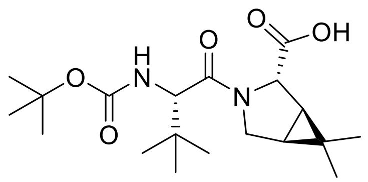

(1*R*,2*S*,5*S*)-3-((*S*)-2-((*t*-Butoxycarbonyl)amino)-3,3-dimethylbutanoyl)-6,6-dimethyl-3-azabicyclo[3.1.0]hexane-2-carboxylic acid (6): ^{1}H NMR (500 MHz, DMSO-*d$_6$*) δ 12.62 (s, 1H), 6.64 (d, *J* = 9.5 Hz, 1H), 4.13 (s, 1H), 4.05 (d, *J* = 9.6 Hz, 1H), 3.91 (d, *J* = 10.5 Hz, 1H), 3.77 (dd, *J* = 10.3, 5.3 Hz, 1H), 1.52-1.46 (m, 1H), 1.39 (d, *J* = 7.5 Hz, 1H), 1.35 (s, 9H), 1.01 (s, 3H), 0.94 (s, 9H), 0.84 (s, 3H). **^{13}C NMR (126 MHz, DMSO-*d$_6$*) δ** 172.6, 170.0, 155.9, 78.2, 58.9, 58.5, 47.0, 34.1, 29.8, 28.1, 26.9, 26.3, 25.9, 18.8, 12.2. **R$_f$:** 0.30 (10% MeOH/CH_2Cl_2, bromocresol green stain). Spectral data matched those previously reported.[3]

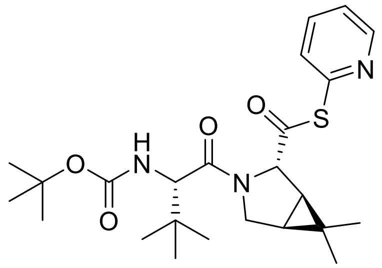

***S*-(Pyridin-2-yl) (1*R*,2*S*,5*S*)-3-((*S*)-2-((*tert*-butoxycarbonyl)amino)-3,3-dimethylbutanoyl)-6,6-dimethyl-3-azabicyclo[3.1.0]hexane-2-carbothioate (7): ^{1}H NMR (500 MHz, DMSO-*d$_6$*) δ** 8.61 (d, *J* = 6.4 Hz, 1H), 7.90 (td, *J* = 7.8, 2.0 Hz, 1H), 7.62 (d, *J* = 7.8 Hz, 1H), 7.45 (dd, *J* = 7.6, 4.7 Hz, 1H), 6.91 (d, *J* = 9.6 Hz, 1H), 4.40 (s, 1H), 4.14 (d, *J* = 9.6 Hz, 1H), 4.03 (d, *J* = 10.7 Hz, 1H), 3.91 (dd, *J* = 10.6, 5.4 Hz, 1H), 1.69 – 1.60 (m, 1H), 1.55 (d, *J* = 7.6 Hz, 1H), 1.36 (s, 9H), 1.04 (s, 3H), 0.98 (s, 9H), 0.86 (s, 3H). **^{13}C NMR (126 MHz, DMSO-*d$_6$*) δ** 196.9, 171.0, 155.9, 150.5, 150.3, 137.8, 130.3, 124.1, 78.3, 66.5, 58.6, 47.5, 34.4, 30.7, 28.1, 27.2, 26.41, 25.7, 19.5, 12.3. **R$_f$:** 0.29 (30% EtOAc/Hexanes). **HRMS (ESI):** *m/z* calcd for $C_{24}H_{35}N_3O_4S+Na^+$: 484.2241 [*M*+Na]$^+$ found: 484.2257.

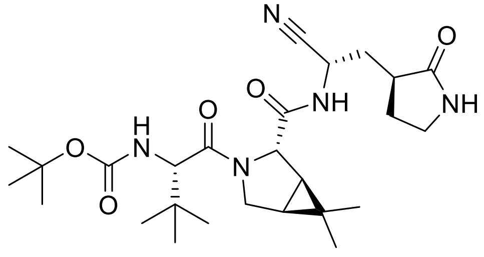

***t*-Butyl ((*S*)-1-((1*R*,2*S*,5*S*)-2-(((*S*)-1-cyano-2-((*S*)-2-oxopyrrolidin-3-yl)ethyl)carbamoyl)-6,6-dimethyl-3-azabicyclo[3.1.0]hexan-3-yl)-3,3-dimethyl-1-oxobutan-2-yl)carbamate (9): ^{1}H NMR (500 MHz, DMSO-*d$_6$*) δ** 8.98 (d, *J* = 8.5 Hz, 1H), 7.65 (s, 1H), 6.68 (d, *J* = 9.2 Hz, 1H), 4.95 (ddd, *J* = 11.0, 8.5, 5.2 Hz, 1H), 4.15 (s, 1H), 4.00 (d, *J* = 9.3 Hz, 1H), 3.88 (d, *J* = 10.4 Hz, 1H), 3.83 (dd, *J* = 10.2, 5.3 Hz, 1H), 3.13 (t, *J* = 9.2 Hz, 1H), 3.03 (td, *J* = 9.5, 7.1 Hz, 1H), 2.41

(tt, *J* = 10.5, 5.1 Hz, 1H), 2.15 (ddd, *J* = 13.4, 10.8, 4.4 Hz, 1H), 2.11 – 2.02 (m, 1H), 1.78 – 1.62 (m, 2H), 1.53 (dd, *J* = 7.8, 5.2 Hz, 1H), 1.34 (s, 9H), 1.27 (d, *J* = 7.6 Hz, 1H), 1.02 (s, 3H), 0.91 (s, 9H), 0.87 (s, 3H). **^{13}C NMR (101 MHz, DMSO-*d*$_6$) δ** 177.5, 171.0, 170.0, 156.0, 119.7, 78.2, 59.7, 58.7, 47.4, 37.7, 36.7, 34.1, 34.0, 30.3, 28.1, 27.5, 26.9, 26.4, 25.8, 18.9, 12.4. **R$_f$:** 0.25 (2% MeOH/CH_2Cl_2, CAM stain). **HRMS (ESI):** *m/z* calcd for $C_{26}H_{41}N_5O_5$+Na^+: 526.3005 [*M*+Na]$^+$ found: 526.3005.

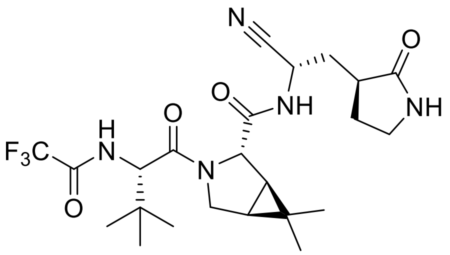

(1*R*,2*S*,5*S*)-N-((*S*)-1-Cyano-2-((*S*)-2-oxopyrrolidin-3-yl)ethyl)-3-((*S*)-3,3-dimethyl-2-(2,2,2-trifluoroacetamido)butanoyl)-6,6-dimethyl-3-azabicyclo[3.1.0]hexane-2-carboxamide (1; nirmatrelvir): ^{1}H NMR (500 MHz, DMSO-*d*$_6$) δ 9.40 (d, *J* = 8.5 Hz, 1H), 9.02 (d, *J* = 8.5 Hz, 1H), 7.67 (s, 1H), 4.97 (ddd, *J* = 11.0, 8.5, 5.2 Hz, 1H), 4.41 (d, *J* = 8.4 Hz, 1H), 4.16 (s, 1H), 3.91 (dd, J = 10.3, 5.6 Hz, 1H), 3.69 (d, *J* = 10.5 Hz, 1H), 3.14 (t, *J* = 8.6 Hz, 1H), 3.04 (td, *J* = 9.4, 7.0 Hz, 1H), 2.47 – 2.33 (m, 1H), 2.21 – 2.02 (m, 2H), 1.79 – 1.63 (m, 2H), 1.57 (dd, *J* = 7.7, 5.4 Hz, 1H), 1.32 (d, *J* = 7.8 Hz, 1H), 1.03 (s, 3H), 0.98 (s, 9H), 0.85 (s, 3H). **^{13}C NMR (126 MHz, DMSO-*d*$_6$) δ** 177.5, 170.7, 167.5, 157.0 (q, *J* = 36.5 Hz), 119.6, 115.8 (q, *J* = 288.0 Hz), 60.1, 58.2, 47.6, 37.8, 36.7, 34.6, 34.2, 30.3, 27.4, 26.9, 26.3, 25.7, 18.9, 12.3. **^{19}F NMR (471 MHz, DMSO-*d*$_6$) δ** -72.94. **R$_f$:** 0.55 (5% MeOH/CH_2Cl_2, CAM stain). Spectral data matched those previously reported.[3]

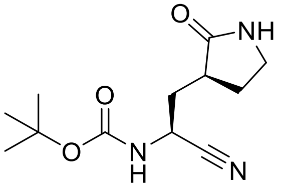

***t*-Butyl ((*S*)-1-cyano-2-((*S*)-2-oxopyrrolidin-3-yl)ethyl)carbamate (13): ^{1}H NMR (600 MHz, CD_3OD) δ** 4.72 (dd, *J* = 10.2, 8.2 Hz, 1H), 3.40 – 3.32 (m, 2H, partially obscured by solvent peak), 2.59-2.44 (m, 1H), 2.36 (dddd, *J* = 11.8, 8.6, 6.7, 3.0 Hz, 1H), 2.21 (ddd, *J* = 15.0, 9.3, 5.7 Hz, 1H), 1.92 – 1.80 (m, 2H), 1.46 (s, 9H). **^{13}C NMR (101 MHz, CD_3OD) δ** 181.1, 157.2, 120.5, 81.6, 41.9, 41.6, 39.3, 35.5, 28.7. **R$_f$:** 0.65 (10% MeOH/CH_2Cl_2, I_2 stain). Spectral data matched those previously reported.[15]

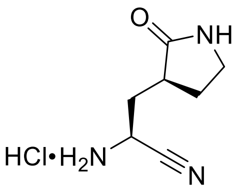

(*S*)-2-Amino-3-((*S*)-2-oxopyrrolidin-3-yl)propanenitrile hydrochloride (8): **^{1}H NMR (400 MHz, CD_3OD) δ** 4.81 (dd, apparent triplet, *J* = 7.3, 7.3 Hz, 1H), 3.45 – 3.35 (m, 2H), 2.78 (ddt, *J* = 10.8, 8.7, 7.3 Hz, 1H), 2.52 – 2.37 (m, 1H), 2.22 (t, *J* = 7.5 Hz, 2H), 1.91 (ddt, *J* = 12.6, 10.8, 9.2 Hz, 1H). **^{13}C NMR (101 MHz, CD_3OD) δ** 180.9, 117.2, 42.2, 41.9, 41.1, 34.2, 29.4. **R_f:** N/A for hydrochloride salt.
