# Supplementary material for: A sustainable synthesis of the SARS-CoV-2 Mpro inhibitor nirmatrelvir, the active ingredient in Paxlovid
Source: Commun Chem. 2022 Nov 21;5:156. doi: 10.1038/s42004-022-00758-5 (PMC9685088; doi:10.1038/s42004-022-00758-5)
Supplement: Supplementary file 2 — Supplementary Data 1 [file 42004_2022_758_MOESM2_ESM.pdf]

A Sustainable Synthesis of the SARS-CoV-2 M^{pro} Inhibitor Nirmatrelvir, the Active Ingredient in Paxlovid

Joseph R. A. Kincaid, Juan C. Caravez, Karthik S. Iyer, Rahul D. Kavthe, Nico Fleck, Donald H. Aue,* and Bruce H. Lipshutz*

Department of Chemistry and Biochemistry, University of California, Santa Barbara, CA 93106 USA

Phone: 805-893-2521
Fax: 805-893-8265
Email: aue@chem.ucsb.edu
Email: lipshutz@chem.ucsb.edu
Website: https://lipshutz.chem.ucsb.edu/

Supplementary Data 1

Computational Studies

Table SD-1. Thermodynamic parameters from unscaled frequencies and Cartesian coordinates from optimized structures at various levels of theory.

Conformer **A**: B3LYPD3BJ/6-31+G(d,p)

Processing: nirc6pbed3j.log
PG=C01

Method	BasisSet	Imaginary Freqs
RB3LYP	6-31+G(d,p)	0

HF Energy
-1770.1930390

ZPE	E298	S298	Squasihar	Equasihar	Strans	Srot
344.42362	367.132	234.504	204.649	367.252	44.511	37.624

Label	Frequencies	IR Inten	Raman Activ	Depolar (P)	Depolar (U)	Dipole
A	3643.5793	45.1402	163.9387	0.2325	0.3772	5.0792
A	3613.3868	66.3749	40.6568	0.0765	0.1422	
A	3500.7915	172.5339	96.4886	0.1452	0.2536	
A	3215.0745	1.8873	91.7950	0.1441	0.2519	
A	3185.6006	11.0760	114.7754	0.2777	0.4347	
A	3147.4041	3.8851	35.9732	0.0767	0.1425	
A	3145.4090	19.4871	66.4349	0.6107	0.7583	
A	3143.0186	24.0036	53.2056	0.5626	0.7201	
A	3124.6816	28.2279	54.2600	0.6699	0.8023	
A	3124.5221	18.7371	88.2972	0.5481	0.7081	
A	3121.2342	21.2543	63.7572	0.7351	0.8473	
A	3116.1643	55.2597	112.1152	0.6815	0.8106	
A	3111.1848	3.3272	16.3025	0.7220	0.8386	
A	3109.8398	25.4756	60.9216	0.7330	0.8459	
A	3108.6747	5.1206	68.9930	0.2426	0.3904	
A	3107.8671	15.1110	30.1250	0.6556	0.7920	
A	3104.0143	12.6369	37.9796	0.7497	0.8570	
A	3097.0102	44.4113	156.8292	0.7500	0.8571	
A	3096.5697	16.2351	22.1910	0.4229	0.5944	
A	3096.1357	30.7490	138.1088	0.3395	0.5070	
A	3094.0450	0.7150	10.8160	0.7436	0.8530	
A	3071.4171	4.2839	56.2107	0.0754	0.1402	
A	3058.2560	26.6609	139.7871	0.0292	0.0568	
A	3056.4107	30.8817	83.6938	0.1416	0.2481	
A	3050.6703	18.2235	363.5049	0.0138	0.0273	
A	3045.7249	10.9167	60.6233	0.0460	0.0880	
A	3044.0098	21.3567	17.9672	0.1807	0.3061	
A	3042.1326	27.0068	16.5726	0.0760	0.1413	
A	3041.3218	38.9582	218.9798	0.0491	0.0936	
A	3032.4027	0.9600	82.2010	0.1648	0.2830	
A	3030.7076	42.3690	133.4183	0.0499	0.0951	
A	3022.4720	63.2515	219.0093	0.1748	0.2977	
A	2349.9040	5.6615	130.1194	0.1627	0.2799	

A	1787.3674	498.7214	12.9735	0.2643	0.4181
A	1785.5613	294.8213	11.2079	0.1760	0.2993
A	1767.3168	224.6782	3.7453	0.4126	0.5841
A	1673.1747	248.7107	10.5011	0.1417	0.2482
A	1574.4348	289.3440	1.0057	0.6899	0.8165
A	1560.4454	204.3408	3.9479	0.1238	0.2204
A	1537.5276	4.2614	13.6449	0.5791	0.7335
A	1530.2153	23.4294	0.9508	0.3911	0.5623
A	1521.3579	8.3348	3.5239	0.7325	0.8456
A	1518.6402	5.9544	6.8329	0.6962	0.8209
A	1516.7178	1.8224	8.9783	0.7496	0.8569
A	1510.3022	7.2719	13.6621	0.7475	0.8555
A	1508.3030	5.4758	1.3541	0.7384	0.8495
A	1503.4052	8.4565	8.2000	0.7139	0.8331
A	1500.7957	2.2006	8.1104	0.7425	0.8522
A	1496.4950	10.0555	0.2393	0.7500	0.8571
A	1495.1859	1.8742	3.4735	0.7092	0.8299
A	1491.7410	7.8006	11.3591	0.7343	0.8468
A	1489.5110	1.0479	10.3502	0.7500	0.8571
A	1484.6021	7.1553	3.1514	0.7500	0.8571
A	1478.2980	206.2042	5.3655	0.0301	0.0584
A	1453.1453	55.3245	1.4789	0.4931	0.6605
A	1452.0380	14.4728	15.6879	0.2255	0.3680
A	1442.8232	11.7205	0.3228	0.7405	0.8509
A	1431.0151	3.3439	5.2597	0.2344	0.3797
A	1418.0242	4.0711	2.1434	0.7496	0.8569
A	1412.4983	8.2648	2.2675	0.6556	0.7920
A	1411.0644	22.4757	1.1541	0.5839	0.7373
A	1409.9835	6.3403	2.5866	0.6379	0.7789
A	1393.8356	1.7291	3.7351	0.7450	0.8538
A	1385.1806	6.7603	5.6495	0.7036	0.8260
A	1377.0967	26.5612	9.6563	0.7468	0.8551
A	1375.9873	4.4399	2.2777	0.7013	0.8245
A	1367.7547	2.1108	4.0997	0.6660	0.7995
A	1353.1083	5.8754	2.6020	0.5964	0.7472
A	1352.8604	29.8118	3.1905	0.7345	0.8470
A	1339.3602	2.2410	0.9371	0.6921	0.8180
A	1338.0588	19.0543	4.0598	0.7499	0.8571
A	1322.2084	2.5267	4.9954	0.2320	0.3767
A	1311.6952	55.3899	9.0730	0.6753	0.8062
A	1309.7157	14.4578	11.0058	0.7495	0.8568
A	1296.5700	81.5364	7.3749	0.3443	0.5122
A	1286.3429	20.3408	2.7203	0.7428	0.8524
A	1270.6585	85.4920	3.0391	0.2964	0.4573
A	1258.2516	19.3910	7.9922	0.3879	0.5590
A	1253.3240	5.7580	10.2881	0.6358	0.7774
A	1244.7530	2.9238	4.5601	0.3778	0.5484
A	1239.0356	78.7788	1.7114	0.7225	0.8389
A	1229.3412	0.7796	9.4232	0.4378	0.6090
A	1225.4104	9.4738	2.7657	0.7176	0.8356
A	1220.5583	42.4383	4.4421	0.6154	0.7619

A	1209.9341	26.8060	3.1503	0.4700	0.6394
A	1209.2912	257.0372	3.4723	0.2106	0.3480
A	1204.6446	7.1805	2.7573	0.5921	0.7438
A	1188.9475	15.8330	1.3141	0.4207	0.5923
A	1179.5436	254.4913	1.9080	0.6888	0.8157
A	1157.3666	4.6210	4.2620	0.7472	0.8553
A	1152.9976	137.9270	1.8402	0.4147	0.5863
A	1151.5027	21.3744	6.9474	0.1869	0.3150
A	1139.0228	3.7367	3.7375	0.4403	0.6114
A	1133.8331	2.9944	6.6784	0.4487	0.6194
A	1116.2496	11.9282	1.0040	0.7178	0.8357
A	1114.9932	1.0260	2.8362	0.3221	0.4872
A	1093.5873	7.6966	4.2163	0.2953	0.4559
A	1078.8538	3.8641	4.5360	0.3444	0.5124
A	1071.4541	2.0870	1.4963	0.6672	0.8004
A	1065.2704	13.3122	1.5428	0.7379	0.8492
A	1054.7475	1.8119	4.8045	0.3871	0.5581
A	1046.6950	2.1365	2.0698	0.7123	0.8320
A	1043.6432	1.3157	0.9017	0.4817	0.6502
A	1030.4887	23.2257	1.7849	0.7485	0.8561
A	1021.8225	2.2435	4.7845	0.2296	0.3734
A	1010.5181	4.7674	1.6488	0.6196	0.7652
A	998.6484	5.1252	3.0161	0.6871	0.8145
A	976.9070	7.3036	6.8976	0.3174	0.4818
A	973.4931	2.8487	2.0325	0.7475	0.8555
A	971.4723	1.7298	2.4845	0.6827	0.8114
A	967.9111	0.3071	0.2735	0.3729	0.5432
A	954.2172	1.2471	3.2497	0.7469	0.8551
A	949.0797	6.4320	5.2357	0.2938	0.4541
A	946.5544	1.4940	3.2143	0.7413	0.8514
A	944.7067	3.5783	10.5688	0.3045	0.4668
A	928.3557	13.5556	10.3383	0.1574	0.2719
A	903.7493	4.4061	6.4810	0.3325	0.4991
A	900.8818	1.5337	5.1126	0.2514	0.4018
A	895.7928	14.9644	8.0124	0.2416	0.3892
A	851.4828	5.2452	1.7549	0.0378	0.0729
A	839.0580	1.9525	3.6074	0.1808	0.3063
A	828.3195	6.2944	4.4342	0.6210	0.7662
A	809.7668	1.4933	2.5401	0.2220	0.3634
A	797.0444	1.4604	0.4408	0.2695	0.4245
A	783.3342	3.6119	6.1435	0.1569	0.2713
A	769.5503	15.1965	1.3598	0.5869	0.7397
A	763.3959	7.9279	1.8860	0.1468	0.2561
A	748.1563	1.8466	1.7682	0.7459	0.8545
A	716.4283	23.6617	0.0920	0.1351	0.2381
A	711.2448	14.6232	1.6560	0.3984	0.5698
A	706.3231	10.6650	14.7409	0.0282	0.0548
A	695.4627	17.8196	4.1123	0.2481	0.3976
A	692.0493	2.4572	2.0584	0.5769	0.7317
A	679.0236	29.6027	4.0157	0.5148	0.6797
A	634.1345	18.7762	0.9033	0.6628	0.7972

A	622.2308	49.7210	0.9055	0.3033	0.4654
A	616.6989	101.4728	0.0962	0.5056	0.6716
A	603.5146	17.0420	2.4959	0.0986	0.1795
A	597.7430	24.7705	2.6235	0.6593	0.7946
A	574.2017	1.4274	0.4091	0.5578	0.7161
A	553.8649	8.8164	1.9719	0.1722	0.2938
A	539.7518	5.1751	1.9179	0.3851	0.5561
A	531.5800	85.9610	0.6958	0.4977	0.6646
A	504.9914	14.5247	0.5048	0.6524	0.7897
A	482.8420	14.0982	1.1504	0.5590	0.7171
A	475.4184	9.5094	1.9927	0.0475	0.0908
A	457.2774	8.6095	0.1157	0.1818	0.3076
A	450.6907	10.2704	0.5701	0.6831	0.8117
A	420.1490	5.4463	1.2304	0.2857	0.4444
A	416.4935	4.5604	0.6901	0.3573	0.5265
A	405.2233	4.1854	1.3065	0.7179	0.8358
A	394.1553	3.7818	0.3840	0.7234	0.8395
A	386.3267	7.5517	0.6752	0.7415	0.8516
A	366.6480	10.3051	0.3791	0.3763	0.5468
A	362.5177	2.7831	0.5828	0.6038	0.7530
A	353.8163	11.5671	0.5438	0.7453	0.8541
A	323.4364	0.9646	0.3232	0.7474	0.8554
A	318.4294	1.7866	0.5019	0.1909	0.3205
A	306.2881	8.7262	1.0322	0.5282	0.6913
A	299.1386	4.3849	0.7072	0.7500	0.8571
A	293.5781	3.4585	0.9558	0.4245	0.5960
A	284.7894	0.8065	0.5653	0.2543	0.4054
A	276.5217	1.1190	1.1691	0.7177	0.8357
A	272.9630	1.5450	0.7341	0.4752	0.6442
A	268.2163	7.7123	2.0134	0.7347	0.8471
A	253.6405	2.6127	1.5240	0.0866	0.1594
A	251.8411	7.3510	0.4863	0.6673	0.8005
A	242.0548	6.8564	1.5875	0.3576	0.5268
A	235.4736	5.1270	2.1214	0.2566	0.4084
A	230.6807	1.1089	0.7670	0.1917	0.3218
A	222.5871	0.6877	0.3476	0.1291	0.2286
A	199.9141	11.8019	1.0544	0.0742	0.1381
A	187.0057	0.6542	1.6707	0.3926	0.5638
A	183.2721	4.7603	1.1850	0.2780	0.4350
A	172.1434	5.1901	0.0911	0.4587	0.6289
A	168.4541	0.0902	1.2683	0.4861	0.6542
A	155.6376	0.2413	1.2962	0.4304	0.6018
A	144.9300	0.5006	0.2290	0.4441	0.6151
A	127.0782	1.3444	0.4214	0.5271	0.6903
A	107.8774	1.6827	0.2666	0.7460	0.8545
A	101.0713	1.2056	0.3449	0.3597	0.5291
A	87.0177	0.7390	0.3594	0.7464	0.8548
A	80.3411	4.4045	0.7342	0.6756	0.8064
A	70.5970	0.7321	0.2476	0.6826	0.8114
A	66.1293	4.5416	3.1389	0.7496	0.8569
A	57.3863	1.5768	0.6399	0.7497	0.8570

A	49.3634	0.5242	1.3648	0.7495	0.8568
A	45.7844	2.4525	1.0181	0.7448	0.8538
A	38.4754	2.3297	0.5558	0.7492	0.8566
A	29.5535	4.4369	0.2771	0.7450	0.8539
A	28.7958	0.8393	0.1547	0.7161	0.8346
A	19.4568	0.6785	0.2021	0.7476	0.8556
A	17.0812	0.3589	0.1127	0.6551	0.7916
A	13.7254	0.8998	0.2218	0.7499	0.8571
A	4.2747	1.9081	0.2027	0.7414	0.8515

67

C	2.237026	4.043649	-0.248934
C	0.748456	3.777959	-0.186179
C	0.245572	2.488603	0.419011
N	0.912387	1.418270	-0.362892
C	1.510011	1.932955	-1.616574
C	1.525204	3.436807	-1.442035
C	-1.290048	2.364269	0.275358
O	-1.956118	3.134660	-0.403775
C	1.047390	0.181314	0.155863
O	0.625051	-0.113007	1.289994
C	2.622587	5.506302	-0.402920
C	3.237971	3.252591	0.576211
N	-1.811724	1.304023	0.952537
C	-3.211221	0.931227	0.843722
C	-3.998489	1.498250	1.961224
N	-4.626291	1.898696	2.852042
C	-3.347382	-0.604909	0.820643
C	-4.781826	-1.095260	0.628188
C	-5.308348	-0.860413	-0.793946
N	-6.054416	-1.952082	-1.132444
C	-6.183222	-2.947731	-0.072647
C	-4.982089	-2.607453	0.833317
O	-5.099193	0.118606	-1.499730
C	1.723891	-0.903711	-0.682070
N	2.656557	-1.588654	0.204018
C	3.961528	-1.723956	-0.108669
O	4.496959	-1.386993	-1.153464
C	0.695443	-1.886333	-1.342646
C	-0.048030	-2.723867	-0.288740
C	-0.317781	-1.074621	-2.168601
C	1.478763	-2.824193	-2.277686
C	4.817363	-2.408908	0.988021
F	4.115178	-2.637732	2.130969
F	5.271353	-3.597561	0.548535
F	5.873525	-1.643557	1.306718
H	1.890547	6.054091	-1.004604
H	3.599662	5.599757	-0.890993
H	2.685717	5.994843	0.576162
H	2.960682	2.206792	0.717368
H	4.223582	3.274086	0.097534

H	3.342912	3.702095	1.570069
H	1.409950	4.032017	-2.342094
H	0.053883	4.609127	-0.176265
H	0.520737	2.355221	1.470788
H	2.509559	1.511228	-1.752539
H	0.892202	1.658866	-2.477515
H	2.324419	-0.473973	-1.482358
H	2.335579	-1.787959	1.143676
H	-1.017054	-1.752413	-2.667470
H	-0.905116	-0.390053	-1.549473
H	0.180184	-0.488109	-2.947589
H	0.790177	-3.518086	-2.770745
H	2.011833	-2.263275	-3.051846
H	2.213654	-3.417260	-1.726108
H	-0.763822	-3.383939	-0.789992
H	0.642499	-3.358122	0.275893
H	-0.590653	-2.098144	0.422194
H	-1.166807	0.698199	1.456032
H	-3.608202	1.348217	-0.087235
H	-2.945015	-1.012357	1.756027
H	-2.719655	-0.974236	0.003860
H	-4.094485	-3.146884	0.482627
H	-5.155186	-2.879527	1.876881
H	-7.135174	-2.836836	0.464345
H	-6.132936	-3.962579	-0.476706
H	-6.609259	-1.950246	-1.976933
H	-5.450712	-0.544247	1.304516

Conformer **A**: B3LYPD3BJ/6-31+G(d,p), in acetonitrile solvent

Processing: nirc6pbed3jacs.log
PG=C01

Method	BasisSet	Imaginary Freqs
RB3LYP	6-31+G(d,p)	0

HF Energy
-1770.2430739

ZPE	E298	S298	Squasihar	Equasihar	Strans	Srot
343.47426	366.063	228.538	204.272	366.175	44.511	37.619

Label	Frequencies	IR Inten	Raman Activ	Depolar (P)	Depolar (U)	Dipole
A	3608.2390	102.7433	338.8821	0.2094	0.3463	8.6917
A	3597.6563	172.6363	134.6635	0.0893	0.1639	
A	3467.9703	352.2808	236.1841	0.1197	0.2138	
A	3211.7936	7.2831	358.6476	0.1642	0.2820	
A	3185.8356	21.7545	368.9694	0.2935	0.4538	
A	3158.7204	3.3304	159.9854	0.1147	0.2058	
A	3148.2374	33.2491	176.2491	0.5123	0.6776	

A	3147.2053	30.9989	206.3283	0.6051	0.7540
A	3132.4765	41.3691	236.9104	0.5768	0.7316
A	3127.1589	14.5195	301.2001	0.6720	0.8038
A	3125.2493	24.0514	66.6015	0.4494	0.6201
A	3119.3770	29.3552	155.2865	0.5280	0.6911
A	3115.7919	57.4565	196.0059	0.7401	0.8506
A	3113.7965	95.6434	396.6016	0.7441	0.8532
A	3113.1853	38.7284	312.0659	0.3407	0.5083
A	3108.9109	25.9317	117.1408	0.7404	0.8508
A	3107.5678	53.6839	101.3265	0.2541	0.4053
A	3105.8150	50.8242	193.1935	0.7490	0.8565
A	3104.4661	24.3951	103.4213	0.7342	0.8467
A	3088.1248	101.5018	460.2932	0.7497	0.8569
A	3086.3196	8.3265	48.9354	0.7313	0.8448
A	3081.9629	4.9749	361.3265	0.1023	0.1856
A	3076.6704	55.9246	336.4386	0.0430	0.0825
A	3065.8106	68.2980	643.4450	0.0541	0.1026
A	3062.7927	3.0052	49.4552	0.3424	0.5102
A	3049.1177	23.5142	1128.2234	0.0084	0.0166
A	3043.1582	57.2419	15.4027	0.7432	0.8527
A	3039.8755	78.8328	369.6961	0.1804	0.3056
A	3038.1885	47.2395	100.7932	0.0101	0.0200
A	3036.3170	81.9550	646.0782	0.0319	0.0618
A	3033.0077	14.6824	455.7181	0.1638	0.2814
A	3025.2863	60.7134	299.2475	0.0086	0.0171
A	2342.3702	52.8186	373.5780	0.1543	0.2674
A	1753.5757	643.0136	30.7963	0.1941	0.3252
A	1721.3789	589.6002	17.2046	0.3462	0.5144
A	1719.3796	943.1179	38.7717	0.3048	0.4672
A	1649.4829	552.4249	39.2681	0.2277	0.3709
A	1578.6681	242.3510	18.7782	0.0966	0.1761
A	1564.1896	574.4006	5.9149	0.5741	0.7294
A	1517.4817	13.6902	22.9332	0.7116	0.8315
A	1508.2752	32.8359	3.4722	0.5162	0.6809
A	1503.6488	4.1785	25.9020	0.5320	0.6945
A	1502.1613	11.3432	12.5912	0.6309	0.7736
A	1497.7786	5.7793	20.8790	0.7490	0.8565
A	1494.7210	8.9313	37.0907	0.7427	0.8524
A	1487.2346	12.4539	2.0917	0.7456	0.8543
A	1487.0012	1.2572	24.0466	0.7495	0.8568
A	1486.3760	28.9559	14.1819	0.7495	0.8568
A	1485.9707	0.6340	19.2805	0.7486	0.8562
A	1482.6332	38.4243	1.3833	0.2762	0.4329
A	1478.1988	11.4913	29.5662	0.7045	0.8267
A	1474.4459	23.0931	7.7171	0.7445	0.8535
A	1473.7381	0.8883	32.3983	0.7498	0.8570
A	1472.3041	295.7438	12.5674	0.0320	0.0620
A	1455.6329	74.7555	15.9906	0.4972	0.6641
A	1442.5473	21.5310	56.9172	0.2213	0.3624
A	1427.1990	7.8555	2.9576	0.0636	0.1195
A	1421.0188	3.6703	8.5878	0.4837	0.6520

A	1412.2635	5.1376	3.2053	0.4767	0.6456
A	1407.3647	16.8454	1.5765	0.7465	0.8548
A	1402.0500	11.6102	1.0795	0.1284	0.2276
A	1395.5591	24.9106	22.3484	0.6802	0.8097
A	1383.3149	2.6279	13.6763	0.7474	0.8554
A	1379.4714	6.4639	8.8101	0.4744	0.6436
A	1376.3682	62.4638	34.1973	0.7426	0.8523
A	1370.1865	3.8847	1.0538	0.6236	0.7682
A	1363.4753	7.4172	7.7650	0.6356	0.7772
A	1343.6824	17.3168	7.3350	0.5296	0.6925
A	1342.1360	41.5737	11.0790	0.7399	0.8505
A	1333.1734	1.3184	2.8585	0.6541	0.7909
A	1325.8536	50.0875	14.3132	0.5185	0.6829
A	1315.3906	2.0081	9.8988	0.3032	0.4653
A	1312.0125	26.9501	18.5722	0.1571	0.2716
A	1302.1847	81.5267	51.0083	0.7097	0.8302
A	1300.4440	64.8201	17.8190	0.6376	0.7787
A	1281.1810	35.6423	11.3016	0.3710	0.5412
A	1277.8120	77.4339	5.3546	0.6475	0.7860
A	1252.0082	5.2783	27.5451	0.5030	0.6694
A	1247.2502	69.5962	30.7426	0.7443	0.8534
A	1245.1827	16.4807	36.1028	0.3512	0.5198
A	1238.4356	62.7298	20.9841	0.5265	0.6898
A	1227.1398	10.3213	22.6736	0.4815	0.6500
A	1219.9926	7.6148	6.2175	0.7419	0.8518
A	1211.2621	16.1128	16.1469	0.5765	0.7314
A	1206.8933	2.7230	11.6312	0.5167	0.6813
A	1200.5962	15.5322	6.0447	0.5525	0.7117
A	1199.1880	361.5101	5.6673	0.3434	0.5113
A	1185.0306	30.5081	6.5982	0.3297	0.4959
A	1151.5038	17.1419	16.7287	0.7460	0.8546
A	1145.3351	7.7113	10.0709	0.4160	0.5875
A	1144.0170	379.5049	4.0657	0.2916	0.4516
A	1133.2403	1.4705	8.9058	0.6066	0.7551
A	1127.1056	2.6362	25.5125	0.3099	0.4732
A	1114.6913	429.0578	4.6309	0.7500	0.8571
A	1108.4151	4.9369	14.2457	0.3379	0.5051
A	1103.7156	79.7050	4.1019	0.7478	0.8557
A	1091.3043	14.1168	10.4463	0.3907	0.5619
A	1070.5718	7.2806	10.7718	0.2203	0.3610
A	1065.1665	7.3811	4.9168	0.2679	0.4226
A	1061.8632	20.9252	6.8838	0.6265	0.7703
A	1052.9703	5.4541	12.9405	0.4639	0.6338
A	1042.2956	6.0911	7.9239	0.7180	0.8359
A	1040.8711	2.3688	2.6711	0.4825	0.6509
A	1029.2502	33.9195	6.1285	0.7149	0.8338
A	1021.6003	4.3129	11.0590	0.2421	0.3898
A	1005.5213	7.6641	4.4976	0.6313	0.7740
A	993.0036	9.2839	11.0300	0.6197	0.7652
A	979.1749	15.1971	17.4155	0.1979	0.3303
A	975.1132	4.1902	4.3672	0.7315	0.8449

A	970.4542	2.1646	5.2869	0.7004	0.8238
A	969.7879	3.0720	8.8031	0.5930	0.7445
A	953.4647	0.5698	9.6361	0.6295	0.7727
A	950.1157	9.5513	12.2918	0.4115	0.5831
A	947.0368	1.9642	10.1853	0.5268	0.6901
A	946.5203	11.3386	20.0433	0.4496	0.6203
A	924.5647	19.0964	25.4064	0.1878	0.3162
A	900.1489	6.0314	27.7041	0.2557	0.4073
A	895.6120	8.4110	8.6712	0.4405	0.6116
A	888.5864	29.7099	12.4808	0.2716	0.4272
A	845.7620	4.3091	5.6019	0.0627	0.1181
A	840.6053	2.6330	7.1028	0.3347	0.5016
A	825.0090	18.1773	10.7199	0.7488	0.8564
A	807.9568	1.3721	8.6427	0.1381	0.2427
A	794.0276	5.4999	0.7359	0.5491	0.7089
A	785.2848	8.1202	13.5051	0.1268	0.2251
A	766.3461	30.6925	4.2774	0.4490	0.6198
A	758.9709	16.9039	7.0656	0.2709	0.4263
A	741.0412	4.3184	4.8114	0.7499	0.8571
A	713.8593	45.7984	5.1825	0.2000	0.3333
A	711.1647	21.0648	1.5983	0.1808	0.3062
A	702.3757	58.9299	22.5055	0.0916	0.1679
A	696.5509	27.7900	13.0885	0.1349	0.2378
A	695.0920	38.9136	2.6104	0.3065	0.4692
A	682.4780	43.7246	14.0922	0.5739	0.7293
A	638.7927	53.8148	1.5554	0.7290	0.8433
A	624.5740	34.0324	2.1437	0.4102	0.5818
A	603.1477	36.0666	4.3416	0.1263	0.2243
A	598.6846	32.8537	7.2227	0.3711	0.5414
A	572.8998	13.8586	1.7651	0.7206	0.8376
A	563.6044	156.5546	1.3376	0.3667	0.5366
A	550.8198	11.1866	5.7595	0.2235	0.3654
A	534.9457	17.7577	3.8942	0.3674	0.5374
A	526.0433	172.8621	1.2732	0.6878	0.8150
A	500.3098	67.0705	0.9498	0.7011	0.8243
A	481.4692	17.6600	1.7862	0.6484	0.7867
A	473.5905	21.7162	4.6001	0.1695	0.2898
A	457.7726	11.1220	0.3659	0.2189	0.3592
A	450.6154	19.3557	1.1428	0.7472	0.8553
A	423.1221	10.5153	1.3892	0.6021	0.7516
A	415.5018	10.0486	1.5037	0.5032	0.6695
A	405.9033	5.4591	2.4813	0.5329	0.6953
A	396.6023	4.1201	0.9133	0.6845	0.8127
A	384.5134	8.3185	0.9800	0.6011	0.7509
A	371.6766	27.9303	0.8946	0.5091	0.6747
A	364.1949	2.6549	0.8230	0.6870	0.8145
A	359.7493	23.7485	1.6552	0.7440	0.8532
A	332.9758	1.4275	0.5162	0.6403	0.7807
A	326.1880	3.5246	0.4748	0.4348	0.6060
A	309.2977	16.3698	4.0258	0.4830	0.6514
A	306.9487	6.2593	0.6587	0.7256	0.8410

A	295.8314	8.6039	0.9695	0.4140	0.5856
A	289.7471	4.8669	1.8179	0.3858	0.5568
A	285.9026	1.7642	1.3456	0.6479	0.7864
A	280.3608	1.1756	1.8619	0.4183	0.5899
A	270.1968	8.6374	3.5778	0.7247	0.8404
A	257.5655	9.3364	0.8771	0.0925	0.1693
A	253.4745	4.0172	2.2054	0.2024	0.3367
A	242.4483	13.0286	3.9994	0.3792	0.5499
A	239.1170	4.1005	2.0892	0.2989	0.4603
A	235.1114	14.0791	3.1577	0.4253	0.5968
A	222.7565	3.2819	0.3735	0.2996	0.4610
A	202.1939	20.0537	2.6579	0.1460	0.2548
A	191.2789	12.0092	3.5363	0.7403	0.8508
A	186.7948	2.4964	1.8157	0.1281	0.2270
A	176.5153	4.6165	2.0707	0.3988	0.5702
A	171.4773	5.6772	1.7937	0.7154	0.8341
A	155.9463	0.6448	1.5150	0.5010	0.6676
A	144.9711	1.0730	0.2771	0.4427	0.6137
A	119.8576	2.7120	0.4933	0.7282	0.8427
A	108.9612	4.3033	0.3161	0.6327	0.7751
A	103.2652	0.8760	0.5873	0.4803	0.6489
A	91.3575	0.3689	0.1505	0.7213	0.8381
A	84.6907	0.9682	0.2399	0.7109	0.8311
A	82.9638	5.6996	1.7515	0.7495	0.8568
A	69.3861	0.5461	3.8104	0.7481	0.8559
A	62.0263	18.2711	3.3251	0.7445	0.8535
A	52.6990	0.0349	2.3361	0.7495	0.8568
A	43.7042	0.9682	1.9809	0.7498	0.8570
A	38.7767	7.1142	1.5617	0.7496	0.8569
A	33.1296	5.9296	0.4764	0.7467	0.8550
A	32.3017	0.8610	0.4878	0.7496	0.8569
A	27.4952	3.6609	0.0514	0.6766	0.8071
A	23.4447	4.9301	0.7439	0.7488	0.8564
A	22.0095	0.1703	0.4976	0.7497	0.8570
A	12.0914	4.4460	0.2532	0.7498	0.8570

67

C	2.000650	4.114019	-0.366438
C	0.531366	3.783924	-0.203075
C	0.127991	2.477853	0.440558
N	0.798435	1.433319	-0.372243
C	1.280583	1.964423	-1.670356
C	1.239297	3.468727	-1.506885
C	-1.402955	2.270215	0.404240
O	-2.162797	2.992005	-0.243333
C	1.057843	0.224007	0.161932
O	0.716417	-0.071545	1.325340
C	2.305050	5.592194	-0.548013
C	3.090547	3.376884	0.392534
N	-1.819455	1.209926	1.142297
C	-3.188037	0.726324	1.105095

C	-3.817122	0.908398	2.429512
N	-4.310996	1.039351	3.472545
C	-3.244061	-0.746291	0.651376
C	-4.653929	-1.319627	0.515735
C	-5.479621	-0.688348	-0.610039
N	-6.162559	-1.680721	-1.224604
C	-5.986429	-3.001711	-0.619915
C	-4.679946	-2.821071	0.173512
O	-5.529146	0.513332	-0.902216
C	1.775525	-0.828186	-0.687971
N	2.750714	-1.470820	0.186766
C	4.050319	-1.557750	-0.113286
O	4.612867	-1.158987	-1.129861
C	0.794885	-1.870799	-1.332092
C	0.048945	-2.689903	-0.267024
C	-0.218324	-1.127902	-2.218388
C	1.628821	-2.818629	-2.210672
C	4.918169	-2.269701	0.955173
F	4.235292	-2.617140	2.071991
F	5.452830	-3.399871	0.441312
F	5.939980	-1.474535	1.336094
H	1.508099	6.101825	-1.099452
H	3.241758	5.727954	-1.102410
H	2.414747	6.087554	0.424503
H	2.889466	2.313359	0.530150
H	4.044467	3.468531	-0.140659
H	3.222689	3.822039	1.385958
H	1.044092	4.051519	-2.401497
H	-0.184437	4.594562	-0.139334
H	0.470790	2.374175	1.474868
H	2.285190	1.587597	-1.873815
H	0.617299	1.652059	-2.481281
H	2.338405	-0.365885	-1.496096
H	2.421175	-1.792152	1.090041
H	-0.887066	-1.853556	-2.692573
H	-0.837367	-0.432600	-1.643461
H	0.286277	-0.566860	-3.011435
H	0.967400	-3.539857	-2.702344
H	2.169242	-2.267085	-2.987391
H	2.355632	-3.382894	-1.617895
H	-0.605383	-3.413769	-0.764453
H	0.740875	-3.252946	0.368845
H	-0.568808	-2.058622	0.374245
H	-1.101987	0.620516	1.565510
H	-3.747602	1.344076	0.397519
H	-2.673149	-1.356416	1.359071
H	-2.731153	-0.796130	-0.313648
H	-3.824200	-3.062714	-0.466627
H	-4.638765	-3.458458	1.058995
H	-6.831742	-3.240180	0.037686
H	-5.914615	-3.773890	-1.389455

H -6.874712 -1.480078 -1.916854
H -5.218649 -1.145862 1.442854

Conformer **B**: B3LYPD3BJ/6-31+G(d,p)

Processing: nira6pbed3j.log
PG=C01

Method	BasisSet	Imaginary Freqs
RB3LYP	6-31+G(d,p)	0

HF Energy
-1770.1930682

ZPE	E298	S298	Squasihar	Equasihar	Strans	Srot
344.47285	367.129	231.448	204.582	367.245	44.511	37.620

Label	Frequencies	IR Inten	Raman Activ	Depolar (P)	Depolar (U)	Dipole
A	3642.4320	45.4150	187.9987	0.2412	0.3886	6.1505
A	3609.2795	69.2329	41.1391	0.0772	0.1434	
A	3498.4335	173.9302	83.4660	0.1447	0.2529	
A	3212.4237	2.1407	94.9015	0.1440	0.2518	
A	3185.0530	11.2801	111.4801	0.2838	0.4422	
A	3143.2791	22.3185	56.8298	0.5157	0.6805	
A	3142.4860	5.8529	32.1708	0.0790	0.1465	
A	3140.6909	26.9617	69.4090	0.6434	0.7830	
A	3127.1392	22.9071	61.5645	0.6858	0.8136	
A	3124.1064	20.9664	48.2547	0.7314	0.8448	
A	3121.8390	21.8928	68.5929	0.5145	0.6794	
A	3116.1855	54.3897	118.7486	0.7191	0.8366	
A	3111.8475	0.5726	27.2446	0.1789	0.3035	
A	3110.3284	5.4930	22.7194	0.7165	0.8348	
A	3109.6420	24.8615	26.1575	0.6135	0.7605	
A	3109.4748	10.8644	53.4313	0.7493	0.8567	
A	3103.2880	16.2000	43.4059	0.7424	0.8521	
A	3100.7750	17.2553	43.8244	0.3109	0.4743	
A	3097.4937	40.5941	153.1837	0.7500	0.8571	
A	3094.5207	1.3753	13.0330	0.7440	0.8532	
A	3094.0504	32.2915	139.4600	0.3254	0.4910	
A	3076.8077	4.0764	51.4203	0.0857	0.1578	
A	3059.0813	32.6070	166.1668	0.0193	0.0379	
A	3057.5099	36.9417	112.9532	0.1515	0.2632	
A	3052.1079	10.1632	240.3239	0.0283	0.0550	
A	3051.0136	15.6471	149.3954	0.0412	0.0791	
A	3044.3742	19.6042	26.6199	0.1152	0.2065	
A	3042.0325	38.2446	45.7514	0.0506	0.0963	
A	3041.7208	28.0873	185.1515	0.0509	0.0969	
A	3031.2185	1.4602	70.5764	0.1362	0.2398	
A	3030.8588	42.4917	132.2609	0.0512	0.0974	
A	3019.7436	63.7351	197.1660	0.1741	0.2966	

A	2356.9569	7.7530	133.7112	0.1640	0.2817
A	1789.1248	428.0465	11.9290	0.2449	0.3934
A	1784.8522	305.6811	10.7641	0.1944	0.3256
A	1758.6699	255.0655	3.6929	0.3734	0.5438
A	1675.8933	227.5704	9.5488	0.1649	0.2831
A	1571.7242	279.5325	1.9427	0.7021	0.8250
A	1559.5843	210.9523	4.0296	0.1279	0.2267
A	1538.0690	4.3888	12.6459	0.5937	0.7450
A	1529.2781	24.2760	1.3608	0.4586	0.6288
A	1521.8958	7.5952	2.8801	0.7322	0.8454
A	1518.9212	3.4868	9.0486	0.7156	0.8342
A	1516.4934	1.0447	9.2466	0.7500	0.8571
A	1509.6825	4.3380	14.1075	0.7444	0.8535
A	1508.1698	5.7170	1.2416	0.7408	0.8511
A	1503.1229	17.5286	8.9740	0.7382	0.8494
A	1498.1395	3.3947	6.0305	0.7263	0.8415
A	1496.6840	8.9616	0.3373	0.7497	0.8569
A	1496.2066	2.3543	7.7490	0.7489	0.8564
A	1489.4799	1.2102	9.3685	0.7479	0.8557
A	1489.0977	2.6382	4.9944	0.6208	0.7660
A	1484.4546	1.6348	5.8565	0.7500	0.8571
A	1476.1339	215.8944	4.5911	0.0323	0.0625
A	1453.6328	54.5821	1.4293	0.4488	0.6195
A	1451.9299	19.5397	15.7600	0.2170	0.3566
A	1443.3782	10.8153	0.3883	0.7361	0.8480
A	1431.2185	3.3263	5.3631	0.2330	0.3779
A	1418.2495	3.8890	2.1242	0.7491	0.8565
A	1412.1494	14.7265	0.6746	0.6970	0.8214
A	1411.2685	3.1654	0.4157	0.7396	0.8503
A	1397.5967	11.0292	7.4804	0.6452	0.7843
A	1386.7325	2.3197	5.7656	0.6100	0.7578
A	1381.0619	5.3420	2.4764	0.5562	0.7148
A	1376.8358	26.4463	11.4785	0.7466	0.8549
A	1367.5978	1.9822	3.7625	0.6144	0.7612
A	1363.9979	1.9714	1.9467	0.1268	0.2250
A	1354.1637	16.9427	2.7283	0.6586	0.7941
A	1350.3973	14.7113	1.7815	0.7384	0.8495
A	1337.6176	23.0234	5.0958	0.7469	0.8551
A	1333.8320	2.4692	1.2681	0.4342	0.6055
A	1324.2644	30.0817	5.3016	0.5241	0.6878
A	1321.5605	16.0982	12.0712	0.5685	0.7249
A	1311.4587	26.3200	2.7696	0.6225	0.7673
A	1295.4186	77.2482	7.4982	0.3666	0.5365
A	1290.5195	6.1946	1.9281	0.7467	0.8550
A	1271.3602	111.2670	3.0078	0.4171	0.5887
A	1258.1202	17.9211	8.4851	0.3514	0.5200
A	1252.9869	3.0865	9.8765	0.6817	0.8107
A	1244.0153	2.5084	5.0755	0.3616	0.5311
A	1238.2121	66.2266	2.1694	0.7242	0.8400
A	1230.5399	0.7354	8.6659	0.4583	0.6285
A	1225.4993	9.5734	2.8116	0.6869	0.8144

A	1219.8469	36.3779	4.2478	0.5916	0.7434
A	1211.3972	1.9150	4.1581	0.5276	0.6908
A	1208.9607	290.9547	2.6394	0.3366	0.5037
A	1204.9112	2.0909	3.4907	0.5396	0.7010
A	1190.4871	15.3625	1.1884	0.4208	0.5923
A	1179.1777	254.4217	1.8713	0.6954	0.8203
A	1157.0978	5.8957	4.2006	0.7474	0.8554
A	1154.6501	144.7656	2.6450	0.3457	0.5138
A	1146.6580	6.7287	3.4404	0.4700	0.6395
A	1135.3270	10.1957	2.9273	0.2965	0.4574
A	1124.0399	1.2213	6.4007	0.3099	0.4732
A	1116.3224	9.5227	3.0956	0.5341	0.6963
A	1116.1314	4.6382	3.2439	0.5507	0.7103
A	1097.5757	12.6163	4.2691	0.4812	0.6498
A	1075.1972	13.0477	3.6902	0.4776	0.6465
A	1071.6700	4.1852	2.2359	0.7274	0.8422
A	1058.4989	3.6118	1.0124	0.1524	0.2645
A	1054.7790	2.6731	6.5117	0.3499	0.5184
A	1050.1800	5.7638	1.5790	0.6766	0.8071
A	1047.4151	2.8122	1.9598	0.6997	0.8233
A	1041.4656	3.5337	1.6657	0.4076	0.5792
A	1021.8144	3.2840	5.4525	0.2199	0.3605
A	1009.5279	2.4603	2.0517	0.4776	0.6465
A	997.8972	4.3543	3.1417	0.6366	0.7780
A	984.9156	8.8794	6.9640	0.0833	0.1537
A	973.6265	1.1299	2.8961	0.7481	0.8559
A	973.2224	4.7371	3.7945	0.7441	0.8533
A	968.5029	0.4037	0.1568	0.4503	0.6209
A	952.6304	6.0771	5.0296	0.6864	0.8140
A	949.4176	6.5190	5.0856	0.2957	0.4564
A	946.1110	2.5428	7.6243	0.2352	0.3809
A	945.5419	0.8441	4.8307	0.7010	0.8242
A	928.9129	13.7530	9.6236	0.1392	0.2444
A	904.2485	2.4726	8.1819	0.2932	0.4535
A	900.4155	2.6757	4.6009	0.3254	0.4910
A	896.0323	15.1412	7.1188	0.1869	0.3149
A	853.0060	5.8913	2.7653	0.2001	0.3334
A	848.0473	1.9469	1.0034	0.7444	0.8535
A	828.6556	6.0030	4.2298	0.7372	0.8487
A	808.8099	1.5657	2.8581	0.1940	0.3250
A	793.3246	2.5775	0.4308	0.6960	0.8208
A	790.7375	5.6130	10.6687	0.1348	0.2376
A	769.4853	11.8849	1.3433	0.5331	0.6955
A	763.1614	9.8657	2.7117	0.1546	0.2677
A	741.5286	4.5110	1.3274	0.6355	0.7771
A	716.7222	27.5667	0.3858	0.0682	0.1277
A	711.5436	10.2895	11.2775	0.0504	0.0959
A	709.9674	30.7473	0.8680	0.4614	0.6314
A	703.1262	28.0752	3.7258	0.0572	0.1081
A	694.2823	8.5788	2.4426	0.6011	0.7509
A	683.0298	20.8669	6.8351	0.4225	0.5941

A	644.7399	48.5762	1.6944	0.4408	0.6119
A	627.2835	6.9189	1.5090	0.3505	0.5191
A	618.9234	95.9524	0.0973	0.4013	0.5728
A	604.6523	20.8222	2.2464	0.0891	0.1635
A	589.2542	6.1036	1.1383	0.4359	0.6072
A	566.1752	8.1124	0.3972	0.7497	0.8570
A	549.1435	11.1618	2.9729	0.2796	0.4370
A	540.2585	5.1496	2.1383	0.2352	0.3808
A	534.4332	89.2179	0.7488	0.6213	0.7664
A	504.8520	14.0242	0.4804	0.7359	0.8479
A	483.0009	14.1276	1.3439	0.6342	0.7762
A	473.1401	6.5369	2.3099	0.0744	0.1385
A	457.1631	6.1597	0.1514	0.2843	0.4428
A	451.1423	10.0244	0.3768	0.7351	0.8473
A	419.9134	5.1221	1.5317	0.3907	0.5619
A	416.8793	5.5261	0.5969	0.3750	0.5454
A	405.2944	4.2547	1.2721	0.7083	0.8292
A	396.2010	2.2227	0.1234	0.7015	0.8246
A	381.2422	3.1786	0.6744	0.6955	0.8204
A	366.5253	22.9581	0.6975	0.6788	0.8086
A	360.2978	0.5048	0.6298	0.7435	0.8529
A	355.5107	12.7059	0.9155	0.7114	0.8314
A	325.8255	0.6543	0.4498	0.7452	0.8540
A	315.6268	2.1651	0.4305	0.2534	0.4043
A	305.0464	6.8549	0.7425	0.3345	0.5014
A	300.0171	2.0555	1.0420	0.7202	0.8373
A	291.3703	9.2153	0.6590	0.1724	0.2941
A	288.2593	0.7406	1.0203	0.3491	0.5176
A	285.4291	4.8720	1.1393	0.6574	0.7933
A	274.2882	1.1691	0.7956	0.3791	0.5497
A	263.6134	2.7261	0.9468	0.6248	0.7690
A	253.6399	2.4133	0.4618	0.6998	0.8234
A	250.7208	1.3557	2.1284	0.0865	0.1591
A	240.8683	7.8467	3.0155	0.3104	0.4738
A	239.1022	9.1994	1.1026	0.5989	0.7492
A	227.8758	0.6317	0.5135	0.3954	0.5667
A	221.6715	0.4973	0.3940	0.2249	0.3672
A	206.6083	11.0200	1.6800	0.2110	0.3484
A	187.2000	4.8353	1.1817	0.3613	0.5308
A	185.7739	2.2306	1.2993	0.4650	0.6349
A	173.5308	1.3151	1.4847	0.4476	0.6184
A	170.2139	6.2016	0.4056	0.5810	0.7350
A	160.5932	1.6082	0.5806	0.5726	0.7282
A	145.6859	0.3885	0.2533	0.6343	0.7763
A	115.6425	5.1179	0.6780	0.7412	0.8514
A	109.0472	1.2446	0.2203	0.5677	0.7242
A	101.7754	0.1474	0.2752	0.2452	0.3939
A	91.0015	0.0551	0.5369	0.7295	0.8436
A	79.1993	4.2701	0.9544	0.6840	0.8123
A	73.1015	3.2802	1.4023	0.7360	0.8480
A	68.3849	4.4945	0.4287	0.6511	0.7887

A	62.6358	0.0830	0.1068	0.5719	0.7277
A	49.9598	1.1645	1.5005	0.7367	0.8484
A	44.5180	1.7104	1.6338	0.7446	0.8536
A	39.3917	2.2410	0.3130	0.7294	0.8436
A	33.2960	0.6184	0.6163	0.7500	0.8571
A	29.6276	1.4191	0.1563	0.6216	0.7667
A	22.9259	0.6853	0.2753	0.6230	0.7677
A	19.3345	0.7167	0.0335	0.7060	0.8277
A	13.4251	1.0939	0.1588	0.7484	0.8561
A	10.4908	2.0036	0.4338	0.7376	0.8490

67

C	-5.858566	-0.406163	-0.092432
C	-4.683533	-1.350740	0.195117
C	-4.822472	-2.436009	-0.888477
C	-6.328591	-2.442844	-1.222371
N	-6.710565	-1.065067	-0.932084
C	-3.321048	-0.658179	0.218968
C	-3.151009	0.400453	1.334792
C	-3.425078	-0.181531	2.657742
N	-3.640196	-0.692178	3.677744
O	-6.019210	0.721688	0.356076
N	-1.808267	0.968304	1.334987
C	-1.483143	1.978701	0.483041
O	-2.293151	2.549325	-0.239385
C	0.021211	2.331609	0.488665
C	0.276325	3.664712	-0.174999
C	0.991378	3.410272	-1.486652
C	1.180082	1.916212	-1.642848
N	0.761524	1.348975	-0.341158
C	1.700746	4.140903	-0.363696
C	1.856665	5.639761	-0.565341
C	2.868033	3.520271	0.385081
C	1.111532	0.155606	0.182486
C	1.888240	-0.839360	-0.681460
C	0.977571	-1.964984	-1.283671
C	-0.129534	-1.313647	-2.130425
O	0.815340	-0.166661	1.347190
N	2.945143	-1.375638	0.166819
C	4.238351	-1.351696	-0.215109
C	5.232754	-1.887492	0.847024
F	5.820815	-3.015084	0.405726
C	1.855531	-2.845487	-2.190172
C	0.345459	-2.834687	-0.184843
O	4.669416	-0.979137	-1.295655
F	6.190559	-0.978973	1.093861
F	4.626740	-2.178469	2.029374
H	1.007094	6.062978	-1.110485
H	2.767464	5.861179	-1.133656
H	1.927007	6.154655	0.399625
H	2.759093	2.448130	0.555931

H	3.799334	3.675553	-0.171244
H	2.983380	3.999212	1.363876
H	0.718730	3.962083	-2.380444
H	-0.528136	4.388111	-0.117779
H	0.407778	2.264375	1.510708
H	2.216337	1.638196	-1.854201
H	0.543638	1.538465	-2.448989
H	2.387219	-0.343176	-1.513061
H	2.700451	-1.586298	1.127069
H	-0.758944	-2.090570	-2.575526
H	-0.776482	-0.664260	-1.533827
H	0.291416	-0.722431	-2.950336
H	1.242660	-3.622105	-2.659178
H	2.329870	-2.257411	-2.982059
H	2.645910	-3.342353	-1.621061
H	-0.269594	-3.613777	-0.647197
H	1.109757	-3.334753	0.417789
H	-0.283736	-2.250738	0.489902
H	-1.039938	0.440040	1.745250
H	-3.869420	1.206318	1.158040
H	-3.150078	-0.139178	-0.729728
H	-2.536504	-1.414255	0.333927
H	-4.252689	-2.141265	-1.777192
H	-4.463563	-3.415459	-0.564003
H	-6.877659	-3.150585	-0.586056
H	-6.526772	-2.696223	-2.267583
H	-7.622092	-0.678897	-1.134963
H	-4.882616	-1.789404	1.18330

Conformer **B**: B3LYPD3BJ/6-31+G(d,p), in acetonitrile solvent

Processing: nira6pbed3jacs.log
PG=C01

Method	BasisSet	Imaginary Freqs
RB3LYP	6-31+G(d,p)	0

HF Energy
-1770.2430254

ZPE	E298	S298	Squasihar	Equasihar	Strans	Srot
343.54147	366.128	229.251	204.203	366.242	44.511	37.603

ccl00:/aue/chem126/aue/ark/nir> gtfr nira6pbed3jacs.log
Processing: nira6pbed3jacs.log
Population analysis using the SCF Density.

Label	Frequencies	IR Inten	Raman Activ	Depolar (P)	Depolar (U)	Dipole
A	3616.4407	98.5069	335.2336	0.2103	0.3475	9.2206
A	3596.9768	174.0960	136.4778	0.0911	0.1670	
A	3462.7896	358.6931	232.9275	0.1220	0.2175	
A	3210.9014	7.6725	361.6720	0.1629	0.2802	

A	3185.8944	21.7109	367.5785	0.2972	0.4582
A	3159.3906	2.9814	163.4029	0.1131	0.2033
A	3148.8063	33.5493	176.0512	0.5097	0.6753
A	3144.0950	32.5505	197.7083	0.6165	0.7627
A	3132.4419	41.7786	232.6747	0.5600	0.7180
A	3128.1287	17.9035	216.4171	0.7174	0.8354
A	3122.3023	26.3821	118.4864	0.4760	0.6450
A	3119.7736	26.9085	173.3025	0.5681	0.7246
A	3114.8048	60.5448	225.7094	0.7441	0.8533
A	3113.8742	45.3448	333.0501	0.3590	0.5283
A	3113.6642	86.1226	347.0185	0.7496	0.8569
A	3108.5517	25.8190	121.2072	0.7448	0.8537
A	3108.0491	55.1101	133.8608	0.3058	0.4683
A	3105.9961	50.8147	193.0520	0.7489	0.8564
A	3104.8122	24.7552	100.8733	0.7479	0.8558
A	3088.1847	100.7737	457.3544	0.7497	0.8570
A	3086.3544	8.3127	51.1764	0.7063	0.8279
A	3084.2125	7.5056	319.1843	0.1153	0.2068
A	3077.1577	54.7903	368.3419	0.0485	0.0925
A	3065.5867	63.9355	632.5112	0.0518	0.0985
A	3062.0653	8.5025	30.7013	0.7377	0.8491
A	3048.6983	23.5681	1149.8052	0.0078	0.0155
A	3042.6041	53.1908	26.8996	0.2766	0.4333
A	3039.6886	80.6428	378.6989	0.1777	0.3017
A	3037.4058	48.2116	121.9279	0.0087	0.0173
A	3036.4220	81.0972	632.9386	0.0325	0.0630
A	3031.5401	15.1680	446.4391	0.1638	0.2815
A	3025.3439	60.7786	300.6181	0.0087	0.0173
A	2343.6632	55.3951	389.4615	0.1576	0.2722
A	1754.0030	641.6678	30.5127	0.1958	0.3275
A	1722.2173	699.8420	20.1142	0.3048	0.4672
A	1719.9135	826.8208	35.2031	0.3232	0.4885
A	1649.3420	546.4582	38.9865	0.2417	0.3893
A	1579.5242	238.4045	18.3939	0.0896	0.1644
A	1564.8703	584.0504	6.4523	0.5809	0.7349
A	1519.1047	13.3388	22.9712	0.7124	0.8321
A	1506.8846	30.0302	5.4920	0.6092	0.7571
A	1503.5939	5.4818	22.9934	0.5221	0.6860
A	1502.3882	12.4115	14.3101	0.6384	0.7793
A	1498.2796	4.8891	23.1501	0.7463	0.8547
A	1494.3483	6.0429	35.2458	0.7424	0.8522
A	1488.5307	29.7422	27.6355	0.7454	0.8541
A	1487.2455	13.5448	1.2504	0.7500	0.8571
A	1486.4300	4.0808	11.5406	0.7369	0.8485
A	1484.1255	1.0385	29.3054	0.7500	0.8571
A	1482.5053	36.7006	1.3546	0.2431	0.3912
A	1480.2841	3.4231	10.4678	0.6979	0.8221
A	1476.3769	33.1618	17.7805	0.6815	0.8106
A	1474.0513	2.0257	32.9898	0.7492	0.8566
A	1472.3955	284.8790	12.6490	0.0562	0.1064
A	1457.5373	73.2617	14.7122	0.4979	0.6648

A	1442.6524	20.5289	56.4688	0.2271	0.3702
A	1427.6271	7.9310	2.8413	0.0789	0.1462
A	1421.0056	3.8513	9.0033	0.4751	0.6442
A	1412.2047	4.9274	3.0945	0.4866	0.6547
A	1407.9653	20.1708	1.0675	0.7492	0.8566
A	1401.3359	10.2667	0.9294	0.0803	0.1486
A	1394.2276	21.5051	26.6153	0.7115	0.8314
A	1382.4075	4.1983	9.6004	0.7497	0.8570
A	1377.1346	15.4720	3.8476	0.2394	0.3864
A	1376.7990	53.6582	37.5604	0.7499	0.8571
A	1365.0557	2.8257	5.4909	0.3591	0.5285
A	1362.3810	7.2101	6.4176	0.6816	0.8107
A	1344.6430	21.8390	5.9895	0.5290	0.6920
A	1343.4830	32.3738	13.0487	0.7273	0.8421
A	1331.2369	0.8816	2.8218	0.6776	0.8079
A	1325.2803	47.5680	13.2602	0.5572	0.7156
A	1315.3555	2.3213	9.3194	0.3048	0.4672
A	1312.4304	27.7339	19.3954	0.1601	0.2759
A	1302.9218	86.7839	50.2208	0.7203	0.8374
A	1301.4534	61.7422	17.6453	0.6348	0.7766
A	1282.7187	40.5196	10.2317	0.2906	0.4504
A	1277.0401	74.2361	5.3129	0.6464	0.7852
A	1252.4453	9.3262	28.5658	0.4715	0.6408
A	1247.3340	57.9483	49.5544	0.6491	0.7872
A	1246.0315	29.8371	15.8191	0.2266	0.3695
A	1237.6359	55.5330	20.6051	0.5652	0.7222
A	1227.8474	10.4508	22.7664	0.4904	0.6581
A	1219.9558	7.0485	6.2190	0.7453	0.8541
A	1211.2728	15.5029	15.6590	0.6233	0.7680
A	1204.5590	1.2466	12.8141	0.4875	0.6555
A	1199.7993	16.9217	6.3328	0.5379	0.6995
A	1198.0333	366.7035	5.2294	0.3612	0.5307
A	1185.6365	29.6603	6.6208	0.3215	0.4865
A	1151.4783	15.8984	16.7206	0.7482	0.8560
A	1144.6257	270.2690	1.6771	0.7197	0.8370
A	1143.9161	121.5433	12.0759	0.3716	0.5418
A	1133.2931	1.8994	7.5405	0.5845	0.7377
A	1126.0891	2.3716	25.2639	0.3111	0.4746
A	1114.3773	443.9438	3.2951	0.7226	0.8390
A	1109.1015	1.9538	15.2840	0.3656	0.5355
A	1103.6463	68.6865	4.9114	0.7485	0.8562
A	1092.4122	16.1442	11.2687	0.4314	0.6028
A	1068.5603	9.7544	8.5446	0.2613	0.4144
A	1064.9118	11.1492	8.8326	0.1844	0.3115
A	1063.3939	15.8692	5.5231	0.7353	0.8475
A	1053.6526	4.2222	12.8567	0.4684	0.6380
A	1043.2386	7.7355	7.6248	0.7285	0.8429
A	1041.3129	2.3916	2.0380	0.5158	0.6806
A	1032.9484	27.5815	6.0417	0.6942	0.8195
A	1021.4432	4.5498	11.6794	0.2482	0.3977
A	1005.8907	6.1774	4.8798	0.6093	0.7572

A	993.0807	8.2541	11.7428	0.5968	0.7475
A	981.3962	18.7803	15.8028	0.1545	0.2677
A	975.1780	4.4829	4.2077	0.7375	0.8489
A	971.7840	2.3483	4.9836	0.6285	0.7719
A	969.9935	3.5511	10.6378	0.5800	0.7342
A	953.4812	0.6130	8.8750	0.7228	0.8391
A	950.4105	7.3376	16.7850	0.3734	0.5438
A	947.8975	6.5174	11.9959	0.2875	0.4466
A	946.8737	9.9390	14.0731	0.7198	0.8371
A	926.2246	16.4596	24.9416	0.2171	0.3567
A	900.9014	7.2096	27.5730	0.2132	0.3515
A	896.6650	7.9319	11.1213	0.4259	0.5974
A	889.2206	31.2899	11.5046	0.2682	0.4229
A	846.9434	4.4487	7.1180	0.0850	0.1566
A	843.5857	2.9415	5.2662	0.5164	0.6811
A	825.3500	18.4808	10.5569	0.7491	0.8565
A	808.4109	0.8128	9.1593	0.1311	0.2319
A	795.0706	5.8583	1.8584	0.1611	0.2775
A	789.9610	10.4749	13.2861	0.1408	0.2468
A	767.2589	30.1453	3.4985	0.5805	0.7346
A	760.0677	20.0360	8.7994	0.2720	0.4276
A	740.2443	4.7713	4.6992	0.7223	0.8388
A	714.9995	95.0599	2.1885	0.5154	0.6802
A	711.9788	14.0724	4.2664	0.1394	0.2446
A	708.4419	66.7541	15.9030	0.0930	0.1702
A	698.1188	40.0177	18.0985	0.0454	0.0868
A	695.8636	13.1279	3.6447	0.7442	0.8533
A	683.2643	27.9676	14.9831	0.5685	0.7249
A	641.6848	55.7117	1.6369	0.7378	0.8491
A	625.7053	21.7823	2.8730	0.3735	0.5439
A	603.1925	37.7266	3.6702	0.1162	0.2083
A	598.2635	26.5435	6.5822	0.3543	0.5232
A	571.2632	37.2387	1.7285	0.7479	0.8558
A	567.4047	140.4120	0.9148	0.3842	0.5551
A	550.8311	10.6226	6.5223	0.2243	0.3665
A	537.0178	181.6754	1.5219	0.5805	0.7346
A	533.7706	19.8554	3.7894	0.3518	0.5205
A	500.8266	60.8076	1.2285	0.7032	0.8257
A	484.4447	16.4543	2.1982	0.7498	0.8570
A	473.6986	13.9221	4.0057	0.1342	0.2367
A	458.5187	12.7190	0.3643	0.2418	0.3894
A	451.9338	16.3425	1.0621	0.7492	0.8566
A	423.8942	9.0058	1.4464	0.5863	0.7392
A	415.3854	11.0001	1.4315	0.4959	0.6630
A	405.3714	6.0495	2.4802	0.5699	0.7260
A	398.3991	2.0705	0.6540	0.6788	0.8086
A	386.0507	4.7336	1.0275	0.6689	0.8016
A	372.5510	28.6368	0.9478	0.6413	0.7814
A	364.6859	7.1480	0.8204	0.7380	0.8493
A	361.2067	23.6867	1.7981	0.7469	0.8551
A	331.3825	1.1852	0.5480	0.7388	0.8498

A	319.7315	3.8878	0.6508	0.2505	0.4006
A	308.8287	17.8916	3.4501	0.5033	0.6696
A	306.0200	5.7713	0.9975	0.7414	0.8515
A	292.6874	7.0752	0.9879	0.2795	0.4369
A	289.9742	2.9672	1.4555	0.1460	0.2548
A	288.0826	1.7033	1.4140	0.7325	0.8456
A	279.0773	3.1611	1.6240	0.5120	0.6772
A	270.1400	7.4571	3.0603	0.7155	0.8341
A	258.4559	9.1641	0.9641	0.0572	0.1082
A	254.7261	4.2155	1.7673	0.2971	0.4581
A	245.6031	10.2631	5.8135	0.2997	0.4611
A	237.5345	14.1311	2.1532	0.4209	0.5924
A	235.3806	5.0985	1.3804	0.6462	0.7851
A	223.6209	3.2022	0.6591	0.1991	0.3320
A	205.0058	15.4599	2.7865	0.1255	0.2230
A	193.2109	13.2702	4.3605	0.6028	0.7522
A	185.8566	5.8829	1.0501	0.2401	0.3872
A	175.1530	3.6127	1.9574	0.3448	0.5128
A	172.0450	7.3274	2.6406	0.7046	0.8267
A	153.5549	0.6355	1.0671	0.5108	0.6762
A	145.1785	0.6991	0.2026	0.5795	0.7338
A	118.6001	3.2660	0.4530	0.7499	0.8571
A	110.2059	4.8643	0.4548	0.6995	0.8232
A	102.2312	0.7391	0.5198	0.3970	0.5683
A	91.7036	0.6009	0.3597	0.7030	0.8256
A	82.7435	5.8584	2.7687	0.7489	0.8564
A	77.3374	0.6008	0.7402	0.7278	0.8425
A	71.0838	5.3029	2.6679	0.7488	0.8564
A	62.8283	11.0430	1.7653	0.7383	0.8494
A	51.6577	0.7063	3.0198	0.7498	0.8570
A	44.4681	2.0942	2.9748	0.7476	0.8556
A	41.7239	8.9895	0.8249	0.7488	0.8564
A	36.7566	5.3681	0.4438	0.7252	0.8407
A	31.7293	1.7007	0.1125	0.7449	0.8538
A	24.8472	0.6692	0.5115	0.7491	0.8565
A	22.0020	5.2428	0.3885	0.7492	0.8566
A	16.5213	3.0423	0.4433	0.7478	0.8557
A	11.5239	2.5665	0.3429	0.7487	0.8563

ccl00:/aue/chem126/aue/ark/nir> gtg nira6pbed3jacs.log
Processing: nira6pbed3jacs.log
67

C	-5.593891	-0.536617	-0.490994
C	-4.670313	-1.293825	0.468117
C	-4.709925	-2.737018	-0.068337
C	-6.076319	-2.838347	-0.769765
N	-6.315404	-1.452915	-1.176114
C	-3.259435	-0.716140	0.567122
C	-3.174749	0.691684	1.192273
C	-3.719741	0.710383	2.563505
N	-4.149486	0.709012	3.642474

O	-5.681574	0.691886	-0.612280
N	-1.806032	1.178393	1.208903
C	-1.400677	2.215156	0.431216
O	-2.169119	2.921897	-0.222648
C	0.130559	2.415842	0.433151
C	0.527755	3.719495	-0.218995
C	1.201698	3.400280	-1.539749
C	1.224558	1.895793	-1.705869
N	0.774859	1.367519	-0.395622
C	1.995535	4.037641	-0.417112
C	2.307073	5.513729	-0.603800
C	3.097640	3.291589	0.315317
C	1.047361	0.160317	0.137046
C	1.774520	-0.884925	-0.714613
C	0.811643	-1.948011	-1.347820
C	-0.220217	-1.227344	-2.230647
O	0.720617	-0.134542	1.304772
N	2.769202	-1.502435	0.156788
C	4.076608	-1.501448	-0.121890
C	4.975679	-2.123840	0.976139
F	5.720599	-3.124887	0.461362
C	1.660810	-2.882105	-2.226335
C	0.085584	-2.776123	-0.275914
O	4.626993	-1.068991	-1.131171
F	5.826599	-1.192899	1.462121
F	4.285893	-2.629110	2.026129
H	1.501187	6.030514	-1.135196
H	3.231366	5.643150	-1.180084
H	2.443701	6.006424	0.366674
H	2.894726	2.228438	0.452536
H	4.040299	3.380935	-0.237879
H	3.253319	3.731739	1.307566
H	0.990005	3.986009	-2.428674
H	-0.179599	4.535972	-0.136755
H	0.497600	2.308561	1.458612
H	2.218410	1.508974	-1.939791
H	0.534508	1.590632	-2.497110
H	2.324339	-0.415092	-1.527413
H	2.449868	-1.839245	1.058152
H	-0.875611	-1.966809	-2.702173
H	-0.851586	-0.546175	-1.652119
H	0.268897	-0.655233	-3.025499
H	1.013567	-3.620399	-2.711464
H	2.184997	-2.321931	-3.008229
H	2.403657	-3.425826	-1.634069
H	-0.557376	-3.514211	-0.767285
H	0.789840	-3.323346	0.360256
H	-0.541404	-2.152776	0.364789
H	-1.078720	0.578411	1.600136
H	-3.773976	1.386731	0.598074
H	-2.819464	-0.639415	-0.431875

H	-2.632294	-1.397844	1.150728
H	-3.908136	-2.873511	-0.802590
H	-4.588472	-3.485074	0.717595
H	-6.861289	-3.179264	-0.083097
H	-6.061512	-3.500471	-1.638635
H	-7.087822	-1.176887	-1.770724
H	-5.158685	-1.256941	1.452369

Conformer **C**: B3LYPD3BJ/6-31G(d)

Processing: nirb6dbed3j.log
PG=C01

Method	BasisSet	Imaginary Freqs
RB3LYP	6-31G(d)	0

HF Energy
-1770.0740894

ZPE	E298	S298	Squasihar	Equasihar	Strans	Srot
347.06914	369.498	227.187	203.672	369.606	44.511	37.564

Label	Frequencies	IR Inten	Raman Activ	Depolar (P)	Depolar (U)	Dipole
A	3631.4536	36.4390	169.6530	0.2749	0.4313	3.2344
A	3619.0557	59.8197	33.6264	0.1050	0.1901	
A	3480.8885	159.8457	70.6293	0.1561	0.2700	
A	3220.0629	2.5510	82.4168	0.1722	0.2938	
A	3193.3786	10.9296	93.2720	0.3254	0.4910	
A	3167.9010	18.5696	54.5303	0.6665	0.7999	
A	3154.2204	13.1985	59.6458	0.4476	0.6184	
A	3151.0695	22.2270	49.9774	0.5795	0.7338	
A	3146.6725	15.2330	70.4281	0.7428	0.8524	
A	3144.6084	6.4995	31.8800	0.1043	0.1889	
A	3142.7839	2.4051	100.2413	0.2398	0.3868	
A	3134.4327	36.6694	77.9876	0.7226	0.8390	
A	3130.6345	7.2437	25.7899	0.4169	0.5885	
A	3122.9841	24.3630	53.8748	0.7061	0.8278	
A	3119.1154	25.2874	40.9065	0.7116	0.8315	
A	3116.3981	26.8256	57.6623	0.7197	0.8370	
A	3112.7824	20.5346	31.1560	0.6701	0.8025	
A	3106.2885	40.8311	146.4042	0.7496	0.8569	
A	3103.8193	35.3664	133.0783	0.3323	0.4989	
A	3102.7189	0.0585	6.7074	0.7485	0.8562	
A	3086.4850	20.3258	9.6406	0.2874	0.4464	
A	3085.2814	5.5459	46.5550	0.1116	0.2008	
A	3080.5018	13.4738	41.3562	0.2797	0.4372	
A	3071.8454	20.9456	100.6125	0.0318	0.0617	
A	3066.8640	20.0871	232.4790	0.0280	0.0544	
A	3064.5226	3.5674	6.8808	0.6218	0.7668	

A	3056.0344	17.2205	87.0173	0.0310	0.0601
A	3052.2233	30.0292	55.5836	0.1055	0.1909
A	3051.8634	36.0116	119.0618	0.0470	0.0898
A	3041.8474	35.1420	85.8274	0.0403	0.0774
A	3028.0396	70.8801	160.6603	0.2094	0.3463
A	3017.3546	8.6013	65.9767	0.2256	0.3681
A	2366.6721	5.6802	105.0158	0.2316	0.3760
A	1816.7928	357.4619	10.3694	0.4845	0.6527
A	1809.5905	335.1080	7.8125	0.2122	0.3500
A	1789.6301	238.5566	2.8112	0.4614	0.6314
A	1692.2887	208.5991	9.5025	0.1840	0.3109
A	1579.7966	269.5550	1.5048	0.7454	0.8542
A	1569.1651	220.0604	3.0068	0.2842	0.4426
A	1559.9852	3.1108	24.6152	0.5493	0.7091
A	1554.4485	18.9157	2.8381	0.5116	0.6769
A	1545.6109	10.0852	8.9409	0.7096	0.8301
A	1542.4400	7.6774	20.5679	0.7483	0.8560
A	1539.2812	5.0576	13.4082	0.7401	0.8506
A	1535.1654	18.8565	38.8982	0.7407	0.8511
A	1532.7885	5.2634	3.3115	0.7422	0.8520
A	1526.4487	3.5722	14.9563	0.7488	0.8564
A	1522.1526	5.2426	0.3133	0.1325	0.2340
A	1520.9010	12.7976	8.1281	0.7494	0.8568
A	1517.5784	1.5985	16.4540	0.7363	0.8482
A	1514.9692	0.5915	20.8861	0.7500	0.8571
A	1513.1070	5.7757	14.1533	0.7405	0.8509
A	1506.8349	11.9173	2.8412	0.7410	0.8512
A	1479.3823	195.7847	2.5686	0.0895	0.1644
A	1466.5098	10.5273	3.3154	0.5281	0.6912
A	1465.9289	46.3434	0.6898	0.4478	0.6186
A	1464.6160	42.8875	7.8871	0.2254	0.3678
A	1451.0628	2.6060	10.2340	0.2119	0.3497
A	1439.9458	2.4970	8.5672	0.6768	0.8073
A	1436.7150	10.2392	1.1199	0.7456	0.8543
A	1432.4443	5.0385	2.6479	0.7489	0.8564
A	1414.7150	8.4763	9.5974	0.7019	0.8248
A	1396.5855	9.7672	5.7928	0.5624	0.7199
A	1389.8251	4.7970	1.4435	0.6131	0.7602
A	1387.0176	3.7839	3.6674	0.2807	0.4384
A	1384.9021	37.3420	10.8828	0.7449	0.8538
A	1380.1540	2.3749	7.8387	0.7016	0.8247
A	1365.6787	4.6740	4.6365	0.7328	0.8458
A	1362.1749	27.0074	1.5479	0.7342	0.8467
A	1348.1053	30.7915	2.8628	0.7363	0.8481
A	1345.9811	19.3446	6.7364	0.7456	0.8543
A	1336.2078	7.7597	3.8240	0.7000	0.8235
A	1333.5901	1.1361	6.7497	0.2832	0.4414
A	1321.1432	39.4598	2.2318	0.6088	0.7569
A	1304.9181	107.7672	7.0968	0.3207	0.4856
A	1296.6498	104.0281	13.2609	0.6604	0.7955
A	1283.1212	62.3643	2.2468	0.5248	0.6883

A	1269.4567	18.4733	2.4554	0.3311	0.4974
A	1267.6308	11.5494	5.9969	0.5068	0.6727
A	1255.6374	3.2209	2.7624	0.6456	0.7846
A	1254.6512	12.2623	7.5192	0.5872	0.7399
A	1245.2000	253.6562	0.9614	0.6984	0.8224
A	1239.6788	62.4243	4.1305	0.5740	0.7293
A	1234.1981	30.8139	1.4469	0.6586	0.7942
A	1230.8077	71.6268	4.5416	0.4560	0.6264
A	1227.6109	9.1997	7.4996	0.5273	0.6905
A	1222.6205	186.3638	2.8173	0.5256	0.6890
A	1207.9259	8.3930	3.9326	0.7343	0.8468
A	1199.6819	18.6045	1.5990	0.4091	0.5806
A	1178.8751	122.0632	3.0611	0.3331	0.4997
A	1171.0784	2.5804	1.6232	0.6818	0.8108
A	1155.5818	7.3395	4.6199	0.5271	0.6903
A	1147.2747	5.4548	4.1778	0.5597	0.7177
A	1130.2422	3.6071	1.8963	0.7422	0.8520
A	1127.5052	1.8785	6.6496	0.0952	0.1738
A	1125.5946	1.7266	1.8060	0.5272	0.6904
A	1108.1325	7.3528	7.3322	0.3410	0.5085
A	1084.7218	5.7130	4.1048	0.5584	0.7166
A	1078.3793	2.0583	1.8530	0.2450	0.3936
A	1071.2570	26.3161	6.0421	0.6956	0.8205
A	1066.9094	0.8935	7.3257	0.5266	0.6899
A	1062.5705	2.9185	4.1137	0.7375	0.8489
A	1054.2622	0.7541	1.6173	0.6472	0.7858
A	1030.9030	2.0770	9.1554	0.2916	0.4515
A	1020.8014	3.3157	2.2202	0.1782	0.3025
A	1014.3419	0.6287	2.1185	0.4022	0.5737
A	1004.6709	5.9301	4.1356	0.7108	0.8310
A	987.5422	7.1689	3.8872	0.5968	0.7475
A	983.1757	2.6158	2.9916	0.6882	0.8153
A	982.3287	2.3612	8.0086	0.6852	0.8132
A	980.9567	0.0600	0.9864	0.7464	0.8548
A	967.6940	9.6989	4.6443	0.6995	0.8232
A	960.9862	5.3171	4.8353	0.4772	0.6461
A	953.6734	2.5151	4.8414	0.7006	0.8239
A	948.0536	6.6357	4.5841	0.4631	0.6330
A	936.0006	14.7419	11.7879	0.1143	0.2052
A	909.7992	1.4166	9.7199	0.6544	0.7911
A	904.6056	14.7228	5.4256	0.2363	0.3822
A	890.1038	0.8956	7.4970	0.3068	0.4696
A	855.3111	5.4342	1.5761	0.2321	0.3767
A	848.3175	5.9007	9.3604	0.1437	0.2513
A	836.0688	7.1841	4.4580	0.6989	0.8228
A	825.8587	2.0311	4.0354	0.7166	0.8349
A	812.2237	2.5957	2.0820	0.3816	0.5524
A	796.8081	4.9784	1.6408	0.3873	0.5584
A	773.8600	31.6274	2.1404	0.4264	0.5979
A	766.0279	6.1105	1.0211	0.1448	0.2530
A	748.7868	13.8715	2.1343	0.7426	0.8523

A	732.0885	65.0513	2.5872	0.1264	0.2244
A	718.9456	29.6530	0.6438	0.2031	0.3376
A	711.3673	8.5601	8.8694	0.2663	0.4206
A	706.9663	16.6737	7.8111	0.0607	0.1144
A	691.4590	13.1462	2.0767	0.6269	0.7707
A	688.9433	5.4683	6.6860	0.4671	0.6368
A	656.1044	27.8373	1.4417	0.4727	0.6420
A	617.1994	76.0029	1.4614	0.2435	0.3916
A	614.2988	29.2539	0.9171	0.6717	0.8036
A	608.7340	31.1550	2.0451	0.1379	0.2424
A	598.2728	14.7617	0.7094	0.4292	0.6006
A	566.3252	4.4246	0.3088	0.4967	0.6637
A	552.6423	27.8238	2.1760	0.2567	0.4085
A	546.0406	3.9101	2.0848	0.3475	0.5158
A	537.0514	53.3754	2.5593	0.6036	0.7528
A	511.1344	33.6951	2.4668	0.7461	0.8546
A	506.7826	11.6575	0.9679	0.7364	0.8482
A	476.6658	3.5246	2.2768	0.0720	0.1344
A	457.3678	8.8092	0.1911	0.5937	0.7450
A	451.8161	8.5228	0.4674	0.7043	0.8265
A	421.8362	6.4253	0.7652	0.2257	0.3683
A	419.5112	5.2503	0.7516	0.4159	0.5874
A	407.2413	2.4835	1.0558	0.7499	0.8571
A	402.4066	2.9434	0.8032	0.4542	0.6247
A	391.3710	2.0448	1.0806	0.7497	0.8569
A	364.8141	8.5599	0.4322	0.2828	0.4409
A	362.6353	18.0127	0.3589	0.2912	0.4510
A	356.1730	24.2062	0.8070	0.7485	0.8562
A	339.8130	0.4618	0.9269	0.5746	0.7299
A	323.2098	3.8787	0.3989	0.5922	0.7439
A	316.2223	1.8137	0.6904	0.7108	0.8310
A	310.5646	4.2596	0.9768	0.5512	0.7107
A	302.5604	7.0553	0.7222	0.4804	0.6490
A	290.4105	2.5647	1.0757	0.4223	0.5938
A	289.1926	2.1910	1.0603	0.6855	0.8134
A	277.3866	1.1537	0.5993	0.2582	0.4105
A	262.7579	1.8852	0.7441	0.7338	0.8465
A	255.4914	1.3873	2.1794	0.2455	0.3942
A	254.5652	5.8885	0.4720	0.2836	0.4419
A	238.5505	7.7184	1.7434	0.7377	0.8490
A	229.9866	0.3426	0.0979	0.7497	0.8570
A	223.4187	0.5570	0.8140	0.1378	0.2422
A	214.8375	2.5241	0.7961	0.1744	0.2970
A	199.2637	1.4869	1.0853	0.1847	0.3118
A	188.9368	5.1167	1.4566	0.5607	0.7185
A	182.6132	4.0304	0.8438	0.5550	0.7138
A	177.2721	1.9422	0.7479	0.0933	0.1707
A	169.3436	1.8003	0.5967	0.7334	0.8462
A	153.0104	3.3797	0.3468	0.7377	0.8490
A	141.4724	1.7747	0.8019	0.7496	0.8569
A	131.5056	5.0861	2.1050	0.4953	0.6625

A	111.5459	2.6528	1.0835	0.7351	0.8473
A	104.3571	5.4719	0.5863	0.7344	0.8468
A	103.0404	0.4510	0.2600	0.5669	0.7236
A	91.8470	0.2492	0.1008	0.7346	0.8470
A	80.8749	1.5650	0.5588	0.7401	0.8507
A	76.6305	3.5609	0.6534	0.7063	0.8279
A	62.0481	1.3932	0.9816	0.7280	0.8426
A	55.9763	0.9057	0.3267	0.7463	0.8548
A	45.0648	2.2634	0.7428	0.7496	0.8569
A	40.3025	2.2170	0.9524	0.7359	0.8479
A	38.7778	1.1970	0.2385	0.7475	0.8555
A	37.2924	2.6133	0.5803	0.7046	0.8267
A	27.4023	1.1179	0.3422	0.6873	0.8147
A	19.6744	0.4680	1.1986	0.7480	0.8558
A	18.9327	1.2327	0.3535	0.7406	0.8510
A	12.5127	0.2272	0.1250	0.7495	0.8568

67

C	-4.523431	-1.826832	-1.003005
C	-4.629964	-0.536934	-0.167054
C	-5.673355	-0.894213	0.907659
C	-6.547492	-1.971947	0.232549
N	-5.612716	-2.587370	-0.701018
C	-3.243007	-0.102032	0.294301
C	-3.137475	1.332743	0.843244
C	-3.916933	1.501721	2.084095
N	-4.542311	1.595663	3.057088
O	-3.632998	-2.113570	-1.790207
N	-1.746923	1.701277	1.061882
C	-1.138851	2.607570	0.239457
O	-1.736511	3.309739	-0.564727
C	0.396248	2.639786	0.395603
C	0.983082	3.879062	-0.238807
C	1.732701	3.459528	-1.485171
C	1.609444	1.957354	-1.630053
N	0.983119	1.512546	-0.367921
C	2.486565	4.039776	-0.305650
C	2.971884	5.468219	-0.489830
C	3.429018	3.198395	0.537590
C	1.009215	0.273206	0.170928
C	1.606827	-0.878273	-0.638951
C	0.510459	-1.744519	-1.352838
C	-0.331906	-0.844044	-2.272640
O	0.551750	0.041413	1.303238
N	2.405423	-1.658219	0.293702
C	3.685475	-1.993642	0.026050
C	4.376997	-2.783710	1.158718
F	4.562969	-4.058566	0.780176
C	1.226485	-2.803034	-2.208501
C	-0.393032	-2.451052	-0.328730
O	4.292077	-1.763309	-1.007465

F	5.569664	-2.246004	1.441088
F	3.643362	-2.795268	2.300028
H	2.278143	6.053904	-1.102624
H	3.952474	5.485052	-0.980697
H	3.072127	5.972478	0.478810
H	3.085676	2.172096	0.682809
H	4.419341	3.154834	0.068837
H	3.553566	3.649567	1.529164
H	1.660605	4.043194	-2.397563
H	0.348944	4.757770	-0.246767
H	0.663124	2.506289	1.448853
H	2.574885	1.460224	-1.762195
H	0.971968	1.709536	-2.484528
H	2.296793	-0.516981	-1.401525
H	2.028083	-1.783006	1.224343
H	-1.150853	-1.418365	-2.713784
H	-0.783839	-0.007675	-1.730212
H	0.277155	-0.430576	-3.085211
H	0.485885	-3.407056	-2.743952
H	1.891567	-2.340938	-2.946027
H	1.826906	-3.477286	-1.590358
H	-1.206969	-2.966184	-0.846955
H	0.173857	-3.190961	0.247058
H	-0.835191	-1.745567	0.376430
H	-1.136149	1.038802	1.539400
H	-3.519957	2.043158	0.101544
H	-2.575026	-0.164091	-0.567910
H	-2.864113	-0.804674	1.046062
H	-5.167702	-1.324081	1.779056
H	-6.257743	-0.041066	1.254875
H	-7.403461	-1.527484	-0.295008
H	-6.932891	-2.705645	0.946909
H	-5.808242	-3.430608	-1.222835
H	-5.037322	0.229660	-0.844263

Conformer **C**: B3LYPD3BJ/6-31+G(d,p)

Processing: nirb6pbed3j.log
PG=C01

Method	BasisSet	Imaginary Freqs
RB3LYP	6-31+G(d,p)	0

HF Energy
-1770.1954274

ZPE	E298	S298	Squasihar	Equasihar	Strans	Srot
344.63795	367.254	229.918	204.552	367.366	44.511	37.576

Population analysis using the SCF Density.
Label Frequencies IR Inten Raman Activ Depolar (P) Depolar (U) Dipole

A	3644.8362	45.2617	176.0968	0.2345	0.3799	3.8756
A	3617.6783	65.3090	39.2929	0.0766	0.1423	
A	3488.2601	168.4457	75.8342	0.1317	0.2328	
A	3210.2788	2.7335	99.0427	0.1450	0.2533	
A	3185.2448	11.3392	113.7810	0.2922	0.4522	
A	3156.1319	18.9813	57.7127	0.6375	0.7786	
A	3145.6708	12.5291	62.5227	0.4250	0.5965	
A	3144.4083	22.3181	55.0880	0.5450	0.7055	
A	3143.2877	4.6658	37.0101	0.0843	0.1555	
A	3134.4258	18.0782	74.0403	0.6960	0.8207	
A	3129.6601	2.1835	81.4949	0.2601	0.4128	
A	3123.0624	35.0996	73.6694	0.7497	0.8569	
A	3118.5306	24.6921	63.1178	0.5382	0.6998	
A	3115.8457	22.9469	40.9871	0.7157	0.8343	
A	3113.7615	11.0737	37.0644	0.7495	0.8568	
A	3108.9234	28.4487	62.8082	0.7299	0.8439	
A	3107.4037	12.6591	31.4563	0.6993	0.8231	
A	3099.6558	29.7592	147.3791	0.3367	0.5038	
A	3097.5765	39.8633	154.3980	0.7493	0.8567	
A	3094.5673	1.6609	14.3669	0.7483	0.8560	
A	3078.6039	21.7913	12.0933	0.1522	0.2643	
A	3074.9951	4.6027	55.9400	0.0755	0.1404	
A	3071.2907	14.6104	52.8880	0.1506	0.2618	
A	3063.2782	22.0754	118.9947	0.0232	0.0453	
A	3056.6542	13.3655	146.7624	0.0392	0.0755	
A	3054.0175	7.6443	216.4405	0.0122	0.0241	
A	3045.9633	24.9622	87.4674	0.0392	0.0754	
A	3042.3310	28.7922	64.8352	0.0557	0.1055	
A	3041.9284	38.0170	178.6783	0.0524	0.0995	
A	3030.7152	43.3431	140.1340	0.0473	0.0904	
A	3023.0994	67.3074	191.1442	0.1918	0.3219	
A	3013.4561	7.7896	89.2026	0.1430	0.2502	
A	2354.9867	8.0122	141.1271	0.1732	0.2953	
A	1785.5478	68.8008	1.7891	0.6463	0.7852	
A	1783.8714	741.0741	23.0020	0.2326	0.3774	
A	1758.4030	281.4835	4.8830	0.3814	0.5522	
A	1672.5088	235.0914	10.7996	0.1620	0.2788	
A	1570.1381	261.1598	1.4889	0.7329	0.8459	
A	1562.0734	210.4206	4.8802	0.1261	0.2239	
A	1538.0254	4.0571	15.0688	0.5798	0.7340	
A	1530.3702	24.2003	1.4371	0.4797	0.6483	
A	1522.3842	7.7801	2.5976	0.7468	0.8550	
A	1518.6779	4.7459	9.9138	0.7033	0.8258	
A	1514.9318	7.2013	6.9482	0.7134	0.8327	
A	1510.6822	12.8981	18.0636	0.7457	0.8543	
A	1508.4200	8.1838	1.1766	0.7495	0.8568	
A	1503.1206	13.9112	2.5204	0.6531	0.7901	
A	1499.5470	2.8951	11.7462	0.7485	0.8562	
A	1496.6924	8.9951	0.2399	0.7404	0.8508	
A	1494.1292	2.0322	15.2105	0.7106	0.8308	
A	1491.1777	12.4086	3.1671	0.7488	0.8563	

A	1489.5213	0.6711	10.4086	0.7500	0.8571
A	1483.9177	12.1951	2.0272	0.7475	0.8555
A	1473.3348	203.3511	3.4414	0.0240	0.0469
A	1454.0325	52.4351	1.7519	0.3746	0.5450
A	1451.7228	22.6859	15.7605	0.2313	0.3756
A	1445.1109	7.6754	0.3816	0.7269	0.8419
A	1431.4840	3.3803	5.2483	0.2440	0.3923
A	1418.4672	3.8186	2.1327	0.7447	0.8537
A	1415.9477	10.8238	0.7126	0.7192	0.8366
A	1411.3395	7.7662	0.5446	0.6104	0.7581
A	1400.6815	7.7652	9.2019	0.6860	0.8137
A	1385.4707	8.5052	3.6913	0.5775	0.7322
A	1377.2399	3.8344	1.1343	0.7261	0.8413
A	1375.7022	34.7475	11.7143	0.7414	0.8515
A	1370.0902	3.1235	3.5799	0.3904	0.5616
A	1368.5113	0.4775	4.1763	0.7464	0.8548
A	1351.9135	4.6797	2.9604	0.5672	0.7238
A	1350.7382	23.0176	2.7423	0.7379	0.8492
A	1336.9879	18.5686	3.2901	0.7173	0.8354
A	1332.3070	19.5679	5.1332	0.7408	0.8511
A	1326.6742	10.4020	4.0428	0.7478	0.8557
A	1322.7521	0.2203	6.3448	0.3152	0.4793
A	1311.3676	32.1707	3.0455	0.5108	0.6762
A	1296.8095	72.6012	7.2658	0.3598	0.5292
A	1285.9966	122.8539	8.6765	0.6521	0.7894
A	1273.2963	36.7127	1.9656	0.6838	0.8122
A	1256.7131	11.3307	6.4943	0.5283	0.6913
A	1253.8638	14.3287	10.6152	0.5416	0.7026
A	1246.2569	3.0659	2.0844	0.6331	0.7753
A	1244.7085	9.8987	5.7400	0.4337	0.6050
A	1233.0636	51.2254	3.4320	0.6611	0.7960
A	1225.3071	18.4887	1.8394	0.5992	0.7494
A	1218.8128	44.4632	4.6913	0.5415	0.7026
A	1217.8184	5.2302	5.2445	0.3672	0.5371
A	1208.5785	288.5856	3.1052	0.2930	0.4532
A	1197.9440	7.1942	2.1431	0.6664	0.7998
A	1191.5149	15.7825	1.3205	0.3539	0.5228
A	1178.8711	257.5101	1.8271	0.7100	0.8304
A	1157.1689	4.5830	4.0946	0.7471	0.8553
A	1153.6870	142.5018	2.3532	0.3380	0.5052
A	1147.9416	11.6432	4.8823	0.4862	0.6543
A	1138.5843	4.3859	2.8522	0.4967	0.6637
A	1118.1398	4.5249	5.6835	0.1012	0.1838
A	1116.7573	0.9590	6.7216	0.3188	0.4835
A	1115.8404	8.5435	0.7878	0.6661	0.7996
A	1100.5355	5.6398	5.6134	0.2685	0.4233
A	1077.2656	5.8134	4.1060	0.4893	0.6571
A	1070.5643	2.6167	1.5351	0.3774	0.5480
A	1063.6217	28.3188	4.3715	0.6697	0.8022
A	1055.7246	1.1020	5.1682	0.3533	0.5222
A	1048.3493	2.5010	1.7669	0.7453	0.8541

A	1043.7754	0.8115	0.9645	0.6458	0.7848
A	1022.8432	2.0779	7.8102	0.1848	0.3120
A	1014.2613	3.6722	2.3656	0.1838	0.3105
A	1006.2899	0.6242	2.4771	0.3396	0.5070
A	997.3026	5.1448	3.2048	0.7333	0.8461
A	981.5689	5.7443	2.4169	0.2007	0.3343
A	974.8829	2.8137	6.1774	0.7493	0.8567
A	973.9211	2.6329	2.2099	0.7492	0.8566
A	969.3624	0.0728	0.1822	0.5432	0.7040
A	961.5156	8.7627	3.2126	0.5664	0.7232
A	952.8006	6.0727	4.0614	0.3781	0.5488
A	945.9125	2.2474	3.7771	0.6572	0.7931
A	942.5981	6.3688	4.6979	0.4252	0.5967
A	928.3955	15.6474	9.9621	0.1395	0.2448
A	903.5380	2.3878	9.8337	0.3824	0.5532
A	898.1328	17.7255	3.8984	0.2528	0.4036
A	884.6691	0.7241	8.0959	0.2779	0.4350
A	850.5093	7.2070	3.6685	0.0706	0.1319
A	844.2766	5.3902	10.1819	0.1096	0.1976
A	831.3933	7.7443	2.3919	0.7107	0.8309
A	820.8950	1.6786	3.5833	0.6438	0.7833
A	807.6307	1.3306	2.2810	0.1706	0.2914
A	794.0423	3.4454	1.3379	0.1996	0.3328
A	769.9768	22.9928	1.9626	0.3872	0.5582
A	763.3823	7.7463	1.9669	0.1313	0.2322
A	746.6671	8.9529	1.9584	0.6363	0.7778
A	719.1418	42.0368	0.9780	0.1516	0.2633
A	712.7912	24.3904	1.7932	0.1768	0.3005
A	707.5432	8.8700	11.2940	0.1471	0.2565
A	702.5594	23.8884	7.0407	0.0418	0.0802
A	688.7066	9.2418	1.5685	0.7163	0.8347
A	684.7041	15.7195	6.6954	0.4142	0.5857
A	653.0438	32.5901	1.2753	0.4437	0.6147
A	610.1351	21.5713	1.0255	0.4286	0.6000
A	608.4360	83.5216	0.4175	0.7208	0.8377
A	603.4722	24.0204	2.6642	0.0819	0.1515
A	594.0824	15.6860	0.6564	0.3804	0.5511
A	562.8436	3.1790	0.5864	0.3585	0.5278
A	547.9536	29.6229	1.9355	0.1583	0.2733
A	539.0735	3.9359	2.0256	0.3448	0.5127
A	532.2352	46.9228	2.0063	0.3482	0.5166
A	509.1006	35.4545	1.1801	0.7463	0.8547
A	504.6350	10.0426	0.9300	0.7306	0.8443
A	475.0511	3.0775	1.9229	0.0757	0.1408
A	457.1178	7.1249	0.1741	0.2554	0.4069
A	451.1551	9.6239	0.4017	0.7498	0.8570
A	421.7582	7.6494	0.6066	0.2719	0.4275
A	416.0853	3.2166	0.8655	0.3499	0.5184
A	405.5181	2.2141	1.0381	0.7233	0.8395
A	400.6223	4.0593	0.6974	0.5696	0.7258
A	389.1612	1.7212	1.0231	0.7495	0.8568

A	363.8020	1.0264	0.3243	0.6942	0.8195
A	362.0999	22.3721	0.3914	0.2106	0.3479
A	354.0719	25.3569	0.6946	0.6894	0.8162
A	338.3618	0.7100	0.8572	0.5453	0.7057
A	315.8927	2.1070	0.4136	0.7347	0.8471
A	314.8722	2.9079	0.6862	0.3217	0.4868
A	308.2853	5.0689	0.8560	0.5267	0.6900
A	296.9638	5.9337	0.7462	0.5470	0.7071
A	287.4789	1.8323	1.0540	0.6932	0.8188
A	285.7466	0.5573	0.6548	0.1957	0.3273
A	272.1064	0.6837	0.5288	0.3884	0.5595
A	261.8900	1.9090	0.7119	0.7026	0.8253
A	253.2290	2.9213	1.8029	0.1763	0.2998
A	252.7890	4.1221	1.2097	0.1555	0.2691
A	239.6277	9.0053	1.5556	0.6933	0.8189
A	223.6993	0.4195	0.1345	0.6498	0.7877
A	222.0939	0.6220	0.9721	0.1114	0.2005
A	213.2222	2.6712	0.8892	0.1209	0.2157
A	197.2254	1.6552	1.5407	0.1370	0.2409
A	190.1672	4.4627	1.2811	0.7090	0.8297
A	183.4511	4.2473	0.9363	0.4568	0.6272
A	175.7616	2.5993	0.9241	0.1771	0.3010
A	164.8903	1.9141	0.4823	0.7395	0.8502
A	152.0976	4.0758	0.3025	0.7140	0.8331
A	139.7449	1.8069	1.0190	0.7426	0.8523
A	130.5473	5.3539	2.1145	0.4403	0.6114
A	110.6717	1.5529	0.6869	0.6914	0.8175
A	102.2730	6.7626	0.6536	0.7500	0.8571
A	98.0649	0.2432	0.3045	0.5223	0.6862
A	81.9234	0.2411	0.0562	0.4366	0.6078
A	80.3121	1.3042	0.6914	0.7486	0.8562
A	77.4262	4.9115	0.6324	0.6911	0.8174
A	57.6478	1.2526	0.4245	0.7234	0.8395
A	50.6575	0.3706	0.6989	0.7385	0.8496
A	43.9152	3.7059	0.7733	0.7500	0.8571
A	36.8403	2.1279	0.7617	0.7455	0.8542
A	34.3020	3.4061	0.6173	0.7414	0.8515
A	31.8794	0.7466	0.2045	0.7245	0.8402
A	26.7124	2.5514	0.3902	0.6979	0.8221
A	19.2639	0.0201	1.0336	0.7456	0.8543
A	14.2999	1.0664	0.5876	0.7428	0.8524
A	13.6974	0.4628	0.2443	0.7439	0.8531

67

C	-4.623041	-1.775349	-1.000295
C	-4.689761	-0.504716	-0.134207
C	-5.720232	-0.867418	0.952340
C	-6.621109	-1.920884	0.274417
N	-5.712727	-2.531502	-0.692253
C	-3.288636	-0.098331	0.312552
C	-3.158309	1.330946	0.872633

C	-3.902471	1.500726	2.135122
N	-4.499869	1.598517	3.125529
O	-3.753340	-2.050588	-1.818267
N	-1.759908	1.687332	1.062426
C	-1.148706	2.563743	0.213464
O	-1.747666	3.245873	-0.611184
C	0.386722	2.595699	0.363949
C	0.979564	3.833577	-0.268022
C	1.738157	3.411345	-1.510019
C	1.613261	1.909511	-1.655519
N	0.970516	1.465356	-0.398272
C	2.484590	3.989522	-0.324421
C	2.976484	5.416886	-0.503906
C	3.418293	3.144317	0.525436
C	0.995031	0.227782	0.142396
C	1.606106	-0.922026	-0.661072
C	0.528435	-1.809661	-1.376990
C	-0.317673	-0.924065	-2.308092
O	0.528032	-0.003897	1.272560
N	2.421662	-1.687153	0.273691
C	3.729588	-1.920256	0.046349
C	4.453771	-2.684367	1.184701
F	4.867761	-3.888377	0.748710
C	1.270562	-2.859460	-2.222497
C	-0.380332	-2.524548	-0.363388
O	4.361637	-1.609708	-0.952124
F	5.527685	-1.994186	1.604032
F	3.653819	-2.894419	2.265247
H	2.290579	6.004368	-1.122445
H	3.960818	5.428832	-0.986007
H	3.069187	5.919277	0.465801
H	3.067213	2.122667	0.678406
H	4.407821	3.088817	0.057978
H	3.543987	3.601454	1.513411
H	1.672068	3.995398	-2.422262
H	0.349287	4.714825	-0.279565
H	0.653734	2.465040	1.417697
H	2.579599	1.411806	-1.774386
H	0.983560	1.664207	-2.515793
H	2.288660	-0.552154	-1.425228
H	2.022219	-1.878735	1.184157
H	-1.104592	-1.520117	-2.776533
H	-0.811472	-0.110702	-1.769081
H	0.297650	-0.484303	-3.100491
H	0.545185	-3.477791	-2.760609
H	1.931273	-2.387889	-2.957156
H	1.877529	-3.521342	-1.598162
H	-1.155193	-3.080971	-0.897950
H	0.188681	-3.233558	0.246961
H	-0.873771	-1.820740	0.307848
H	-1.152927	1.032896	1.554951

H	-3.558000	2.049153	0.147769
H	-2.631831	-0.157688	-0.558361
H	-2.910304	-0.813261	1.052253
H	-5.206158	-1.318116	1.807811
H	-6.288151	-0.013202	1.322348
H	-7.478725	-1.455021	-0.229731
H	-7.000869	-2.663452	0.981270
H	-5.933275	-3.356507	-1.232161
H	-5.095933	0.280510	-0.789947

Conformer **C**: B3LYPD3BJ-SMD/6-31+G(d,p), in water solvent

Processing: nirb6pbed3jws.log
PG=C01

Method	BasisSet	Imaginary Freqs
RB3LYP	6-31+G(d,p)	0

HF Energy
-1770.2368373

ZPE	E298	S298	Squasihar	Equasihar	Strans	Srot
343.83934	366.334	225.307	204.275	366.438	44.511	37.587

Population analysis using the SCF Density.

Label	Frequencies	IR Inten	Raman Activ	Depolar (P)	Depolar (U)	Dipole
A	3613.8636	116.2420	321.2165	0.2082	0.3447	5.4864
A	3599.2421	214.6188	166.8384	0.1037	0.1878	
A	3507.6697	269.2965	198.8827	0.0970	0.1768	
A	3205.7433	10.8446	421.5785	0.1411	0.2473	
A	3191.1745	19.3695	343.7303	0.3617	0.5312	
A	3169.2571	2.6651	197.2606	0.1214	0.2164	
A	3152.3979	30.1621	213.8571	0.6164	0.7626	
A	3148.5173	34.5254	241.2721	0.5226	0.6865	
A	3145.1560	31.0686	179.6247	0.5364	0.6982	
A	3137.5790	12.3130	262.1845	0.4369	0.6082	
A	3132.3500	18.7220	203.2436	0.7500	0.8571	
A	3130.9381	36.3557	302.2534	0.3759	0.5464	
A	3124.4679	44.1746	168.4447	0.6988	0.8227	
A	3119.7912	81.5937	331.1620	0.7459	0.8545	
A	3118.1720	60.5548	216.7024	0.7470	0.8552	
A	3113.7265	44.8926	201.0205	0.7288	0.8431	
A	3110.6595	17.4830	74.9454	0.7476	0.8556	
A	3109.4413	25.0170	125.0930	0.2103	0.3475	
A	3109.2011	51.1713	201.0463	0.7498	0.8570	
A	3099.7940	10.5219	277.4759	0.1285	0.2278	
A	3092.9004	93.2519	438.4645	0.7499	0.8571	
A	3091.1027	18.0927	86.6738	0.7500	0.8571	
A	3082.1619	46.6572	482.5842	0.0409	0.0787	
A	3074.9578	35.7561	599.4428	0.0469	0.0896	
A	3071.9416	23.6834	46.5163	0.5856	0.7386	

A	3058.7372	76.1030	424.6333	0.1527	0.2650
A	3053.6670	21.7924	1100.5489	0.0062	0.0123
A	3047.2320	55.9218	108.0625	0.0611	0.1152
A	3041.4162	46.7424	144.2006	0.0116	0.0229
A	3039.6939	78.6406	669.2429	0.0280	0.0545
A	3029.2333	13.3148	454.1267	0.1708	0.2918
A	3028.9409	59.4523	302.3478	0.0092	0.0183
A	2313.9429	48.3376	390.5801	0.1679	0.2875
A	1706.0659	832.0078	33.5531	0.1853	0.3127
A	1671.8832	916.5723	24.1945	0.1983	0.3309
A	1666.0462	932.4589	23.5126	0.7239	0.8398
A	1616.7310	973.3415	42.5263	0.3497	0.5182
A	1586.6986	232.1373	29.6704	0.1593	0.2748
A	1551.3330	534.8962	6.1368	0.6546	0.7912
A	1521.0101	28.4968	21.0152	0.7198	0.8371
A	1508.2640	35.6316	5.8594	0.4667	0.6364
A	1503.3615	16.6071	9.5543	0.3678	0.5378
A	1500.9870	8.9425	32.4805	0.6515	0.7890
A	1498.1466	5.2341	11.9913	0.6286	0.7719
A	1494.2795	8.0181	43.5864	0.7494	0.8568
A	1486.7437	15.2129	5.2212	0.6808	0.8101
A	1486.1777	37.2377	25.3326	0.7335	0.8462
A	1485.4574	11.6646	3.5546	0.7117	0.8316
A	1483.0571	10.4550	18.4901	0.7235	0.8396
A	1482.1920	183.7693	22.0959	0.2714	0.4269
A	1478.6456	77.8788	21.3688	0.3329	0.4995
A	1476.5882	82.8397	25.7107	0.6355	0.7772
A	1473.4122	2.9840	8.5988	0.5323	0.6947
A	1471.8537	0.7816	33.7653	0.7498	0.8570
A	1457.3792	75.7820	20.6518	0.4098	0.5814
A	1444.1911	14.9539	58.9209	0.2834	0.4416
A	1426.6834	5.1338	4.3431	0.0748	0.1391
A	1419.1980	2.8038	7.3675	0.5783	0.7328
A	1410.6201	5.4062	3.0230	0.5047	0.6708
A	1406.3114	16.8240	2.1744	0.5762	0.7312
A	1401.7219	6.6443	0.8806	0.1084	0.1956
A	1396.6964	10.7790	25.4742	0.6894	0.8161
A	1389.6484	9.3006	12.8465	0.3416	0.5092
A	1387.1815	87.1740	40.8889	0.7498	0.8570
A	1375.7558	9.3562	12.5214	0.3063	0.4690
A	1369.7533	1.8463	3.8487	0.6319	0.7744
A	1363.4784	11.1967	7.5311	0.5820	0.7358
A	1345.4960	39.5757	9.2986	0.7500	0.8571
A	1340.1336	4.7494	10.9139	0.3783	0.5490
A	1333.4995	49.0813	8.8574	0.6514	0.7889
A	1326.6080	52.3562	16.9042	0.7398	0.8504
A	1319.4436	39.5309	9.6963	0.7378	0.8491
A	1317.3568	6.0185	16.1277	0.3899	0.5611
A	1315.9255	26.1166	32.9442	0.0968	0.1765
A	1304.8618	68.0218	15.4988	0.7477	0.8556
A	1299.4857	121.6044	23.2786	0.5400	0.7013

A	1271.8223	7.5471	8.0256	0.7080	0.8290
A	1258.0042	5.8136	36.2366	0.6080	0.7562
A	1250.6548	56.4057	16.8965	0.7482	0.8560
A	1248.9949	6.3463	19.3569	0.3711	0.5413
A	1244.0658	38.5109	45.2583	0.3368	0.5039
A	1241.8030	32.4258	16.3052	0.7234	0.8395
A	1221.9969	8.7761	7.2273	0.7278	0.8424
A	1216.7582	0.2990	17.1899	0.3370	0.5041
A	1215.0771	24.2216	16.1637	0.5500	0.7097
A	1197.7299	337.5293	9.6851	0.3129	0.4767
A	1197.1612	3.9721	6.4549	0.6240	0.7684
A	1185.2845	30.5186	11.3030	0.3033	0.4654
A	1150.6434	12.9569	14.2339	0.7490	0.8565
A	1148.2634	434.2277	2.4613	0.3583	0.5276
A	1141.8813	29.6935	16.2933	0.7165	0.8348
A	1133.5521	12.2542	8.2458	0.6217	0.7668
A	1116.1364	307.5184	6.7870	0.4021	0.5736
A	1116.0035	153.6641	7.7999	0.2111	0.3486
A	1109.9621	2.9956	24.2921	0.2815	0.4394
A	1102.9381	40.0751	5.9372	0.7069	0.8283
A	1095.9212	12.1531	17.0146	0.2594	0.4120
A	1076.4789	20.6164	16.4260	0.3810	0.5518
A	1066.9389	9.0192	4.5878	0.2740	0.4301
A	1054.5487	4.5798	16.0047	0.4095	0.5810
A	1053.4870	48.0064	10.5510	0.5790	0.7333
A	1042.7471	1.3617	2.8392	0.6424	0.7823
A	1040.6721	7.8420	10.4847	0.6887	0.8157
A	1023.6641	1.7798	21.1409	0.1370	0.2410
A	1015.0168	6.3268	6.8293	0.2721	0.4278
A	1004.5443	1.9692	10.0275	0.2892	0.4486
A	996.1220	9.8345	9.8277	0.6515	0.7890
A	986.6670	6.5993	7.5354	0.2119	0.3497
A	975.3189	4.6414	18.7683	0.6715	0.8035
A	974.5708	4.7716	6.3221	0.7104	0.8307
A	969.8198	0.2935	0.2626	0.6919	0.8179
A	964.8046	19.3116	12.2177	0.5030	0.6693
A	950.1690	9.4978	9.9881	0.3943	0.5656
A	948.0005	14.3477	8.2070	0.4967	0.6637
A	947.1806	5.9113	10.9096	0.6700	0.8024
A	924.1071	20.0596	30.0655	0.1759	0.2991
A	902.5690	7.4259	28.6712	0.3902	0.5613
A	891.3913	41.3683	5.3479	0.2217	0.3629
A	886.5283	2.4369	23.2280	0.2731	0.4291
A	848.2036	18.1829	27.5739	0.0982	0.1788
A	845.2969	8.1730	4.9212	0.1922	0.3225
A	831.9147	19.7177	7.7911	0.5089	0.6746
A	822.6018	4.1700	10.3512	0.6408	0.7811
A	810.2017	0.6119	7.6723	0.1604	0.2764
A	792.4829	7.3810	2.1892	0.5454	0.7058
A	767.9368	22.2857	3.9983	0.4959	0.6630
A	762.3450	20.5946	9.6125	0.3095	0.4727

A	746.9025	24.5127	4.9430	0.5465	0.7068
A	713.8753	36.0729	10.5729	0.2297	0.3737
A	711.9150	6.8280	2.6226	0.4282	0.5997
A	704.6181	8.5264	27.4873	0.0208	0.0408
A	697.2962	8.0451	5.4172	0.4048	0.5763
A	691.5621	25.2763	6.1581	0.7351	0.8474
A	676.4738	41.2635	10.9991	0.5407	0.7019
A	642.5678	147.0053	4.1766	0.6097	0.7576
A	608.4936	37.1533	3.7520	0.5236	0.6873
A	602.3689	49.0026	7.9688	0.1805	0.3058
A	600.5629	158.9727	1.1270	0.0880	0.1618
A	586.1587	78.5550	4.3082	0.6997	0.8233
A	563.8278	13.4480	3.0281	0.4446	0.6156
A	546.7016	91.1052	8.1857	0.1523	0.2643
A	541.1450	6.6787	4.3964	0.3658	0.5357
A	531.8060	96.6064	4.0947	0.4676	0.6372
A	507.7606	73.9201	2.4154	0.7498	0.8570
A	502.2816	33.1473	1.6911	0.7329	0.8458
A	472.3374	5.6104	2.9418	0.0734	0.1368
A	457.5185	5.6737	0.3451	0.3894	0.5606
A	451.9965	19.6980	1.2061	0.5615	0.7192
A	422.8773	15.3327	1.3192	0.6076	0.7559
A	416.3307	9.1054	2.1581	0.4815	0.6500
A	407.3508	5.6215	2.6069	0.6139	0.7608
A	399.7729	6.2334	1.0787	0.5041	0.6703
A	388.2221	5.3805	1.6886	0.7409	0.8512
A	365.4714	5.7136	0.2425	0.5978	0.7483
A	361.3420	28.5900	0.8315	0.4708	0.6402
A	353.1771	39.6302	2.0006	0.7348	0.8471
A	342.6163	2.8218	1.6325	0.7241	0.8400
A	328.4215	0.8402	0.7036	0.4966	0.6636
A	319.0688	8.8024	0.9349	0.6787	0.8086
A	308.3834	10.5296	2.3345	0.3681	0.5381
A	304.6335	5.9629	1.7412	0.5075	0.6733
A	294.2837	6.0942	0.6029	0.7287	0.8431
A	290.0620	4.1891	2.6277	0.5291	0.6920
A	281.6886	1.6555	0.8858	0.5644	0.7215
A	264.9694	2.7654	1.7477	0.7309	0.8446
A	254.1108	7.1385	1.7360	0.0392	0.0755
A	252.9113	2.1168	2.9655	0.3038	0.4660
A	244.9035	7.2370	0.9088	0.4448	0.6158
A	234.4345	21.3056	3.5350	0.6612	0.7960
A	220.9837	3.2151	2.4691	0.1600	0.2758
A	208.9606	6.4779	1.0214	0.2896	0.4491
A	201.2147	2.7290	2.4477	0.2512	0.4015
A	191.3648	5.1470	4.2139	0.6459	0.7849
A	181.5208	5.5544	2.3538	0.2724	0.4281
A	175.4097	11.5787	1.8853	0.5816	0.7355
A	159.0533	1.0036	0.4685	0.7406	0.8509
A	148.1375	10.3491	1.1063	0.7432	0.8527
A	138.9856	3.6563	1.2720	0.7498	0.8570

A	131.9360	9.7883	3.7809	0.6334	0.7756
A	115.4323	7.1003	1.8353	0.7326	0.8456
A	104.8808	10.8744	0.7548	0.7495	0.8568
A	98.8953	1.3744	0.6898	0.5935	0.7449
A	91.5589	0.8534	0.2033	0.6815	0.8106
A	84.7741	2.8567	2.3800	0.7435	0.8529
A	76.0821	13.1148	1.4636	0.7246	0.8403
A	70.0241	1.8114	1.2244	0.7438	0.8531
A	57.8881	1.9515	0.5250	0.7489	0.8564
A	44.6701	3.9536	1.8669	0.7499	0.8571
A	42.0089	1.7529	1.3516	0.7491	0.8566
A	39.3610	10.7789	1.7218	0.7500	0.8571
A	35.5825	11.4936	2.0760	0.7499	0.8571
A	32.6914	0.7492	0.3242	0.7499	0.8571
A	26.7804	6.7239	0.7259	0.7499	0.8571
A	21.5832	0.5824	0.6079	0.7499	0.8571
A	19.7180	0.9747	1.1806	0.7490	0.8565

67

C	-4.609976	-1.841928	-1.004866
C	-4.679418	-0.573565	-0.154543
C	-5.699386	-0.942388	0.940462
C	-6.573721	-2.025402	0.287730
N	-5.656073	-2.624524	-0.685344
C	-3.290781	-0.132877	0.300792
C	-3.238409	1.285051	0.899746
C	-3.941687	1.384054	2.189039
N	-4.483057	1.460041	3.215180
O	-3.743861	-2.100803	-1.861620
N	-1.860809	1.728389	1.086293
C	-1.245425	2.507692	0.165238
O	-1.843133	3.085949	-0.753314
C	0.278681	2.599286	0.344794
C	0.848308	3.857975	-0.267148
C	1.635529	3.466658	-1.503175
C	1.549716	1.964505	-1.663998
N	0.906778	1.491654	-0.413452
C	2.351394	4.043459	-0.299368
C	2.812210	5.483379	-0.449901
C	3.289693	3.208825	0.554787
C	0.982361	0.265690	0.133882
C	1.659792	-0.855225	-0.655921
C	0.644544	-1.802570	-1.385120
C	-0.200597	-0.966290	-2.358955
O	0.512546	0.018973	1.266592
N	2.501856	-1.580259	0.292648
C	3.787915	-1.841954	0.069633
C	4.506684	-2.626721	1.192592
F	4.960128	-3.805505	0.714815
C	1.457431	-2.832461	-2.187497
C	-0.274899	-2.536157	-0.396952

O	4.455961	-1.531641	-0.921642
F	5.573123	-1.928824	1.636005
F	3.720995	-2.894125	2.260126
H	2.122225	6.062728	-1.071709
H	3.804131	5.522849	-0.915555
H	2.879996	5.972202	0.529294
H	2.974701	2.170653	0.672851
H	4.291685	3.203695	0.110180
H	3.373831	3.647579	1.555846
H	1.577495	4.058571	-2.410605
H	0.212647	4.735894	-0.258620
H	0.528547	2.478614	1.401983
H	2.528549	1.493722	-1.776586
H	0.934114	1.708863	-2.530032
H	2.324773	-0.446225	-1.412813
H	2.069413	-1.885462	1.157839
H	-0.894183	-1.622166	-2.893294
H	-0.793685	-0.206916	-1.841028
H	0.432225	-0.466837	-3.099411
H	0.774093	-3.474283	-2.752865
H	2.129464	-2.338968	-2.897301
H	2.055332	-3.475861	-1.534677
H	-0.930528	-3.212683	-0.954283
H	0.295905	-3.140376	0.316065
H	-0.905449	-1.844763	0.164640
H	-1.252122	1.122635	1.634877
H	-3.719594	1.988984	0.214439
H	-2.624766	-0.115389	-0.565223
H	-2.879978	-0.846421	1.022296
H	-5.171489	-1.363173	1.802596
H	-6.291381	-0.092187	1.280390
H	-7.442022	-1.594623	-0.223391
H	-6.925709	-2.774257	0.999305
H	-5.879990	-3.451845	-1.225039
H	-5.099154	0.200359	-0.813615

Conformer **C**: B3LYPD3BJ-SMD/6-31+G(d,p), in acetonitrile solvent

Processing: nirb6pbed3jacs.log
PG=C01

Method	BasisSet	Imaginary Freqs
RB3LYP	6-31+G(d,p)	0

HF Energy
-1770.2426031

ZPE	E298	S298	Squasihar	Equasihar	Strans	Srot
343.62496	366.173	227.600	204.250	366.281	44.511	37.641

Label Frequencies IR Inten Raman Activ Depolar (P) Depolar (U) Dipole

A	3621.0826	95.6603	343.9299	0.2123	0.3503	4.9249
A	3600.6758	174.9457	137.3673	0.0888	0.1632	
A	3455.4984	386.7800	235.8222	0.1283	0.2274	
A	3211.5142	7.2367	354.9127	0.1637	0.2813	
A	3185.5021	21.9519	367.1649	0.2943	0.4547	
A	3159.8945	2.8459	171.1046	0.1186	0.2121	
A	3144.7559	32.3207	174.6732	0.5152	0.6800	
A	3144.1005	29.0733	191.2641	0.4666	0.6363	
A	3142.3268	36.3664	215.1841	0.6309	0.7737	
A	3129.5979	20.1542	221.6214	0.5040	0.6702	
A	3127.3106	24.7117	182.9071	0.7427	0.8524	
A	3122.3113	37.7111	136.7974	0.6920	0.8180	
A	3115.7383	84.1316	331.0420	0.7429	0.8525	
A	3115.3289	61.9817	233.9852	0.7465	0.8549	
A	3112.4187	43.3282	304.6587	0.3758	0.5463	
A	3109.5031	36.5903	165.6133	0.7323	0.8454	
A	3106.0900	20.5718	84.6048	0.7499	0.8571	
A	3105.2525	50.3488	194.1335	0.7491	0.8566	
A	3098.0441	29.3796	111.3465	0.1934	0.3242	
A	3088.2681	98.4690	447.2634	0.7498	0.8570	
A	3086.3782	11.1069	59.0318	0.7419	0.8519	
A	3080.5420	2.3629	429.4957	0.0905	0.1660	
A	3076.9782	57.5292	276.2815	0.0431	0.0826	
A	3069.7219	41.1417	587.1342	0.0427	0.0819	
A	3066.1467	20.3469	42.2674	0.6555	0.7919	
A	3049.6414	19.9428	1126.2135	0.0067	0.0133	
A	3043.7007	54.9781	15.1850	0.6990	0.8228	
A	3038.9977	82.1755	429.0322	0.1535	0.2661	
A	3038.2721	49.9355	105.3587	0.0145	0.0285	
A	3036.0549	80.2591	647.2764	0.0302	0.0585	
A	3025.0833	60.7932	306.6214	0.0070	0.0139	
A	3024.4317	16.6548	420.5276	0.1841	0.3109	
A	2347.8212	49.4498	374.4412	0.1629	0.2801	
A	1753.6387	648.7105	30.1766	0.1969	0.3291	
A	1726.6084	958.7524	33.3234	0.3037	0.4659	
A	1718.9554	572.2164	17.7594	0.5598	0.7178	
A	1649.1521	514.6982	39.4916	0.2240	0.3660	
A	1576.5331	238.8561	18.3418	0.1200	0.2142	
A	1558.4992	629.5984	6.4509	0.6573	0.7932	
A	1519.1658	14.2486	23.4142	0.7117	0.8315	
A	1508.3341	33.9414	3.9416	0.5553	0.7141	
A	1503.4710	5.8074	17.6974	0.4351	0.6063	
A	1500.4562	17.1842	16.4149	0.6663	0.7998	
A	1499.7052	1.3034	24.8603	0.7481	0.8559	
A	1494.6315	7.7297	35.1800	0.7318	0.8451	
A	1487.2859	10.1831	3.8731	0.6892	0.8160	
A	1487.0466	24.8908	2.4936	0.7114	0.8314	
A	1485.7526	2.2714	24.3324	0.7493	0.8567	
A	1484.6730	3.0197	36.5668	0.7485	0.8562	
A	1482.2768	64.5163	4.4200	0.3256	0.4912	
A	1480.1569	2.2831	21.2069	0.7008	0.8241	

A	1477.1168	111.4757	13.3588	0.5092	0.6748
A	1474.6430	188.4046	12.5491	0.1048	0.1897
A	1473.0879	5.1638	31.4018	0.7448	0.8537
A	1457.7703	70.0818	9.6844	0.4353	0.6066
A	1443.7425	14.9927	56.2823	0.2478	0.3972
A	1426.3116	8.4849	2.8867	0.0965	0.1760
A	1419.8349	3.3564	7.7325	0.5082	0.6739
A	1411.0288	5.5935	3.1212	0.4602	0.6303
A	1405.5120	20.3215	1.8201	0.4325	0.6038
A	1401.1306	6.2741	0.6868	0.1401	0.2457
A	1395.1923	16.2614	27.8680	0.7375	0.8489
A	1381.4649	54.9095	29.4701	0.7404	0.8508
A	1378.9420	15.8388	7.7352	0.6507	0.7884
A	1373.6522	14.2854	7.7826	0.5624	0.7199
A	1372.4648	14.4706	10.1639	0.5003	0.6669
A	1360.9957	5.7910	7.0962	0.6184	0.7642
A	1344.1022	37.7957	9.5610	0.7499	0.8571
A	1342.7811	4.3685	10.8648	0.4671	0.6367
A	1331.0035	38.3815	10.1609	0.7199	0.8372
A	1323.7025	31.7522	16.1573	0.7471	0.8552
A	1317.7499	18.8410	10.0780	0.7182	0.8360
A	1315.3425	1.2851	12.8676	0.4004	0.5718
A	1309.6231	47.0855	21.1110	0.1234	0.2198
A	1302.0218	46.3351	17.4973	0.6405	0.7808
A	1290.1770	184.8464	21.4031	0.5798	0.7340
A	1271.9124	6.4485	8.4360	0.7344	0.8468
A	1257.1944	6.1250	34.6577	0.6638	0.7979
A	1249.6608	6.6374	18.9239	0.6075	0.7558
A	1245.6576	17.9033	7.5020	0.4181	0.5897
A	1243.9974	51.0954	48.0131	0.4076	0.5791
A	1238.8464	49.7069	13.0917	0.6879	0.8151
A	1220.2421	7.1745	6.1810	0.6417	0.7818
A	1216.8062	1.9168	17.3661	0.3989	0.5703
A	1214.3757	20.7353	15.2843	0.5729	0.7284
A	1197.2126	350.6945	6.2836	0.3282	0.4942
A	1194.8447	3.5551	5.3528	0.6528	0.7900
A	1185.1988	28.1593	6.7296	0.3890	0.5601
A	1150.8976	14.4113	16.1310	0.7500	0.8571
A	1144.6725	342.9917	1.8659	0.2820	0.4399
A	1140.9295	30.4323	15.7326	0.6792	0.8090
A	1131.0795	7.4737	9.7441	0.6071	0.7555
A	1116.8056	4.7342	14.5787	0.1441	0.2519
A	1114.0850	436.6284	4.4590	0.7416	0.8516
A	1110.0945	4.8635	22.8877	0.3264	0.4921
A	1103.4562	72.2428	4.7902	0.7164	0.8347
A	1094.9945	6.5808	11.3594	0.3497	0.5182
A	1073.9559	10.4093	15.4724	0.3350	0.5019
A	1067.3292	10.4459	3.8395	0.4890	0.6569
A	1060.1901	58.2942	8.0847	0.7121	0.8318
A	1053.7885	2.6493	14.3777	0.4128	0.5843
A	1042.0775	1.0456	3.5476	0.6702	0.8025

A	1040.6037	7.2564	8.4816	0.6904	0.8168
A	1022.6995	2.9206	18.0452	0.1945	0.3256
A	1011.3031	5.8329	7.0195	0.2395	0.3865
A	1003.8420	0.9822	7.9480	0.2740	0.4301
A	993.8326	11.5170	8.4939	0.6931	0.8188
A	981.6397	6.8689	8.4598	0.1486	0.2588
A	973.9891	4.1732	4.0664	0.7427	0.8523
A	973.3143	3.3425	19.9824	0.7076	0.8287
A	969.4797	0.2927	0.2573	0.7273	0.8421
A	963.3616	16.1808	9.2555	0.4780	0.6468
A	949.2763	9.4511	8.8586	0.4688	0.6383
A	947.7207	2.4674	10.4798	0.5364	0.6982
A	944.0971	13.2423	9.8878	0.5390	0.7004
A	924.5712	18.5848	25.6007	0.2114	0.3490
A	901.7181	5.8475	27.6537	0.3571	0.5263
A	890.9228	38.1305	5.1530	0.2667	0.4211
A	884.2518	1.4209	17.7440	0.2636	0.4172
A	845.7411	9.2635	14.6251	0.0687	0.1285
A	843.8210	10.1299	15.1157	0.1264	0.2245
A	829.1341	14.8083	6.3383	0.6521	0.7894
A	819.8569	3.1027	9.4931	0.6814	0.8105
A	808.6042	0.4468	7.5752	0.1416	0.2481
A	792.6242	5.3130	1.2009	0.7402	0.8507
A	766.3560	28.7597	4.9306	0.3529	0.5217
A	759.8595	14.6798	7.2456	0.2169	0.3564
A	740.0164	6.6604	5.1299	0.6713	0.8033
A	713.5248	50.7557	2.9962	0.2914	0.4513
A	710.6312	6.1801	7.2600	0.2647	0.4186
A	703.5980	46.4310	20.4215	0.0235	0.0458
A	697.7246	36.8613	10.6610	0.0615	0.1159
A	690.5976	29.8030	5.4026	0.6842	0.8125
A	682.2134	55.8206	14.2388	0.5664	0.7232
A	637.9226	61.8416	1.9355	0.7495	0.8568
A	612.5228	30.7948	3.2862	0.5826	0.7363
A	602.5726	45.0902	3.6321	0.0954	0.1741
A	594.7041	17.6814	5.3818	0.2851	0.4437
A	569.1036	172.9719	0.7775	0.5941	0.7454
A	563.5787	11.8495	0.3222	0.6226	0.7674
A	549.8306	43.4773	7.1636	0.2405	0.3878
A	536.9505	18.1743	4.1698	0.4851	0.6533
A	530.9632	97.6413	3.4197	0.3387	0.5060
A	509.3056	71.2531	2.0689	0.7396	0.8503
A	501.4701	47.0854	1.8160	0.7148	0.8337
A	477.3224	5.3776	3.0096	0.0830	0.1533
A	456.7625	10.1154	0.3244	0.2821	0.4401
A	451.4104	17.8052	0.7435	0.7480	0.8558
A	425.5503	18.6251	1.3563	0.5870	0.7398
A	415.3840	9.2080	1.8168	0.4647	0.6345
A	405.1812	6.8561	2.3037	0.6103	0.7580
A	401.8434	7.9666	1.3649	0.5249	0.6884
A	392.9499	1.7966	1.2575	0.7457	0.8543

A	365.5881	30.8969	0.8718	0.5970	0.7476
A	365.3769	9.0683	0.5917	0.4425	0.6135
A	354.6259	40.2118	1.8364	0.7246	0.8403
A	339.9078	4.6448	1.5291	0.6177	0.7637
A	327.2028	1.4531	0.5432	0.4713	0.6406
A	319.6655	7.5943	0.9867	0.7500	0.8571
A	309.6595	9.4750	2.9077	0.2656	0.4197
A	302.5126	8.4653	1.2872	0.4655	0.6353
A	292.9508	3.4664	0.2626	0.7399	0.8505
A	288.2018	5.3441	2.2804	0.5260	0.6894
A	279.8146	1.6033	0.8340	0.4808	0.6494
A	266.1343	2.5216	1.4790	0.7358	0.8478
A	254.4110	5.4798	2.0409	0.0793	0.1469
A	252.0357	3.8541	2.3970	0.3242	0.4896
A	239.7835	7.9032	1.1120	0.6341	0.7761
A	236.4422	16.9674	2.9477	0.6258	0.7699
A	223.1457	2.3007	1.2721	0.2921	0.4522
A	213.6033	5.7485	1.7363	0.1638	0.2815
A	194.0978	8.8056	4.1888	0.4004	0.5718
A	193.2325	0.5937	2.7980	0.3322	0.4987
A	183.7055	7.5907	1.7776	0.5627	0.7202
A	177.3156	8.3303	1.5517	0.3107	0.4741
A	162.8210	4.3928	0.7848	0.6784	0.8084
A	151.7026	8.9290	0.4451	0.6965	0.8211
A	140.7430	4.1778	0.6456	0.6768	0.8072
A	133.0392	4.4237	4.2390	0.5903	0.7424
A	112.8701	3.2799	0.9979	0.7408	0.8511
A	106.2792	11.5885	1.3889	0.7486	0.8563
A	97.3731	0.0013	0.3076	0.5681	0.7246
A	83.8047	2.5798	1.6761	0.7443	0.8534
A	79.9762	5.5527	0.9978	0.7488	0.8564
A	79.4901	1.3226	0.1972	0.7494	0.8567
A	67.6373	3.0636	1.3958	0.7435	0.8529
A	51.9934	1.8124	1.5197	0.7294	0.8435
A	44.1858	5.5722	1.9593	0.7495	0.8568
A	39.4855	2.7395	0.3645	0.7491	0.8566
A	35.4096	1.6532	1.0137	0.7500	0.8571
A	31.8630	6.9193	0.7902	0.7495	0.8568
A	28.5702	5.8224	0.2986	0.7379	0.8492
A	27.2174	6.4913	1.0512	0.7499	0.8571
A	22.6140	0.1471	0.7937	0.7500	0.8571
A	11.3899	0.5175	0.9483	0.7484	0.8561

67

C	-5.023778	-1.548915	-1.144918
C	-4.876927	-0.462312	-0.070476
C	-5.710122	-1.011827	1.104635
C	-6.744075	-1.935575	0.434251
N	-6.045251	-2.356554	-0.779304
C	-3.407107	-0.146622	0.199894
C	-3.162223	1.171698	0.959456

C	-3.704840	1.151787	2.331813
N	-4.119070	1.128627	3.416150
O	-4.343605	-1.656531	-2.170548
N	-1.746075	1.495548	1.027866
C	-1.202351	2.503088	0.298297
O	-1.866859	3.297083	-0.369878
C	0.339293	2.558150	0.383229
C	0.892796	3.821477	-0.233346
C	1.629021	3.444269	-1.504483
C	1.530559	1.944131	-1.680599
N	0.933818	1.456595	-0.413344
C	2.393512	4.006935	-0.323320
C	2.850011	5.448696	-0.476624
C	3.365886	3.162055	0.481656
C	1.053551	0.227380	0.123929
C	1.709323	-0.887525	-0.694411
C	0.667223	-1.825644	-1.399361
C	-0.213620	-0.984380	-2.336393
O	0.631916	-0.036629	1.268727
N	2.562494	-1.625670	0.231539
C	3.862386	-1.841648	0.009106
C	4.595277	-2.619055	1.131934
F	5.054457	-3.798334	0.657058
C	1.450918	-2.852434	-2.234228
C	-0.218524	-2.563703	-0.383291
O	4.518761	-1.511207	-0.975198
F	5.661003	-1.915399	1.569384
F	3.819571	-2.890983	2.208301
H	2.135533	6.036189	-1.062642
H	3.822063	5.494060	-0.982812
H	2.958632	5.927201	0.504422
H	3.053269	2.123387	0.601165
H	4.349200	3.159221	-0.003799
H	3.492824	3.588600	1.483894
H	1.535622	4.047866	-2.401686
H	0.256195	4.697180	-0.195679
H	0.635412	2.416888	1.427454
H	2.502129	1.470691	-1.837843
H	0.881058	1.702898	-2.526093
H	2.360815	-0.480973	-1.465254
H	2.149717	-1.899874	1.115975
H	-0.918241	-1.639429	-2.858271
H	-0.797757	-0.237883	-1.790121
H	0.389894	-0.469781	-3.090901
H	0.749763	-3.502199	-2.768577
H	2.088524	-2.356888	-2.974189
H	2.081491	-3.488047	-1.604560
H	-0.909622	-3.221250	-0.921298
H	0.374898	-3.188762	0.292618
H	-0.809872	-1.872053	0.220354
H	-1.105741	0.819511	1.446332

H	-3.655652	1.992355	0.429011
H	-2.888574	-0.039718	-0.756831
H	-2.934394	-0.972253	0.742397
H	-5.063193	-1.599838	1.764732
H	-6.188029	-0.232136	1.698806
H	-7.665516	-1.396418	0.180672
H	-7.004545	-2.798427	1.051775
H	-6.409367	-3.061499	-1.408868
H	-5.362057	0.432112	-0.488531

Conformer **D**: B3LYPD3BJ/6-31G(d)

Processing: nir-ba6dbed3j.log
PG=C01

Method	BasisSet	Imaginary Freqs
RB3LYP	6-31G(d)	0

HF Energy
-1770.0740324

ZPE	E298	S298	Squasihar	Equasihar	Strans	Srot
346.84519	369.237	221.468	203.512	369.334	44.511	36.969

Population analysis using the SCF Density.

Label	Frequencies	IR Inten	Raman Activ	Depolar (P)	Depolar (U)	Dipole
A	3633.7445	33.4786	137.5346	0.2759	0.4325	4.6564
A	3610.1418	24.4486	49.0967	0.1548	0.2681	
A	3589.6233	68.1754	33.4355	0.1040	0.1884	
A	3195.3451	11.2671	106.7635	0.0999	0.1817	
A	3187.6993	8.5347	62.1391	0.6459	0.7849	
A	3178.5835	8.7798	133.6299	0.3417	0.5094	
A	3166.1823	8.3140	79.0351	0.4880	0.6559	
A	3157.0758	12.2114	35.8056	0.7144	0.8334	
A	3138.6343	33.0075	46.3240	0.7152	0.8339	
A	3138.1786	17.0314	58.6698	0.6791	0.8089	
A	3136.1254	8.4913	32.1538	0.1638	0.2815	
A	3124.4781	44.2253	82.2303	0.6987	0.8226	
A	3119.3999	48.5723	90.9316	0.6377	0.7788	
A	3115.8437	9.6080	19.4968	0.6940	0.8194	
A	3114.7247	26.1448	61.8338	0.7003	0.8237	
A	3113.0929	23.1454	37.8299	0.1073	0.1938	
A	3107.6981	3.1733	39.6593	0.6977	0.8219	
A	3107.5625	31.1582	68.1173	0.4776	0.6465	
A	3104.5382	33.6572	120.7291	0.7280	0.8426	
A	3098.5140	6.5955	29.6385	0.6975	0.8218	
A	3097.7714	29.6875	98.8518	0.1851	0.3124	
A	3087.2446	14.5543	98.9701	0.3179	0.4824	
A	3068.0886	18.8774	93.4800	0.0607	0.1144	

A	3059.3342	26.5818	263.9674	0.0422	0.0810
A	3058.9251	25.2996	60.8887	0.1384	0.2431
A	3052.5873	17.6848	22.4642	0.0787	0.1459
A	3048.5458	49.7747	205.9318	0.0440	0.0842
A	3042.9502	20.0078	28.5609	0.0499	0.0950
A	3040.8068	29.1196	69.9534	0.0344	0.0665
A	3039.2052	24.0727	17.0952	0.5957	0.7467
A	3036.0956	15.6167	72.6805	0.3241	0.4896
A	3026.8842	84.5127	187.9372	0.2379	0.3844
A	2369.2409	7.4741	118.6084	0.2372	0.3834
A	1812.0491	398.3928	6.9265	0.4388	0.6100
A	1787.5790	290.1596	11.0284	0.1654	0.2838
A	1779.3382	188.3408	9.4692	0.0676	0.1266
A	1744.5406	236.5240	9.3348	0.4042	0.5757
A	1575.0267	176.0662	3.3183	0.3447	0.5127
A	1560.4142	4.7215	19.7254	0.5946	0.7457
A	1549.6644	13.5990	2.1175	0.7475	0.8555
A	1546.1233	222.3427	7.5596	0.7306	0.8443
A	1543.9678	14.9629	4.0023	0.4010	0.5724
A	1541.2437	6.8762	24.8858	0.7495	0.8568
A	1539.8742	3.9043	11.4627	0.7500	0.8571
A	1535.4209	4.2472	16.3660	0.6698	0.8022
A	1534.6401	5.7326	7.4889	0.6991	0.8229
A	1533.6825	5.8479	27.9836	0.7424	0.8522
A	1521.3454	5.5257	0.6572	0.4499	0.6206
A	1519.2721	3.2479	20.7418	0.7411	0.8513
A	1517.2753	12.3647	4.7878	0.7469	0.8551
A	1516.2827	0.8009	22.1973	0.7490	0.8565
A	1514.1443	0.2413	3.6517	0.7454	0.8541
A	1507.7586	1.7785	12.9562	0.7462	0.8547
A	1473.7056	52.7915	2.6640	0.4048	0.5763
A	1466.1790	46.6268	2.4851	0.7469	0.8551
A	1462.0609	6.9827	1.3676	0.7500	0.8571
A	1452.3284	142.2251	4.6044	0.4681	0.6377
A	1445.8598	45.4200	18.5977	0.1609	0.2773
A	1438.6140	4.4007	6.7994	0.6687	0.8015
A	1431.4540	19.1305	2.7886	0.6948	0.8200
A	1430.0355	4.5570	2.0973	0.7353	0.8474
A	1426.8172	5.2899	1.2680	0.4684	0.6380
A	1410.5212	2.0189	13.5684	0.5867	0.7395
A	1394.8875	1.5737	8.4891	0.3629	0.5325
A	1393.9078	50.8694	3.1740	0.6817	0.8107
A	1390.4357	8.0627	4.9763	0.7432	0.8527
A	1370.7242	8.2062	4.7559	0.7493	0.8567
A	1363.3840	13.6099	2.0548	0.3775	0.5481
A	1355.7917	42.4134	2.9532	0.4368	0.6080
A	1352.5137	48.0702	2.0982	0.6213	0.7664
A	1343.3918	24.9601	2.9415	0.5879	0.7405
A	1339.6246	20.8099	4.0698	0.2769	0.4338
A	1329.9530	74.9691	13.3359	0.7164	0.8347
A	1308.4495	62.3424	7.9297	0.2133	0.3516

A	1302.2519	13.8082	6.7625	0.5716	0.7274
A	1290.1808	84.1243	1.7248	0.2703	0.4255
A	1284.5252	6.0914	5.4325	0.5794	0.7337
A	1277.7001	14.6834	3.6011	0.2806	0.4382
A	1263.9734	1.3162	1.2559	0.7052	0.8271
A	1251.1144	15.1093	6.1146	0.7031	0.8256
A	1246.1288	3.3841	11.2636	0.5328	0.6952
A	1242.8856	182.3002	0.7456	0.7156	0.8342
A	1241.5767	116.3228	2.0081	0.7500	0.8571
A	1237.8410	32.0347	2.4396	0.5518	0.7112
A	1233.5955	4.1634	7.1451	0.6071	0.7555
A	1226.9925	15.8856	3.9308	0.7390	0.8499
A	1223.8764	19.4638	8.1111	0.6633	0.7975
A	1220.6283	37.2374	3.7222	0.4057	0.5772
A	1214.9113	176.9747	0.5398	0.5930	0.7445
A	1192.4105	162.5277	2.8322	0.3438	0.5116
A	1171.3745	4.6875	2.5562	0.5786	0.7331
A	1144.3109	14.8203	2.7452	0.3190	0.4837
A	1142.1842	0.9031	5.9225	0.5330	0.6953
A	1124.4632	0.9967	0.4722	0.6183	0.7641
A	1122.6252	1.9510	2.3468	0.6393	0.7799
A	1121.0481	3.1738	6.2502	0.6012	0.7510
A	1096.4875	11.5154	2.9061	0.2464	0.3954
A	1087.5494	18.8317	7.5592	0.3372	0.5043
A	1075.8078	1.9414	6.4621	0.5677	0.7242
A	1067.9288	6.9310	2.0144	0.1286	0.2278
A	1065.4072	0.6722	1.7103	0.7146	0.8336
A	1060.6848	3.1560	8.0916	0.6915	0.8176
A	1056.7764	2.1202	3.8001	0.7367	0.8484
A	1049.8206	2.2815	1.0253	0.5192	0.6835
A	1033.2550	0.1496	8.6449	0.4107	0.5823
A	1012.6655	1.0257	2.3343	0.5248	0.6884
A	1009.4699	4.0290	4.6286	0.5983	0.7487
A	991.3398	4.8232	4.1809	0.2369	0.3831
A	984.1565	3.6595	5.1645	0.7057	0.8274
A	981.1969	1.1043	4.0712	0.7418	0.8518
A	979.2057	4.1142	5.0776	0.7497	0.8570
A	959.5994	6.6268	4.0528	0.3691	0.5392
A	954.7947	1.7042	4.1324	0.6154	0.7619
A	954.3546	0.7451	5.0487	0.6059	0.7546
A	951.6523	1.4362	6.3120	0.1338	0.2361
A	940.9762	10.4513	10.0943	0.1287	0.2281
A	913.3931	19.3930	18.2916	0.5243	0.6879
A	907.0928	2.3605	2.9745	0.2878	0.4470
A	901.6313	1.9397	4.0554	0.3914	0.5626
A	870.8382	10.8582	2.7534	0.7495	0.8568
A	852.6658	1.1647	4.7425	0.2757	0.4323
A	829.0243	6.1383	6.6080	0.7493	0.8567
A	813.9964	2.6394	1.9461	0.6501	0.7879
A	810.3807	8.6414	1.1343	0.3766	0.5472
A	790.3235	6.1042	6.1331	0.2485	0.3981

A	774.6827	6.8941	0.2386	0.5273	0.6905
A	768.0230	9.7346	4.1032	0.0545	0.1033
A	735.3325	15.0883	1.5991	0.7327	0.8457
A	725.9937	15.9161	6.1271	0.0838	0.1546
A	719.7847	4.1601	0.4715	0.7121	0.8318
A	716.7475	39.4886	3.8226	0.1048	0.1897
A	703.9247	10.1269	3.5621	0.4701	0.6395
A	687.8931	4.4944	7.8436	0.2941	0.4545
A	660.8405	62.9300	0.9211	0.4864	0.6545
A	659.4421	28.9736	4.7680	0.2008	0.3345
A	630.2255	3.8808	2.0666	0.5638	0.7211
A	612.8929	15.6270	3.3431	0.1523	0.2643
A	595.0710	11.3502	1.4664	0.7498	0.8570
A	561.4737	0.3323	2.0114	0.7361	0.8480
A	558.9238	1.1404	0.3063	0.4769	0.6458
A	545.7200	1.8141	1.3084	0.1471	0.2564
A	529.4743	98.9281	3.2260	0.7129	0.8324
A	509.3175	14.7277	0.5634	0.7408	0.8511
A	482.7623	18.6183	1.9749	0.4624	0.6324
A	466.6729	7.4479	1.0499	0.3182	0.4827
A	462.2579	15.2326	0.2665	0.5445	0.7051
A	450.4588	5.3841	0.3741	0.5412	0.7023
A	439.6407	74.5594	0.8435	0.6901	0.8166
A	426.7674	9.5063	2.2023	0.0523	0.0994
A	421.0824	1.3196	0.7807	0.6760	0.8067
A	406.5459	8.7483	0.7737	0.7470	0.8552
A	404.4624	15.1921	0.5670	0.6033	0.7525
A	392.0825	3.9806	0.3598	0.2758	0.4324
A	373.4334	7.0134	0.9448	0.6742	0.8054
A	361.9206	3.2973	1.2744	0.6264	0.7703
A	342.5255	3.3554	1.0887	0.7500	0.8571
A	330.7915	12.1764	0.5217	0.6483	0.7867
A	317.7725	0.7710	0.7129	0.3809	0.5516
A	313.6445	3.9616	0.9239	0.3661	0.5360
A	300.1459	10.1091	0.4035	0.6559	0.7922
A	295.0149	7.0315	0.6724	0.4168	0.5884
A	290.7457	3.4403	1.1967	0.7441	0.8533
A	285.7263	0.7518	0.4313	0.4374	0.6086
A	281.1106	0.3052	1.1420	0.5051	0.6712
A	269.0752	1.5594	0.8742	0.7410	0.8512
A	254.0789	0.8125	0.1616	0.7186	0.8362
A	240.8072	5.1133	2.2348	0.0348	0.0673
A	238.1436	4.7235	0.9821	0.3821	0.5529
A	234.1848	6.8725	0.8992	0.4338	0.6051
A	230.2189	1.1149	0.6003	0.1967	0.3287
A	224.4657	3.5757	1.7986	0.6337	0.7758
A	205.0036	2.3993	0.3459	0.5076	0.6734
A	193.0899	2.3675	0.8432	0.4667	0.6364
A	181.0198	4.7488	1.0126	0.6438	0.7833
A	173.9140	5.3655	0.1824	0.5778	0.7324
A	165.2621	2.9999	1.5123	0.4817	0.6502

A	164.0686	2.3428	0.6265	0.7396	0.8503
A	137.0162	1.1875	0.4178	0.2074	0.3436
A	125.9114	14.9764	1.1041	0.7490	0.8565
A	116.1278	1.3474	0.9126	0.4349	0.6062
A	112.4164	2.3508	0.6203	0.6655	0.7992
A	101.2860	2.5070	0.1421	0.5183	0.6828
A	94.4066	0.7475	0.8356	0.7395	0.8502
A	77.2619	0.7762	0.5102	0.7127	0.8322
A	75.4063	4.7937	0.1993	0.6835	0.8120
A	67.1321	0.7963	1.2631	0.7450	0.8539
A	65.1761	0.3545	0.3423	0.7488	0.8564
A	60.1511	1.0870	1.6471	0.7500	0.8571
A	55.8001	0.9898	0.2329	0.6668	0.8001
A	54.8607	4.6279	1.0681	0.7483	0.8560
A	49.5930	0.6365	0.0355	0.7460	0.8545
A	38.5388	0.9825	0.0829	0.7481	0.8559
A	30.1132	0.0677	0.3180	0.7352	0.8474
A	22.7756	0.4931	0.2981	0.7440	0.8532
A	18.8816	1.8725	0.2614	0.7405	0.8509

67

C	-4.451143	0.417002	1.540606
C	-3.713653	1.592563	0.863936
C	-4.069615	1.436850	-0.623308
N	-5.102633	0.554464	-0.701847
C	-5.593161	0.045373	0.568596
C	-2.209068	1.565735	1.145453
C	-1.356266	2.558180	0.329186
C	-1.634975	3.952498	0.692304
N	-1.866447	5.039329	1.027018
O	-3.550682	2.014783	-1.569421
N	0.073470	2.290822	0.508432
C	0.673318	1.291514	-0.194799
O	0.098950	0.648414	-1.062752
C	2.154162	1.028548	0.174406
C	3.009619	2.247835	-0.151566
C	3.844113	1.908482	-1.370069
C	3.509336	0.490439	-1.778192
N	2.705932	-0.037698	-0.660233
C	4.519548	2.170399	-0.040083
C	5.214999	3.516872	0.078859
C	5.215448	1.069629	0.738820
C	2.162050	-1.280418	-0.806452
C	1.300922	-1.848063	0.328524
C	2.041279	-2.948274	1.154536
C	1.155898	-3.317365	2.356945
O	2.394901	-1.972191	-1.795847
N	0.081284	-2.332639	-0.310651
C	-1.130733	-1.992772	0.159541
C	-2.283349	-2.166246	-0.849189
F	-3.414872	-2.524831	-0.221099

O	-1.365113	-1.522208	1.266850
C	2.309084	-4.206177	0.314126
C	3.372382	-2.369070	1.662087
F	-2.503771	-1.002109	-1.480199
F	-2.005484	-3.101998	-1.785169
H	4.693005	4.294413	-0.489581
H	6.241956	3.456095	-0.300907
H	5.263394	3.840439	1.125765
H	4.715188	0.104625	0.648047
H	6.246065	0.950807	0.383567
H	5.263516	1.328653	1.803824
H	3.975621	2.633350	-2.167681
H	2.532220	3.217810	-0.054441
H	2.213829	0.756594	1.236378
H	4.381099	-0.153972	-1.927347
H	2.920188	0.473951	-2.702057
H	0.989984	-1.072230	1.028424
H	0.181116	-2.633781	-1.273626
H	3.857808	-3.080789	2.338417
H	4.065210	-2.167591	0.838687
H	3.220391	-1.435576	2.218516
H	1.653813	-4.080348	2.966012
H	0.955962	-2.447257	2.991603
H	0.191352	-3.718767	2.033653
H	2.824105	-4.954858	0.926797
H	1.369971	-4.649423	-0.033706
H	2.923813	-3.984256	-0.560681
H	0.542589	2.691808	1.309769
H	-1.592765	2.419756	-0.729633
H	-1.827992	0.571999	0.912959
H	-2.034958	1.733700	2.215524
H	-3.764568	-0.430081	1.630748
H	-4.823757	0.661946	2.538314
H	-6.543675	0.521906	0.846904
H	-5.752953	-1.036361	0.521727
H	-5.535345	0.359441	-1.593918
H	-4.135882	2.556909	1.180885

Conformer **D**: B3LYPD3BJ/6-31+G(d,p)

Processing: nir-ba6pbed3j.log
PG=C01

Method	BasisSet	Imaginary Freqs
RB3LYP	6-31+G(d,p)	0

HF Energy
-1770.1920545

ZPE	E298	S298	Squasihar	Equasihar	Strans	Srot
344.26993	366.950	229.545	204.556	367.059	44.511	37.105

Population analysis using the SCF Density.

Label	Frequencies	IR Inten	Raman Activ	Depolar (P)	Depolar (U)	Dipole
A	3645.8389	42.7039	146.4683	0.2247	0.3670	5.5782
A	3622.9718	25.2570	59.0876	0.1080	0.1949	
A	3592.5881	85.0341	38.8600	0.0739	0.1375	
A	3187.6815	9.8368	134.8128	0.0798	0.1479	
A	3180.4293	8.5865	70.0630	0.6367	0.7780	
A	3156.5465	8.7108	79.5372	0.4919	0.6594	
A	3143.6629	13.3965	40.8768	0.7126	0.8322	
A	3140.3473	14.6597	151.1061	0.3769	0.5475	
A	3133.0996	19.8193	37.3835	0.1658	0.2845	
A	3129.6271	17.1569	48.2628	0.6267	0.7705	
A	3125.0960	24.7260	74.4905	0.5066	0.6725	
A	3119.8428	29.9745	58.4904	0.7335	0.8463	
A	3111.9914	61.5586	123.2899	0.6552	0.7917	
A	3108.2064	10.2604	18.6027	0.6715	0.8035	
A	3107.8926	25.0894	69.6782	0.7086	0.8295	
A	3102.0278	16.7200	39.4671	0.4060	0.5775	
A	3099.1302	13.7383	67.0617	0.6939	0.8193	
A	3096.1691	23.2247	35.2804	0.0925	0.1694	
A	3095.1802	37.0472	145.9616	0.7051	0.8270	
A	3092.1394	30.8239	122.7275	0.2858	0.4445	
A	3090.6026	6.1771	30.1876	0.6765	0.8071	
A	3069.5670	13.6699	98.9449	0.1736	0.2958	
A	3060.6723	21.1481	128.3623	0.0387	0.0746	
A	3048.8901	30.7720	390.1908	0.0378	0.0728	
A	3043.1453	33.3132	117.2208	0.0501	0.0955	
A	3042.2478	19.7073	24.1874	0.1162	0.2083	
A	3038.3191	58.2057	293.6056	0.0389	0.0749	
A	3035.2483	16.3043	14.8703	0.1782	0.3026	
A	3029.9393	14.8739	82.9960	0.0421	0.0808	
A	3029.5814	44.6653	42.6637	0.1946	0.3257	
A	3026.3486	11.5065	74.2574	0.2544	0.4056	
A	3017.8191	80.5044	229.0360	0.2096	0.3466	
A	2353.3563	10.6100	154.2544	0.1715	0.2927	
A	1782.0344	544.6556	8.7399	0.3169	0.4813	
A	1771.2942	256.7016	15.0833	0.1394	0.2447	
A	1765.4403	199.8619	9.7440	0.1095	0.1974	
A	1717.2233	298.5609	12.3106	0.3020	0.4639	
A	1567.1742	170.4426	6.0347	0.1556	0.2692	
A	1541.0813	221.8905	6.2073	0.7376	0.8490	
A	1537.7797	16.2160	11.4586	0.5435	0.7043	
A	1527.2550	26.5458	0.7027	0.6752	0.8061	
A	1520.4301	6.9143	1.7679	0.5538	0.7129	
A	1517.5050	6.9412	11.0796	0.6939	0.8193	
A	1516.6348	4.5125	5.9848	0.7436	0.8529	
A	1510.0365	10.6158	6.4007	0.7208	0.8378	
A	1509.5687	4.2695	9.7740	0.7160	0.8345	
A	1507.6799	9.4691	10.7086	0.6514	0.7889	
A	1500.2132	4.2392	1.6186	0.7342	0.8467	

A	1495.8960	9.4762	1.3804	0.7396	0.8503
A	1495.1965	2.8974	9.6335	0.7448	0.8537
A	1491.7899	0.2739	9.0179	0.7481	0.8559
A	1488.9145	1.7510	4.7617	0.7445	0.8535
A	1483.3122	1.7060	5.8543	0.7499	0.8571
A	1459.6371	179.0940	3.0642	0.3742	0.5446
A	1458.6800	46.6697	2.7540	0.3927	0.5639
A	1444.2359	44.3977	11.5290	0.1612	0.2776
A	1441.6630	45.8987	5.0659	0.2500	0.4001
A	1429.0797	1.4258	8.3883	0.2138	0.3523
A	1417.6237	3.8756	1.7697	0.7486	0.8562
A	1411.9733	19.4305	0.4034	0.6060	0.7547
A	1410.1902	5.9852	1.8559	0.6036	0.7528
A	1409.6696	5.7556	0.1492	0.7465	0.8548
A	1393.5187	1.1495	10.8233	0.6564	0.7926
A	1383.9507	37.4270	3.0764	0.7143	0.8333
A	1375.6738	7.1298	3.6219	0.1290	0.2286
A	1373.2223	25.3202	6.7723	0.7438	0.8531
A	1357.9564	15.9911	0.9205	0.6349	0.7767
A	1354.6942	4.5578	2.6279	0.5497	0.7094
A	1343.7503	42.4020	4.6216	0.4787	0.6474
A	1339.4659	21.7668	1.2474	0.7398	0.8504
A	1333.2788	17.1627	2.7409	0.6458	0.7848
A	1328.2331	18.3932	5.8203	0.4144	0.5860
A	1321.0523	61.5595	13.6534	0.7208	0.8378
A	1296.8301	60.5123	7.9558	0.2200	0.3607
A	1292.6319	11.7805	3.0322	0.4638	0.6337
A	1278.6573	89.4668	2.5491	0.3792	0.5499
A	1273.5584	3.9572	4.7923	0.6748	0.8058
A	1259.9117	12.7436	10.7846	0.4009	0.5724
A	1250.2946	0.8997	4.4265	0.6555	0.7919
A	1242.6388	16.3990	4.1769	0.7285	0.8429
A	1233.8334	52.5535	6.7544	0.5123	0.6775
A	1231.2858	22.3396	3.2664	0.3795	0.5502
A	1229.6854	47.2756	2.7032	0.4979	0.6648
A	1220.0565	57.1897	5.5789	0.5831	0.7366
A	1217.6975	72.5310	3.7387	0.7500	0.8571
A	1215.5196	124.8120	2.5863	0.6325	0.7749
A	1212.4295	4.8317	0.4104	0.1429	0.2501
A	1211.2438	20.1850	7.9551	0.4473	0.6181
A	1173.2846	196.7466	3.1896	0.3571	0.5263
A	1157.1918	197.6064	1.1651	0.6991	0.8229
A	1156.9872	23.4740	6.7291	0.6242	0.7686
A	1137.0596	15.1386	1.5650	0.1410	0.2471
A	1133.6487	1.1827	4.5927	0.5432	0.7040
A	1117.9932	2.8717	7.1901	0.4645	0.6343
A	1114.9046	3.5697	0.7153	0.4512	0.6219
A	1113.2941	4.3613	1.4840	0.7300	0.8439
A	1091.0263	10.8286	2.9767	0.2625	0.4158
A	1075.7071	16.6618	5.3960	0.3595	0.5289
A	1066.9522	2.4496	5.1216	0.4388	0.6099

A	1060.8416	5.9392	1.6969	0.1694	0.2897
A	1055.1196	1.7243	1.5367	0.5053	0.6714
A	1049.3443	2.4871	3.7773	0.6648	0.7987
A	1046.6208	5.8446	2.0122	0.7499	0.8571
A	1039.6835	2.5886	0.8034	0.4510	0.6216
A	1024.8653	0.1576	5.8175	0.3348	0.5017
A	1003.0788	0.1807	2.8134	0.1805	0.3058
A	1002.1088	5.8582	3.9702	0.7366	0.8483
A	985.0066	4.8102	5.6076	0.1780	0.3022
A	975.6287	3.6431	2.9745	0.7450	0.8539
A	972.4450	1.7161	4.0377	0.7421	0.8520
A	969.7064	2.1774	1.3177	0.6779	0.8081
A	952.8800	5.3930	2.1825	0.4773	0.6462
A	948.6703	3.3127	3.6932	0.4791	0.6479
A	946.9564	0.5371	4.0440	0.7453	0.8541
A	945.8613	1.4752	8.2633	0.1803	0.3055
A	931.0478	18.9098	11.4411	0.0913	0.1673
A	907.0362	17.2265	15.7238	0.4013	0.5727
A	901.0740	0.7293	2.4723	0.5274	0.6906
A	896.9492	2.8986	4.6925	0.2084	0.3449
A	865.6134	7.7568	1.4007	0.7499	0.8571
A	849.0549	2.1636	4.7350	0.1839	0.3107
A	824.0393	6.5124	4.2867	0.7115	0.8315
A	808.8639	1.6946	2.2252	0.1952	0.3266
A	803.7388	8.4762	0.8731	0.2661	0.4204
A	788.0162	5.0536	5.9358	0.1733	0.2953
A	770.9363	11.6353	1.0690	0.0739	0.1376
A	760.5948	3.1469	4.6279	0.0424	0.0813
A	733.2483	11.9637	1.3538	0.6557	0.7921
A	718.7370	13.6776	4.5679	0.0839	0.1547
A	713.5873	12.0278	1.1512	0.2047	0.3398
A	712.3384	27.5802	6.9917	0.0333	0.0645
A	697.3958	4.5943	3.1375	0.5263	0.6896
A	684.9313	5.3783	7.1120	0.2803	0.4379
A	664.5716	4.6309	5.2026	0.2063	0.3420
A	652.2257	86.8703	0.9274	0.1322	0.2336
A	627.1709	2.3173	1.1839	0.6761	0.8067
A	606.9726	20.1971	4.1936	0.1029	0.1866
A	594.2873	8.7348	1.0173	0.7496	0.8569
A	559.7813	0.1021	1.5889	0.6837	0.8122
A	554.9854	3.0561	0.4153	0.3224	0.4876
A	543.2408	1.8942	1.8037	0.2135	0.3519
A	524.1940	92.7546	0.6527	0.7111	0.8311
A	504.2855	9.8159	0.5986	0.7325	0.8456
A	482.1284	18.1633	1.4950	0.4175	0.5891
A	465.3383	6.5343	0.9497	0.2405	0.3877
A	461.2681	15.5034	0.0817	0.6529	0.7900
A	450.9338	6.1703	0.3227	0.6111	0.7586
A	440.5258	70.7356	0.2113	0.6858	0.8136
A	421.5931	7.5492	2.1281	0.0962	0.1755
A	417.7422	1.5531	0.4548	0.6826	0.8114

A	405.4503	1.4019	0.5983	0.6268	0.7706
A	398.8281	14.5177	0.5242	0.6983	0.8224
A	391.7957	2.2730	0.6170	0.4974	0.6644
A	370.9523	8.6936	0.9185	0.6229	0.7676
A	360.1588	3.1215	1.7990	0.5455	0.7059
A	340.2717	5.1010	0.7295	0.6965	0.8211
A	326.4449	10.8550	0.3276	0.7028	0.8255
A	318.0203	0.6381	0.7863	0.3407	0.5082
A	308.4372	3.6151	1.0572	0.4208	0.5924
A	295.6171	3.9644	0.5070	0.7367	0.8484
A	293.0034	6.2477	0.4986	0.2486	0.3983
A	287.6494	4.5723	0.9500	0.7361	0.8480
A	282.9956	1.5589	0.1516	0.6000	0.7500
A	279.4246	0.8413	1.5226	0.3208	0.4857
A	268.0732	1.3397	0.9851	0.7163	0.8347
A	254.6989	1.5397	0.2071	0.4531	0.6236
A	244.9427	8.6835	1.6700	0.2543	0.4055
A	236.1704	1.6023	1.8439	0.1353	0.2384
A	233.5901	7.0517	0.2192	0.1217	0.2170
A	228.9223	2.1645	1.4925	0.0768	0.1426
A	224.8297	2.2904	1.2699	0.5210	0.6851
A	204.6383	1.6093	0.4451	0.2680	0.4227
A	189.6891	2.8963	0.9621	0.3277	0.4937
A	175.7723	1.4986	0.7529	0.6715	0.8035
A	174.4122	3.1749	0.6084	0.7324	0.8456
A	172.3934	7.5225	0.3316	0.7044	0.8266
A	163.3949	3.5382	1.6996	0.5042	0.6704
A	133.4755	0.1582	0.6126	0.2181	0.3581
A	118.1658	11.5063	1.2590	0.7391	0.8500
A	110.6668	1.5472	0.7145	0.4851	0.6533
A	106.7046	2.7055	0.1673	0.5085	0.6742
A	94.8212	2.0408	0.4177	0.6877	0.8149
A	90.2632	0.6826	0.5803	0.7360	0.8479
A	76.5922	0.3510	0.8296	0.6579	0.7937
A	68.4558	4.7852	0.5334	0.7284	0.8428
A	64.2697	6.1893	1.1670	0.7444	0.8535
A	58.3547	0.8028	1.5024	0.7494	0.8567
A	53.4948	0.1630	1.0772	0.7477	0.8556
A	46.4726	0.0695	0.1332	0.7349	0.8472
A	36.0691	1.7857	0.7026	0.7473	0.8554
A	31.4808	0.4236	0.1675	0.7450	0.8539
A	23.8927	1.3122	0.2640	0.7038	0.8262
A	22.0790	0.0744	0.0392	0.7276	0.8424
A	13.0263	2.1821	0.3515	0.7373	0.8488
A	9.6540	2.0872	0.1008	0.7459	0.8544

67

C	-4.572218	0.932330	1.649814
C	-3.700749	1.902616	0.826735
C	-4.105006	1.602779	-0.624732
N	-5.254390	0.875160	-0.585525

C	-5.792813	0.645360	0.749428
C	-2.205005	1.732016	1.098601
C	-1.279300	2.608977	0.230158
C	-1.474646	4.041086	0.498790
N	-1.636855	5.159732	0.764485
O	-3.521786	1.970061	-1.639426
N	0.127673	2.271906	0.448000
C	0.741738	1.310853	-0.296489
O	0.195722	0.723626	-1.220344
C	2.207342	1.027307	0.105695
C	3.092276	2.209822	-0.276292
C	3.974197	1.772928	-1.430346
C	3.643006	0.331091	-1.748928
N	2.748751	-0.097388	-0.655362
C	4.595313	2.123167	-0.094181
C	5.307800	3.464957	-0.036590
C	5.235372	1.067888	0.788880
C	2.200181	-1.339440	-0.726012
C	1.231890	-1.791692	0.375368
C	1.890291	-2.789782	1.387531
C	0.887902	-3.045655	2.527018
O	2.496217	-2.126530	-1.627429
N	0.086263	-2.366207	-0.322733
C	-1.177388	-2.069960	0.014145
C	-2.258458	-2.639790	-0.936261
F	-2.941346	-3.627942	-0.315198
O	-1.542273	-1.439715	1.000698
C	2.254854	-4.126053	0.721244
C	3.158006	-2.138329	1.966029
F	-3.133442	-1.682632	-1.279172
F	-1.738134	-3.161249	-2.074823
H	4.822969	4.208174	-0.677663
H	6.347626	3.362957	-0.367724
H	5.317248	3.857528	0.986946
H	4.732701	0.102421	0.730736
H	6.281575	0.920060	0.498211
H	5.226429	1.392095	1.836249
H	4.147034	2.437254	-2.271132
H	2.625717	3.189550	-0.267494
H	2.250217	0.817391	1.182469
H	4.512450	-0.332376	-1.781567
H	3.121471	0.247688	-2.708256
H	0.850151	-0.951057	0.954270
H	0.283152	-2.802368	-1.216859
H	3.575264	-2.772437	2.754366
H	3.929628	-2.007402	1.202033
H	2.942642	-1.159365	2.410094
H	1.332987	-3.720446	3.265761
H	0.608688	-2.116066	3.033332
H	-0.029685	-3.510618	2.157700
H	2.704951	-4.791831	1.465326

H	1.364098	-4.624446	0.326540
H	2.960431	-3.991092	-0.100887
H	0.592643	2.644940	1.264657
H	-1.509471	2.417378	-0.822639
H	-1.921679	0.694621	0.911160
H	-2.004679	1.939838	2.157054
H	-4.015168	0.004416	1.814244
H	-4.862144	1.336354	2.622588
H	-6.625718	1.329776	0.961732
H	-6.155805	-0.380838	0.856245
H	-5.740855	0.643893	-1.440157
H	-3.999231	2.943597	1.017724

Conformer **D**: B3LYPD3BJ-SMD/6-31+G(d,p), in water solvent

Processing: nir-ba6pbed3jws.log
PG=C01

Method	BasisSet	Imaginary Freqs
RB3LYP	6-31+G(d,p)	0

HF Energy
-1770.2380057

ZPE	E298	S298	Squasihar	Equasihar	Strans	Srot
343.45066	366.161	227.267	204.922	366.274	44.511	37.228

.

Label	Frequencies	IR Inten	Raman Activ	Depolar (P)	Depolar (U)	Dipole
A	3613.4931	112.7153	310.1031	0.2048	0.3400	15.5331
A	3597.4522	210.7110	173.9909	0.1099	0.1981	
A	3591.5893	153.4475	212.9088	0.1309	0.2314	
A	3197.9543	12.5552	464.0313	0.0914	0.1675	
A	3190.5888	15.0994	253.6806	0.5891	0.7414	
A	3175.4625	7.9544	201.1551	0.1284	0.2276	
A	3155.3085	18.8449	208.9342	0.4935	0.6609	
A	3140.7921	41.3553	249.2164	0.6304	0.7733	
A	3135.4083	43.9731	216.9334	0.6952	0.8202	
A	3130.0101	34.1789	308.9897	0.3360	0.5030	
A	3127.3730	1.0902	186.9036	0.2221	0.3635	
A	3117.9259	51.4005	181.2127	0.7500	0.8571	
A	3116.9097	24.7208	168.1561	0.4277	0.5991	
A	3116.3080	62.1149	226.2348	0.7494	0.8568	
A	3115.4335	117.2231	453.5819	0.7360	0.8479	
A	3112.2029	5.4942	39.2580	0.7369	0.8485	
A	3111.3092	51.3126	199.3071	0.7496	0.8569	
A	3108.0467	26.0752	110.2180	0.7495	0.8568	
A	3102.9681	30.9012	218.6197	0.7009	0.8241	
A	3093.0660	104.1564	497.9754	0.7396	0.8503	
A	3091.7473	0.1717	27.7001	0.6728	0.8044	
A	3090.8944	20.1373	369.4050	0.1880	0.3166	
A	3069.9664	59.0292	346.1447	0.1036	0.1877	

A	3064.9005	13.5528	304.6717	0.0503	0.0957
A	3062.0533	55.3279	452.9876	0.0368	0.0710
A	3059.5525	72.7729	413.3667	0.1785	0.3030
A	3049.1571	25.9204	1347.6342	0.0065	0.0129
A	3042.2262	62.3287	180.1922	0.0123	0.0242
A	3039.7351	86.0684	288.9696	0.0471	0.0899
A	3039.4903	9.6802	431.9167	0.1595	0.2752
A	3039.3265	36.3528	314.0739	0.0296	0.0574
A	3029.9829	57.5571	305.0856	0.0088	0.0175
A	2305.1250	36.4742	295.9245	0.1363	0.2399
A	1717.6888	724.4100	44.6781	0.1872	0.3154
A	1672.5742	798.5270	39.0492	0.1785	0.3029
A	1666.3369	1188.4221	35.2101	0.3888	0.5599
A	1623.4179	970.0705	33.7256	0.4531	0.6236
A	1582.6521	200.7655	27.0578	0.1281	0.2272
A	1545.9758	492.0999	7.7442	0.7238	0.8398
A	1521.8409	24.1806	21.4037	0.7291	0.8433
A	1503.4400	37.6438	14.7778	0.7273	0.8421
A	1501.2258	24.2953	4.0850	0.7172	0.8353
A	1500.5184	2.9228	33.8638	0.5206	0.6848
A	1497.7254	8.1919	10.3957	0.7379	0.8492
A	1491.2498	8.4956	36.2470	0.7434	0.8528
A	1487.4259	35.5105	14.9177	0.7482	0.8560
A	1486.5042	31.4462	4.8542	0.5404	0.7017
A	1482.8383	170.6553	22.7462	0.1780	0.3022
A	1482.1485	84.2531	11.7755	0.2921	0.4521
A	1478.7132	72.6205	11.0112	0.1420	0.2488
A	1477.9732	3.1536	33.7769	0.7182	0.8360
A	1477.5530	2.5439	9.4097	0.6504	0.7881
A	1473.0333	8.5976	35.3905	0.7235	0.8396
A	1472.3105	0.7006	22.2695	0.7471	0.8552
A	1458.3718	72.7172	29.6566	0.4529	0.6235
A	1442.0770	21.5226	57.6515	0.2508	0.4010
A	1423.5727	4.8206	5.9833	0.0514	0.0977
A	1418.7769	1.1895	7.9282	0.4731	0.6424
A	1411.6330	7.4641	2.8725	0.6989	0.8228
A	1403.0658	2.2452	10.4627	0.2619	0.4151
A	1401.8134	18.7192	0.9908	0.6021	0.7516
A	1401.1569	29.8048	10.9976	0.4161	0.5877
A	1396.0499	42.2883	15.2165	0.1924	0.3228
A	1385.4604	18.4348	20.6155	0.7125	0.8321
A	1384.4113	64.4263	22.3082	0.7366	0.8483
A	1376.4536	23.3587	11.6763	0.6884	0.8154
A	1373.8214	4.6509	6.7489	0.7494	0.8568
A	1350.9133	68.9958	12.2586	0.6857	0.8135
A	1344.6137	16.0180	7.7069	0.5065	0.6724
A	1338.0164	19.0077	11.2523	0.5695	0.7257
A	1337.0406	3.4109	3.7293	0.7427	0.8523
A	1323.9017	1.4878	9.5774	0.2060	0.3416
A	1314.8870	45.3564	37.0793	0.1967	0.3287
A	1309.5517	154.4745	51.0939	0.6349	0.7767

A	1300.8093	15.1626	16.8692	0.6205	0.7658
A	1288.2234	32.0286	10.8667	0.5343	0.6965
A	1282.5175	56.4821	8.4261	0.4753	0.6443
A	1261.9730	91.6542	7.9209	0.3916	0.5628
A	1253.0233	26.1677	45.9083	0.4230	0.5945
A	1246.8635	18.7264	33.5732	0.5207	0.6848
A	1235.0087	29.6549	14.7481	0.7478	0.8557
A	1231.8234	10.9533	24.7406	0.4955	0.6627
A	1228.1411	21.4709	5.7379	0.5775	0.7322
A	1217.5872	28.8127	22.2565	0.6934	0.8189
A	1214.0296	55.0381	11.3711	0.4850	0.6532
A	1209.0564	1.6398	8.9938	0.3011	0.4629
A	1205.2752	8.4379	9.6909	0.3521	0.5208
A	1198.1457	327.9044	9.6111	0.3528	0.5216
A	1148.9139	4.9122	17.7494	0.7435	0.8529
A	1148.2952	425.4908	4.8227	0.3821	0.5529
A	1146.5301	38.5884	8.4893	0.1089	0.1965
A	1134.0801	3.3862	29.2648	0.5767	0.7315
A	1132.1144	8.9421	17.7946	0.4264	0.5979
A	1117.9012	450.3342	3.7788	0.7464	0.8548
A	1110.9437	3.4136	4.7735	0.6236	0.7681
A	1102.1027	41.9964	7.0413	0.5389	0.7004
A	1095.8748	8.2673	10.0593	0.1863	0.3140
A	1082.7137	4.8942	5.2064	0.2640	0.4178
A	1063.9662	12.3862	7.9224	0.4997	0.6664
A	1059.5991	16.6530	9.0923	0.4811	0.6497
A	1054.0061	2.9660	8.2676	0.4516	0.6222
A	1041.9208	4.0900	10.0226	0.7286	0.8430
A	1039.4926	3.3684	9.7977	0.5111	0.6764
A	1026.6773	4.5091	16.4997	0.3139	0.4778
A	1025.3658	44.4984	5.2081	0.6645	0.7984
A	1006.4769	3.3425	12.2926	0.4335	0.6048
A	998.5480	9.7921	11.6088	0.7481	0.8559
A	981.1612	10.1569	19.2868	0.4654	0.6352
A	976.2097	3.1963	4.7443	0.6720	0.8038
A	969.9700	0.6478	6.9373	0.5377	0.6994
A	969.0348	1.9025	3.5651	0.6026	0.7521
A	963.0434	7.5076	7.2239	0.6175	0.7635
A	953.0151	0.6410	13.9470	0.4333	0.6046
A	950.3679	13.1563	14.8507	0.3285	0.4946
A	945.8631	3.6820	11.3989	0.5271	0.6903
A	927.9072	29.4928	31.4167	0.1241	0.2207
A	901.0720	32.6526	32.0725	0.2904	0.4501
A	896.8393	3.2951	14.7750	0.4249	0.5964
A	891.9851	5.2706	7.0746	0.3260	0.4917
A	856.9783	14.7751	6.2488	0.5743	0.7296
A	834.6598	17.1765	21.9400	0.1611	0.2775
A	821.8987	10.5453	13.3114	0.7106	0.8308
A	807.9645	0.8860	4.6165	0.1244	0.2212
A	799.9921	16.8033	3.0889	0.6545	0.7912
A	787.5393	4.9023	8.7488	0.1899	0.3191

A	770.7603	24.7216	6.3852	0.1905	0.3201
A	764.2669	2.1170	4.6152	0.1465	0.2556
A	753.0939	30.1238	4.9263	0.2439	0.3922
A	715.8900	9.8199	6.9221	0.1131	0.2032
A	712.7183	18.5850	14.9088	0.0711	0.1327
A	703.2057	28.6446	14.6312	0.0309	0.0600
A	698.8723	8.0930	5.3467	0.7029	0.8256
A	688.4488	10.1083	6.3383	0.6660	0.7995
A	684.5560	3.5911	17.8011	0.3987	0.5701
A	643.4157	7.7489	2.9633	0.4041	0.5756
A	607.7331	52.9422	7.3120	0.2052	0.3405
A	601.5931	28.6846	2.8217	0.6419	0.7819
A	592.2879	142.8121	1.1848	0.1506	0.2618
A	582.0846	8.0082	4.3931	0.1786	0.3031
A	557.6523	13.1214	5.2783	0.7187	0.8364
A	540.7443	3.7034	3.9075	0.2773	0.4343
A	531.1623	188.7517	1.5702	0.6943	0.8195
A	500.6802	34.5898	1.2818	0.6951	0.8201
A	476.3903	29.3325	3.1612	0.5451	0.7056
A	461.9919	8.9190	1.4294	0.4906	0.6582
A	455.5532	20.2210	0.0809	0.6248	0.7691
A	452.9476	22.5165	1.1158	0.7079	0.8290
A	434.9231	146.5574	0.4016	0.0722	0.1347
A	416.8129	7.5540	4.2180	0.3556	0.5247
A	411.1596	8.0726	2.7091	0.3951	0.5664
A	402.9562	4.2114	2.0579	0.2755	0.4320
A	390.5983	8.3729	1.3354	0.3999	0.5713
A	384.7291	8.6879	1.2801	0.6699	0.8023
A	377.4191	21.1403	0.9536	0.6664	0.7998
A	357.2257	10.1386	4.0707	0.3641	0.5338
A	344.6337	6.3320	1.5889	0.6323	0.7747
A	324.5719	15.7460	1.3778	0.6787	0.8086
A	317.9718	1.6517	0.8307	0.4658	0.6356
A	308.7124	7.1497	2.3178	0.6705	0.8027
A	294.3187	10.1667	4.1317	0.7495	0.8568
A	293.8450	6.2294	0.8992	0.4378	0.6090
A	288.6591	6.8784	1.8984	0.7340	0.8466
A	280.7894	5.6590	2.0633	0.6804	0.8098
A	273.6814	1.8539	1.0964	0.4536	0.6241
A	258.0586	6.4238	2.7619	0.4865	0.6546
A	257.2987	4.1615	0.4621	0.7353	0.8475
A	246.0112	5.6238	5.8579	0.0495	0.0943
A	238.0937	28.0517	1.2752	0.7229	0.8391
A	232.9756	4.6534	2.3257	0.6694	0.8019
A	229.7123	2.0596	1.4543	0.1526	0.2648
A	221.9592	7.5243	1.1987	0.7288	0.8432
A	206.9499	0.7321	0.9427	0.2785	0.4357
A	197.1020	5.7831	1.8623	0.4111	0.5827
A	185.9061	14.8682	3.8735	0.6368	0.7781
A	172.9363	3.5988	0.3845	0.3654	0.5352
A	164.0550	5.0238	2.1455	0.5871	0.7398

A	161.4543	0.3905	1.0934	0.6335	0.7757
A	133.3789	1.8529	0.4869	0.3711	0.5413
A	117.1299	5.4476	0.1730	0.7261	0.8413
A	116.4265	7.8844	0.6782	0.7187	0.8363
A	96.9228	13.9669	1.2870	0.7399	0.8505
A	87.1851	2.4401	1.7851	0.7426	0.8523
A	82.6193	1.1341	0.2188	0.5582	0.7164
A	72.5827	4.1791	6.6489	0.7492	0.8566
A	68.7436	22.5774	2.5461	0.7331	0.8460
A	58.8584	1.6094	0.5278	0.7478	0.8557
A	55.1890	1.3569	1.3546	0.7489	0.8564
A	50.7093	2.4650	0.8704	0.7497	0.8569
A	44.9202	7.3458	0.4945	0.7410	0.8512
A	42.1053	5.1159	2.6827	0.7451	0.8539
A	36.6780	0.2491	0.4878	0.7470	0.8552
A	31.8329	1.2136	0.1482	0.7452	0.8540
A	25.3557	0.3130	0.3654	0.7497	0.8570
A	22.8052	3.9109	0.3795	0.7497	0.8570
A	16.2210	4.9723	0.3286	0.7377	0.8491

67

C	-4.969475	2.060074	1.931848
C	-4.070077	2.002185	0.682610
C	-4.622983	0.806245	-0.091758
N	-5.819628	0.481924	0.432723
C	-6.278821	1.390858	1.486812
C	-2.580993	1.912788	1.017880
C	-1.645945	1.955788	-0.201761
C	-1.839384	3.186364	-0.996435
N	-1.988297	4.154438	-1.623885
O	-4.082630	0.236827	-1.059369
N	-0.258644	1.841868	0.220821
C	0.684799	1.289098	-0.567287
O	0.451722	0.884348	-1.714932
C	2.088339	1.219970	0.050311
C	2.856948	2.484336	-0.316498
C	3.980635	2.097599	-1.257594
C	3.917812	0.602843	-1.476752
N	2.867387	0.128441	-0.551354
C	4.298270	2.629836	0.125581
C	4.808303	4.059480	0.175060
C	4.895471	1.741502	1.200206
C	2.572640	-1.181080	-0.484960
C	1.476462	-1.642600	0.481208
C	2.027020	-2.418722	1.723323
C	0.847046	-2.660908	2.680154
O	3.169468	-2.021154	-1.189097
N	0.529834	-2.428376	-0.307891
C	-0.786253	-2.220335	-0.270661
C	-1.621471	-3.090250	-1.238088
F	-2.576221	-3.758506	-0.557581

O	-1.396639	-1.414013	0.434599
C	2.659787	-3.766720	1.348313
C	3.071485	-1.531579	2.418987
F	-2.240568	-2.303185	-2.143475
F	-0.891268	-4.002462	-1.919110
H	4.352057	4.675238	-0.606625
H	5.895624	4.084144	0.036083
H	4.581755	4.517863	1.145058
H	4.559974	0.705514	1.142999
H	5.988674	1.743997	1.120029
H	4.633900	2.123789	2.193848
H	4.215662	2.713494	-2.119286
H	2.268625	3.381790	-0.476045
H	2.013216	1.085760	1.133311
H	4.853602	0.081982	-1.252198
H	3.636694	0.371322	-2.509339
H	0.918694	-0.795610	0.870982
H	0.910992	-3.128638	-0.935032
H	3.389940	-2.004915	3.353195
H	3.960483	-1.391569	1.796545
H	2.661148	-0.545528	2.660387
H	1.205669	-3.167562	3.581906
H	0.380621	-1.716544	2.979726
H	0.080265	-3.294876	2.223972
H	2.967747	-4.282002	2.264290
H	1.950335	-4.416907	0.825922
H	3.542170	-3.640355	0.717067
H	-0.012857	2.160126	1.151905
H	-1.873257	1.117743	-0.864768
H	-2.377625	0.973838	1.540658
H	-2.320816	2.736523	1.691321
H	-4.511474	1.474113	2.735803
H	-5.124888	3.078225	2.292327
H	-6.990592	2.117275	1.078745
H	-6.769209	0.836794	2.289081
H	-6.423276	-0.196008	-0.016801
H	-4.258971	2.881592	0.050947

Conformer **D**: B3LYPD3BJ-SMD/6-31+G(d,p), in acetonitrile solvent

Processing: nir-ba6pbed3jacs.log
PG=C01

Method	BasisSet	Imaginary Freqs
RB3LYP	6-31+G(d,p)	0

HF Energy
-1770.2421746

ZPE	E298	S298	Squasihar	Equasihar	Strans	Srot
343.46422	366.082	225.528	204.622	366.192	44.511	37.238

Label	Frequencies	IR Inten	Raman Activ	Depolar (P)	Depolar (U)	Dipole
A	3616.7794	96.4286	333.6804	0.2089	0.3457	11.8663
A	3593.2101	177.9213	149.8026	0.0904	0.1658	
A	3587.9150	114.4758	182.8143	0.1266	0.2248	
A	3190.1311	16.6713	476.1806	0.0848	0.1564	
A	3183.3085	15.2206	222.0273	0.7176	0.8356	
A	3157.1150	17.5082	211.1995	0.4636	0.6335	
A	3153.6092	11.9492	183.7396	0.1373	0.2415	
A	3132.8041	40.0260	194.5936	0.6967	0.8212	
A	3131.6832	36.7752	231.3171	0.6599	0.7951	
A	3129.1645	9.0892	206.6715	0.2988	0.4602	
A	3117.8621	47.2439	174.5455	0.7461	0.8546	
A	3117.1820	57.5821	190.1699	0.7348	0.8471	
A	3113.3435	30.2817	112.3950	0.1898	0.3190	
A	3111.9997	43.8067	307.9760	0.3619	0.5315	
A	3111.0458	118.2807	426.2189	0.7393	0.8501	
A	3107.3276	10.8396	55.6050	0.7461	0.8546	
A	3107.1310	50.6437	191.4817	0.7495	0.8568	
A	3103.6412	28.2253	111.9230	0.7499	0.8571	
A	3095.5678	34.3855	233.0832	0.7171	0.8353	
A	3088.1423	108.8265	496.1338	0.7453	0.8540	
A	3086.5438	1.1058	27.0562	0.7478	0.8557	
A	3078.8884	17.6626	311.5737	0.1560	0.2699	
A	3064.2777	56.9872	369.2125	0.0931	0.1703	
A	3059.9853	23.6946	252.7883	0.0869	0.1599	
A	3056.6428	62.9668	459.4513	0.0424	0.0814	
A	3046.3346	29.9633	1406.7729	0.0086	0.0171	
A	3039.9679	81.0097	376.4371	0.1858	0.3134	
A	3039.4644	69.3175	31.7783	0.1043	0.1889	
A	3037.5855	47.9329	4.3280	0.4987	0.6655	
A	3035.5546	70.9933	593.7632	0.0341	0.0660	
A	3032.3718	18.5224	430.7664	0.1687	0.2887	
A	3026.0021	56.9615	280.3910	0.0103	0.0203	
A	2342.1771	46.7527	342.5299	0.1454	0.2538	
A	1755.7043	599.9402	39.6230	0.1770	0.3008	
A	1727.6034	543.6042	36.5907	0.1837	0.3103	
A	1720.1021	933.3272	38.5003	0.3063	0.4690	
A	1676.9621	742.8755	35.8067	0.3214	0.4865	
A	1580.7689	227.4260	19.4264	0.1301	0.2302	
A	1541.7738	399.7854	8.2163	0.7496	0.8569	
A	1519.1757	13.1611	23.2839	0.7097	0.8302	
A	1505.3974	34.1963	2.9295	0.7090	0.8297	
A	1501.7214	1.6855	33.4273	0.5203	0.6844	
A	1499.8540	0.8409	18.2758	0.7496	0.8569	
A	1499.2089	13.0180	7.0733	0.6611	0.7960	
A	1491.7728	16.9668	36.5506	0.7227	0.8390	
A	1488.5528	16.4488	7.9357	0.7497	0.8570	
A	1488.1330	26.9645	22.5375	0.6956	0.8205	
A	1483.2955	6.3505	27.7427	0.7475	0.8555	
A	1482.7651	1.1174	6.4094	0.3374	0.5046	

A	1482.0344	30.9874	1.5272	0.3847	0.5556
A	1481.1602	4.3489	13.7045	0.7495	0.8568
A	1475.1426	31.0938	23.7307	0.6608	0.7957
A	1473.4562	0.8754	26.0980	0.7499	0.8571
A	1471.1920	309.0026	19.6548	0.1576	0.2723
A	1457.2844	76.7208	15.8880	0.4828	0.6512
A	1440.2332	17.9902	59.7761	0.2337	0.3788
A	1424.1344	8.4046	3.3606	0.1249	0.2221
A	1419.6531	1.5674	9.1302	0.4004	0.5718
A	1411.8343	7.2258	3.4090	0.4918	0.6594
A	1402.5979	20.8612	1.1104	0.6299	0.7730
A	1400.9896	12.2333	0.1811	0.4248	0.5963
A	1396.4031	26.8923	21.8325	0.6324	0.7748
A	1385.6437	29.6214	9.2464	0.3669	0.5369
A	1383.1790	42.6664	16.3089	0.5632	0.7206
A	1377.7669	56.9649	19.7579	0.5556	0.7143
A	1371.4230	36.1703	14.4345	0.6271	0.7709
A	1364.8556	6.7340	3.6315	0.7137	0.8330
A	1346.7901	50.0756	11.2101	0.6398	0.7804
A	1344.2972	11.2336	8.6921	0.5868	0.7396
A	1334.7137	15.1553	7.0625	0.6672	0.8004
A	1333.3087	1.9288	2.7130	0.5815	0.7354
A	1323.3835	0.8467	12.6485	0.2147	0.3535
A	1314.1773	34.8068	29.1884	0.2802	0.4378
A	1301.4411	159.9024	56.1995	0.6879	0.8151
A	1294.9152	9.6874	11.5273	0.6190	0.7646
A	1282.4673	38.9334	12.8382	0.3527	0.5215
A	1278.4542	85.2193	5.2513	0.6335	0.7756
A	1252.2094	20.0710	35.6034	0.4513	0.6220
A	1249.1204	34.7468	24.9997	0.5657	0.7226
A	1244.0009	105.7621	12.8241	0.4530	0.6235
A	1233.1736	106.6738	15.9599	0.7265	0.8416
A	1228.0637	6.2437	6.1478	0.2456	0.3943
A	1227.0225	7.3223	23.7190	0.5490	0.7089
A	1215.2407	40.3814	17.9883	0.6552	0.7917
A	1214.1509	27.3857	12.1355	0.7067	0.8281
A	1208.5151	1.3770	17.7051	0.3776	0.5482
A	1202.1658	12.4617	5.1564	0.5308	0.6935
A	1200.0553	329.4757	5.9093	0.3658	0.5356
A	1150.4094	15.7126	19.9365	0.7250	0.8406
A	1143.9573	350.6299	3.1341	0.2092	0.3460
A	1139.9606	34.4039	5.3239	0.1669	0.2861
A	1132.4397	11.8608	17.3916	0.6096	0.7575
A	1125.3879	4.0453	25.9796	0.4246	0.5961
A	1115.2605	438.0358	3.6165	0.7474	0.8554
A	1109.7111	3.8413	5.4229	0.5526	0.7118
A	1101.6802	61.3626	6.6705	0.5656	0.7225
A	1096.4446	11.1925	7.9255	0.2203	0.3610
A	1072.2644	7.5293	12.1508	0.1901	0.3195
A	1064.3988	31.1266	4.5240	0.5890	0.7414
A	1062.7003	6.0485	10.2858	0.5728	0.7284

A	1052.2025	2.6141	6.9237	0.4419	0.6130
A	1042.2911	4.5830	10.6340	0.7496	0.8569
A	1039.4119	3.4290	7.9839	0.5096	0.6752
A	1028.6515	34.4143	5.0127	0.6802	0.8096
A	1025.9774	1.6302	12.9703	0.2702	0.4254
A	1002.2138	3.3804	11.4602	0.3972	0.5686
A	998.9910	7.7886	10.9198	0.7496	0.8569
A	980.9983	13.3029	16.8756	0.3561	0.5251
A	975.7638	3.1739	5.2805	0.6895	0.8162
A	971.6227	1.6368	9.5231	0.7493	0.8567
A	968.4852	2.7088	3.2837	0.7350	0.8473
A	956.2137	6.0964	4.4041	0.4289	0.6004
A	949.8710	1.8512	4.0680	0.5920	0.7437
A	948.3308	6.7127	23.0278	0.4440	0.6149
A	946.4664	2.8856	12.0064	0.4425	0.6135
A	925.7591	34.7790	28.5820	0.0953	0.1740
A	902.9613	31.7715	35.8119	0.3387	0.5061
A	895.5842	1.3095	6.6392	0.7188	0.8364
A	890.8397	5.0146	9.1814	0.2569	0.4087
A	859.4203	13.4827	6.0251	0.6363	0.7778
A	838.3804	10.5909	11.8148	0.1512	0.2627
A	821.4075	14.5277	15.5641	0.6435	0.7831
A	808.4541	0.8117	5.3701	0.1598	0.2755
A	797.9890	15.9992	2.2334	0.6070	0.7555
A	788.7763	6.3482	12.3984	0.1358	0.2390
A	768.6865	18.3450	6.3643	0.1385	0.2432
A	762.8939	2.8830	7.2629	0.0831	0.1535
A	742.2560	27.9152	3.7803	0.4308	0.6021
A	714.3936	11.9180	3.7151	0.1362	0.2398
A	712.4070	20.7043	13.5942	0.0687	0.1286
A	704.2322	29.3336	14.5120	0.0242	0.0472
A	698.4238	4.6359	5.9321	0.6768	0.8073
A	685.6797	14.5888	8.9646	0.4865	0.6546
A	681.7548	0.0552	14.2532	0.3808	0.5516
A	635.0196	8.7541	1.6796	0.7249	0.8405
A	610.0701	118.8000	5.3343	0.1365	0.2403
A	602.5167	51.4390	4.8418	0.4626	0.6326
A	599.6589	64.6984	1.4580	0.0906	0.1662
A	573.6371	4.3046	5.4212	0.3777	0.5483
A	557.8139	9.7500	2.1034	0.7496	0.8569
A	542.4387	185.1222	1.5650	0.5948	0.7459
A	540.2354	4.1265	3.7662	0.3542	0.5231
A	501.2454	30.2151	1.3026	0.7117	0.8315
A	481.3442	22.4704	3.0090	0.4618	0.6318
A	465.9378	52.9609	1.1548	0.6200	0.7654
A	459.8929	60.8549	0.1486	0.1602	0.2762
A	452.6564	20.0190	0.7737	0.5836	0.7371
A	446.7734	75.2760	0.2437	0.1478	0.2575
A	417.2224	7.9787	3.5759	0.2586	0.4109
A	411.2252	7.8924	2.0135	0.4277	0.5992
A	404.4845	2.4865	1.3267	0.4855	0.6536

A	392.9289	4.2173	0.7867	0.7468	0.8550
A	388.6987	4.9493	2.2682	0.3307	0.4970
A	375.5175	32.0585	0.8730	0.7440	0.8532
A	357.9910	6.2344	4.0875	0.4480	0.6188
A	346.3431	7.5731	1.5409	0.6410	0.7813
A	322.4186	11.4279	1.1315	0.5880	0.7406
A	321.7038	5.4161	0.4287	0.3120	0.4756
A	307.9639	7.4132	1.6171	0.6231	0.7678
A	297.3373	7.2793	2.2480	0.7378	0.8491
A	297.0711	5.7558	1.1211	0.3266	0.4924
A	292.4827	2.3098	1.0618	0.6281	0.7716
A	284.4930	8.7930	2.3846	0.7165	0.8348
A	273.2295	0.9178	1.4898	0.3039	0.4662
A	262.6967	6.0303	2.5978	0.4147	0.5862
A	256.3027	7.1685	0.4903	0.7177	0.8357
A	245.4352	6.1310	4.4090	0.1036	0.1877
A	239.7106	14.4795	1.4614	0.6747	0.8057
A	234.3667	9.2359	0.9795	0.1974	0.3298
A	230.4900	0.7197	1.8812	0.0901	0.1653
A	223.7218	7.7940	1.7151	0.6981	0.8222
A	210.4256	2.4708	0.6668	0.2938	0.4542
A	196.9789	4.4358	1.9397	0.4998	0.6665
A	185.4285	12.2257	2.8035	0.6060	0.7546
A	173.6442	8.3943	0.8406	0.6608	0.7958
A	169.9602	1.2615	1.0121	0.7283	0.8428
A	163.6479	6.0902	3.0614	0.5272	0.6904
A	134.1371	0.6301	0.4671	0.3965	0.5678
A	115.7256	10.4233	0.8342	0.7132	0.8326
A	114.6864	2.6079	0.0842	0.5077	0.6735
A	99.7369	9.3037	1.3184	0.7113	0.8313
A	92.2257	0.6806	1.1496	0.7409	0.8512
A	87.6753	2.8168	0.4139	0.6758	0.8065
A	77.1263	5.8993	4.8787	0.7459	0.8545
A	65.5661	9.6286	1.5587	0.7499	0.8571
A	61.8867	4.4516	1.9529	0.7331	0.8460
A	58.5636	0.6650	1.0685	0.7497	0.8570
A	55.0753	4.1665	0.6331	0.7234	0.8395
A	51.8363	3.2185	1.1071	0.7492	0.8566
A	46.0323	10.4510	1.5820	0.7496	0.8569
A	41.2500	0.6136	0.2501	0.7463	0.8547
A	29.1948	1.2661	0.1804	0.7476	0.8556
A	24.9232	1.0116	0.1522	0.7461	0.8546
A	22.7719	2.0727	0.2062	0.7494	0.8567
A	18.5351	5.0169	0.4771	0.7254	0.8409

67

C	-4.884291	1.465719	1.932280
C	-3.960507	1.974992	0.809517
C	-4.482281	1.249325	-0.435529
N	-5.691343	0.724997	-0.131107
C	-6.180722	1.065945	1.204876

C	-2.475582	1.747812	1.096023
C	-1.522941	2.268727	0.003177
C	-1.694011	3.717515	-0.231441
N	-1.829972	4.857973	-0.404226
O	-3.917483	1.172833	-1.534001
N	-0.138708	1.985026	0.336574
C	0.693363	1.319332	-0.502455
O	0.355934	0.896510	-1.608040
C	2.126959	1.162471	0.035376
C	2.939712	2.388899	-0.367643
C	3.975106	1.952257	-1.385650
C	3.817280	0.463362	-1.605509
N	2.813645	0.038689	-0.609328
C	4.414111	2.458045	-0.025459
C	4.997913	3.860730	-0.005920
C	5.040673	1.533268	1.000851
C	2.434346	-1.255132	-0.558221
C	1.373050	-1.678171	0.469261
C	1.970769	-2.457504	1.690540
C	0.840526	-2.676161	2.711030
O	2.925304	-2.110890	-1.310601
N	0.397019	-2.471762	-0.273367
C	-0.921816	-2.276660	-0.187803
C	-1.772862	-3.141614	-1.150269
F	-2.701052	-3.838683	-0.460163
O	-1.516003	-1.498028	0.553184
C	2.551417	-3.819380	1.280046
C	3.071496	-1.598954	2.333076
F	-2.428842	-2.350225	-2.027118
F	-1.049905	-4.032565	-1.870133
H	4.522114	4.504721	-0.752808
H	6.073678	3.832935	-0.218052
H	4.860744	4.324250	0.978756
H	4.648646	0.516630	0.960536
H	6.124659	1.480848	0.843844
H	4.870106	1.918991	2.013231
H	4.183586	2.562101	-2.258911
H	2.390838	3.318383	-0.480629
H	2.103549	1.025058	1.120851
H	4.738493	-0.107359	-1.452123
H	3.452561	0.254549	-2.616851
H	0.836002	-0.821360	0.871151
H	0.767459	-3.127935	-0.952358
H	3.425591	-2.082180	3.249646
H	3.931257	-1.478830	1.667265
H	2.700128	-0.603522	2.598674
H	1.234494	-3.200808	3.588034
H	0.417018	-1.722814	3.044589
H	0.031940	-3.284229	2.293856
H	2.937608	-4.328249	2.169958
H	1.786192	-4.464518	0.835146

H	3.368333	-3.711560	0.562619
H	0.202237	2.275783	1.246537
H	-1.747955	1.765179	-0.940340
H	-2.274381	0.677154	1.188924
H	-2.218057	2.226672	2.047439
H	-4.436167	0.580393	2.396596
H	-5.057416	2.211886	2.710338
H	-6.891802	1.899979	1.152631
H	-6.680277	0.210457	1.665529
H	-6.282210	0.319677	-0.847314
H	-4.147542	3.044166	0.634888

Conformer **TS1**: B3LYPD3BJ/6-31+G(d,p), in acetonitrile solvent

Processing: nir-tsh6pbed3jacs.log
PG=C01

WARNING: Imaginary frequencies

Method	BasisSet	Imaginary Freqs
RB3LYP	6-31+G(d,p)	1

HF Energy
-1770.2022122

ZPE	E298	S298	Squasihar	Equasihar	Strans	Srot
342.78437	364.976	221.707	201.977	365.075	44.511	37.233

Label	Frequencies	IR Inten	Dipole
A	3621.8294	177.0217	15.0532
A	3614.5323	97.8513	
A	3536.9578	275.0090	
A	3191.2517	15.8641	
A	3175.1748	25.7283	
A	3142.7868	11.7874	
A	3133.2622	8.0082	
A	3131.2946	36.8870	
A	3128.9513	61.2079	
A	3126.2711	17.4403	
A	3116.6388	89.6064	
A	3113.7062	63.0532	
A	3111.9489	42.5900	
A	3111.6584	20.4124	
A	3108.9253	42.9222	
A	3106.4171	41.8357	
A	3103.0458	25.4271	
A	3102.6857	75.4180	
A	3102.3174	12.9840	

A	3083.1154	81.9561
A	3078.2319	18.1390
A	3077.7791	53.4995
A	3076.7563	26.5311
A	3061.8547	39.2315
A	3049.1885	11.0277
A	3043.0015	56.6069
A	3039.3425	80.9767
A	3039.2246	44.7253
A	3031.9409	16.2983
A	3027.2337	114.0244
A	3021.7346	43.9097
A	3010.6764	122.0960
A	2343.1751	58.8334
A	1758.2118	310.5668
A	1740.3551	624.5877
A	1720.5442	1177.8400
A	1719.8191	220.2589
A	1609.5178	238.1624
A	1547.6242	385.9011
A	1518.3712	13.6324
A	1509.4934	11.0490
A	1504.3689	16.1279
A	1501.8322	15.7799
A	1501.4331	9.4239
A	1497.7871	3.7684
A	1493.7542	13.8808
A	1488.2972	31.3970
A	1487.1291	0.3913
A	1483.4326	2.7473
A	1478.7273	2.7237
A	1478.6022	7.9142
A	1478.0549	0.4150
A	1475.8163	2.6238
A	1457.0454	73.0454
A	1438.9913	2.0610
A	1435.9109	12.0996
A	1415.4118	2.3511
A	1414.4989	14.9472
A	1409.8311	10.7260
A	1404.7093	12.5434
A	1396.2505	30.8669
A	1391.1974	6.1718
A	1385.8001	23.1163
A	1383.2170	7.6405
A	1364.5794	18.4596
A	1362.6543	13.5011
A	1360.1284	58.6723
A	1344.4253	13.3552
A	1330.9515	12.6907
A	1329.4582	24.4036

A	1321.5730	31.1577
A	1315.6812	0.5652
A	1304.7937	108.3313
A	1296.3976	12.9653
A	1282.6942	29.8309
A	1278.5652	115.2836
A	1256.7998	43.4078
A	1251.0031	40.1693
A	1241.6802	112.1273
A	1230.4361	142.2307
A	1227.1525	17.2328
A	1218.0060	34.4448
A	1216.9363	4.9159
A	1207.7420	266.1156
A	1206.5344	9.9428
A	1200.7681	12.4687
A	1175.0695	489.6869
A	1152.8575	10.4333
A	1150.7973	345.0627
A	1140.0697	23.8192
A	1132.7585	77.3892
A	1127.5630	6.0401
A	1125.1890	6.2788
A	1118.3580	433.4497
A	1104.9401	18.0095
A	1097.3164	115.1114
A	1092.2100	23.3731
A	1065.5902	6.9710
A	1063.8216	24.5426
A	1062.1463	4.7510
A	1046.0599	10.4705
A	1042.0890	4.5620
A	1033.8556	1.0175
A	1030.4512	17.9814
A	1018.8248	1.2249
A	999.1268	8.7322
A	993.3276	9.4486
A	981.5930	11.5580
A	980.2641	7.9269
A	972.9309	6.6192
A	970.2409	5.0045
A	957.1600	5.4092
A	951.7712	1.5410
A	947.7901	11.1651
A	942.8884	5.5906
A	936.0539	30.6593
A	894.3129	2.6253
A	891.7343	22.6674
A	874.1283	2.7125
A	871.8742	35.1184
A	840.8923	2.5366

A	832.2480	13.9167
A	812.9497	28.4378
A	795.6962	7.9402
A	789.1333	10.6938
A	775.6793	34.1822
A	768.8789	8.8519
A	761.0079	18.2797
A	714.4331	42.6275
A	712.4421	12.6177
A	703.9423	3.3313
A	695.8245	4.8804
A	686.0979	16.3252
A	672.9075	16.7953
A	633.4474	54.3732
A	616.1639	110.9535
A	601.1993	17.2901
A	588.9253	75.0853
A	576.6961	8.8338
A	567.0740	131.9024
A	560.7336	27.8890
A	530.9985	16.1043
A	530.0390	170.9144
A	502.1060	35.5815
A	481.7517	26.7470
A	463.6888	23.7694
A	453.9712	6.1623
A	451.5710	9.7843
A	424.8408	2.7701
A	413.3849	12.8721
A	410.7764	7.4044
A	400.2715	4.3969
A	378.7847	14.8251
A	363.6877	10.1631
A	352.9987	1.7353
A	338.5566	0.8943
A	316.8035	3.5273
A	305.5788	7.2683
A	303.3125	10.5221
A	295.3367	5.4563
A	287.4052	7.2220
A	281.3126	6.6702
A	267.2926	12.8581
A	266.0281	13.0595
A	255.9386	5.4535
A	250.3484	2.5500
A	248.3478	19.4066
A	241.5535	5.5007
A	225.5195	1.4774
A	215.9280	4.2609
A	212.7702	12.0489
A	200.5687	2.0842

A	192.8588	8.6827
A	176.9355	2.6482
A	167.0649	16.6007
A	163.8071	6.7101
A	153.6328	3.2445
A	138.8990	6.1841
A	118.6821	3.1744
A	116.1953	2.3261
A	107.6749	1.1890
A	92.8333	14.4612
A	88.1799	4.0252
A	79.1819	12.2822
A	67.3308	1.2602
A	-66.2188	7.0365
A	62.7398	1.3432
A	58.9089	1.2448
A	53.1183	3.0969
A	45.7054	7.3789
A	38.8244	2.9788
A	34.2685	2.1804
A	28.3773	0.6091
A	19.3062	1.7167
A	15.7126	4.0339

67

C	4.479841	-1.645765	-1.384837
C	4.190574	-0.675526	-0.235602
C	4.842322	0.637537	-0.708324
C	5.964283	0.179675	-1.657447
N	5.441625	-1.091909	-2.158428
C	2.708970	-0.533314	0.110718
C	2.053829	-1.801690	0.696473
C	2.738757	-2.247483	1.924722
N	3.286452	-2.583753	2.892217
O	3.947449	-2.747346	-1.570610
N	0.644482	-1.591734	0.979770
C	-0.329551	-2.153815	0.211355
O	-0.103637	-3.006796	-0.649112
C	-1.747208	-1.648731	0.542294
C	-2.729508	-2.802224	0.464479
C	-3.555648	-2.619847	-0.792157
C	-3.083620	-1.350249	-1.468761
N	-2.286752	-0.656986	-0.426771
C	-4.221055	-2.550851	0.569547
C	-5.037399	-3.779380	0.941418
C	-4.805356	-1.267630	1.133563
C	-1.413295	0.400311	-0.821941
C	-1.883527	1.832663	-0.478911
C	-2.405308	2.677638	-1.685912
C	-1.308265	2.932069	-2.733594
O	-0.321656	0.211972	-1.324332

N	-0.775990	2.535415	0.176189
C	-0.242021	2.089202	1.310470
C	0.855996	2.983403	1.934968
F	1.242730	4.000681	1.131212
O	-0.558118	1.056855	1.905729
C	-3.571301	1.936126	-2.357672
C	-2.932053	4.016672	-1.134486
F	0.406254	3.524188	3.089028
F	1.950725	2.254450	2.227444
H	-4.604146	-4.694624	0.524418
H	-6.063913	-3.689261	0.564729
H	-5.088931	-3.895462	2.031221
H	-4.216470	-0.388517	0.869847
H	-5.826920	-1.124336	0.759739
H	-4.861552	-1.329556	2.227633
H	-3.783997	-3.462153	-1.438520
H	-2.349973	-3.777376	0.750667
H	-1.729537	-1.171356	1.526513
H	-3.907494	-0.705824	-1.785139
H	-2.478622	-1.602446	-2.351240
H	-2.696626	1.738125	0.244952
H	-0.391040	3.369013	-0.251297
H	-4.050038	2.600715	-3.083749
H	-3.228864	1.051902	-2.901873
H	-4.327414	1.629609	-1.627490
H	-3.353867	4.605865	-1.955149
H	-3.720666	3.849964	-0.392467
H	-2.145944	4.618852	-0.667826
H	-1.734119	3.511449	-3.559409
H	-0.465188	3.507718	-2.336317
H	-0.917502	1.996234	-3.141820
H	0.397330	-0.791646	1.556176
H	2.129183	-2.615566	-0.029856
H	2.136240	-0.273635	-0.783971
H	2.588204	0.283905	0.827881
H	4.106480	1.227370	-1.266372
H	5.217117	1.246055	0.117139
H	6.910038	0.023682	-1.123369
H	6.139019	0.878209	-2.479126
H	5.883082	-1.615169	-2.905206
H	4.740979	-1.063068	0.633707

Conformer **TS2**: B3LYPD3BJ/6-31G(d)

Processing: nir-tsd6dbed3j.log
PG=C01

WARNING: Imaginary frequencies

Method	BasisSet	Imaginary Freqs
RB3LYP	6-31G(d)	1

HF Energy
-1770.0407695

ZPE	E298	S298	Squasihar	Equasihar	Strans	Srot
346.51196	368.328	218.120	199.558	368.413	44.511	36.864

Label	Frequencies	IR Inten	Dipole
A	3628.5469	38.3684	6.6564
A	3568.3407	45.9519	
A	3475.8274	276.4918	
A	3202.5426	6.1437	
A	3199.1021	0.8713	
A	3183.5154	13.4091	
A	3157.8671	11.7238	
A	3153.3450	5.5056	
A	3143.9993	14.0694	
A	3134.4216	45.6756	
A	3130.1359	10.3109	
A	3124.0318	21.6887	
A	3120.6357	46.6100	
A	3114.6801	7.9356	
A	3113.8048	2.1448	
A	3111.8217	28.0482	
A	3107.6048	35.5180	
A	3101.6893	5.5992	
A	3099.6208	41.6282	
A	3092.7949	12.2344	
A	3082.0737	10.1990	
A	3078.4827	7.2652	
A	3077.4697	12.4316	
A	3073.8610	39.3234	
A	3063.6635	20.6165	
A	3051.5144	15.2752	
A	3047.0668	21.8083	
A	3042.7868	66.4373	
A	3036.9876	25.2237	
A	3035.0258	63.5024	
A	3028.4775	6.0092	
A	3013.4036	44.0813	
A	2365.5299	5.1970	
A	1804.0649	142.0653	
A	1795.9320	513.3914	
A	1775.6111	31.3276	
A	1771.9460	452.4588	
A	1586.0794	131.9567	
A	1558.7665	3.3092	
A	1551.1242	19.0326	
A	1547.4961	30.1547	

A	1545.8975	157.2715
A	1542.7464	10.1435
A	1540.5508	0.7760
A	1537.8728	1.6385
A	1535.8531	8.4996
A	1522.9395	5.1588
A	1520.8473	4.9643
A	1518.7143	1.9311
A	1517.9556	3.6385
A	1516.4719	0.4992
A	1515.3357	6.6613
A	1513.5027	0.5703
A	1470.5887	34.5910
A	1470.3211	26.9648
A	1464.5486	1.6962
A	1449.4583	0.6886
A	1440.4199	8.7447
A	1437.7532	5.0643
A	1431.5260	5.2887
A	1422.9465	7.8062
A	1416.9978	6.8421
A	1413.4181	7.5101
A	1405.5082	3.3093
A	1391.0351	5.6059
A	1380.5538	23.1547
A	1370.4376	3.5670
A	1360.9817	74.3035
A	1355.8430	4.6989
A	1346.8917	40.9200
A	1340.2678	10.2686
A	1335.4854	0.5693
A	1328.9305	3.9381
A	1314.4412	8.6559
A	1308.1227	122.7118
A	1289.8150	59.2242
A	1282.4653	21.9809
A	1267.6221	11.2249
A	1255.9417	61.7939
A	1252.4188	8.5510
A	1240.2246	219.5016
A	1236.2550	4.4953
A	1235.5743	35.9367
A	1230.6657	4.9085
A	1216.8733	107.4045
A	1212.3837	177.9429
A	1211.0602	92.7240
A	1205.1721	210.2624
A	1185.3369	218.7096
A	1173.7489	2.1567
A	1160.3642	19.7835
A	1147.4287	10.7396

A	1141.7878	17.0685
A	1133.5486	1.7514
A	1116.8797	7.8731
A	1106.2287	37.6041
A	1104.3842	1.3600
A	1078.4949	1.7807
A	1074.4340	1.7979
A	1069.1309	21.1355
A	1060.8606	3.7459
A	1057.0849	4.1685
A	1044.1506	6.5716
A	1029.5398	0.3107
A	1014.2818	2.9186
A	1008.8751	4.9297
A	1006.9675	1.3878
A	990.7991	7.9437
A	989.7658	2.1956
A	984.2203	3.5900
A	983.3867	0.8860
A	967.3999	2.6458
A	961.0372	12.5465
A	955.1306	0.9381
A	947.9595	5.9141
A	944.3481	5.5754
A	905.6844	10.3255
A	891.4468	5.9937
A	886.6517	18.3977
A	871.0212	5.8814
A	853.3249	7.0709
A	843.7376	9.5741
A	829.4524	7.7809
A	816.8734	13.7913
A	800.8051	7.2877
A	787.8724	24.6664
A	770.6555	4.3408
A	758.0735	12.9183
A	729.2820	33.5672
A	721.0989	5.5321
A	714.6810	10.6446
A	706.4211	13.3597
A	693.9228	9.1242
A	682.2622	4.7722
A	659.6415	120.9107
A	643.5713	96.1398
A	614.7520	26.8841
A	607.0181	15.3664
A	597.9600	3.6940
A	582.6150	1.0956
A	564.1908	53.7049
A	544.2486	46.0069
A	530.2875	3.7289

A	514.0210	13.2497
A	511.7776	9.8068
A	460.5094	8.1235
A	458.1200	1.4415
A	451.0753	2.6544
A	424.2720	1.0057
A	415.7735	2.8555
A	410.7379	2.7106
A	394.8037	3.6154
A	376.1378	5.2449
A	360.4471	1.7868
A	351.6048	13.7541
A	343.2438	3.8710
A	327.0373	1.7081
A	315.5712	5.3661
A	313.4863	3.8792
A	297.8127	2.6259
A	295.7861	5.8421
A	274.1009	3.3752
A	272.9895	7.0213
A	263.0154	1.1838
A	254.5071	3.8849
A	245.4366	10.2000
A	239.9013	6.1366
A	237.0925	6.2148
A	227.6393	0.0591
A	222.9849	2.5498
A	212.7470	1.0695
A	206.9206	1.4695
A	198.6589	0.3129
A	194.4173	0.6704
A	176.8932	1.8723
A	162.9617	1.9603
A	160.0793	2.4609
A	153.0392	2.9978
A	136.0921	4.7007
A	119.7413	4.2287
A	114.5190	3.2275
A	107.6672	1.0562
A	89.8436	5.2143
A	87.3159	6.2402
A	80.0350	4.1660
A	78.6434	4.7246
A	65.7079	1.6173
A	59.6760	0.4664
A	57.2237	0.5438
A	-55.8770	4.5258
A	50.4347	0.1758
A	46.0154	0.1900
A	24.2073	1.1336
A	21.3180	1.1117

A 6.1516 2.6850

67

C	3.711568	0.298500	-0.976819
C	3.085941	-1.101878	-1.067501
C	4.304761	-2.040426	-1.093301
C	5.431565	-1.172937	-1.693462
N	5.021219	0.174954	-1.311009
C	2.058003	-1.305694	0.035860
C	1.099355	-2.499266	-0.173442
C	1.715277	-3.753751	0.299340
N	2.258413	-4.711331	0.666763
O	3.145086	1.346549	-0.676765
N	-0.186065	-2.230263	0.460688
C	-1.314183	-2.173287	-0.331735
O	-1.420516	-2.777988	-1.386065
C	-2.398930	-1.233811	0.215711
C	-3.785944	-1.758214	-0.092712
C	-4.417679	-0.846464	-1.118539
C	-3.402059	0.214884	-1.477608
N	-2.365648	0.119618	-0.422360
C	-4.991954	-0.920669	0.282138
C	-6.296460	-1.692387	0.397212
C	-4.895671	0.236083	1.261254
C	-1.091324	0.659502	-0.759206
C	-0.745586	2.053162	-0.192863
C	-0.554510	3.171475	-1.255672
C	0.591770	2.874820	-2.238685
O	-0.246634	0.041350	-1.378842
N	0.451758	1.903656	0.646051
C	0.349103	1.181362	1.779219
C	1.605952	1.122950	2.672216
F	2.712098	1.596507	2.072953
O	-0.665034	0.615714	2.175197
C	-1.866016	3.309573	-2.047603
C	-0.275892	4.491344	-0.513785
F	1.393420	1.840368	3.787071
F	1.841031	-0.153484	3.035787
H	-6.341968	-2.520562	-0.318681
H	-7.155052	-1.036156	0.207655
H	-6.412016	-2.112364	1.403933
H	-3.939011	0.757832	1.197607
H	-5.699223	0.959341	1.072698
H	-5.017734	-0.128708	2.288585
H	-5.000301	-1.254838	-1.939222
H	-3.888723	-2.835676	-0.166747
H	-2.237568	-1.074404	1.286887
H	-3.819339	1.228538	-1.490334
H	-2.986885	-0.004799	-2.472272
H	-1.558221	2.342735	0.476463
H	1.375794	1.999589	0.221024

H	-1.800449	4.163106	-2.730549
H	-2.065938	2.420147	-2.654246
H	-2.723182	3.475259	-1.384423
H	-0.187651	5.312738	-1.233218
H	-1.088547	4.735122	0.180618
H	0.654856	4.444870	0.059489
H	0.659170	3.694401	-2.963316
H	1.563360	2.787974	-1.745268
H	0.418874	1.945457	-2.786288
H	-0.162728	-1.619134	1.269997
H	0.875435	-2.623666	-1.239131
H	1.438476	-0.412537	0.034215
H	2.549909	-1.380697	1.010240
H	4.564950	-2.328683	-0.069313
H	4.137410	-2.957259	-1.662158
H	5.484566	-1.272509	-2.786420
H	6.415236	-1.415164	-1.280761
H	5.601811	0.994912	-1.423768
H	2.562295	-1.126040	-2.034830

Conformer **TS2**: B3LYPD3BJ/6-31+G(d,p)

Processing: nir-tsd6pbed3j.log
PG=C01

WARNING: Imaginary frequencies

Method	BasisSet	Imaginary Freqs
RB3LYP	6-31+G(d,p)	1

HF Energy
-1770.1565340

ZPE	E298	S298	Squasihar	Equasihar	Strans	Srot
343.95459	365.996	218.987	201.091	366.085	44.511	36.925

Label	Frequencies	IR Inten	Dipole
A	3643.2612	45.8938	6.8581
A	3572.0603	66.2234	
A	3548.9418	152.9835	
A	3193.4742	5.7963	
A	3176.1742	13.7981	
A	3174.4004	0.6508	
A	3144.1175	6.0344	
A	3142.6255	15.0165	
A	3137.9345	13.0000	
A	3123.1577	48.2745	
A	3121.2147	15.5637	
A	3115.9744	25.4616	
A	3112.9979	34.4972	
A	3107.6615	2.8490	

A	3104.8266	28.1600
A	3101.8269	27.0425
A	3101.5572	8.0220
A	3089.8676	41.8102
A	3084.7991	5.7353
A	3083.2731	15.1387
A	3074.6101	25.0358
A	3072.4064	10.6038
A	3069.9069	13.1834
A	3068.4620	16.3736
A	3052.0080	19.3695
A	3042.2317	18.2606
A	3038.5121	25.4438
A	3031.3632	78.1014
A	3027.2859	63.2100
A	3025.6421	31.6003
A	3019.7754	6.2029
A	3009.8660	45.5105
A	2352.6397	7.8990
A	1776.6060	160.9069
A	1772.9065	546.7458
A	1766.0374	107.0116
A	1754.8799	422.6876
A	1570.5407	154.7790
A	1541.7572	176.4328
A	1536.8186	5.7284
A	1528.2812	21.3998
A	1522.5968	9.8105
A	1518.3803	13.2112
A	1515.9045	1.6895
A	1513.0908	1.7111
A	1512.5346	11.3863
A	1504.3252	4.0650
A	1497.6673	10.6233
A	1494.3672	1.2496
A	1493.6574	5.1606
A	1492.7345	0.6895
A	1491.4864	2.9347
A	1489.8393	0.7715
A	1456.3284	50.4466
A	1450.2890	7.2703
A	1449.7225	6.8117
A	1427.0661	2.4573
A	1420.4728	11.9465
A	1413.5963	6.0340
A	1412.0246	9.1599
A	1405.5055	7.7973
A	1404.8631	9.2124
A	1396.1641	7.6387
A	1393.5364	4.3266
A	1377.2802	5.6193

A	1366.7782	17.7834
A	1356.1034	2.8827
A	1345.0594	64.1263
A	1343.4534	3.4594
A	1334.3815	40.2031
A	1323.5255	1.8488
A	1320.8553	19.1068
A	1315.1046	6.1245
A	1301.3891	6.9442
A	1296.3512	128.1778
A	1277.3282	58.6070
A	1264.7763	22.9681
A	1255.8294	13.7051
A	1245.3924	25.1328
A	1241.5366	21.9058
A	1226.4042	54.5342
A	1224.2165	22.0533
A	1219.7679	37.9235
A	1214.5720	76.9677
A	1202.4150	188.9228
A	1201.2696	37.6524
A	1191.5772	114.2336
A	1172.9881	254.5579
A	1161.9613	305.7595
A	1159.5127	1.0012
A	1151.4825	18.8064
A	1138.3397	10.9136
A	1130.2001	16.1525
A	1123.8197	1.7433
A	1108.5583	8.2678
A	1099.3247	34.8401
A	1096.9999	14.7536
A	1072.4943	1.4162
A	1063.7858	5.7512
A	1059.6303	20.1727
A	1049.2499	4.2527
A	1046.1898	2.0387
A	1034.4392	5.7760
A	1020.9131	0.8340
A	1005.5416	2.8059
A	1002.3379	4.2475
A	995.9472	0.3949
A	982.4097	7.3683
A	978.6612	3.4568
A	974.5821	5.5001
A	974.0100	0.3338
A	959.4635	3.7639
A	952.8888	7.9477
A	947.3229	2.8551
A	941.3307	5.3061
A	935.7701	9.3265

A	900.0016	10.6310
A	884.7489	2.9482
A	878.1386	11.1425
A	866.4899	10.6976
A	849.0044	5.4227
A	838.7353	8.5300
A	823.1433	7.3099
A	813.8170	13.5047
A	796.3467	9.2154
A	783.2901	22.0945
A	767.4529	5.9354
A	757.3889	12.1268
A	721.7409	22.5657
A	714.7802	6.3250
A	707.9862	2.4312
A	701.3986	9.5833
A	689.4029	7.7099
A	678.0104	5.5092
A	638.0748	47.1763
A	611.2356	24.3468
A	603.0651	14.2670
A	593.2195	7.0930
A	580.0950	18.5024
A	568.0949	103.0848
A	557.3955	56.3821
A	534.6479	49.2949
A	526.0933	16.2105
A	510.4096	23.6689
A	508.0319	8.7531
A	460.0024	8.6575
A	455.5270	2.6253
A	452.7285	1.9642
A	421.3201	0.9407
A	413.2727	1.6460
A	410.1165	4.5264
A	396.4917	3.7352
A	376.9909	4.8693
A	352.1697	14.3491
A	348.1168	1.8944
A	341.4937	3.9988
A	327.8681	1.8508
A	312.1881	0.8181
A	308.0794	5.5828
A	299.2889	2.3742
A	294.3473	6.3281
A	273.2500	3.8150
A	270.6058	7.5973
A	262.2553	1.4515
A	251.7989	3.3127
A	244.9550	6.3010
A	239.9167	7.8927

A	231.7938	7.5424
A	224.4996	0.1606
A	221.9568	1.8640
A	206.8065	0.8601
A	202.3808	1.6429
A	192.2186	1.0540
A	190.7501	0.5089
A	175.4450	2.6779
A	159.0377	2.0716
A	156.3691	2.1303
A	151.0483	2.7411
A	127.1422	6.3512
A	113.9816	3.0986
A	107.5229	1.5926
A	103.2450	2.0532
A	87.9885	6.6492
A	85.8892	6.7679
A	73.4831	1.4693
A	69.8012	8.3655
A	62.6879	0.2437
A	60.4380	0.3981
A	-57.8871	4.2681
A	53.6996	0.3120
A	46.9845	0.1106
A	45.5643	0.8221
A	27.6705	2.1877
A	19.3964	0.7263
A	12.5549	3.2699

67

C	3.756261	0.142277	-1.049660
C	3.137516	-1.264736	-1.030626
C	4.359283	-2.192413	-0.905486
C	5.509055	-1.381557	-1.540316
N	5.080406	-0.002060	-1.312826
C	2.056814	-1.352636	0.038343
C	1.084590	-2.544144	-0.092366
C	1.685691	-3.784245	0.437029
N	2.214743	-4.732736	0.847278
O	3.165586	1.206432	-0.873266
N	-0.180974	-2.218879	0.555771
C	-1.336898	-2.217191	-0.189346
O	-1.480389	-2.889125	-1.201417
C	-2.407013	-1.246576	0.337651
C	-3.796935	-1.803274	0.111914
C	-4.448241	-1.002542	-0.993755
C	-3.449425	0.034247	-1.457835
N	-2.408724	0.058245	-0.397697
C	-5.011878	-0.948816	0.412945
C	-6.302321	-1.728202	0.613980
C	-4.934962	0.300633	1.273113

C	-1.142149	0.598917	-0.778657
C	-0.810024	2.019195	-0.271580
C	-0.714468	3.106817	-1.383917
C	0.430885	2.834799	-2.376336
O	-0.310224	-0.024358	-1.408234
N	0.436699	1.970304	0.500611
C	0.473269	1.310217	1.669129
C	1.756227	1.506021	2.516656
F	2.723679	2.189351	1.871999
O	-0.436784	0.630495	2.135367
C	-2.045845	3.138402	-2.153387
C	-0.502301	4.471033	-0.699782
F	1.442847	2.192520	3.634498
F	2.266984	0.315950	2.891681
H	-6.337011	-2.622963	-0.015996
H	-7.171877	-1.107670	0.366389
H	-6.403156	-2.047735	1.657916
H	-3.997068	0.841079	1.139554
H	-5.763943	0.976350	1.029639
H	-5.027884	0.033931	2.332518
H	-5.027365	-1.497018	-1.767772
H	-3.891071	-2.883349	0.144956
H	-2.206257	-1.016414	1.389192
H	-3.884587	1.034110	-1.560189
H	-3.031933	-0.273132	-2.427207
H	-1.601464	2.300884	0.426859
H	1.320481	2.195084	0.050169
H	-2.044304	3.975093	-2.858618
H	-2.198227	2.223526	-2.733131
H	-2.898825	3.269516	-1.478746
H	-0.479091	5.263775	-1.454286
H	-1.315445	4.693285	0.000107
H	0.439966	4.509986	-0.146180
H	0.440488	3.626657	-3.132660
H	1.416054	2.823873	-1.901454
H	0.304428	1.875263	-2.881821
H	-0.133417	-1.558252	1.324075
H	0.842318	-2.723860	-1.146176
H	1.452238	-0.454749	-0.072807
H	2.507369	-1.345446	1.035571
H	4.573833	-2.372726	0.152956
H	4.221042	-3.162879	-1.385055
H	5.607017	-1.588190	-2.614404
H	6.473479	-1.575089	-1.063461
H	5.665079	0.805878	-1.475218
H	2.664193	-1.388236	-2.016151

Conformer **TS2**: B3LYPD3BJ-SMD/6-31+G(d,p), in acetonitrile solvent

Processing: nir-tsd6pbed3jacs.log
PG=C01

WARNING: Imaginary frequencies

Method	BasisSet	Imaginary Freqs
RB3LYP	6-31+G(d,p)	1

HF Energy
-1770.2019702

ZPE	E298	S298	Squasihar	Equasihar	Strans	Srot
342.67023	364.885	221.236	202.385	364.979	44.511	37.148

Label	Frequencies	IR Inten	Dipole
A	3618.9445	104.6780	7.5639
A	3618.7641	159.1650	
A	3554.0349	254.0949	
A	3190.0186	14.9897	
A	3175.4475	25.3678	
A	3143.7403	12.3989	
A	3142.5870	35.8213	
A	3134.5396	4.9135	
A	3130.5554	69.0471	
A	3128.5564	8.7288	
A	3116.2910	88.4897	
A	3112.7429	42.0412	
A	3112.5316	70.2324	
A	3111.0337	23.1687	
A	3106.6497	10.6025	
A	3105.4153	28.6157	
A	3103.0084	66.1930	
A	3101.4999	44.8157	
A	3090.1009	19.7130	
A	3083.1051	82.8622	
A	3078.0195	50.2405	
A	3076.5651	25.7230	
A	3076.3405	22.0363	
A	3066.6310	34.2752	
A	3049.3796	10.4900	
A	3043.2659	57.6628	
A	3038.7891	84.8044	
A	3038.2565	44.7629	
A	3027.6400	108.3963	
A	3027.3383	21.6608	
A	3021.9201	44.7652	
A	3012.9328	119.6918	
A	2345.2770	49.1867	
A	1759.0711	299.2996	
A	1741.9571	690.6695	
A	1725.8238	772.8785	
A	1719.8787	552.2509	
A	1582.8603	226.6134	

A	1548.6841	399.7593
A	1518.5716	13.7387
A	1517.5279	14.7924
A	1505.6212	10.7622
A	1500.1920	21.2013
A	1497.3632	2.9969
A	1497.0789	3.8317
A	1494.0344	15.0647
A	1487.9266	17.5245
A	1483.9062	3.0630
A	1483.3295	16.7688
A	1479.0396	1.7767
A	1477.7782	9.8869
A	1474.9913	3.0039
A	1474.5559	0.2229
A	1456.5544	72.6299
A	1439.3017	2.3086
A	1433.3374	11.0825
A	1414.6844	2.1264
A	1412.5524	16.0195
A	1407.5656	10.0370
A	1404.9747	10.9427
A	1398.9115	23.3602
A	1397.2399	14.3371
A	1379.6741	29.2098
A	1373.2598	3.2307
A	1369.1810	19.4183
A	1364.2344	19.2766
A	1347.7358	76.4105
A	1341.7313	3.6287
A	1330.4802	29.0148
A	1326.8095	40.8735
A	1317.8444	27.9205
A	1315.6870	5.1366
A	1308.6308	1.1478
A	1299.2910	24.0133
A	1288.5001	232.2751
A	1273.2766	5.4924
A	1255.4318	31.2604
A	1251.0948	57.0989
A	1245.7926	60.9338
A	1238.9414	33.6760
A	1231.5406	131.2636
A	1219.0937	49.5385
A	1217.7619	1.9309
A	1215.5626	7.5256
A	1206.2890	277.3296
A	1195.6196	5.3534
A	1173.1788	428.1082
A	1158.7252	385.8286
A	1152.6437	10.9429

A	1138.6978	16.1774
A	1131.6847	56.0908
A	1128.1472	18.8364
A	1117.9067	5.3480
A	1111.6504	313.9344
A	1106.0622	99.9163
A	1097.5941	136.5851
A	1094.3559	22.5299
A	1072.5037	10.0908
A	1059.8154	24.6904
A	1058.6230	33.1490
A	1044.5836	3.2459
A	1042.2852	2.8633
A	1035.2106	5.4267
A	1020.2389	1.4681
A	1004.3215	1.8572
A	998.7941	10.3951
A	992.7483	4.1966
A	978.4167	10.5724
A	977.9101	1.1610
A	973.3430	16.4967
A	970.6588	4.5610
A	960.7460	6.8538
A	955.4164	10.0190
A	946.6256	10.7878
A	941.1535	7.2889
A	935.5147	25.2936
A	894.3348	19.5251
A	878.9091	7.5663
A	872.3601	3.3134
A	871.1886	35.6228
A	843.8612	11.4255
A	832.6582	12.7982
A	818.3442	1.5426
A	810.9991	31.6789
A	795.6906	11.9352
A	777.8181	34.4682
A	767.4280	10.2223
A	760.6479	16.1356
A	715.2947	31.2169
A	711.4516	13.0741
A	704.6841	4.1991
A	695.8728	0.5734
A	685.3455	11.6400
A	671.9423	12.8024
A	631.2624	45.4255
A	602.1405	59.2745
A	597.7541	38.6125
A	585.0759	106.0087
A	575.0619	26.6868
A	561.3503	61.3137

A	533.4133	21.4460
A	532.4974	117.7535
A	523.0141	26.3611
A	508.0117	64.0468
A	492.9951	103.5271
A	467.0370	19.5707
A	452.8848	5.8721
A	451.9735	6.5622
A	421.5922	1.9759
A	413.9455	14.6388
A	411.2605	4.2622
A	401.5293	4.0343
A	380.1106	15.8182
A	360.5681	31.9644
A	352.5431	1.0684
A	344.6170	7.2899
A	340.6592	3.4792
A	320.6402	2.9098
A	305.9890	11.1622
A	301.7102	5.7944
A	292.3779	7.2162
A	273.3224	4.3347
A	264.4190	15.4300
A	260.3544	1.8944
A	251.6012	2.8562
A	243.4056	14.1777
A	235.7738	5.7290
A	224.1568	2.1448
A	216.8994	5.1313
A	208.9355	16.0128
A	203.9443	3.4500
A	198.0180	4.0741
A	185.3960	2.0503
A	183.2475	2.3798
A	172.9807	5.8976
A	155.2825	9.3565
A	144.0526	2.2702
A	140.1422	2.8778
A	123.9025	17.7801
A	113.0589	2.6731
A	108.0959	8.1163
A	102.0262	8.1935
A	85.9232	14.4946
A	79.4413	7.1329
A	75.1204	6.2785
A	66.5779	0.1703
A	-65.1476	6.6703
A	61.4048	0.2083
A	54.4845	1.3640
A	47.8422	1.2850
A	41.8219	2.3046

A	37.6998	3.3043	
A	23.9277	3.9595	
A	19.6777	5.9390	
A	17.2756	0.9511	

67

C	4.394879	0.115102	-1.410877
C	3.789838	-1.231125	-0.988714
C	4.942975	-1.913311	-0.226485
C	6.214234	-1.279563	-0.821564
N	5.735527	0.034264	-1.249983
C	2.474728	-1.030407	-0.239794
C	1.618126	-2.301339	-0.086036
C	2.250538	-3.314446	0.784044
N	2.739471	-4.105178	1.479776
O	3.771644	1.092805	-1.837616
N	0.301339	-1.975878	0.437213
C	-0.835881	-2.235328	-0.265147
O	-0.860270	-2.950226	-1.268854
C	-2.089164	-1.558857	0.322098
C	-3.285105	-2.481265	0.187625
C	-4.176883	-1.924951	-0.903312
C	-3.528126	-0.660049	-1.423385
N	-2.504946	-0.324177	-0.401230
C	-4.677994	-1.987811	0.527111
C	-5.684884	-3.094747	0.800171
C	-4.932552	-0.745091	1.361815
C	-1.468630	0.591953	-0.760732
C	-1.565752	2.006755	-0.147049
C	-1.999840	3.137877	-1.134743
C	-0.987850	3.333703	-2.276760
O	-0.510450	0.279906	-1.441746
N	-0.268317	2.339194	0.447736
C	0.235942	1.638402	1.461537
C	1.545809	2.201809	2.066969
F	2.233185	2.987563	1.206584
O	-0.266147	0.634970	1.970757
C	-3.367724	2.782119	-1.737241
C	-2.143128	4.443251	-0.328505
F	1.260296	2.949602	3.160220
F	2.360703	1.207703	2.458434
H	-5.486225	-3.981582	0.189179
H	-6.703769	-2.753049	0.579232
H	-5.653390	-3.396306	1.854611
H	-4.206640	0.045408	1.168043
H	-5.935418	-0.351959	1.153192
H	-4.893248	-0.994999	2.429495
H	-4.632129	-2.579193	-1.640967
H	-3.082751	-3.544611	0.260599
H	-1.883413	-1.278985	1.359925
H	-4.231844	0.171093	-1.517569

H	-3.079662	-0.850564	-2.408594
H	-2.302019	1.958706	0.658641
H	0.268363	3.110200	0.069899
H	-3.754473	3.644603	-2.289180
H	-3.293739	1.949987	-2.442523
H	-4.094213	2.521608	-0.960469
H	-2.507290	5.238008	-0.987592
H	-2.861813	4.320970	0.489377
H	-1.192826	4.779727	0.098294
H	-1.356641	4.118647	-2.945313
H	-0.001439	3.650902	-1.921082
H	-0.859948	2.420209	-2.863279
H	0.256634	-1.295642	1.190563
H	1.468028	-2.768889	-1.064904
H	1.856916	-0.320709	-0.795984
H	2.668048	-0.602635	0.748691
H	4.871646	-1.665727	0.838208
H	4.945146	-2.999462	-0.326189
H	6.589269	-1.851539	-1.679625
H	7.020114	-1.180131	-0.090551
H	6.351572	0.759708	-1.596628
H	3.588435	-1.770894	-1.925693

Conformer **X**: B3LYPD3BJ/6-31G(d)

Processing: nir-aa6dbed3j.log
PG=C01

Method	BasisSet	Imaginary Freqs
RB3LYP	6-31G(d)	0

HF Energy
-1770.0736332

ZPE	E298	S298	Squasihar	Equasihar	Strans	Srot
346.72463	369.201	224.489	203.657	369.303	44.511	37.039

67

C	-4.576839	-2.121849	-0.088028
C	-3.067549	-2.222075	-0.189758
C	-2.194644	-1.020944	0.157166
N	-2.718086	0.063077	-0.671970
C	-3.528122	-0.436996	-1.798267
C	-3.887667	-1.854605	-1.409786
C	-0.717400	-1.310735	-0.202888
O	-0.132627	-0.698323	-1.085894
C	-2.140009	1.290982	-0.806486
O	-2.356180	2.000695	-1.787075
C	-5.294558	-3.458252	0.009961
C	-5.260241	-1.019101	0.698785

N	-0.134980	-2.301422	0.525620
C	1.284066	-2.613824	0.339819
C	1.529976	-3.996367	0.766806
N	1.736627	-5.072383	1.149358
C	2.179182	-1.609170	1.093257
C	3.677419	-1.734152	0.812441
C	4.040121	-1.646586	-0.678786
N	5.157134	-0.875160	-0.775637
C	5.676666	-0.363078	0.482511
C	4.494028	-0.591270	1.449692
O	3.463690	-2.189439	-1.612510
C	-1.258995	1.822710	0.330559
N	-0.054600	2.331196	-0.315730
C	1.170673	2.036038	0.146328
O	1.432852	1.518040	1.226232
C	-1.985019	2.901774	1.199211
C	-2.276649	4.176398	0.392521
C	-3.302807	2.308263	1.724371
C	-1.075484	3.249246	2.390162
C	2.331561	2.433817	-0.786255
F	3.149999	1.384067	-0.967306
F	1.908018	2.851587	-1.998920
F	3.047920	3.428491	-0.233407
H	-4.780811	-4.237321	-0.563862
H	-6.317569	-3.376542	-0.376484
H	-5.355847	-3.793290	1.052570
H	-4.743604	-0.061570	0.622268
H	-6.286331	-0.879385	0.338088
H	-5.319532	-1.288994	1.760498
H	-4.024816	-2.567392	-2.217271
H	-2.606122	-3.200480	-0.099846
H	-2.260185	-0.758539	1.221158
H	-4.388915	0.223453	-1.941080
H	-2.936051	-0.418093	-2.720079
H	-0.935698	1.027618	1.002711
H	-0.169476	2.645031	-1.272808
H	-3.773210	3.004113	2.427298
H	-4.014477	2.123537	0.913481
H	-3.136814	1.363382	2.256991
H	-1.568503	3.989398	3.030616
H	-0.850370	2.365213	2.996324
H	-0.123826	3.672321	2.056894
H	-2.781145	4.909072	1.032608
H	-1.347810	4.631741	0.033249
H	-2.910009	3.971363	-0.473223
H	-0.612055	-2.678741	1.333578
H	1.510132	-2.533547	-0.727518
H	1.999790	-1.702721	2.171406
H	1.844607	-0.614379	0.799279
H	3.873346	0.308949	1.487933
H	4.829984	-0.818110	2.464553

H 6.574930 -0.914491 0.794247
H 5.939745 0.696183 0.397364
H 5.605415 -0.738821 -1.670864
H 4.041001 -2.712550 1.159085

Conformer **X**: B3LYPD3BJ/6-31+G(d,p)

Processing: nir-aa6pbed3j.log
PG=C01

Method	BasisSet	Imaginary Freqs
RB3LYP	6-31+G(d,p)	0

HF Energy
-1770.1920545

ZPE	E298	S298	Squasihar	Equasihar	Strans	Srot
344.26996	366.950	229.555	204.556	367.059	44.511	37.105

67
C 4.595242 2.123321 -0.094055
C 3.092206 2.209955 -0.276160
C 2.207273 1.027415 0.105698
N 2.748804 -0.097245 -0.655310
C 3.642978 0.331325 -1.748899
C 3.974155 1.773155 -1.430246
C 0.741671 1.310865 -0.296656
O 0.195814 0.723609 -1.220576
C 2.200395 -1.339367 -0.725888
O 2.496631 -2.126515 -1.627204
C 5.307635 3.465170 -0.036403
C 5.235407 1.068055 0.788938
N 0.127499 2.271947 0.447719
C -1.279507 2.608834 0.229894
C -1.474977 4.040968 0.498279
N -1.637290 5.159640 0.763799
C -2.205102 1.731947 1.098536
C -3.700906 1.902588 0.826826
C -4.105273 1.602398 -0.624540
N -5.254536 0.874602 -0.585081
C -5.792964 0.645307 0.749966
C -4.572326 0.932533 1.650222
O -3.522227 1.969617 -1.639358
C 1.232034 -1.791649 0.375423
N 0.086501 -2.366237 -0.322784
C -1.177203 -2.069892 0.013887
O -1.542168 -1.439609 1.000387
C 1.890368 -2.789669 1.387692
C 2.255171 -4.125894 0.721449
C 3.157922 -2.138080 1.966395
C 0.887827 -3.045664 2.527015

C	-2.258196	-2.639892	-0.936520
F	-3.133433	-1.682946	-1.279270
F	-1.737802	-3.161114	-2.075156
F	-2.940781	-3.628293	-0.315515
H	4.822772	4.208369	-0.677470
H	6.347478	3.363250	-0.367506
H	5.317025	3.857710	0.987145
H	4.732788	0.102562	0.730787
H	6.281605	0.920302	0.498219
H	5.226488	1.392226	1.836319
H	4.147002	2.437509	-2.271007
H	2.625671	3.189692	-0.267289
H	2.249984	0.817458	1.182470
H	4.512422	-0.332129	-1.781681
H	3.121362	0.248006	-2.708196
H	0.850214	-0.950996	0.954256
H	0.283520	-2.802279	-1.216942
H	3.575108	-2.772119	2.754823
H	3.929664	-2.007101	1.202527
H	2.942394	-1.159122	2.410394
H	1.332864	-3.720444	3.265797
H	0.608451	-2.116121	3.033326
H	-0.029668	-3.510684	2.157543
H	2.705246	-4.791629	1.465579
H	1.364513	-4.624367	0.326623
H	2.960830	-3.990856	-0.100600
H	0.592274	2.644735	1.264604
H	-1.509714	2.417018	-0.822859
H	-2.004662	1.939876	2.156946
H	-1.921834	0.694524	0.911157
H	-4.015283	0.004660	1.814900
H	-4.862228	1.336839	2.622883
H	-6.625825	1.329844	0.962051
H	-6.156007	-0.380833	0.857145
H	-5.741277	0.643501	-1.439604
H	-3.999251	2.943629	1.017628
