# Supplementary material for: A sustainable synthesis of the SARS-CoV-2 Mpro inhibitor nirmatrelvir, the active ingredient in Paxlovid
Source: Commun Chem. 2022 Nov 21;5:156. doi: 10.1038/s42004-022-00758-5 (PMC9685088; doi:10.1038/s42004-022-00758-5)
Supplement: Supplementary file 3 — Supplementary Data 2 [file 42004_2022_758_MOESM3_ESM.pdf]

A Sustainable Synthesis of the SARS-CoV-2 M^{pro} Inhibitor Nirmatrelvir, the Active Ingredient in Paxlovid

Supplementary Data 2

^{1}H, ^{13}C, ^{19}F NMR Spectra of synthesized products

Joseph R. A. Kincaid, Juan C. Caravez, Karthik S. Iyer, Rahul D. Kavthe, Nico Fleck, Donald H. Aue, and Bruce H. Lipshutz*

Department of Chemistry and Biochemistry, University of California, Santa Barbara, Santa Barbara, CA
93106 USA

Phone : 805-893-2521
Fax : 805-893-8265
Email: lipshutz@chem.ucsb.edu
Website: https://lipshutz.chem.ucsb.edu/

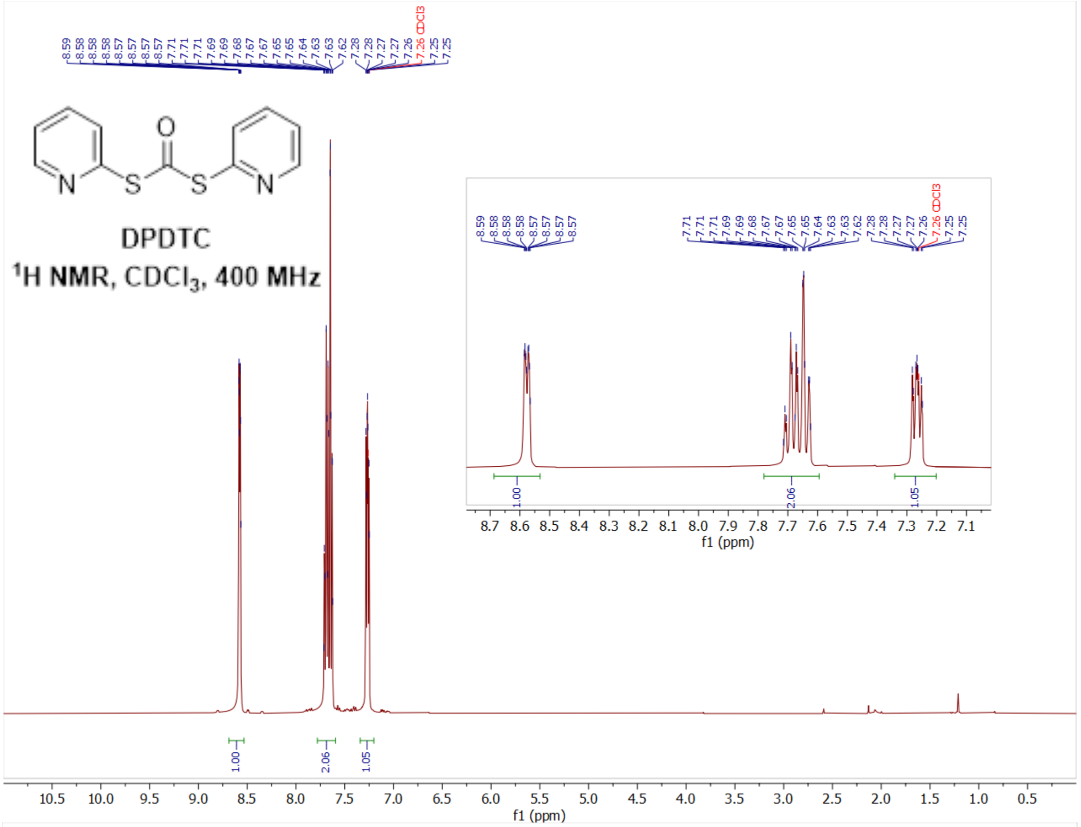
DPDTC
^{1}H NMR, $CDCl_3$, 400 MHz
f1 (ppm)

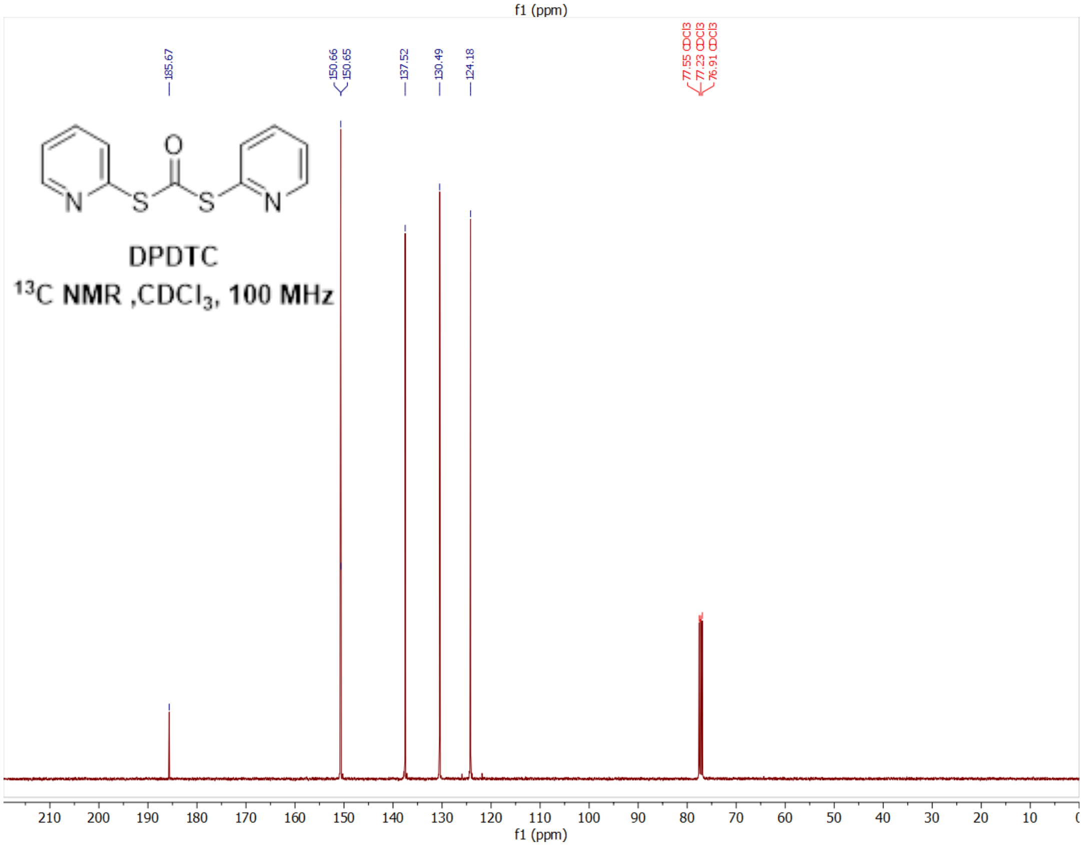
DPDTC
^{13}C NMR ,$CDCl_3$, 100 MHz
185.67
150.66
150.65
137.52
130.49
124.18
77.55 CDCl3
77.23 CDCl3
76.91 CDCl3
f1 (ppm)

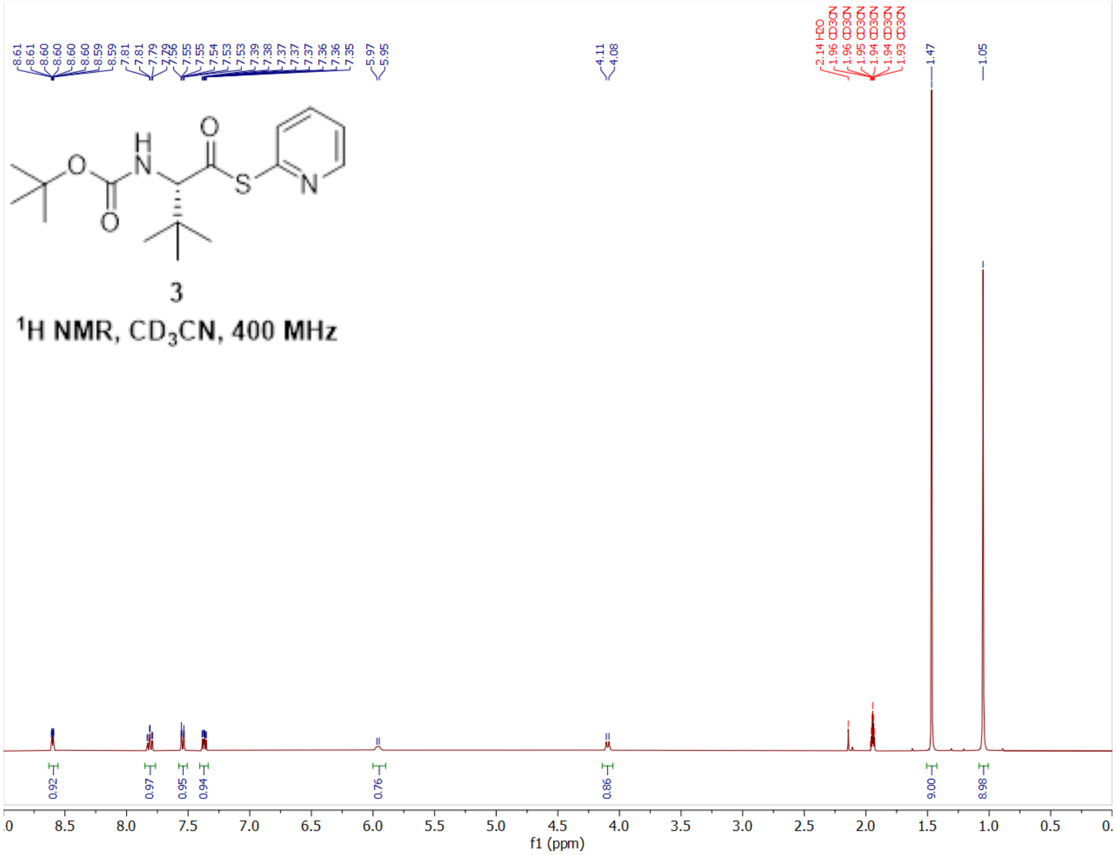
8.61 8.61 8.60 8.60 8.60 8.60 8.59 8.59 7.81 7.81 7.79 7.79 7.56 7.55 7.55 7.54 7.53 7.53 7.39 7.38 7.37 7.37 7.37 7.36 7.36 7.35 5.97 5.95
4.11 4.08
2.14 H2O 1.96 CD3CN 1.96 CD3CN 1.95 CD3CN 1.94 CD3CN 1.94 CD3CN 1.93 CD3CN
1.47
1.05
O
H
N
O
S
N
O
3
^{1}H NMR, CD_3CN, 400 MHz
0.92 0.97 0.95 0.94 0.76 0.86 9.00 8.98
f1 (ppm)

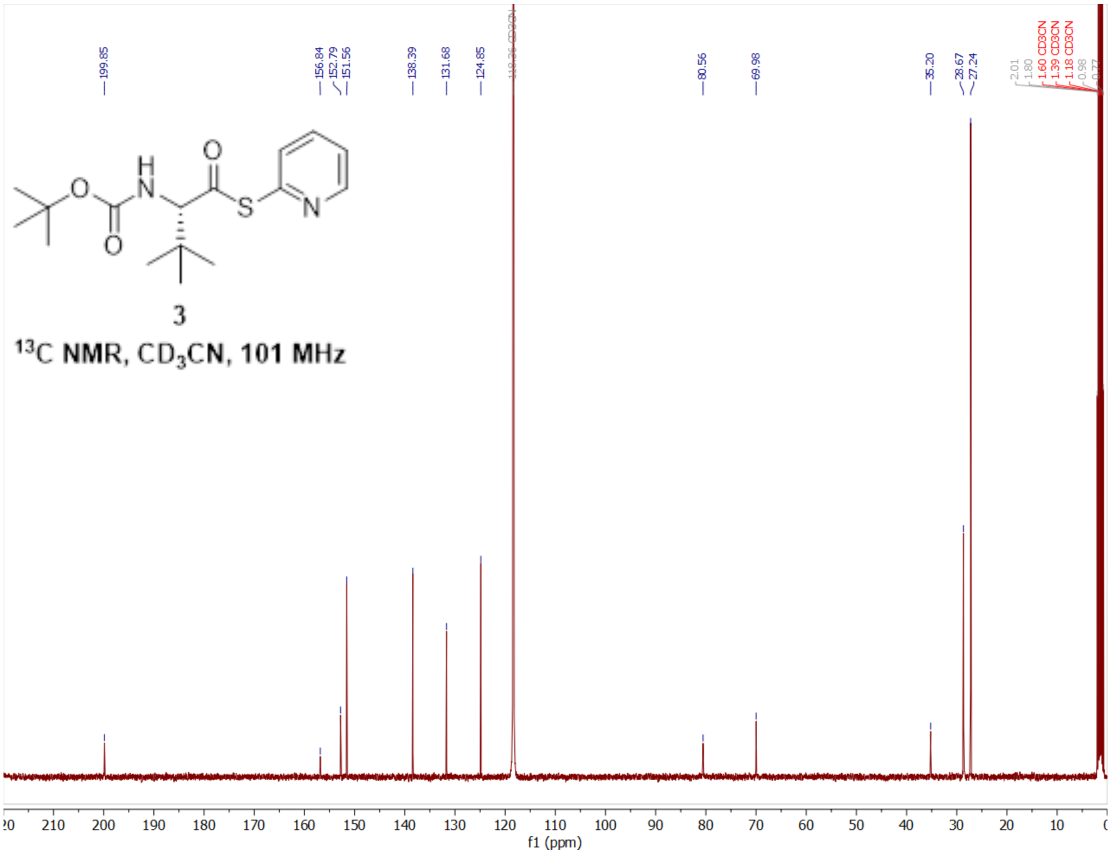
199.85
156.84 152.79 151.56
138.39
131.68
124.85
80.56
69.98
35.20
28.67 27.24
2.01 1.80 1.60 CD3CN 1.39 CD3CN 1.18 CD3CN 0.98 0.77
O
H
N
O
S
N
O
3
^{13}C NMR, CD_3CN, 101 MHz
f1 (ppm)

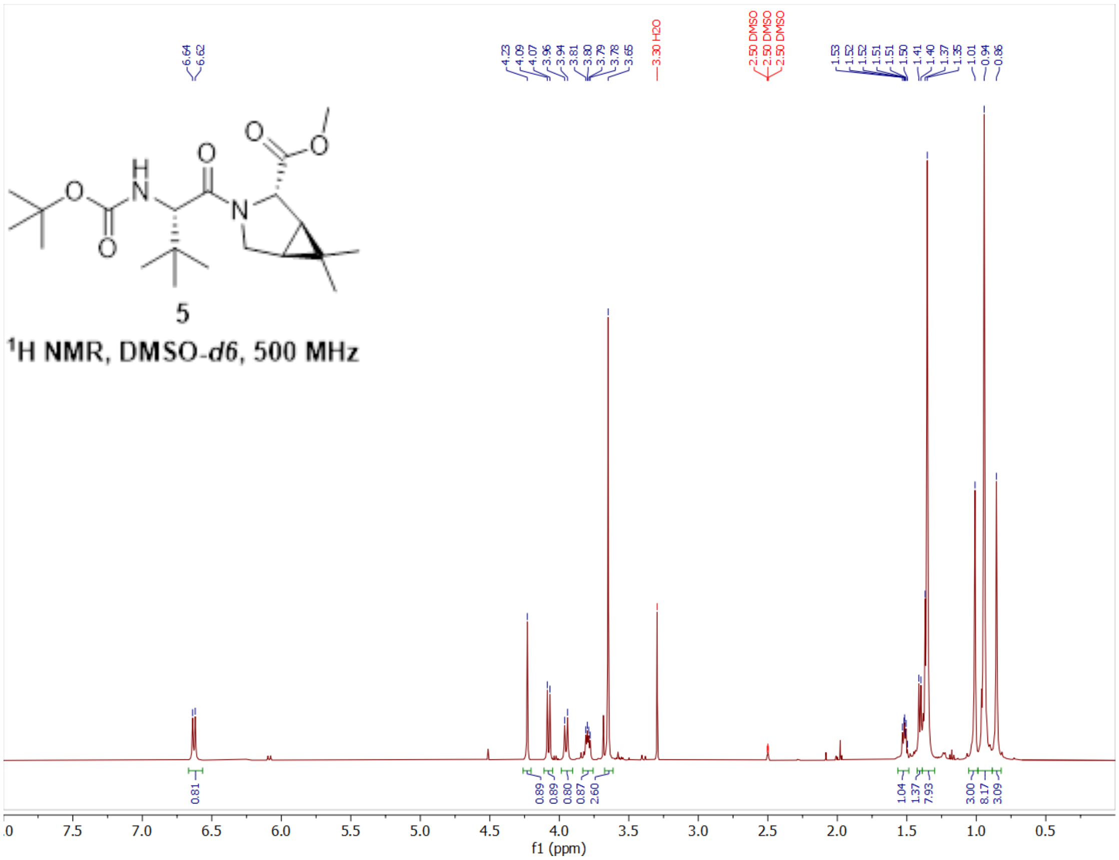

5
¹H NMR, DMSO-d6, 500 MHz
f1 (ppm)

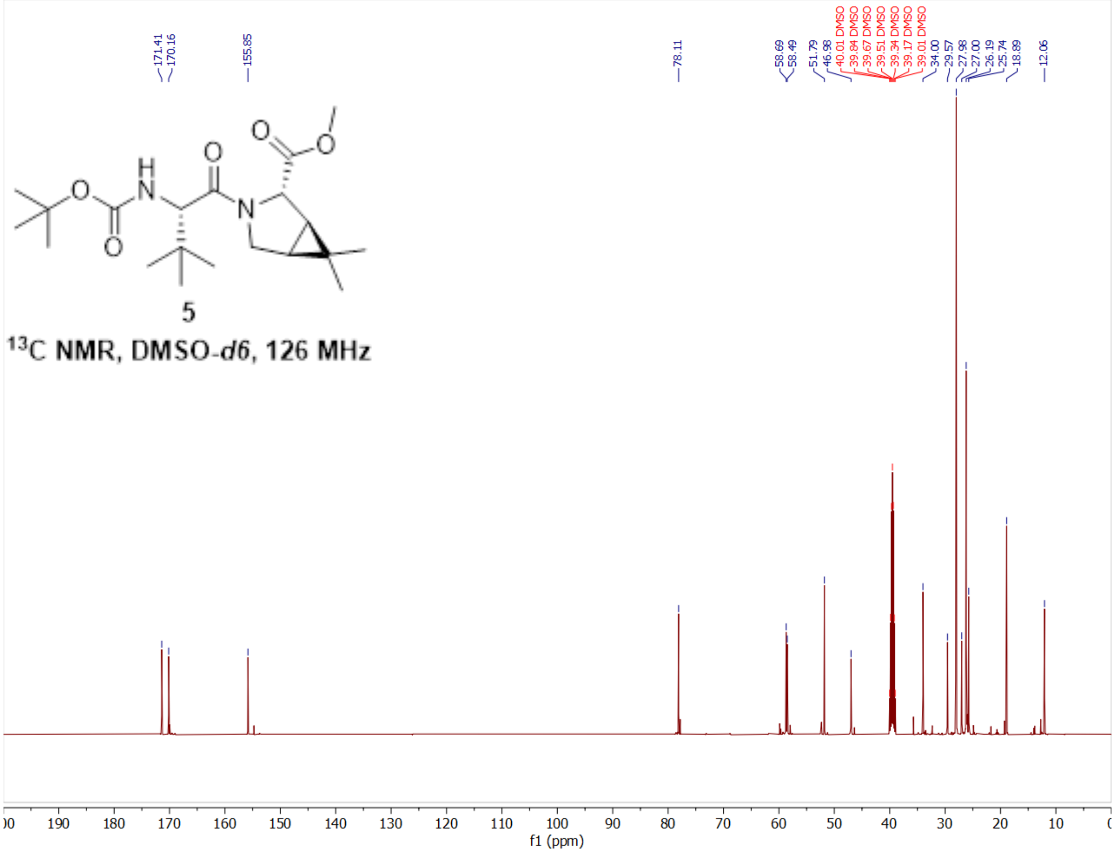

5
¹³C NMR, DMSO-d6, 126 MHz
f1 (ppm)

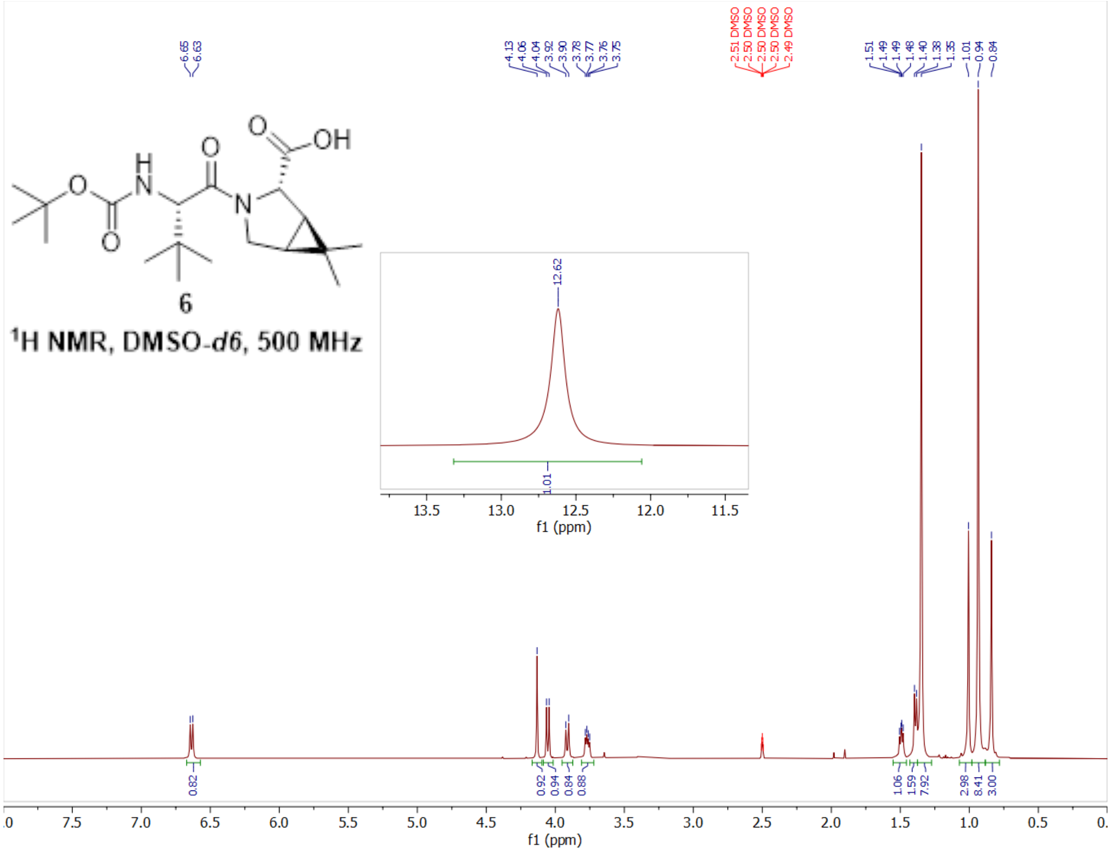
6
¹H NMR, DMSO-d6, 500 MHz
f1 (ppm)

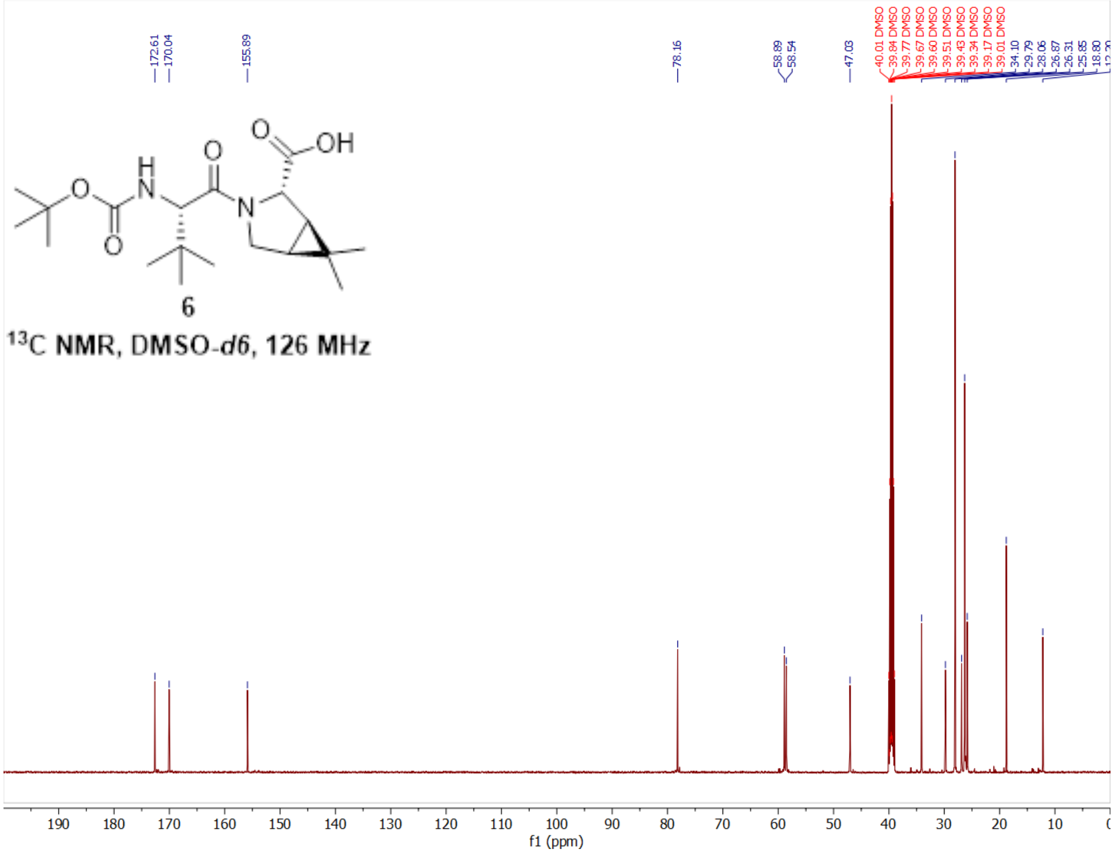
6
¹³C NMR, DMSO-d6, 126 MHz
f1 (ppm)

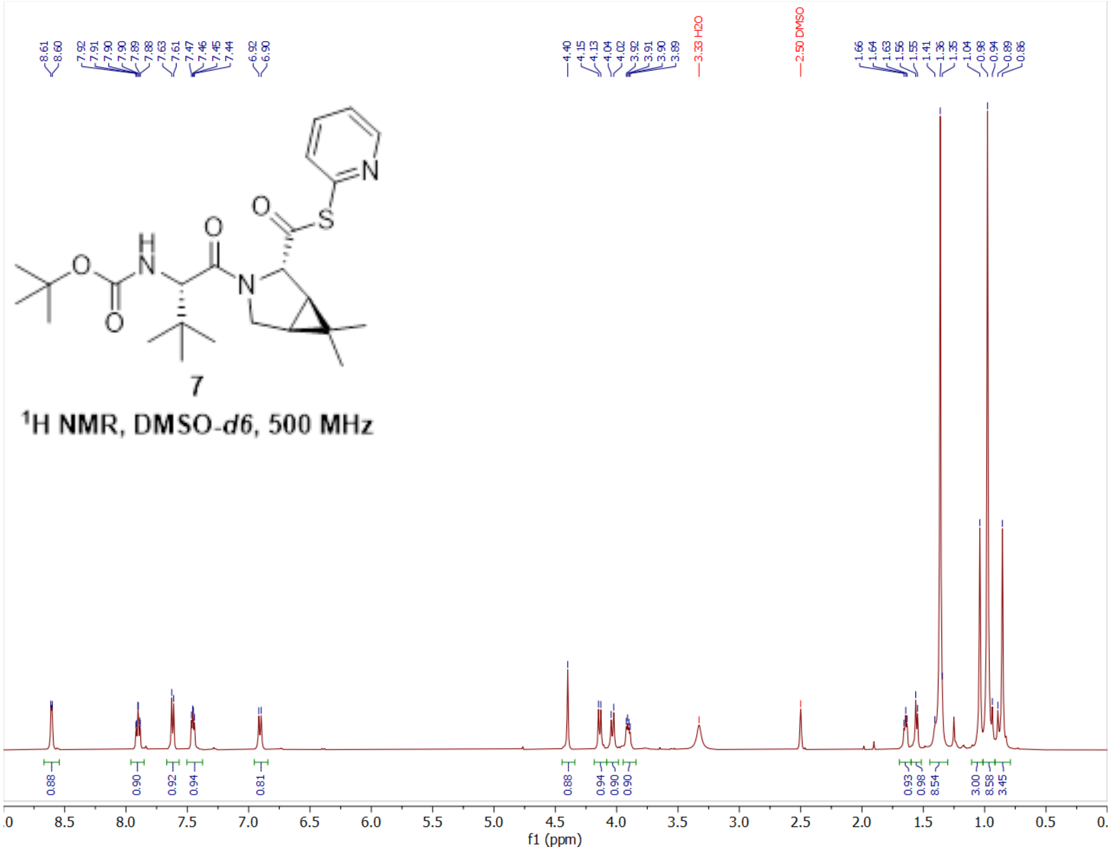
7
¹H NMR, DMSO-d6, 500 MHz
f1 (ppm)

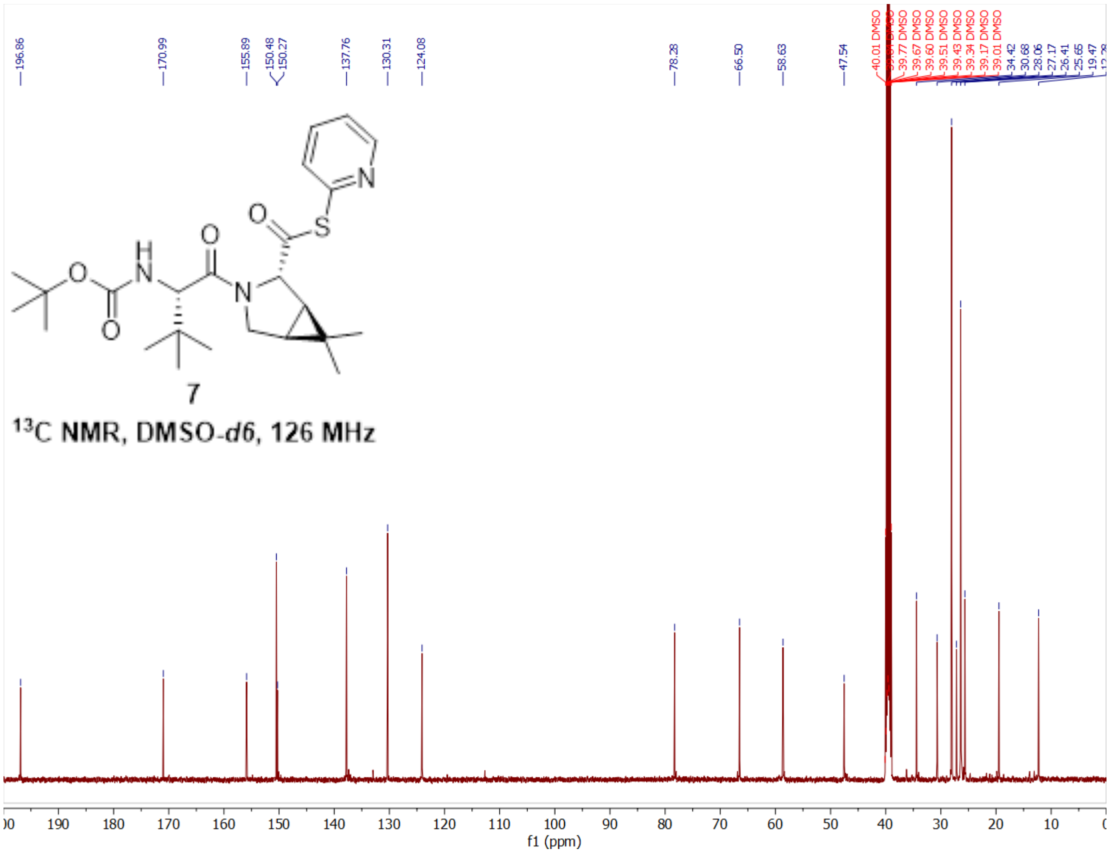
7
¹³C NMR, DMSO-d6, 126 MHz
f1 (ppm)

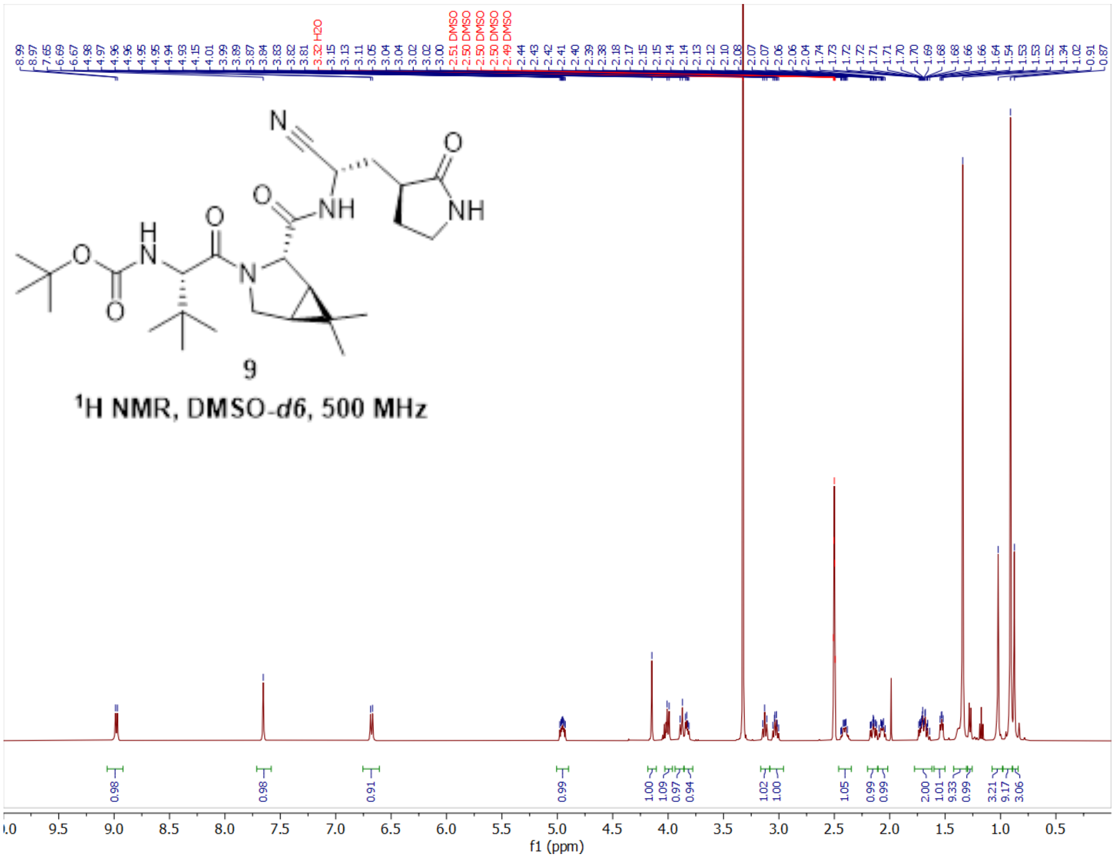
9
¹H NMR, DMSO-d6, 500 MHz
f1 (ppm)

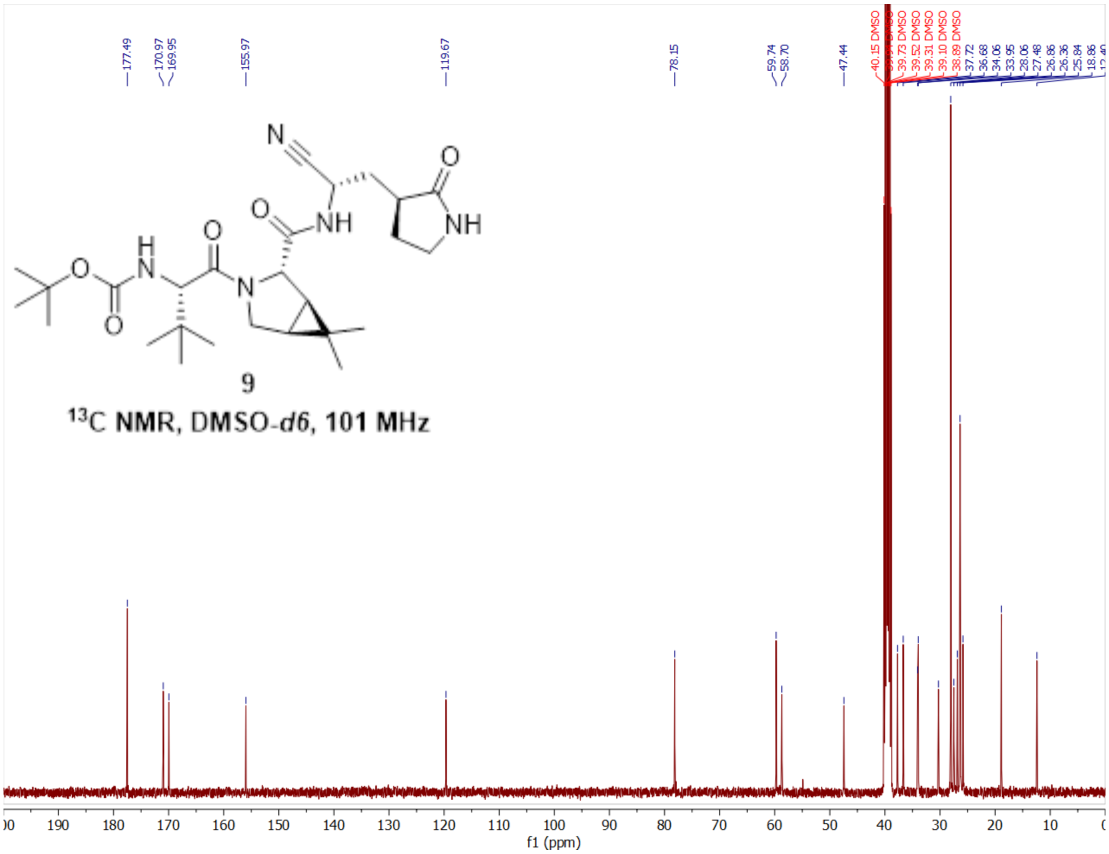
9
¹³C NMR, DMSO-d6, 101 MHz
f1 (ppm)

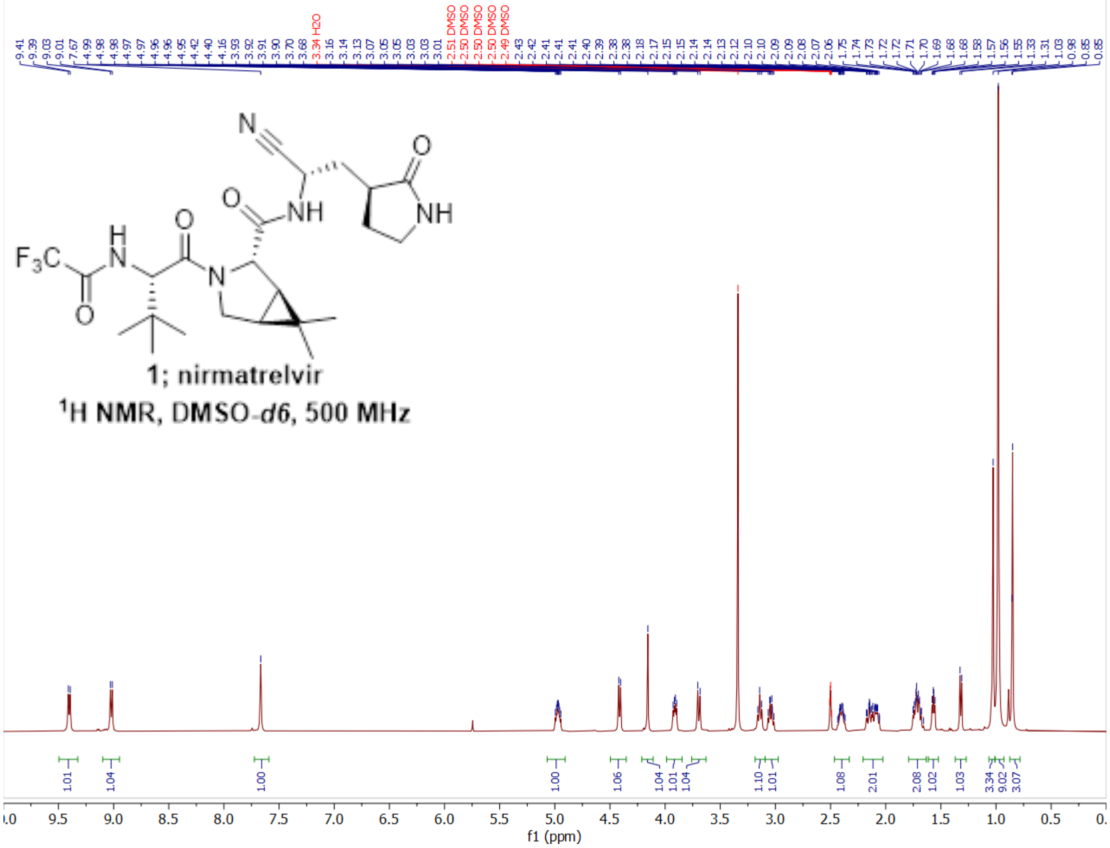
1; nirmatrelvir
¹H NMR, DMSO-d6, 500 MHz
f1 (ppm)

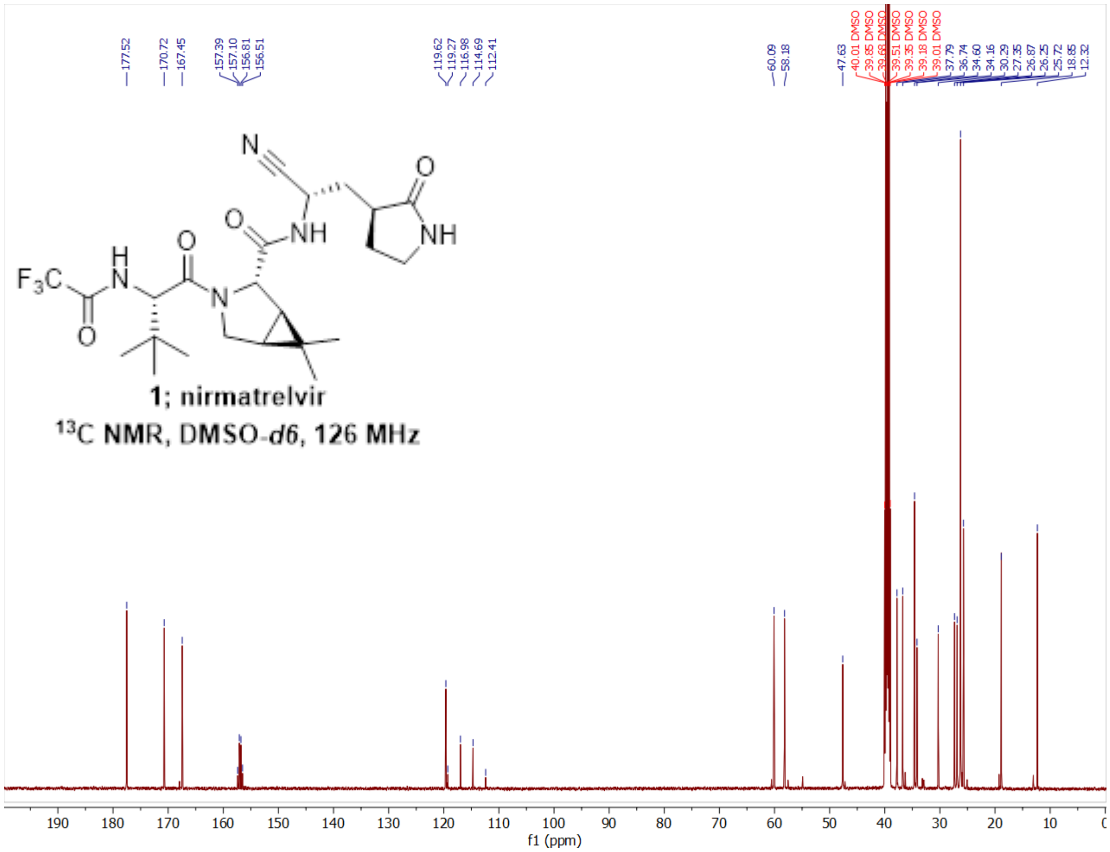
1; nirmatrelvir
¹³C NMR, DMSO-d6, 126 MHz
f1 (ppm)

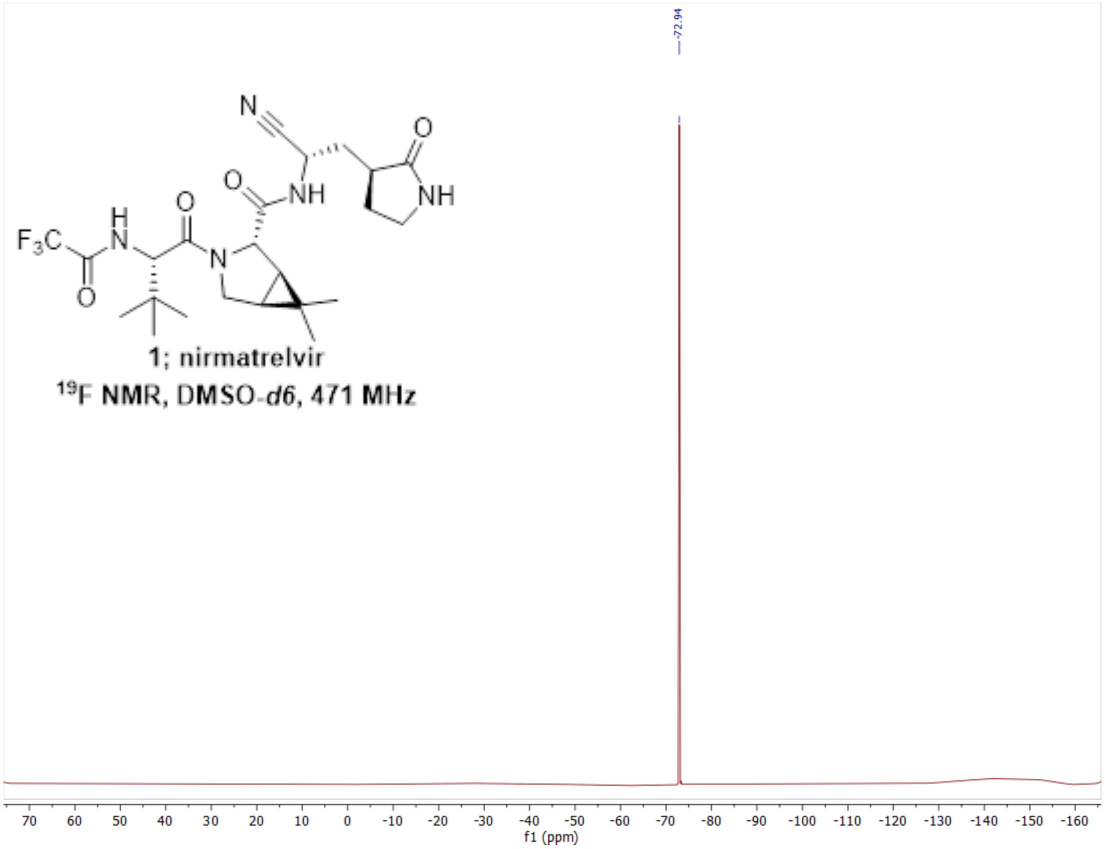

-72.94
N
O
NH
O
NH
H
O
F3C
N
N
O
1; nirmatrelvir
19F NMR, DMSO-d6, 471 MHz
70
60
50
40
30
20
10
0
-10
-20
-30
-40
-50
-60
-70
-80
-90
-100
-110
-120
-130
-140
-150
-160
f1 (ppm)

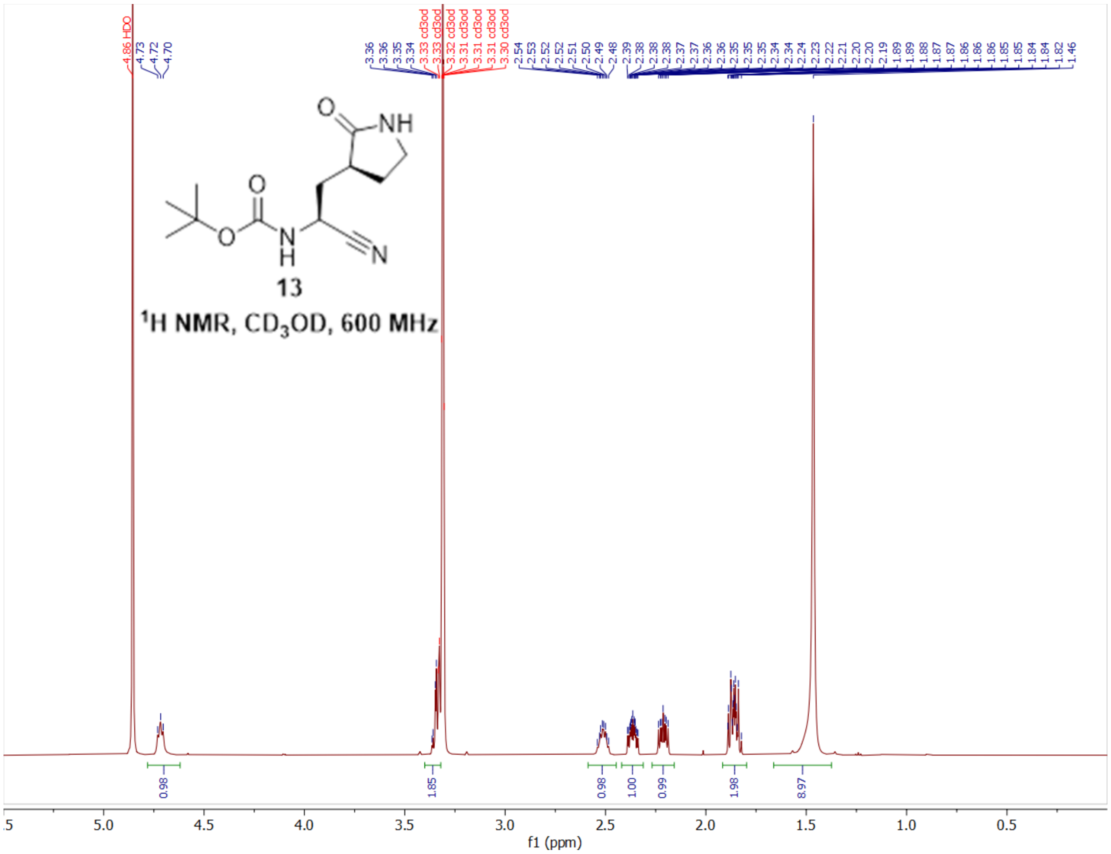
13
¹H NMR, CD₃OD, 600 MHz
f1 (ppm)

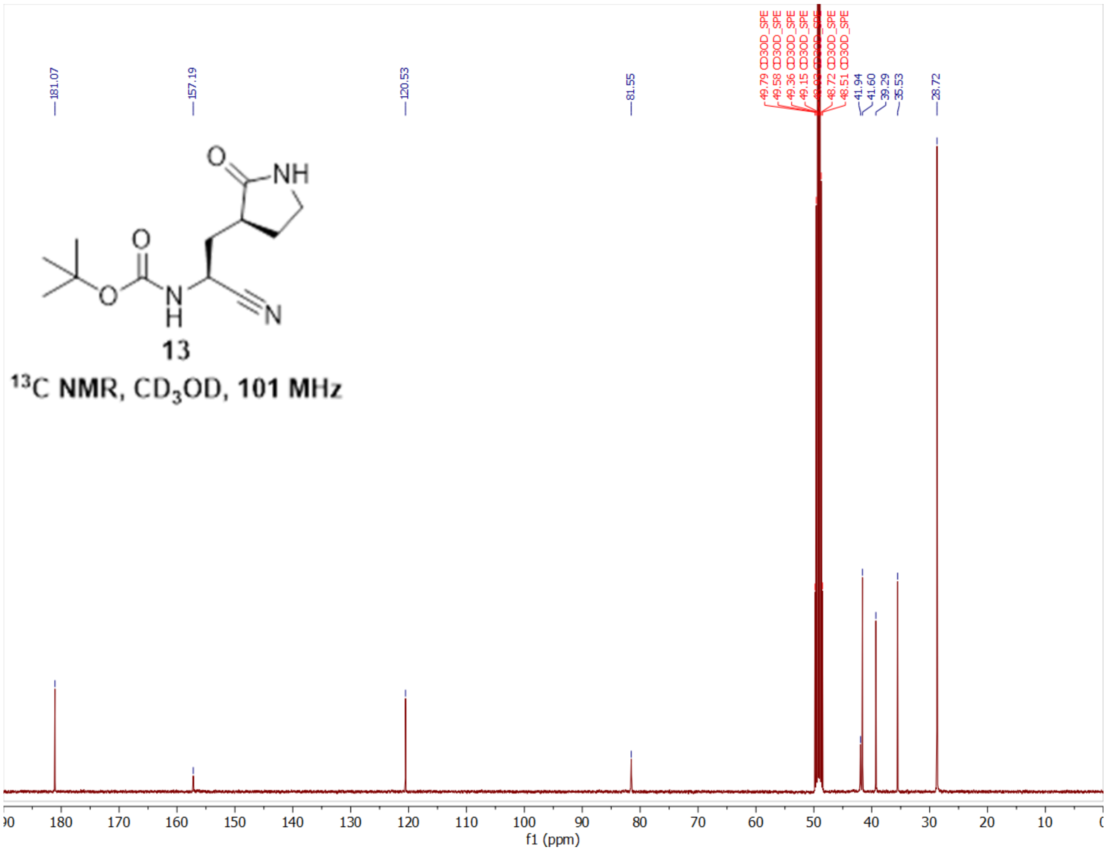
13
¹³C NMR, CD₃OD, 101 MHz
f1 (ppm)

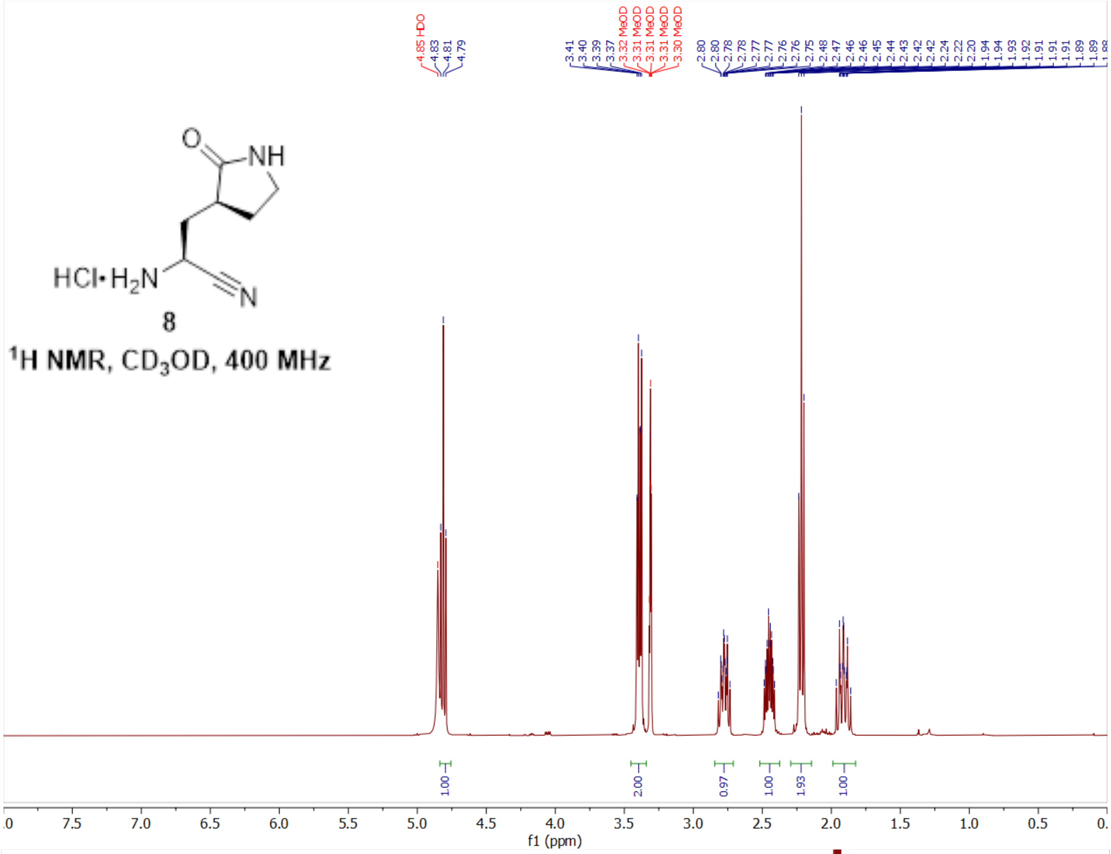
HCl•H2N
8
1H NMR, CD3OD, 400 MHz
4.85 HDO
3.32 MeOD
3.31 MeOD
3.30 MeOD
1.00
2.00
0.97
1.00
1.93
1.00
f1 (ppm)

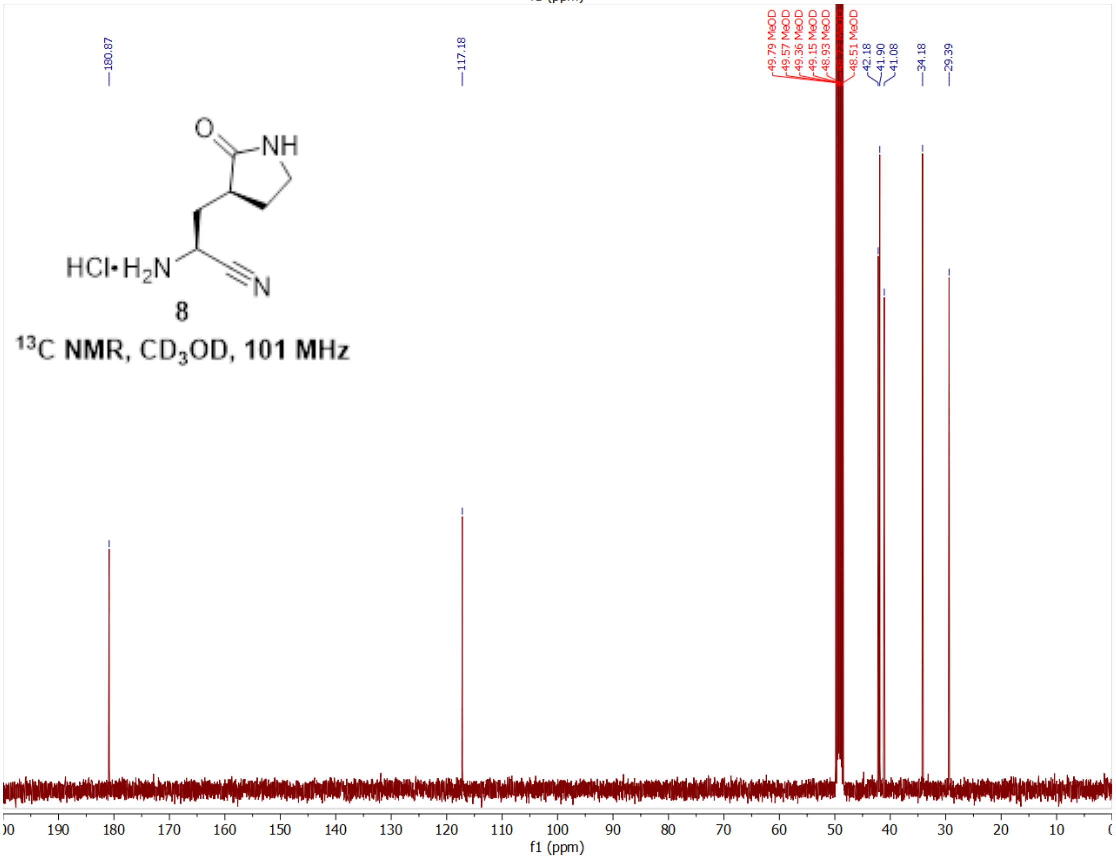
180.87
117.18
49.79 MeOD
49.57 MeOD
49.36 MeOD
49.15 MeOD
48.93 MeOD
48.51 MeOD
42.18
41.90
41.08
34.18
29.39
HCl•H2N
8
13C NMR, CD3OD, 101 MHz
f1 (ppm)
