# Supplementary material for: A sustainable synthesis of the SARS-CoV-2 Mpro inhibitor nirmatrelvir, the active ingredient in Paxlovid
Source: Commun Chem. 2022 Nov 21;5:156. doi: 10.1038/s42004-022-00758-5 (PMC9685088; doi:10.1038/s42004-022-00758-5)
Supplement: Supplementary file 4 — Description of Additional Supplementary Files [file 42004_2022_758_MOESM4_ESM.pdf]

Description of Additional Supplementary Files

File name: Supplementary Data 1

Description: Computational Studies

File name: Supplementary Data 2

Description: 1H, 13C, and 19F NMR spectra
